# Sex-based differences in growth-related IGF1 signaling in response to PAPP-A2 deficiency: comparative effects of rhGH, rhIGF1 and rhPAPP-A2 treatments

**DOI:** 10.1186/s13293-024-00603-5

**Published:** 2024-04-08

**Authors:** María del Mar Fernández-Arjona, Juan Antonio Navarro, Antonio Jesús López-Gambero, Marialuisa de Ceglia, Miguel Rodríguez, Leticia Rubio, Fernando Rodríguez de Fonseca, Vicente Barrios, Julie A. Chowen, Jesús Argente, Patricia Rivera, Juan Suárez

**Affiliations:** 1 Instituto de Investigación Biomédica de Málaga y Plataforma en Nanomedicina-IBIMA Plataforma BIONAND, Avenida Carlos Haya 82, Málaga, 29010 Spain; 2 Servicio de Neurología, Hospital Regional Universitario de Málaga, Málaga, 29010 Spain; 3 UGC Salud Mental, Hospital Regional Universitario de Málaga, Málaga, 29010 Spain; 4 University of Bordeaux, INSERM, Neurocentre Magendie, U1215, Bordeaux, 33000 France; 5 Departamento de Anatomía Humana, Medicina Legal e Historia de la Ciencia. Facultad de Medicina, Universidad de Málaga, Bulevar Louis Pasteur 32, Málaga, 29071 Spain; 6 Departments of Pediatrics & Pediatric Endocrinology, Hospital Infantil Universitario Niño Jesús, Avenida Menéndez Pelayo 65, Madrid, 28009 Spain; 7 La Princesa Research Institute, Madrid, 28009 Spain; 8 Department of Pediatrics, Universidad Autónoma de Madrid, Madrid, 28049 Spain; 9 Centro de Investigación Biomédica en Red Fisiología de la Obesidad y Nutrición (CIBEROBN), Instituto de Salud Carlos III, Madrid, 28029 Spain; 10 IMDEA Food Institute, CEI UAM & CSIC, Madrid, 28049 Spain

**Keywords:** Growth, Sexual dimorphism, Pappalysin-2, IGF1, GH, PI3K, mTOR, AMPK, Hypothalamus, Pituitary gland, Liver

## Abstract

**Background:**

Children with pregnancy-associated plasma protein-A2 (*PAPP-A2*) mutations resulting in low levels of bioactive insulin-like growth factor-1 (IGF1) and progressive postnatal growth retardation have improved growth velocity and height following recombinant human (rh)IGF1 treatment. The present study aimed to evaluate whether *Pappa2* deficiency and pharmacological manipulation of GH/IGF1 system are associated with sex-specific differences in growth-related signaling pathways.

**Methods:**

Plasma, hypothalamus, pituitary gland and liver of *Pappa2*^ko/ko^ mice of both sexes, showing reduced skeletal growth, and liver of these mice treated with rhGH, rhIGF1 and rhPAPP-A2 from postnatal day (PND) 5 to PND35 were analyzed.

**Results:**

Reduced body and femur length of *Pappa2*^ko/ko^ mice was associated with increases in: (1) components of IGF1 ternary complexes (IGF1, IGFBP5/*Igfbp5*, *Igfbp3*, *Igfals*) in plasma, hypothalamus and/or liver; and (2) key signaling regulators (phosphorylated PI3K, AKT, mTOR, GSK3β, ERK1/2 and AMPKα) in hypothalamus, pituitary gland and/or liver, with *Pappa2*^ko/ko^ females having a more prominent effect. Compared to rhGH and rhIGF1, rhPAPP-A2 specifically induced: (1) increased body and femur length, and reduced plasma total IGF1 and IGFBP5 concentrations in *Pappa2*^ko/ko^ females; and (2) increased *Igf1* and *Igf1r* levels and decreased *Ghr*, *Igfbp3* and *Igfals* levels in the liver of *Pappa2*^ko/ko^ females. These changes were accompanied by lower phospho-STAT5, phospho-AKT and phospho-ERK2 levels and higher phospho-AMPK levels in the liver of *Pappa2*^ko/ko^ females.

**Conclusions:**

Sex-specific differences in IGF1 system and signaling pathways are associated with *Pappa2* deficiency, pointing to rhPAPP-A2 as a promising drug to alleviate postnatal growth retardation underlying low IGF1 bioavailability in a female-specific manner.

**Supplementary Information:**

The online version contains supplementary material available at 10.1186/s13293-024-00603-5.

## Abstract (plain language summary)

Understanding the physiological role of pregnancy-associated plasma protein-A2 (PAPP-A2), a proteinase involved in the insulin-like growth factor-1 (IGF1) availability to regulate growth, could provide insight into new treatments for patients with short stature and skeletal abnormalities. Although progressive postnatal growth retardation in patients with *PAPP-A2* mutations can differ between males and females, we do not know the underlying differences in IGF1 system and signaling, and their response to treatment that contribute to growth improvement. The present study examines whether *Pappa2* deficiency and pharmacological administration of rhGH, rhIGF1 and rhPAPP-A2 are associated with sex-specific differences in IGF1 ternary complexes and IGF1 signaling pathways. Reduced body and femur length of *Pappa2*-deficient mice was associated with sex- and tissue-specific alteration of IGF ternary/binary complexes and IGF1 signaling pathways. rhPAPP-A2 treatment induced female-specific increase in body and femur length and reduction in IGF ternary/binary complexes through STAT5-AKT-ERK2-AMPK signaling pathways in liver. The involvement of PAPP-A2 in sex-based growth physiology supports the use of promising drugs to alleviate postnatal growth retardation underlying low IGF1 bioavailability in a female-specific manner.

**Supplementary Information:**

The online version contains supplementary material available at 10.1186/s13293-024-00603-5.

## Highlights

**•**Postnatal growth retardation induced by pregnancy-associated plasma protein-A2 (*PAPP-A2*) mutations can be palliated after recombinant human (rh)IGF1 treatment.
**•***Pappa2* deficiency reduced body and femur length and induced sex- and tissue-specific changes of IGF ternary/binary complexes and IGF1 signaling pathways
**•**rhPAPP-A2 treatment induced female-specific increases in body and femur length and reductions in IGF ternary/binary complexes via AKT-AMPK pathways in the liver.
**•**The involvement of PAPP-A2 in sex-based growth physiology supports the use of promising drugs to alleviate postnatal growth retardation in a female-specific manner.

**Supplementary Information:**

The online version contains supplementary material available at 10.1186/s13293-024-00603-5.

## Background

Homozygous mutations in pregnancy-associated plasma protein-A2 (PAPP-A2 or pappalysin-2) produce a syndrome characterized by progressive postnatal growth retardation, loss or a reduction of PAPP-A2 function, high circulating levels of insulin-like growth factor (IGF)-1 bound in ternary/binary complexes with specific IGF binding proteins (IGFBP3 or IGFBP5) and acid-labile subunit (IGFALS), and decreased concentrations of free IGF1 [1, 2]. Patients with *PAPP-A2* mutations have mild to moderate short stature and skeletal abnormalities including thin long bones and decreased bone mineral density [3, 4] and when treated with recombinant human IGF1 (rhIGF1) both growth [5] and bone mineral density [6] improve.

PAPP-A2 is a metalloproteinase that exerts specific proteolytic activity on IGFBP3 and IGFBP5 and regulates IGF1 dissociation from ternary/binary complexes, and thus IGF1 bioavailability [7, 8]. Inhibitors of PAPP-A2 activity, staniocalcin-1 (STC1) and staniocalcin-2 (STC2), block IGF release from IGFBP3 and IGFBP5, bringing another level of complexity to the control of IGF1 bioavailability [9–11]. In fact, serum concentrations of PAPP-A2 and the free/total IGF1 ratio, but not stanniocalcins, decrease progressively in an age-specific manner [12]. Synthesis and secretion of IGF1, among other IGF-related components such as IGF2, IGFBP3, IGFBP5 and IGFALS, are stimulated by the actions of growth hormone (GH) in the liver [3, 4]. IGF1 primarily circulates in ternary complexes (75–80% of total IGF1) with the high affinity IGFBP3 or IGFBP5 and IGFALS, which increases its half-life and modulates its concentrations and bioavailability [13–15], with only approximately 1% being free or unbound [16]. IGF1 is liberated from these binding proteins by PAPP-A2 in close proximity to target tissues that contain the IGF1 receptor (IGF1R).

PAPP-A2 actions increase the levels of free IGF1 and the subsequent availability of this growth factor to stimulate its receptor both centrally and peripherally that, in conjunction with an adequate nutritional supply, is predominately responsible for activating and/or inhibiting key signaling pathways and transcriptional factors that modulate growth. IGF1R intracellular signaling pathways include phosphoinositide 3-kinase (PI3K)/ protein kinase B (PKB, also known AKT), mitogen-activated protein kinases/extracellular signal-regulated kinase (MAPK/ERK), AMP-activated protein kinase (AMPK), mammalian target of rapamycin (mTOR) and glycogen synthase kinase-3 (GSK3), among others [17–19].

Preclinical studies using knock-out (ko/ko) and knock-in mice with targeted disruption of PAPP-A2 [20–23] have emerged as good experimental models to recapitulate previously described physiological growth derangements related to short stature in patients with PAPP-A2 deficiency [4]. However, more information is needed to identify specific signaling mechanisms affected by low IGF1 availability, to elucidate whether impairment of postnatal longitudinal growth caused by increased IGF1 ternary/binary complexes and reduced IGF1 availability are different between the sexes, and whether pharmacological manipulation of the GH/IGF1 system induces sex-specific differences in growth-related signaling pathways. To this end, plasma, hypothalamus, pituitary gland and liver of constitutive *Pappa2*^ko/ko^ mice of both sexes with reduced skeletal growth and impaired bone composition, as previously described by our group and others [23, 24], as well as the liver of these mice daily treated with rhGH, rhIGF1 and rhPAPP-A2 from postnatal day (PND) 5 to PND35, were used to assess sex-specific differences in the expression of the main components of IGF1 ternary complexes and main mediators of IGF1 and GH intracellular signaling pathways (JAK2, STAT3/5, PI3K, AKT, mTOR, GSK3β, ERK1/2 and AMPK).

## Methods

### Ethics statement

All procedures were conducted in strict adherence to ARRIVE guidelines and the principles of laboratory animal care (National Research Council, Neuroscience CoGftUoAi, Research B, 2003) following the European Community Council Directive (63/2020/UE) and the Spanish Directive (RD53/2013), and were approved by the Ethical Committee of the University of Málaga (Exp. 46-2019-A). Special care was taken to minimize suffering and the number of animals required to perform the procedures.

### Animals

Male and female mice with constitutive deletion of the *Pappa2* gene (*Pappa2*^ko/ko^) and littermate controls (*Pappa2*^wt/wt^) were generated as previously described [21]. *Pappa2* lines (C57BL/6 genetic background) were maintained by crossing heterozygous transgenic mice. The mice were housed in the Animal Center for Experimentation at the University of Malaga with free access to standard rodent chow (3.02 Kcal/g) and tap water under standardized conditions: 22 ± 1 ºC of room temperature, relative humidity of 40 ± 5%, and a 12-h reverse light/dark cycle (lights were turned off at 8:00 am) with dawn/sunset effect. Mice were genotyped using ear clip tissue as previously described [8]. Body length (including tail) was monitored periodically from weaning (approximately PND23) to adulthood (8 months of age).

### Treatments

Recombinant human GH protein (rhGH; cat nº: ABIN803848; antibodies-online GmbH, Aachen, Germany), recombinant human IGF1 protein (rhIGF1; cat. nº: 100 − 11; PeproTech, Inc., Rocky Hill, NJ, USA), and recombinant human PAPP-A2 protein (rhPAPP-A2; cat. nº: abx068418; Abbexa LTD, Cambridge; UK) were dissolved in sterile saline (0.9% NaCl). The rhIGF-1 and rhGH solutions were administered at a dose of 0.3 mg/kg/day, while rhPAPP-A2 was administered at a dose of 0.1 mg/kg/day. Saline, rhGH, rhIGF1 and rhPAPP-A2 were administered by daily interscapular subcutaneous injections, in a volume of 10 mL/kg body weight, for 30 days from postnatal day (PND) 5 to PND35. Doses were selected according to previous studies [25, 26] and previous data from our group. To avoid hypoglycemic crises, mice received hormonal treatment during a non-fasting period, 2 h after the onset of the dark cycle. All mice were sacrificed 24 h after the last administration with 12 h fasting.

### Sample collection

Mice with 35 days of age and 8 months of age were sacrificed by decapitation after administration of Equitesin® (3 ml/kg i.p.; chloral hydrate 2.1 g, sodium pentobarbital 0.46 g, MgSO_4_ 1.06 g, propylene glycol 21.4 ml, ethanol (90%) 5.7 ml, H_2_O 3 ml). Blood samples were collected in tubes containing 6% EDTA and protease inhibitors (Roche cOmplete tablets), and centrifuged (1500 g for 10 min at 4ºC). The plasma was kept at -80 ºC until hormone analysis. Bones (femurs and tibias) were weighed, and their length was measured with a caliper. Brain, pituitary gland and samples from the left lateral lobe of the liver were extracted, flash-frozen in liquid nitrogen and stored at -80 ºC until used for mRNA and protein analysis. The hypothalamus, containing the median eminence, arcuate nucleus and ventromedial nucleus, were precisely dissected according to the mouse brain atlas of Paxinos and Franklin [27].

### Hormone analyses

Plasma levels of IGF1 and IGFBP5 were determined by using commercial enzyme-linked immunosorbent assay (ELISA) kits: Mouse/rat IGF1 DuoSet® ELISA (cat. no. DY791, R&D Systems, Abingdon, UK) and mouse IGFBP5 DuoSet® ELISA (cat. no. DY578, R&D Systems). Plasma and pituitary levels of growth hormone (GH) were determined by using the Mouse Pituitary Magnetic Bead Panel 96-well Plate Assay (cat. no. MPTMAG-49 K, Millipore® Map, Merck). Plates were run on a Bio-Plex MAGPIX™ Multiplex Reader with Bio-Plex manager™ MP Software (Luminex, Austin, TX, USA). Hormone concentrations were expressed in ng/mL or pg/mL, and detection limits were 31.3 (IGF1), 125 (IGFBP5) and 3.9 (GH) pg/mL.

### RNA isolation and RT-qPCR analysis

Real-time PCR (TaqMan, ThermoFisher Scientific, Waltham, MA, USA) was performed as previously described [28] by using specific sets of primer probes from TaqMan® Gene Expression Assays (Supplementary Table S1). Hypothalamus, pituitary and liver samples were homogenized on ice and total RNA was extracted using the Trizol® method according to the manufacturer’s instructions (ThermoFisher Scientific). RNA samples were isolated with RNAeasy minielute cleanup kits, including digestion with DNase I column (Qiagen), and quantified using a spectrophotometer to ensure A260/280 ratios of 1.8-2.0. After the reverse transcript reaction from 1 µg of RNA, a quantitative real-time reverse transcription polymerase chain reaction (qPCR) was performed in a CFX96TM Real-Time PCR Detection System (Bio-Rad, Hercules, CA, USA) and the FAM dye-labeled format for the TaqMan® Gene Expression Assays (ThermoFisher Scientific). Melting curve analysis was performed to ensure that only a single product was amplified. After analysis of several reference genes, the values obtained from the samples were normalized to *Actb* levels, which did not vary significantly between the experimental groups.

### Western blotting analysis

Western blotting was performed as previously described [29]. Briefly, hypothalamus, pituitary and liver samples were homogenized in 500 µL of ice-cold lysis buffer containing Triton X-100, 1 M 4-(2-hydroxyethyl)-1-piperazineethanesulfonic acid (HEPES), 0.1 M ethylenediaminetetraacetic acid (EDTA), sodium pyrophosphate, sodium fluoride (NaF), sodium orthovanadate (NaOV) and protease inhibitors (Roche cOmplete tablets) using a tissue-lyser system (Qiagen). After centrifuging at 26,000 *g* for 30 min at 4ºC, the supernatant was transferred to a new tube. The Bradford method was used to measure the protein concentration of the samples. An amount of 30 µg of each total protein sample was separated on 4–12% polyacrylamide gradient gels. The gels were then transferred onto nitrocellulose membranes (Bio-Rad Laboratories, Hercules, CA, USA) and stained with Ponceau red. Membranes were blocked in TBS-T (50 mM Tris-HCl pH 7.6, 200 mM NaCl, and 0.1% Tween 20) with 2% albumin fraction V from bovine serum (BSA, Roche, Mannheim, Germany) for 1 h at room temperature. The primary antibodies to the proteins of interest (Supplementary Table S2) were incubated overnight at 4ºC. Mouse γ–adaptin and β–actin were used as reference proteins from the same membrane when appropriate. After several washes in TBS-T containing 1% Tween 20, an HRP-conjugated anti-rabbit or anti-mouse IgG (H + L) secondary antibody (Promega, Madison, WI, USA), diluted 1:10,000, was added followed by incubation for 1 h at room temperature. After extensive washing in TBS-T, the membranes were incubated for 1 min with the Western Blotting Luminol Reagent kit (Santa Cruz Biotechnology, Santa Cruz, CA, USA), and the specific protein bands were visualized and quantified by chemiluminescence using a ChemiDocTM MP Imaging System (Bio-Rad, Barcelona, Spain). Bands were quantified by densitometric analysis using ImageJ software (http://imagej.nih.gov/ij). The results are presented as the ratio of total target protein to γ–adaptin or β–actin, the ratio of phosphorylated target protein to their total target protein, and the tyrosine/serine phosphorylated target protein ratio. For more information, see Supplementary Figures S1-4.

### Statistical analysis

Data were analyzed using Graph-Pad Prism 7.0 software. Values are expressed as mean ± SEM for each experimental group (*n* = 8–10 for auxological, hormonal and mRNA analysis; *n* = 6 for protein analysis). The significance of difference within and between groups was analyzed by a three-way analysis of variance (ANOVA), whose factors were treatment (rhGH, rhIGF1 or rhPAPP-A2 vs. saline), genotype (*Pappa2*^wt/wt^*vs. Pappa2*^ko/ko^), and sex (males vs. females), followed by Tukey post-hoc test for multiple comparisons. Given the sex differences found in this experiment, two-way ANOVA was also carried out (genotype and treatment as factors). A *p*-value less than 0.05 was considered statistically significant.

## Results

### Effects of *Pappa2* deletion on body and bone length

**Fig. 1:**
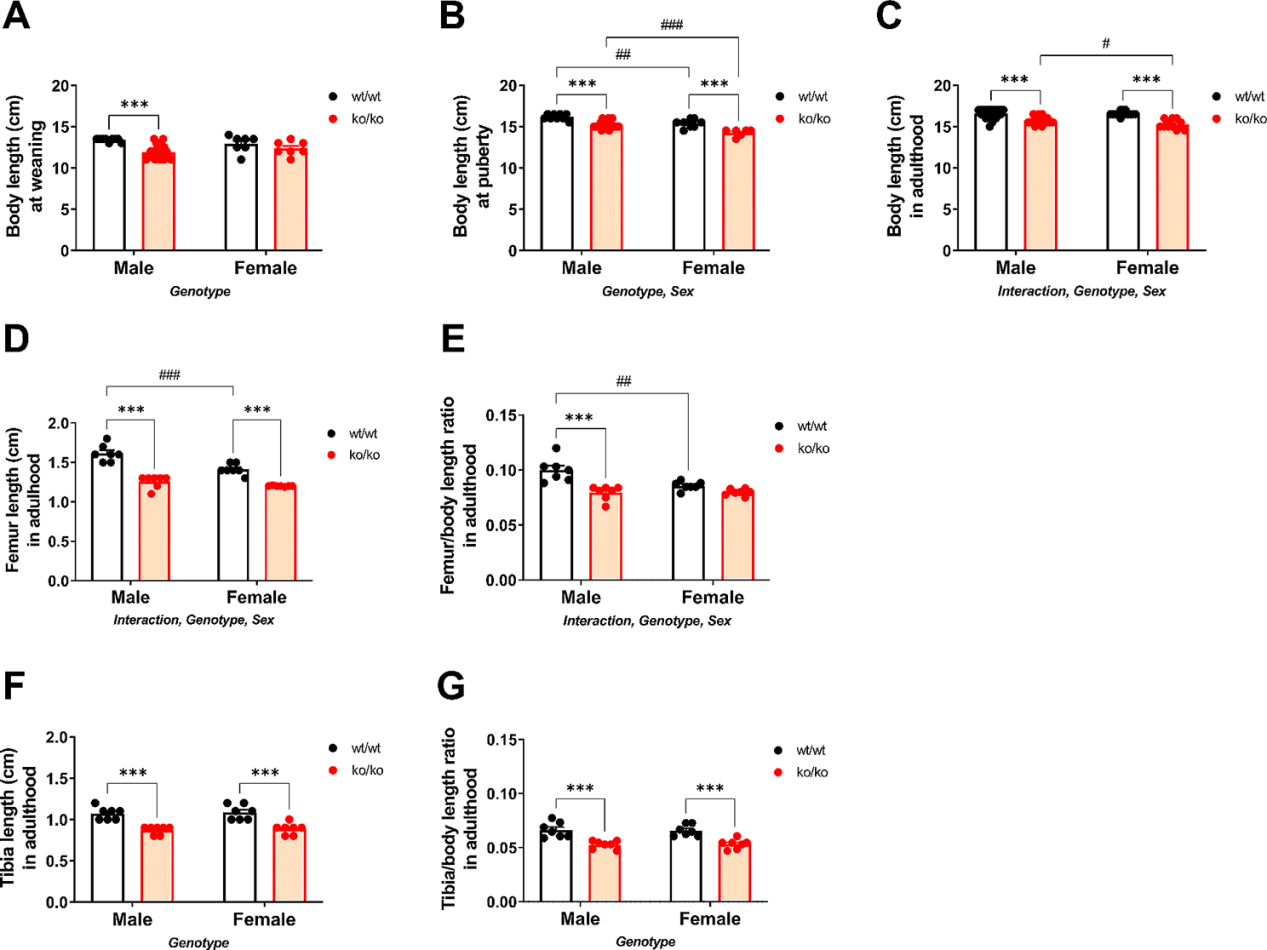
Auxological parameters of body and bone (femur and tibia) length in *Pappa2*^ko/ko^ and *Pappa2*^wt/wt^ mice (males and females) at weaning (**A**), puberty (**B**) and adulthood (**C-G**). Data are represented as mean ± S.E.M (*N* = 8–10 per experimental group). Two-way ANOVA and Tukey-corrected tests: ***p < 0.001 versus respective wt/wt males and females; ^#/##/###^*p* < 0.05/0.01/0.001 versus respective wt/wt and ko/ko males

Since previous studies reported that *Pappa2* deletion reduces IGF1 bioavailability and affects postnatal growth [4], here we first analyzed the body and bone length of *Pappa2*^ko/ko^ mice (Fig. 1). A significant genotype effect on the body length at weaning (PND21) was found (F_1,44_ = 15.95, *p* < 0.001), with ko/ko males having an overall decrease in body length compared to wt/wt males (***p < 0.001; Fig. 1A). At puberty (PND40), the significant effect of genotype on body length remained (F_1,44_ = 56.01, *p* < 0.001), with ko/ko males and females being shorter than their respective wt/wt groups (***p < 0.001; Fig. 1B). We also observed that wt/wt and ko/ko females were shorter than same genotype males (^##/###^*p* < 0.01/0.001; sex effect: F_1,44_ = 36.95, *p* < 0.001; Fig. 1B). In adulthood (8 months of age), there was a significant interaction between genotype and sex on body length (F_1,44_ = 4.05, *p* < 0.05), suggesting that PAPP-A2 deficiency reduced body length differentially in male and female mice with *Pappa2* deletion. In addition, effects of genotype (F_1,44_ = 90.47, *p* < 0.001) and sex (F_1,41_ = 3.99, *p* = 0.05) were found on body length in adulthood, with body length in ko/ko males and females being reduced compared to their respective wt/wt groups (***p < 0.001; Fig. 1C), and with wt/wt and ko/ko females being shorter than the respective male groups (^#^*p* < 0.05; Fig. 1C).

In adulthood (8 months of age), the length of long bones (femurs and tibias) was also affected, with a significant interaction between genotype and sex found in femur length (F_1,28_ = 6.37, *p* < 0.05) and femur length relative to body length (F_1,28_ = 8.35, *p* < 0.01), suggesting that PAPP-A2 deficiency reduced femur length differentially in male and female mice with *Pappa2* deletion. In addition, effects of genotype (F_1,28_ > 27.87, *p* < 0.001) and sex (F_1,28_ > 7.65, *p* < 0.01) were observed on femur length and femur length relative to body length (Fig. 1D, E), with ko/ko males and females having shorter femurs than their respective wt/wt groups (***p < 0.001), and with wt/wt females having shorter femurs than wt/wt males (^##/###^*p* < 0.01/0.001). No significant interaction was observed on tibia length. A significant effect of genotype was found on tibia length (F_1,28_ = 53.45, *p* < 0.001) and tibia length relative to body length (F_1,28_ = 43.52, *p* < 0.001), with ko/ko males and females having a shorter tibia length than their respective wt/wt groups (***p < 0.001; Fig. 1F, G).

In summary, we observed a sex-specific effect of *Pappa2* deletion on body and femur length, with ko/ko females having a more prominent reduction in body length and ko/ko males a more prominent reduction in femur length.

### Effects of *Pappa2* deletion on circulating and/or pituitary concentrations of IGF1, IGFBP5 and GH

**Fig. 2:**
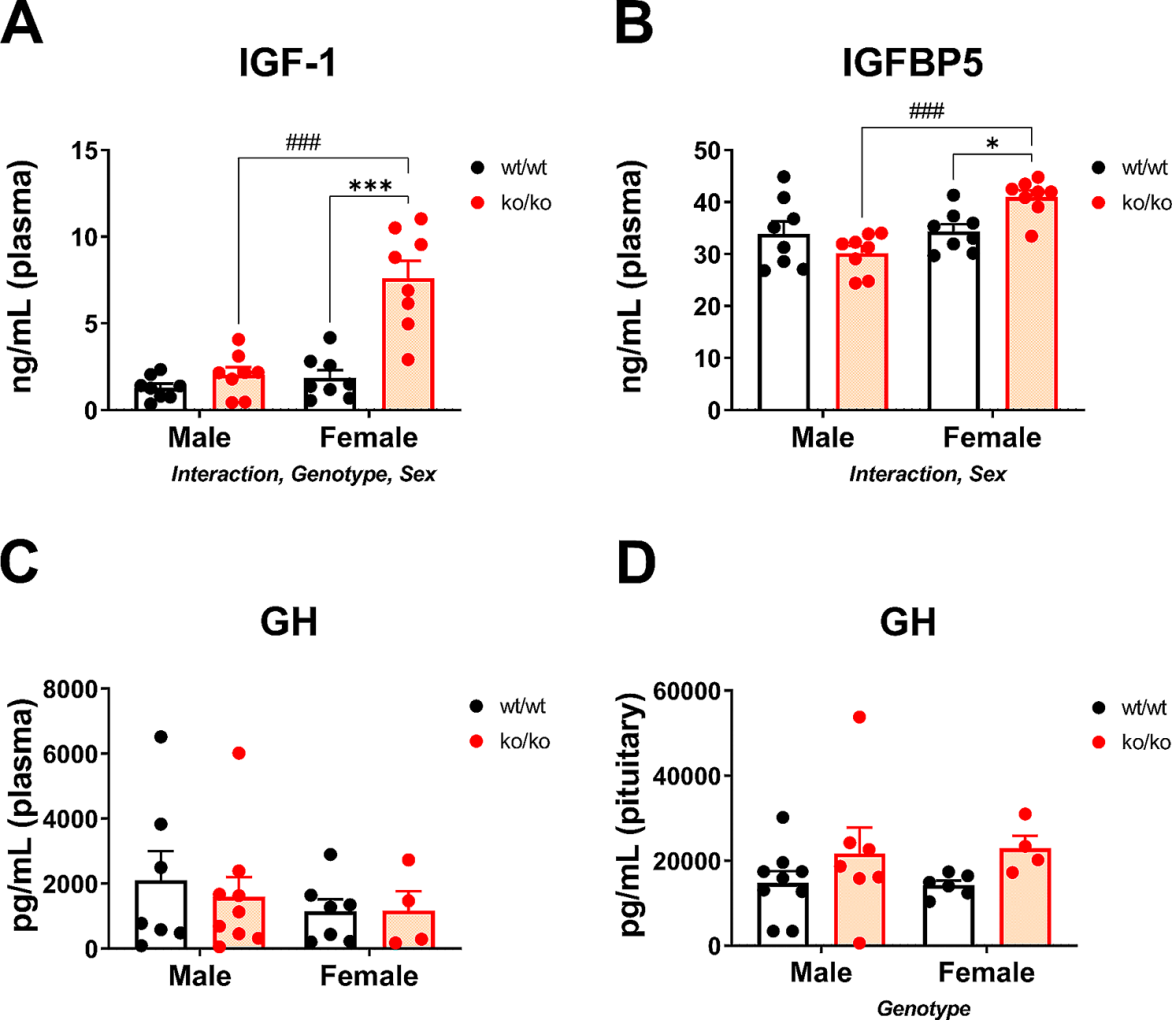
Hormone concentrations in the plasma and pituitary gland of *Pappa2*^ko/ko^ and *Pappa2*^wt/wt^ mice (males and females) in adulthood. Concentrations of total IGF1 (**A**), IGFBP5 (**B**) and GH in plasma (**C**), and GH in pituitary gland (**D**) are represented as mean ± S.E.M (*n* = 8–10 per experimental group). Two-way ANOVA and Tukey-corrected tests: *^/^***p < 0.05/0.001 versus respective wt/wt males and females; ^###^*p* < 0.001 versus respective wt/wt and ko/ko males

Since body and bone length deficits could be consequent to hormonal alterations, we assessed plasma and/or pituitary concentrations of IGF1, IGFBP5 and GH in *Pappa2*^ko/ko^ mice (Fig. 2). A significant interaction between genotype and sex was found in the circulating levels of IGF-1 (F_1,28_ = 17.20, *p* < 0.001) and IGFBP5 (F_1,28_ = 9.22, *p* < 0.01), suggesting that the plasma levels of both IGF-1 and IGFBP5 were differentially increased in male and female mice with *Pappa2* deletion (Fig. 2A, B). A genotype effect was found on the circulating levels of IGF-1 (F_1,28_ = 29.14, *p* < 0.0001), with an overall increase in IGF-1 levels in ko/ko females compared to wt/wt females (***p < 0.001; Fig. 2A). We also found sex effects on the circulating levels of IGF1 (F_1,28_ = 25.85, *p* < 0.0001) and IGFBP5 (F_1,28_ = 11.65; *p* < 0.01), with the ko/ko female group having significantly more IGF1 and IGFBP5 that the ko/ko male group (^###^*p* < 0.001; Fig. 2A, B). No effects or interaction were found on the plasma levels of GH (Fig. 2C). Additional hormonal factors were also evaluated in plasma (Supplementary Figures S5A-F).

Since GH is secreted in a pulsatile fashion and a single plasma measurement does not necessarily reflect its total integrated levels, we also analyzed GH concentrations in *Pappa2*^ko/ko^ mice at the pituitary level (Fig. 2D). An effect of genotype was found on pituitary GH content (F_1,28_ = 3.99, *p* < 0.05), which was most likely due to increased levels of GH in the pituitary gland of the ko/ko males and females (Fig. 2D). Additional hormonal factors were also evaluated in pituitary gland (Supplementary Figures S5G, H).

In summary, there was a sex-specific effect of *Pappa2* deletion on total IGF1 and IGFBP5 concentrations in plasma, with a significant increase in ko/ko females. *Pappa2* deficiency was also accompanied by increased pituitary GH concentrations in both sexes.

### Effects of *Pappa2* deletion on gene expression of the hypothalamic IGF1 system

**Fig. 3:**
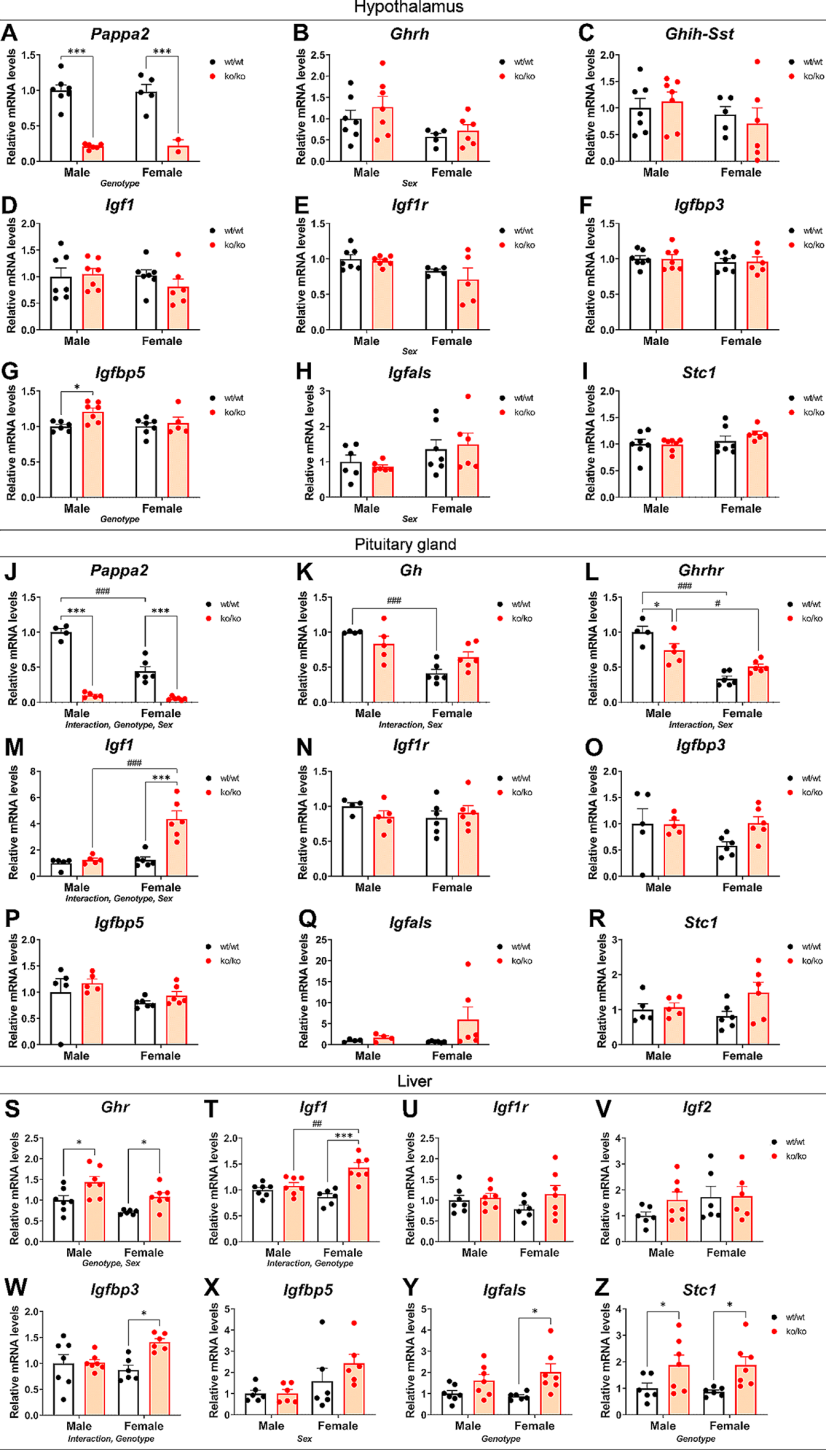
IGF1 system gene expression in the hypothalamus, the pituitary gland and the liver of *Pappa2*^ko/ko^ and *Pappa2*^wt/wt^ mice (males and females) in adulthood. Hypothalamus: relative *Pappa2* (**A**), *Ghrh* (**B**), *Ghih-Sst* (**C**), *Igf1* (**D**), *Igf1r* (**E**), *Igfbp3* (**F**), *Igfbp5* (**G**), *Igfals* (**H**) and *Stc1* (**I**) mRNA levels. Pituitary gland: relative *Pappa2* (**J**), *Gh* (**K**), *Ghrhr* (**L**), *Igf1* (**M**), *Igf1r* (**N**), *Igfbp3* (**O**), *Igfbp5* (**P**), *Igfals* (**Q**) and *Stc1* (**R**) mRNA levels. Liver: relative *Ghr* (**S**), *Igf1* (**T**), *Igf1r* (**U**), *Igf2* (**V**), *Igfbp3* (**W**), *Igfbp5* (**X**), *Igfals* (**Y**) and *Stc1* (**Z**) mRNA levels. Data are represented as mean ± S.E.M (*n* = 8–10 per experimental group). See Table S1 for additional information. Two-way ANOVA and Tukey-corrected tests: *^/^***p < 0.05/0.001 versus respective wt/wt males and females; ^##/###^*p* < 0.01/0.001 versus respective wt/wt and ko/ko males

We analyze whether *Pappa2* insufficiency affects gene expression of the main components of the IGF1 system in the hypothalamus (Fig. 3A-I). An effect of genotype was found on the mRNA levels of *Pappa2* (F_1,28_ = 123.2, *p* < 0.001), with an overall decrease in *Pappa2* mRNA in ko/ko males and females compared to their respective wt/wt groups (***p < 0.001; Fig. 3A). There was a sex effect on the mRNA levels of *Ghrh* (F_1,28_ = 6.649, *p* < 0.05), with females having lower *Ghrh* mRNA levels than males (Fig. 3B). No interaction or main effects were found in *Ghih* expression (Fig. 3C).

No interaction or main effects were found in *Igf1* expression (Fig. 3D), but a sex effect was found on the mRNA levels of *Igf1r* (F_1,28_ = 7.547, *p* < 0.05), with females having lower *Igf1r* mRNA levels compared to males (Fig. 3E). No interaction or main effects were found in *Igfbp3* expression (Fig. 3F). An effect of genotype (F_1,28_ = 5.681, *p* < 0.05) was found on the mRNA levels of *Igfbp5*, with ko/ko males having higher levels compared to wt/wt males (*p < 0.05; Fig. 3G). There was a sex effect on the mRNA levels of *Igfals* (F_1,28_ = 4.445, *p* < 0.05), with females having higher mRNA levels compared to males (Fig. 3H). No main effects or interaction between factors were found in the *Stc1* mRNA levels (Fig. 3I).

Thus, there were sex specific effects of *Pappa2* deletion on the hypothalamic IGF system with an increase in *Igfbp5* and *Igfals* mRNA levels in ko/ko males and decreased *Ghrh* and *Igf1r* mRNA levels in females.

### Effects of *Pappa2* deletion on gene expression of the pituitary IGF1 system

We also analyzed the gene expression of the main components of the IGF1 system in the pituitary gland of *Pappa2*^ko/ko^ mice (Fig. 3J-R). A significant interaction between sex and genotype (F_1,28_ = 34.22; *p* < 0.001), and main effects of sex (F_1,28_ = 48.96; *p* < 0.001) and genotype (F_1,28_ = 219.0, *p* < 0.001) were found on the mRNA levels of *Pappa2*, with an overall decrease in ko/ko male and female mice compared to respective wt/wt mice (***p < 0.001; Fig. 3J), and lower levels of *Pappa2* mRNA in wt/wt females compared to wt/wt males (^###^*p* < 0.001; Fig. 3J). An interaction between sex and genotype (F_1,28_ > 8.177, *p* < 0.01) and a sex effect (F_1,28_ > 31.88, *p* < 0.001) were found on the mRNA levels of both *Gh* and *Ghrhr.* Multiple comparison tests showed lower levels of *Gh* and *Ghrh* in wt/wt females than wt/wt males (^###^*p* < 0.001; Fig. 3K, L). We also found lower *Ghrh* levels in the pituitary gland of ko/ko males compared to wt/wt males (*p < 0.05; Fig. 3L).

A significant interaction between sex and genotype (F_1,28_ = 17.01; *p* < 0.001), and main effects of sex (F_1,28_ = 23.47; *p* < 0.001) and genotype (F_1,28_ = 23.75; *p* < 0.001) were found on the mRNA levels of *Igf1*. Multiple comparison tests indicated overall higher levels if *Igf1* mRNA in ko/ko females compared to wt/wt females and ko/ko males (***^/###^*p* < 0.001; Fig. 3M). No interaction or effects between factors were found in the pituitary mRNA levels of *Igf1r, Igfbp3, Igfbp5, Igfals* or *Stc1* (Fig. 3N-R).

In summary, we observed a sex-specific effect of *Pappa2* deletion on pituitary *Gh*, *Ghrhr* and *Igf1* mRNA levels, with lower *Ghrhr* expression in ko/ko males and higher *Igf1* expression in ko/ko females.

### Effects of *Pappa2* deletion on gene expression of the liver IGF1 system

Since changes in PAPP-A2 activity can also affect the liver, we analyzed the gene expression of the main components of the IGF1 system in the liver of *Pappa2*^ko/ko^ mice (Fig. 3S-Z). Main effects of sex (F_1,28_ = 8.932, *p* < 0.01) and genotype (F_1,28_ = 13.87, *p* < 0.01) were found on the mRNA levels of *Ghr*, with an overall increase in *Ghr* mRNA in ko/ko male and female groups compared to their respective wt/wt groups (*p < 0.05; Fig. 3S).

A significant interaction between sex and genotype was found in the mRNA levels of *Igf1* (F_1,23_ = 9.216, *p* < 0.01), suggesting that the liver *Igf1* mRNA levels were differentially altered in male and female mice with *Pappa2* deletion (Fig. 3T). We also found a genotype effect on the mRNA levels of *Igf1* (F_1,28_ = 16.38, *p* < 0.001). Multiple comparison analysis indicated that ko/ko females showed higher mRNA levels of *Igf1* than wt/wt females (***p < 0.001) and ko/ko males (^##^*p* < 0.01; Fig. 3T). No interaction or effects were found on the mRNA levels of *Ifg1r* or *Igf2* (Fig. 3U, V).

An interaction between sex and genotype (F_1,28_ = 5.518, *p* < 0.05), and an effect of genotype (F_1,28_ = 6.400, *p* < 0.05) on the mRNA levels of *Igfbp3* were found, with ko/ko females having higher *Igfbp3* expression compared to wt/wt females (*p < 0.05; Fig. 3W). An effect of sex was found on the mRNA levels of *Igfbp5* (F_1,28_ = 6.59; *p* < 0.05), with females having higher expression (Fig. 3X). An effect of genotype was found on the mRNA levels of *Igfals* (F_1,28_ = 10.52; *p* < 0.01), with an increase in *Igfals* expression in ko/ko females compared to wt/wt females (*p < 0.05; Fig. 3Y). A genotype effect was also found in the mRNA level of *Stc1* (F_1,28_ = 11.00, *p* < 0.01), with ko/ko males and females expressing significantly more *Stc1* than their respective wt/wt groups (*p < 0.05; Fig. 3Z). *Pappa2* mRNA was undetected in the liver of all experimental groups analyzed.

Summarizing, we found a sex-specific effect of *Pappa2* deletion on liver *Igf1* and *Igfbp3* mRNA levels, with higher mRNA expressions in ko/ko females. *Pappa2* deficiency was also accompanied by increased liver *Ghr*, *Igfals* and *Stc1* mRNA levels in both sexes.

### Effects of *Pappa2* deletion on IGF1 signaling pathways in the hypothalamus

**Fig. 4:**
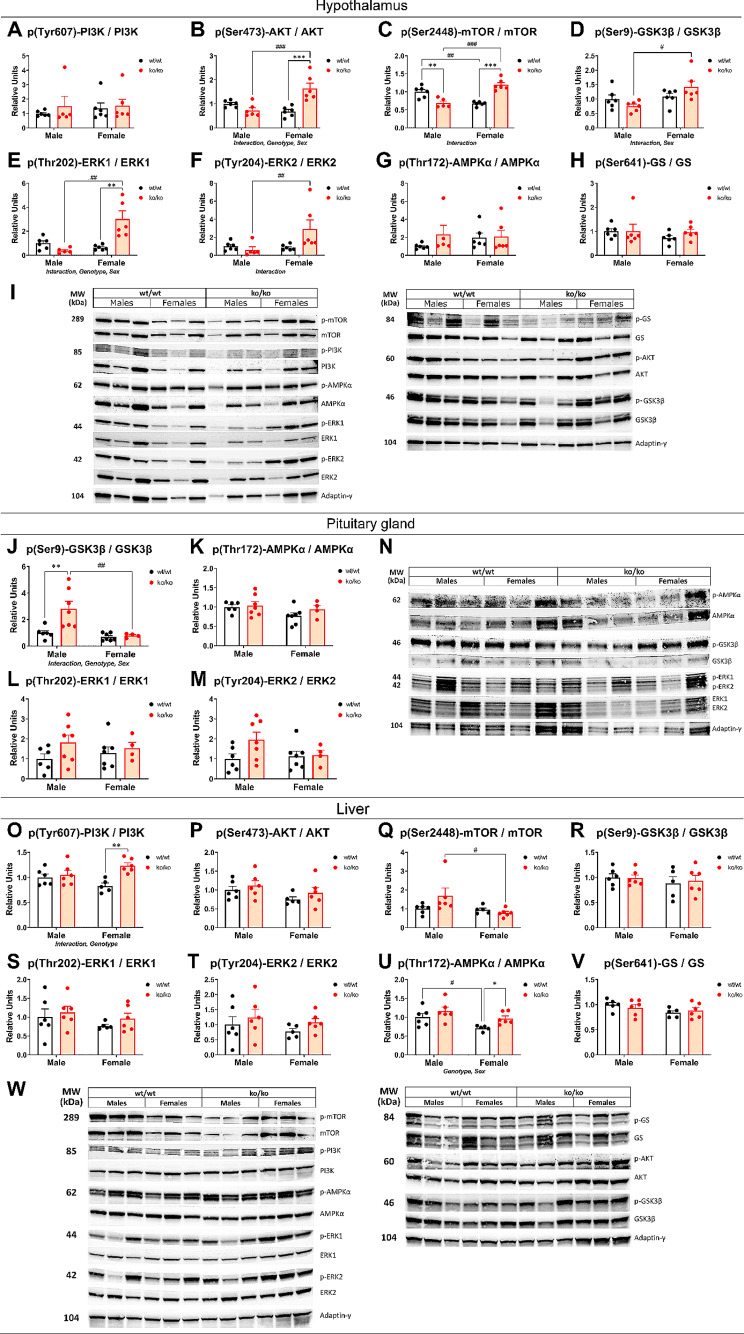
Key sensors of IGF1 signaling pathways in the hypothalamus, the pituitary gland and the liver of *Pappa2*^ko/ko^ and *Pappa2*^wt/wt^ mice (males and females) in adulthood. Hypothalamus: phosphorylated forms of PI3K (**A**), AKT (**B**), mTOR (**C**), GSK3β (**D**), ERK1 (**E**), ERK2 (**F**), AMPKα (**G**) and GS (**H**). Pituitary gland: phosphorylated forms of GSK3β (**J**), AMPKα (**K**), ERK1 (**L**) and ERK2 (**M**). Liver: phosphorylated forms of PI3K (**O**), AKT (**P**), mTOR (**Q**), GSK3β (**R**), ERK1 (**S**), ERK2 (**T**), AMPKα (**U**) and GS (**V**). Data are represented as mean ± S.E.M (*n* = 6 per experimental group). Representative immunoblots (**I, N** and **W**) are also shown. See Table S2 and Figures S1-S3 for additional information. Two-way ANOVA and Tukey-corrected tests: *^/^**^/^***p < 0.05/0.01/0.001 versus respective wt/wt males and females; ^#/##/###^*p* < 0.05/0.01/0.001 versus respective wt/wt and ko/ko males

To further understand how PAPP-A2 deficiency affects hypothalamic regulatory systems, we analyzed IGF1 signaling pathways in this brain area (Fig. 4A-I). No interaction or effects were found on the phosphorylated form (Tyr607) of PI3K (Fig. 4A). A significant interaction between sex and genotype was found in the protein levels of AKT when it was phosphorylated (p) on serine 473 (F_1,20_ = 22.25, *p* < 0.001), mTOR phosphorylated on serine 2448 (F_1,20_ = 57.51, *p* < 0.001), GSK3β phosphorylated on serine 9 (F_1,20_ = 4.57; *p* < 0.05), ERK1 phosphorylated on threonine 202 (F_1,20_ = 15.54, *p* < 0.001), and ERK2 phosphorylated on tyrosine 204 (F_1,20_ = 4.79, *p* < 0.05), suggesting that the hypothalamic levels of p(Ser473)-AKT, p(Ser2448)-mTOR, p(Ser9)-GSK3β, p(Thr202)-ERK1 and p(Tyr204)-ERK2 were differentially altered in male and female mice with *Pappa2* deletion (Fig. 4B-F). We also found genotype effects on the hypothalamic levels of p(Ser473)-AKT (F_1,20_ = 7.024; *p* < 0.05) and p(Thr202)-ERK1 (F_1,20_ = 5.29, *p* < 0.05). Effects of sex were found on the protein levels of p(Ser473)-AKT (F_1,20_ = 5.22, *p* < 0.05), p(Ser9)-GSK3β (F_1,20_ = 7.47, *p* < 0.05) and p(Thr202)-ERK1 (F_1,20_ = 9.029, *p* < 0.01). No interaction or effects were found on the protein levels of p(Thr172)-AMPKα or p(Ser641)-GS (Fig. 4G, H).

Multiple comparison analysis indicated that protein levels of p(Ser473)-AKT, p(Ser2448)-mTOR and p(Thr202)-ERK1 were higher in the hypothalamus of ko/ko females compared to wt/wt females (**^/^***p < 0.01/0.001) and ko/ko males (^##/###^*p* < 0.01/0.001; Fig. 4B, C, E). However, ko/ko males had lower p(Ser2448)-mTOR than wt/wt males (**p < 0.01; Fig. 4C). Regarding sex differences, ko/ko female had higher levels of p(Ser9)-GSK3β and p(Tyr204)-ERK2 than ko/ko males (^#/##^*p* < 0.05/0.01; Fig. 4D, F), whereas wt/wt females had lower p(Ser2448)-mTOR wt/wt males (^##^*p* < 0.01; Fig. 4C). Representative immunoblots are shown in Fig. 4I (see also the Supplementary Figure S1 for unedited blots).

These results indicate a sex-specific effect of *Pappa2* deletion on the main intracellular regulators of IGF1 signaling pathways in the hypothalamus, with higher protein levels of p(Ser473)-AKT, p(Ser2448)-mTOR and p(Thr202)-ERK1 in ko/ko females.

### Effects of *Pappa2* deletion on IGF1 signaling pathways in the pituitary gland

We also analyzed IGF1 signaling pathways in the pituitary gland (Fig. 4J-N). An interaction between sex and genotype (F_1,20_ = 5.9; *p* < 0.05), and effects of sex (F_1,20_ = 11.08, *p* < 0.01) and genotype (F_1,20_ = 7.42, *p* < 0.05) were only found in the protein levels of p(Ser9)-GSK3β. Multiple comparison analysis indicated that protein levels of p(Ser9)-GSK3β were higher in ko/ko males compared to wt/wt males and ko/ko females (**^/##^*p* < 0.01; Fig. 4J). No interaction or effects were found on the protein levels of p(Thr172)-AMPKα, p(Thr202)-ERK1 or p(Tyr204)-ERK2 (Fig. 4K-M). Representative immunoblots are shown in Fig. 4N (see also the Supplementary Figure S2 for unedited blots).

Thus, there is a sex-specific effect of *Pappa2* deletion on p(Ser9)-GSK3β in the pituitary gland, with higher protein levels in ko/ko males.

### Effects of *Pappa2* deletion on IGF1 signaling pathways in the liver

The IGF1 signaling pathways were also analyzed in the liver of *Pappa2*^ko/ko^ mice (Fig. 4O-W). An interaction between sex and genotype was observed when protein levels of p(Tyr607)-PI3K were analyzed (F_1,20_ = 6.55, *p* < 0.05), suggesting that the protein levels of p(Tyr607)-PI3K were differentially altered in the liver of male and female mice with *Pappa2* deletion (Fig. 4O). Effects of genotype were found on the protein levels of p(Tyr607)-PI3K (F_1,20_ = 10.93, *p* < 0.01) and p(Thr172)-AMPKα (F_1,20_ = 6.264, *p* < 0.05), with higher protein levels in the liver of ko/ko females compared to wt/wt females (*/**p < 0.05/0.01; Fig. 4O, U). Effects of sex were found on the protein levels of p(Ser2448)-mTOR (F_1,20_ = 4.19, *p* = 0.05) and p(Thr172)-AMPKα (F_1,20_ = 8.185, *p* < 0.05), with females having lower protein levels than males (^#^*p* < 0.05; Fig. 4Q, U). No interaction or effects on protein levels of p(Ser473)-AKT, p(Ser9)-GSK3β, p(Thr202)-ERK1, p(Tyr204)-ERK2 or p(Ser641)-GS were found (Fig. 4P, R, S, T, V). Representative immunoblots are shown in Fig. 4W (see also the Supplementary Figure S3 for unedited blots).

These results indicate a sex-specific effect of *Pappa2* deletion on p(Tyr607)-PI3K in the liver, with higher protein levels in ko/ko females. *Pappa2* deficiency was also accompanied by increased protein levels of p(Thr172)-AMPKα in ko/ko females.

### Effects of rhGH, rhIGF1 and rhPAPP-A2 on body and bone length

**Fig. 5:**
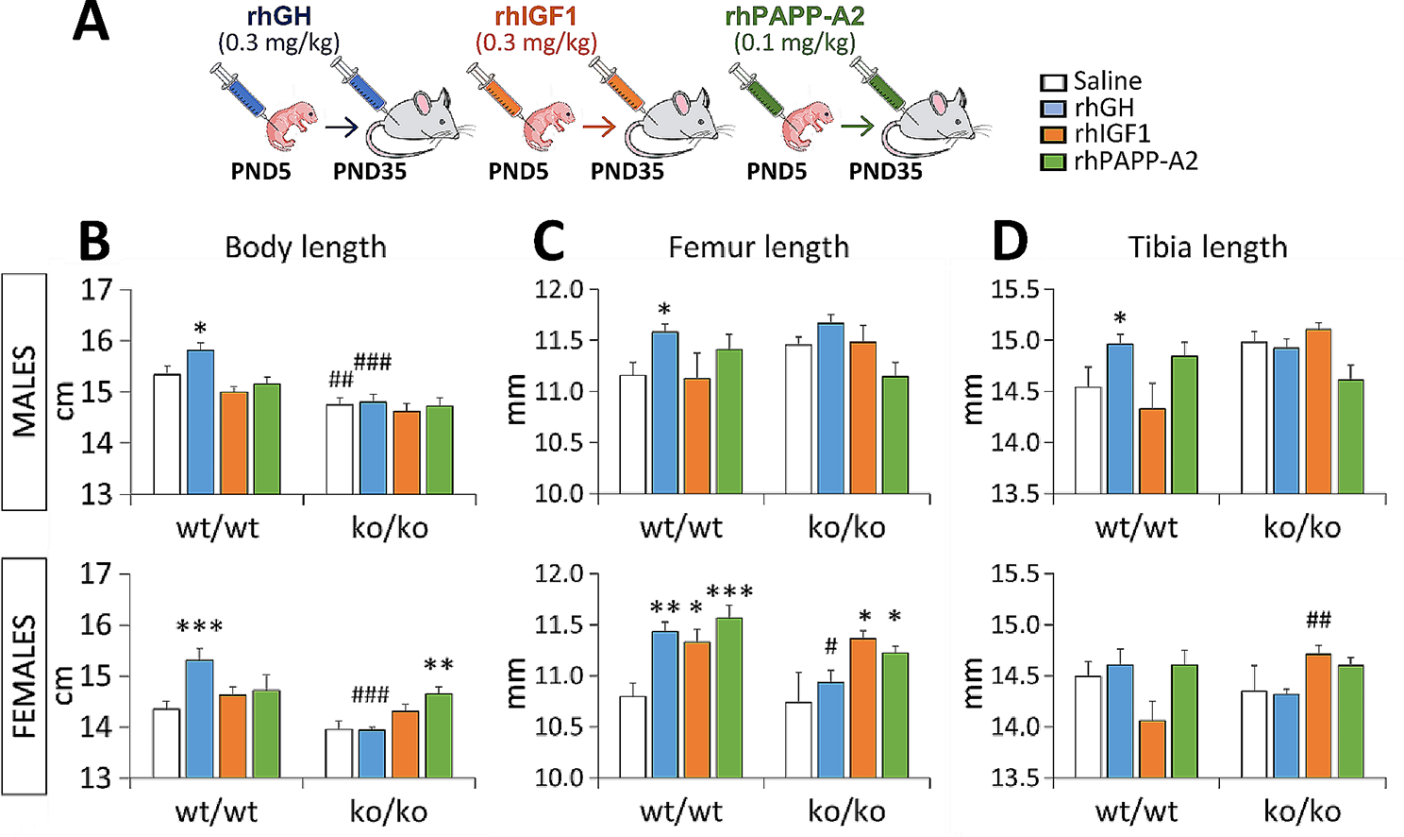
Experimental design (**A**) and auxological parameters of body (**B**), femur (**C**) and tibia (**D**) length in *Pappa2*^ko/ko^ and *Pappa2*^wt/wt^ mice (males and females) treated with rhGH, rhIGF1 and rhPAPP-A2 for 30 days, from PND5 to PND35. Data are represented as mean ± S.E.M (*N* = 8–10 per experimental group). Two-way ANOVA and Tukey-corrected tests: */**/***p < 0.05/0.01/0.001 versus respective saline-treated males and females; ^#/##/###^*p* < 0.05/0.01/0.001 versus respective wt/wt males and females

Given that treatment with recombinant human IGF1 (rhIGF1) in patients with PAPP-A2 mutations improved growth velocity and height following recombinant human (rh)IGF1 treatment [5], here we first analyzed body and bone (femur and tibia) length in *Pappa2*^wt/wt^ and *Pappa2*^ko/ko^ male and female mice treated with rhGH, rhIGF1 and rhPAPP-A2 for 30 postnatal days (PND) from PND5 to PND35 (Fig. 5A). A significant interaction between treatment and genotype was found in body length, but not bone length, of mice treated with rhGH (F_1,64_ = 9.94, *p* < 0.01), suggesting that the effect of rhGH treatment on body length is genotype-dependent. However, interactions between treatment and sex were observed in body and femur lengths of mice treated with rhIGF1 and rhPAPP-A2 (body length: F_1,64_ = 6.69, *p* = 0.01, F_1,64_ = 6.03, *p* = 0.01, respectively; femur length: F_1,64_ = 5.72, *p* < 0.02, F_1,64_ = 9.28, *p* < 0.005, respectively), suggesting that the effect of rhIGF1 and rhPAPP-A2 treatments on body and femur length is sex-dependent.

ffect of rhGH treatment was only observed on body length (F_1,64_ = 11.32, *p* < 0.002), whereas effects of rhGH (F_1,64_ = 13.45, *p* < 0.001), rhIGF1 (F_1,64_ = 5.64, *p* = 0.02) and rhPAPP-A2 (F_1,64_ = 7.64, *p* < 0.01) were found on femur length. Analyzing the sexes separately, the rhPAPP-A2 effect on body length was specifically significant in females (F_1,33_ = 13.1, *p* < 0.001). Genotype and sex effects on body length were observed for all three treatments (genotype: F_1,64_ > 8.5, *p* < 0.005; sex: F_1,64_ > 20.03, *p* < 0.001), as well as the sex effects on bone length (F_1,64_ > 4.28, *p* < 0.05).

Multiple comparisons showed that rhGH treatment increased body and bone length in *Pappa2*^wt/wt^ mice of both sexes, but not *Pappa2*^ko/ko^ mice, compared to respective untreated mice (*/**/***p < 0.05/0.01/0.001; Fig. 5B-D). Moreover, body length of rhGH-treated *Pappa2*^ko/ko^ mice of both sexes and femur length of rhGH-treated *Pappa2*^ko/ko^ females were shorter than respective rhGH-treated *Pappa2*^wt/wt^ mice (^#/###^*p* < 0.05/0.001; Fig. 5B, C). rhIGF1 and rhPAPP-A2 treatments specifically increased femur length in *Pappa2*^wt/wt^ and *Pappa2*^ko/ko^ females, but not males, compared to respective untreated mice (*/***p < 0.05/0.001; Fig. 5C), whereas rhPAPP-A2 also increased body length in *Pappa2*^ko/ko^ females only, compared to untreated *Pappa2*^ko/ko^ females (**p < 0.01; Fig. 5B).

Thus, these results indicate a female-specific effect of rhPAPP-A2 treatment on body and femur length.

### Effects of rhGH, rhIGF1 and rhPAPP-A2 on circulating levels of IGF1 and IGFBP5

**Fig. 6:**
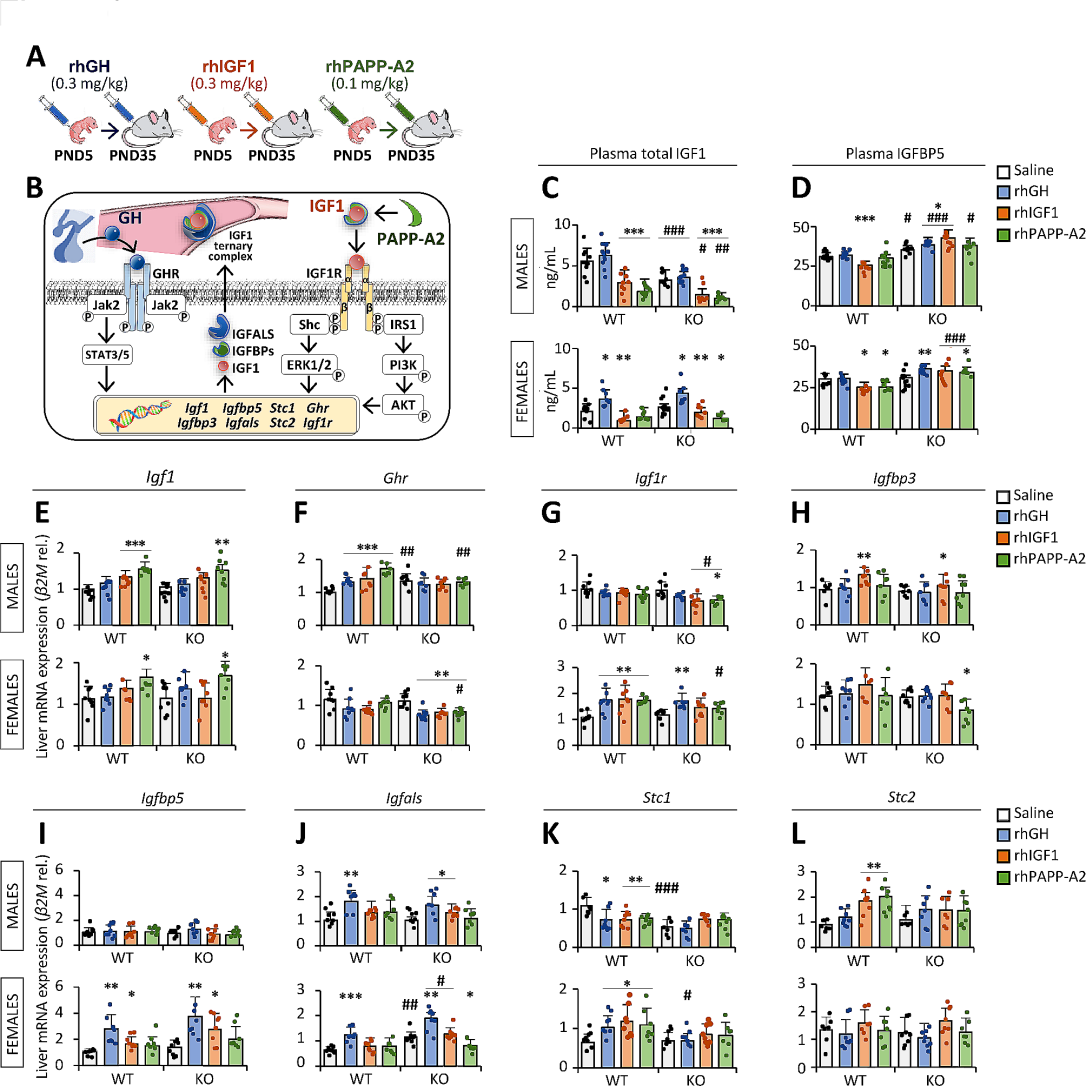
Experimental design (**A**) and schematic diagram (**B**) of the analysis of circulating hormone concentrations, and liver IGF1 system gene expression in *Pappa2*^ko/ko^ and *Pappa2*^wt/wt^ mice (males and females) treated with rhGH, rhIGF1 and rhPAPP-A2 for 30 days, from PND5 to PND35. Concentrations of total IGF1 (**C**) and IGFBP5 (**D**) in plasma, and relative *Igf1* (**E**), *Ghr* (**F**), *Igf1r* (**G**), *Igfbp3* (**H**), *Igfbp5* (**I**), *Igfals* (**J**), *Stc1* (**K**) and *Stc1* (**L**) mRNA levels are represented as mean ± S.E.M (*n* = 8–10 per experimental group). See Table S1 for additional information. Two-way ANOVA and Tukey-corrected tests: */**/***p < 0.05/0.01/0.001 versus respective saline-treated males and females; ^#/##/###^*p* < 0.05/0.01/0.001 versus respective wt/wt males and females

Given that treatment with recombinant human IGF1 (rhIGF1) in patients with PAPP-A2 mutations increased bioactive IGF1 but serum levels of total IGF1 and ternary complexes remained elevated [5], we analyzed circulating concentrations of total IGF1 and IGFBP5 in *Pappa2*^wt/wt^ and *Pappa2*^ko/ko^ male and female mice treated with rhGH, rhIGF1 and rhPAPP-A2 for 30 postnatal days (PND) from PND5 to PND35 (Fig. 6A, B). Complete statistical analysis of the data is shown in the Supplementary Table S3. Interaction between treatment, genotype and sex was only observed in plasma levels of total IGF1 in mice treated with rhPAPP-A2 (F_1,65_ = 5.47, *p* < 0.05), suggesting that the effect of rhPAPP-A2 treatment on plasma total IGF1 levels is sex- and genotype-dependent. However, interactions between treatment and genotype were found in plasma levels of IGFBP5, but not IGF1, in mice treated with rhGH (F_1,68_ = 13.3, *p* = 0.01), rhIGF1 (F_1,63_ = 33.6, *p* < 0.001) and rhPAPP-A2 (F_1,64_ = 5.15, *p* < 0.05), suggesting a similar genotype-dependent effect of all three treatments on plasma IGFBP5 levels. Analyzing the sexes separately, this interaction between treatment and genotype in plasma levels of IGFBP5 was significant in females treated with rhGH (F_1,31_ = 10.1, *p* < 0.01) and rhIGF1 (F_1,34_ = 12.8, *p* < 0.01), and in males treated with rhIGF1 (F_1,34_ = 24.7, *p* < 0.001). Also, an interaction between treatment and genotype was specifically observed in plasma levels of total IGF1 of males treated with rhPAPP-A2 (F_1,35_ = 4.9, *p* < 0.05). Interactions between treatment and sex were only observed in plasma levels of total IGF1 after rhIGF1 (F_1,65_ = 6.45, *p* < 0.05) and rhPAPP-A2 (F_1,65_ = 17.8, *p* < 0.001) treatments. Interactions between genotype and sex were found in plasma levels of total IGF1 after rhGH (F_1,67_ = 26.6, *p* < 0.001), rhIGF1 (F_1,65_ = 25.0, *p* < 0.001) and rhPAPP-A2 (F_1,65_ = 15.0, *p* < 0.001) treatments (Supplementary Table S3).

Effects of rhGH treatment were observed on plasma levels of total IGF1 (F_1,67_ = 12.1, *p* = 0.001) and IGFBP5 (F_1,68_ = 14.7, *p* < 0.001), whereas effects of rhIGF1 (F_1,65_ = 39.8, *p* < 0.001) and rhPAPP-A2 (F_1,65_ = 82.4, *p* < 0.001) were only found on plasma levels of total IGF1. Analyzing the sexes separately, the rhGH effects were significant on IGFBP5 in males (F_1,37_ = 6.83, *p* < 0.05), and total IGF1 (F_1,30_ = 13.8, *p* < 0.001) and IGFBP5 (F_1,31_ = 7.52, *p* = 0.01) in females, whereas the rhIGF1 and rhPAPP-A2 effects were only significant on total IGF1 in both males (F_1,34_ = 32.2, *p* < 0.001 and F_1,34_ = 86.2, *p* < 0.001, respectively) and females (F_1,31_ = 9.74, *p* < 0.01 and F_1,34_ = 12.9, *p* = 0.001, respectively). Genotype and sex effects on plasma levels of both total IGF1 and IGFBP5 were observed for all three treatments (F_1,63-68_ > 4.89, *p* < 0.05 and F_1,62-68_ > 24.8, *p* < 0.001, respectively) (Supplementary Table S3).

Multiple comparisons showed that rhGH treatment increased plasma levels of total IGF1 in *Pappa2*^wt/wt^ and *Pappa2*^ko/ko^ females, but not males, compared to respective untreated mice (*p < 0.05; Fig. 6C). In contrast, rhIGF1 treatment decreased plasma levels of total IGF1 in *Pappa2*^wt/wt^ and *Pappa2*^ko/ko^ males and females compared to respective untreated mice (**/***p < 0.01/0.001; Fig. 6C). rhPAPP-A2 treatment also decreased plasma levels of total IGF1 in *Pappa2*^wt/wt^ and *Pappa2*^ko/ko^ males, and *Pappa2*^ko/ko^ females compared to respective untreated mice (*/***p < 0.05/0.001; Fig. 6C). rhGH treatment increased plasma levels of IGFBP5 specifically in *Pappa2*^ko/ko^ males and females compared to respective untreated mice (*/**p < 0.05/0.01; Fig. 6D). rhIGF1 treatment decreased plasma levels of IGFBP5 in *Pappa2*^wt/wt^ males and females, and increased plasma levels of IGFBP5 in *Pappa2*^ko/ko^ males only, compared to respective untreated mice (*/***p < 0.05/0.001; Fig. 6D). Interestingly, rhPAPP-A2 treatment decreased plasma levels of IGFBP5 in *Pappa2*^wt/wt^ females, and increased plasma levels of IGFBP5 in *Pappa2*^ko/ko^ females, but not males, compared to respective untreated females (*p < 0.05; Fig. 6D). Regarding genotype, lower plasma total IGF1 levels were observed in both untreated (saline) and treated *Pappa2*^ko/ko^ males, but not females, compared to respective *Pappa2*^wt/wt^ mice (^#/##/###^*p* < 0.05/0.01/0.001; Fig. 6C). In contrast, higher plasma IGFBP5 levels were observed in both untreated and treated *Pappa2*^ko/ko^ males, and in rhIGF1- and rmPAPPA2-treated *Pappa2*^ko/ko^ females compared to respective *Pappa2*^wt/wt^ mice (^#/###^*p* < 0.05/0.001; Fig. 6D).

In summary, there is an effect of rhIGF1 and rhPAPP-A2 treatments on circulating total IGF1, resulting in lower plasma levels of total IGF1 in males and females.

### Effects of rhGH, rhIGF1 and rhPAPP-A2 on liver gene expression of IGF1 system components

Since changes in IGF1 bioactivity can be modulated by liver production of IGF1 ternary complexes in response to treatment, we also analyzed liver gene expression of IGF1 system components in *Pappa2*^wt/wt^ and *Pappa2*^ko/ko^ male and female mice treated with rhGH, rhIGF1 and rhPAPP-A2 for 30 postnatal days (PND) from PND5 to PND35 (Fig. 6A, B). Complete statistical analysis of the data is shown in the Supplementary Tables S4 and S5. Interactions between treatment, genotype and sex were only observed in *Stc1* mRNA expression levels after rhGH treatment (F_1,60_ = 6.39, *p* < 0.05), and in *Ghr* and *Stc1* mRNA expression levels after treatment with rhIGF1 (F_1,63_ = 6.5, *p* < 0.05 and F_1,58_ = 9.7, *p* < 0.01, respectively) and rhPAPP-A2 (F_1,59_ = 11.9, *p* = 0.001 and F_1,57_ = 7.75, *p* < 0.01, respectively), suggesting that the effect of rhIGF1 and rhPAPP-A2 treatments on liver gene expression of *Ghr* and *Stc1* are sex- and genotype-dependent. In addition, interactions between treatment and genotype were observed in *Ghr* mRNA expression levels in mice treated with all three treatments (rhGH: F_1,62_ = 7.12, *p* = 0.01; rhIGF1: F_1,63_ = 6.98, *p* = 0.01; rhPAPP-A2: F_1,59_ = 20.6, *p* < 0.001), suggesting a similar genotype-dependent effect of all three treatments on *Ghr* gene expression. Analyzing the sexes separately, this interaction between treatment and genotype in *Ghr* mRNA expression levels was observed in males, but not females, treated with all three treatments (rhGH: F_1,30_ = 12.1, *p* < 0.01; rhIGF1: F_1,31_ = 10.7, *p* < 0.01; rhPAPP-A2: F_1,29_ = 27.2, *p* < 0.001), whereas interaction between factors in *Stc1* mRNA expression levels was only found in males treated with rhIGF1 and rhPAPP-A2 (F_1,27_ = 14.5, *p* = 0.001 and F_1,28_ = 16.4, *p* < 0.001, respectively). Interestingly, interactions between treatment and genotype were also found in *Igf1r* and *Igfals* mRNA expression levels after rhPAPP-A2 treatment only, being specifically significant in females (F_1,29_ = 5.53, *p* < 0.05 and F_1,29_ = 7.7, *p* = 0.01, respectively). Interactions between treatment and sex were observed in *Ghr*, *Igf1r* and *Stc1* mRNA expression levels after all three treatments (rhGH: F_1,60-62_ > 7.94, *p* < 0.01; rhIGF1: F_1,58-63_ > 9.22, *p* < 0.005; rhPAPP-A2: F_1,57-59_ > 6.61, *p* < 0.05). In addition, interactions between treatment and sex were also observed in *Igfbp5* mRNA expression levels after rhGH and rhIGF1 treatments (F_1,60-61_ > 8.7, *p* < 0.005), and in *Stc2* mRNA expression levels after rhGH and rhPAPP-A2 treatments (F_1,57-58_ > 5.29, *p* < 0.05). Interactions between genotype and sex were also found in *Ghr* and *Igfals* mRNA expression levels in mice treated with rhGH (F_1,62_ > 4.53, *p* < 0.05), and in *Igfals* and *Igfbp5* mRNA expression levels in mice treated with rhIGF1 (F_1,61-62_ > 6.76, *p* < 0.05) and rhPAPP-A2 (F_1,58-60_ > 4.65, *p* < 0.05). Further statistical data can be found in the Supplementary Tables S4 and S5.

There were effects of all three treatments on *Igf1* mRNA expression levels (rhGH: F_1,62_ = 4.26, *p* < 0.05; rhIGF1: F_1,63_ = 10.2, *p* < 0.01; rhPAPP-A2: F_1,60_ = 34.7, *p* < 0.001), effects of rhGH and rhIGF1 treatments on *Igfals* and *Igfbp5* mRNA expression levels (rhGH: F_1,60-62_ > 24.6, *p* < 0.001; rhIGF1: F_1,61-63_ > 8.52, *p* < 0.005), and effects of rhIGF1 and rhPAPP-A2 treatments on *Stc2* mRNA expression levels (rhIGF1: F_1,57_ = 15.4, *p* < 0.001; rhPAPP-A2: F_1,57_ = 6.68, *p* < 0.05). Also, an effect of rhGH treatment on *Igf1r* mRNA expression levels (F_1,62_ = 5.46, *p* < 0.05) and an effect of rhIGF1 treatment on *Igfbp3* mRNA expression levels (F_1,59_ = 6.66, *p* < 0.05) were specifically observed. Analyzing the sexes separately, the rhGH treatment effects on *Ghr* and *Igfbp5* mRNA expression levels were specifically observed in females (F_1,31_ > 14.0, *p* < 0.001), whereas those on *Stc1* and *Stc2* mRNA expression levels were specifically observed in males (F_1,27_ > 5.57, *p* < 0.05). The rhIGF1 treatment effects on *Igf1*, *Igfals*, *Igfbp3* and *Stc2* mRNA expression levels were specifically observed in males (F_1,27-31_ > 5.18, *p* < 0.05), whereas those on *Igf1r*, *Igfbp5* and *Stc1* mRNA expression levels were specifically observed in females (F_1,31_ > 8.18, *p* < 0.008). The rhPAPP-A2 treatment effect on *Stc2* mRNA expression levels was specifically observed in males (F_1,28_ = 13.0, *p* = 0.001), whereas that on *Stc1* mRNA expression levels was specifically observed in females (F_1,29_ = 5.45, *p* < 0.05). Genotype effects were observed on *Stc1* mRNA expression levels for all three treatments (F_1,57-60_ > 10.3, *p* < 0.002), *Igfals* mRNA expression levels for rhGH and rhIGF1 treatments (F_1,62-63_ > 4.51, *p* < 0.05), and *Igfbp3* mRNA expression levels for rhIGF1 treatment (F_1,59_ > 5.24, *p* < 0.05). Finally, sex effects on *Ghr*, *Igf1r*, *Igfbp3* and *Igfbp5* mRNA expression levels were observed for all three treatments (rhGH: F_1,60-62_ > 18.9, *p* < 0.001; rhIGF1: F_1,59-62_ > 10.5, *p* < 0.002 rhPAPP-A2: F_1,58-59_ > 6.05, *p* < 0.05), whereas sex effects on *Igfals* mRNA expression levels were specifically found for rhIGF1 (F_1,63_ = 13.7, *p* < 0.001) and rhPAPP-A2 (F_1,60_ = 13.8, *p* < 0.001) treatments. See the Supplementary Tables S4 and S5 for further statistical data.

Multiple comparisons showed that rhIGF1 treatment increased *Igf1* mRNA expression levels only in *Pappa2*^wt/wt^ males compared to respective untreated males (***p < 0.001; Fig. 6E). In addition, rhPAPP-A2 treatment increased *Igf1* mRNA expression levels in *Pappa2*^wt/wt^ and *Pappa2*^ko/ko^ males and females compared to respective untreated mice (*/**/***p < 0.05/0.01/0.001; Fig. 6E). All three treatments increased *Ghr* mRNA expression levels in *Pappa2*^wt/wt^ males, but not *Pappa2*^ko/ko^ males, and decreased *Ghr* mRNA expression levels in *Pappa2*^ko/ko^ females, but not *Pappa2*^wt/wt^ females, compared to respective untreated mice (**/***p < 0.01/0.001; Fig. 6F). All three treatments also increased *Igf1r* mRNA expression levels in *Pappa2*^wt/wt^ females (**p < 0.01; Fig. 6G). In addition, rhGH treatment, but not rhIGF1 and rhPAPP-A2, specifically increased *Igf1r* mRNA expression levels in *Pappa2*^ko/ko^ females compared to untreated females (**p < 0.01; Fig. 6G). In contrast, rhPAPP-A2 treatment, but not rhGH and rhIGF1, specifically decreased *Igf1r* mRNA expression levels in *Pappa2*^ko/ko^ males (*p < 0.05; Fig. 6G). rhIGF1 treatment increased *Igfbp3* mRNA expression levels in *Pappa2*^wt/wt^ and *Pappa2*^ko/ko^ males, whereas rhPAPP-A2 treatment decreased *Igfbp3* mRNA expression levels in *Pappa2*^ko/ko^ females only (*/**p < 0.05/0.01; Fig. 6H). rhGH and rhIGF1 treatments increased *Igfbp5* mRNA expression levels in *Pappa2*^wt/wt^ and *Pappa2*^ko/ko^ females, but not males, compared to respective untreated mice (*/**p < 0.05/0.01; Fig. 6I). rhPAPP-A2 treatment showed no significant changes in *Igfbp5* mRNA expression levels (Fig. 6I). rhGH treatment increased *Igfals* mRNA expression levels in *Pappa2*^wt/wt^ and *Pappa2*^ko/ko^ males and females compared to respective untreated mice (*/**/***p < 0.05/0.01/0.001; Fig. 6J). rhIGF1 treatment also increased *Igfals* mRNA expression levels in *Pappa2*^ko/ko^ males only, whereas rhPAPP-A2 treatment decreased *Igfals* mRNA expression levels in *Pappa2*^ko/ko^ females only (*p < 0.05; Fig. 6J). Interestingly, *Stc1* mRNA expression levels were decreased in *Pappa2*^wt/wt^ males and increased in *Pappa2*^wt/wt^ females after each of the three treatments (*/**p < 0.05/0.01; Fig. 6K). No changes in *Stc1* mRNA expression levels were observed in treated *Pappa2*^ko/ko^ mice. Finally, rhIGF1 and rhPAPP-A2 treatments increased Stc2 mRNA expression levels in *Pappa2*^wt/wt^ males only (**p < 0.01; Fig. 6L). Regarding genotype, *Ghr* mRNA expression levels were higher in untreated (saline) *Pappa2*^ko/ko^ males, but not females, compared to respective untreated *Pappa2*^wt/wt^ mice (^##^*p* < 0.01; Fig. 6F). In contrast, *Ghr* mRNA expression levels were specifically lower in rhPAPPA2-treated *Pappa2*^ko/ko^ males and females compared to respective rmPAPPA2-treated *Pappa2*^wt/wt^ mice (^#/##^*p* < 0.05/0.01; Fig. 6F). rhIGF1- and rhPAPPA2-treated *Pappa2*^ko/ko^ males, and rhPAPPA2-treated *Pappa2*^ko/ko^ females showed lower *Igf1r* mRNA expression levels compared to respective rhPAPPA2-treated *Pappa2*^wt/wt^ mice (^#^*p* < 0.05; Fig. 6G). *Igfals* mRNA expression levels were specifically higher in untreated *Pappa2*^ko/ko^ females, and rhGH- and rhIGF1-treated *Pappa2*^ko/ko^ females, compared to respective *Pappa2*^wt/wt^ females (^#/##^*p* < 0.05/0.01; Fig. 6J). *Stc1* mRNA expression levels were specifically lower in untreated *Pappa2*^ko/ko^ males, compared to respective untreated *Pappa2*^wt/wt^ males (^###^*p* < 0.001; Fig. 6K).

Summarizing, we found a female-specific effect of treatment on liver mRNA levels of components of IGF ternary complexes. Specifically, in females, rhPAPP-A2 increased *Igf1* and *Igf1r*, and decreased *Igfbp3* and *Igfals*, suggesting an upregulation of IGF1 availability in females.

### Effects of rhGH, rhIGF1 and rhPAPP-A2 on liver protein expression of IGF-1 intracellular pathway regulators

**Fig. 7:**
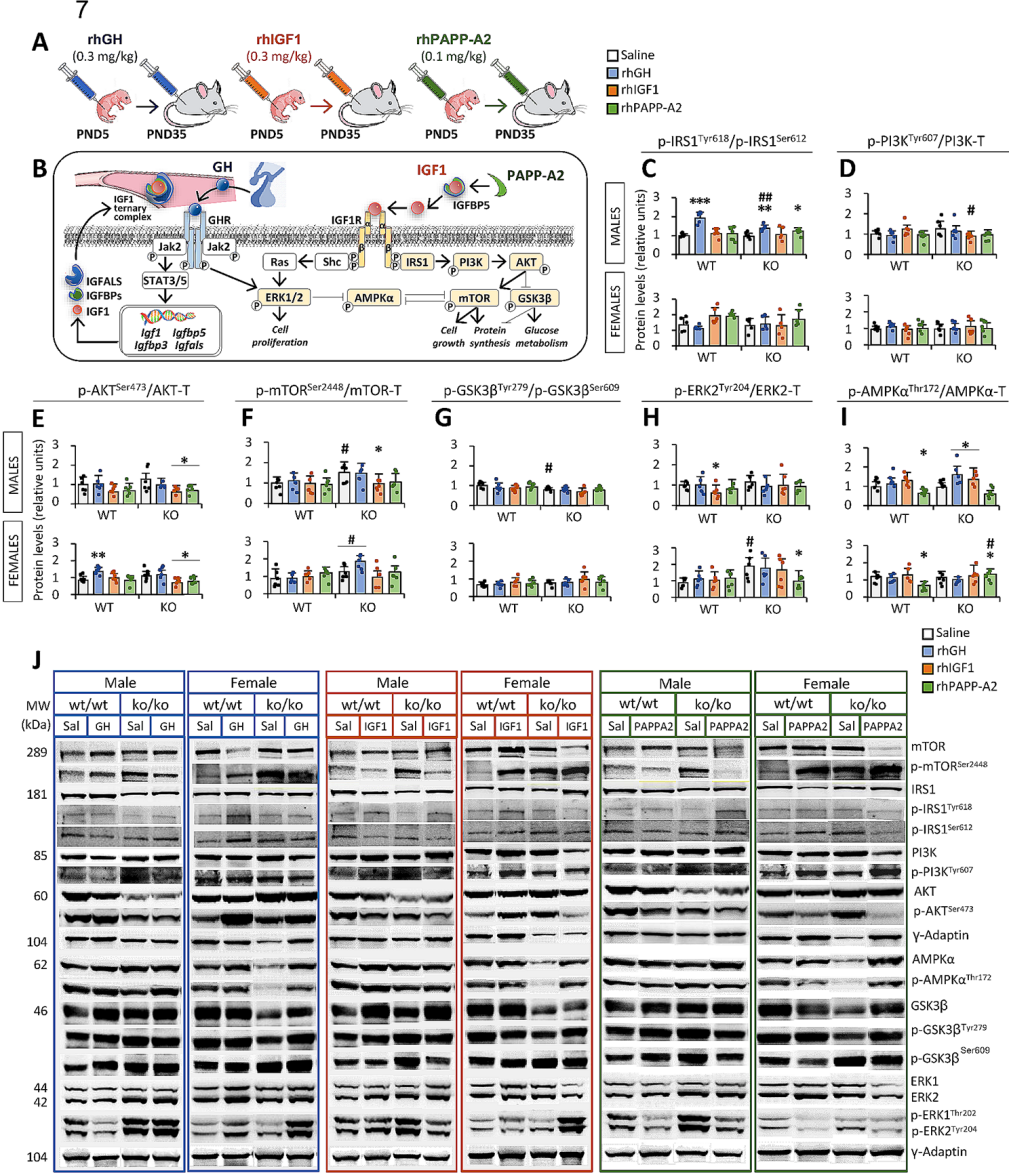
Experimental design (**A**) and schematic diagram (**B**) of the analysis of key regulators of IGF1 signaling pathways in the liver of *Pappa2*^ko/ko^ and *Pappa2*^wt/wt^ mice (males and females) treated with rhGH, rhIGF1 and rhPAPP-A2 for 30 days, from PND5 to PND35. Phosphorylated forms of IRS1 (**C**), PI3K (**D**), AKT (**E**), mTOR (**F**), GSK3β (**G**), ERK2 (**H**) and AMPKα (**I**) are represented as mean ± S.E.M (*n* = 6 per experimental group). Representative immunoblots (**J**) are also shown. See Table S2 and Figures S4 and S6 for additional information. Two-way ANOVA and Tukey-corrected tests: */**/***p < 0.05/0.01/0.001 versus respective saline-treated males and females; ^#/##^*p* < 0.05/0.01 versus respective wt/wt males and females

To understand whether treatment effects on liver gene expression of IGF1 system components are differentially regulated by specific intracellular signaling pathways in response to *Pappa2* deficiency, protein and phosphoprotein expression levels of key pathway regulators of main cell functions, including cell proliferation, cell growth, lipid metabolism, glucose metabolism and protein synthesis, were analyzed in the liver of *Pappa2*^wt/wt^ and *Pappa2*^ko/ko^ male and female mice treated with rhGH, rhIGF1 and rhPAPP-A2 for 30 postnatal days (PND) from PND5 to PND35 (Fig. 7A, B). See the Supplementary Figure S6 for total protein expression levels and the Supplementary Tables S6 and S7 for complete statistical analysis. Interactions between treatment, genotype and sex were specifically observed in phosphorylated IRS1 ratio [p(Tyr618/Ser612)IRS1] in mice treated with rhGH (F_1,40_ = 4.42, *p* < 0.05), and phosphorylated PI3K [p(Tyr607)PI3K] levels in mice treated with rhIGF1 (F_1,47_ = 4.80, *p* < 0.05), suggesting that the effects of rhGH and rhIGF1 treatments on p(Tyr618/Ser612)IRS1 and p(Tyr607)PI3K levels, respectively, are sex- and genotype-dependent. However, interaction between treatment and genotype was specifically observed in phosphorylated ERK2 [p(Tyr204)ERK2] levels in mice treated with rhPAPP-A2 only (F_1,46_ = 9.24, *p* < 0.005), suggesting a genotype-dependent effect of rhPAPP-A2 treatment on p(Tyr204)ERK2 levels.

Analyzing the sexes separately, interactions between treatment and genotype were specifically found in: (1) p(Tyr618/Ser612)IRS1 ratio in males treated with rhGH (F_1,23_ = 12.3, *p* < 0.01), (2) p(Tyr607)PI3K levels in males treated with rhIGF1 (F_1,23_ = 6.88, *p* < 0.05), (3) p(Ser473)AKT levels in females treated with rhIGF1 (F_1,23_ = 5.70, *p* < 0.05), and (4) p(Tyr204)ERK2 and p(Thr172)AMPKα levels in females treated with rhPAPP-A2 (F_1,23_ > 5.42, *p* < 0.05). Interactions between treatment and sex were observed in p(Thr172)AMPKα levels in mice treated with rhGH (F_1,46_ = 6.38, *p* < 0.05) and rhPAPP-A2 (F_1,45_ = 16.5, *p* < 0.001). In addition, interactions between treatment and sex were also found in p(Tyr618/Ser612)IRS1 ratio in mice treated with rhGH (F_1,40_ = 13.5, *p* < 0.001), and in p(Tyr279/Ser609)GSK3β ratio in mice treated with rhIGF1 (F_1,47_ = 5.9, *p* < 0.05). Interactions between genotype and sex were observed in p(Tyr279/Ser609)GSK3β ratio after all three treatments (rhGH: F_1,46_ = 6.97, *p* < 0.05; rhIGF1: F_1,47_ = 7.03, *p* < 0.05; rhPAPP-A2: F_1,46_ = 9.24, *p* = 0.01). In addition, interactions between genotype and sex were found in p(Tyr618/Ser612)IRS1 ratio and p(Tyr204)ERK2 levels in mice treated with rhGH only (F_1,47_ > 4.41, *p* < 0.05). See the Supplementary Tables S6 and S7 for further statistical analysis.

Effects of rhGH and rhPAPP-A2 treatments on the p(Tyr618/Ser612)IRS1 ratio were observed, whereas an effect of rhPAPP-A2 treatment on p(Thr172)AMPKα levels were only found. Analyzing the sexes separately, effects of all three treatments on p(Thr172)AMPKα levels were specifically observed in males, but not females (rhGH: F_1,23_ = 6.06, *p* < 0.05; rhIGF1: F_1,23_ = 7.44, *p* < 0.05; rhPAPP-A2: F_1,23_ = 18.89, *p* < 0.001). In addition, rhGH and rhPAPP-A2 effects on the p(Tyr618/Ser612)IRS1 ratio (F_1,23_ = 87.8, *p* < 0.001 and F_1,23_ = 7.7, *p* < 0.05, respectively), and rhIGF1 and rhPAPP-A2 effects on p(Ser473)AKT levels were observed in males (F_1,23_ = 9.98, *p* < 0.01 and F_1,23_ = 7.65, *p* < 0.05, respectively). Only females showed rhPAPP-A2 effects on the p(Tyr618/Ser612)IRS1 ratio and p(Ser473)AKT levels (F_1,23_ > 4.85, *p* < 0.05). Genotype effects on p(Tyr204)ERK2 levels were observed after rhGH and rhIGF1 treatment (F_1,47_ > 8.62, *p* < 0.005), whereas a genotype effect on p(Ser2448)mTOR levels was specifically found after rhGH treatment (F_1,45_ = 19.9, *p* < 0.001). Analyzing the sexes separately, genotype effects on the p(Tyr279/Ser609)GSK3β ratio after all three treatments (rhGH: F_1,23_ = 4.74, *p* < 0.05; rhIGF1: F_1,23_ = 9.89, *p* < 0.01; rhPAPP-A2: F_1,23_ = 11.1, *p* < 0.01), and on the p(Tyr618/Ser612)IRS1 ratio and p(Ser2448)mTOR levels after rhGH treatment (F_1,23_ = 16.1, *p* < 0.001 and F_1,23_ = 7.25, *p* < 0.05, respectively) were only observed in males. Genotype effects on p(Tyr204)ERK2 levels after rhGH and rhIGF1 treatments (F_1,23_ = 9.82, *p* < 0.005 and F_1,23_ = 7.69, *p* < 0.05, respectively), and on p(Ser2448)mTOR levels after rhGH treatment (F_1,23_ = 13.9, *p* = 0.001) were only found in females. Finally, sex effects on p(Tyr618/Ser612)IRS1 ratio were observed after rhIGF1 and rhPAPP-A2 treatments (F_1,40_ = 7.37, *p* = 0.01 and F_1,23_ = 12.4, *p* = 0.001, respectively), whereas sex effects were also found on the p(Tyr279/Ser609)GSK3β ratio after rhGH treatment, and p(Thr172)AMPKα levels after rhPAPP-A2 treatment (F_1,46_ = 5.45, *p* < 0.05 and F_1,45_ = 10.4, *p* < 0.01, respectively). See the Supplementary Tables S6 and S7 for further statistical analysis.

Multiple comparisons showed that rhGH treatment increased the p(Tyr618/Ser612)IRS1 ratio only in *Pappa2*^wt/wt^ and *Pappa2*^ko/ko^ males, but not females, compared to respective untreated mice (**/***p < 0.01/0.001; Fig. 7C). No significant differences in the p(Tyr618/Ser612)IRS1 ratio were observed after rhIGF1 treatment (Fig. 7C). However, rhPAPP-A2 treatment specifically increased the p(Tyr618/Ser612)IRS1 ratio in *Pappa2*^ko/ko^ males compared to respective untreated males (*p < 0.05; Fig. 7C). No significant differences in p(Tyr607)PI3K levels were found after any of the treatments (Fig. 7D). rhIGF1 and rhPAPP-A2 treatments, but not that of rhGH, specifically decreased p(Ser473)AKT levels in *Pappa2*^ko/ko^ males and females compared to respective untreated mice (*p < 0.05; Fig. 7E). rhGH treatment increased p(Ser473)AKT levels in *Pappa2*^wt/wt^ females only (**p < 0.01; Fig. 7E). In addition, only rhIGF1 treatment decreased p(Ser2448)mTOR levels in *Pappa2*^ko/ko^ males compared to respective untreated males (*p < 0.05; Fig. 7F). No significant differences in the p(Tyr279/Ser609)GSK3β ratio were found after any of the treatments (Fig. 7G). p(Tyr204)ERK2 levels were specifically decreased in *Pappa2*^wt/wt^ males treated with rhIGF1, and in *Pappa2*^ko/ko^ females treated with rhPAPP-A2, compared to respective untreated mice (*p < 0.05; Fig. 7H). p(Thr172)AMPKα levels were specifically increased in *Pappa2*^ko/ko^ males, but not females, treated with rhGH and rhIGF1, and in *Pappa2*^ko/ko^ females, but not males, treated with rhPAPP-A2 (*p < 0.05; Fig. 7I). In contrast, *Pappa2*^wt/wt^ male and female mice treated with rhPAPP-A2 showed decreased p(Thr172)AMPKα levels (*p < 0.05; Fig. 7I). Regarding genotype, the p(Tyr618/Ser612)IRS1 ratio was specifically lower in rhGH-treated *Pappa2*^ko/ko^ males, but not females, compared to respective treated *Pappa2*^wt/wt^ mice (^##^*p* < 0.01; Fig. 7C). p(Tyr607)PI3K levels were specifically lower in rhIGF1-treated *Pappa2*^ko/ko^ males, but not females, compared to respective treated *Pappa2*^wt/wt^ mice (^#^*p* < 0.05; Fig. 7D). Untreated (saline) *Pappa2*^ko/ko^ male and female mice showed higher p(Ser2448)mTOR levels compared to respective untreated *Pappa2*^wt/wt^ mice (^#^*p* < 0.05; Fig. 7F). In addition, untreated *Pappa2*^ko/ko^ males showed a lower p(Tyr279/Ser609)GSK3β ratio, whereas untreated *Pappa2*^ko/ko^ females showed higher p(Tyr204)ERK2 levels compared to respective untreated *Pappa2*^wt/wt^ mice (^#^*p* < 0.05; Fig. 7G, H). Finally, p(Thr172)AMPKα levels were specifically higher in PAPPA2-treated *Pappa2*^ko/ko^ females compared to PAPPA2-treated *Pappa2*^wt/wt^ females (^#^*p* < 0.05; Fig. 7I). Representative immunoblots are shown in Fig. 7J (see also the Supplementary Figure S4 for unedited blots).

In summary, we observed a sex-specific effect of treatment on liver protein levels of key regulators of IGF1 signaling pathways. Specifically, rhPAPP-A2 decreased phosphorylated forms of AKT and ERK2, and increased phosphorylated forms of AMPK in the liver of *Pappa2*^ko/ko^ females, suggesting downregulation of cell growth and protein synthesis and tight regulation of cell proliferation and lipid metabolism.

### Effects of rhGH, rhIGF1 and rhPAPP-A2 on liver protein expression of GH intracellular pathway regulators

**Fig. 8:**
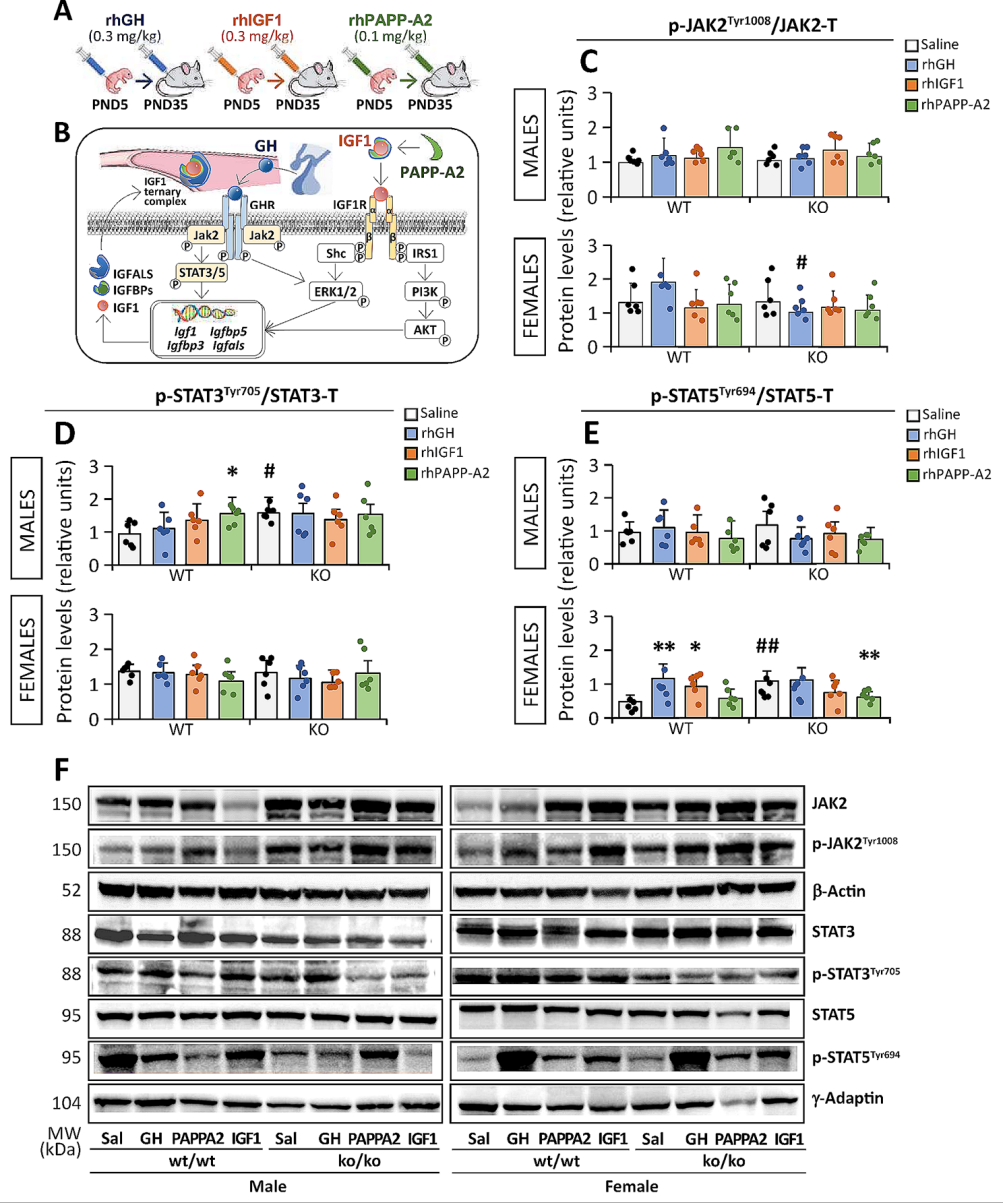
Experimental design (**A**) and schematic diagram (**B**) of the analysis of key regulators of GH signaling pathways in the liver of *Pappa2*^ko/ko^ and *Pappa2*^wt/wt^ mice (males and females) treated with rhGH, rhIGF1 and rhPAPP-A2 for 30 days, from PND5 to PND35. Phosphorylated forms of JAK2 (**C**), STAT3 (**D**) and STAT5 (**E**) are represented as mean ± S.E.M (*n* = 6 per experimental group). Representative immunoblots (**F**) are also shown. See Table S2 and Figures S4 and S7 for additional information. Two-way ANOVA and Tukey-corrected tests: */**p < 0.05/0.01 versus respective saline-treated males and females; ^#/##^*p* < 0.05/0.01 versus respective wt/wt males and females

Protein and phosphoprotein expression levels of canonical intracellular signaling of GH were also analyzed in the liver of *Pappa2*^wt/wt^ and *Pappa2*^ko/ko^ male and female mice treated with rhGH, rhIGF1 and rhPAPP-A2 for 30 postnatal days (PND) from PND5 to PND35 (Fig. 8A, B). See the Supplementary Figure S7 for total protein expression levels and the Supplementary Tables S8 and S9 for complete statistical analysis. Interaction between treatment and genotype was specifically observed in phosphorylated STAT5 [p(Tyr694)STAT5] levels in mice treated with rhIGF-1 (F_1,47_ = 4.65, *p* < 0.05) and rhPAPP-A2 (F_1,47_ = 4.23, *p* < 0.05), suggesting a genotype-dependent effect of rhIGF-1 and rhPAPP-A2 treatments on p(Tyr694)STAT5 levels. Interaction between genotype and sex was observed in p(Tyr705)STAT3 levels after GH treatment only (rhGH: F_1,47_ = 5.56, *p* < 0.05). Analyzing the sexes separately, interaction between treatment and genotype in p(Tyr694)STAT5 levels was specifically observed in females, but not males, treated with all three treatments (rhGH: F_1,31_ = 11.2, *p* < 0.01; rhIGF1: F_1,22_ = 10.0, *p* < 0.01; rhPAPP-A2: F_1,22_ = 8.64, *p* < 0.01).

An effect of rhPAPP-A2 treatment was only found on the p(Tyr694)STAT5 levels was only found (F_1,47_ = 6.83, *p* < 0.05). In addition, a sex effect on p(Tyr1008)JAK2 levels was specifically observed in a rhGH-dependent manner (F_1,47_ = 4.42, *p* < 0.05), suggesting increased levels in females. Analyzing the sexes separately, effects of rhGH and rhPAPP-A2 treatments on p(Tyr694)STAT5 levels were observed in females, but not males (rhGH: F_1,31_ = 12.8, *p* = 0.001; rhPAPP-A2: F_1,23_ = 4.63, *p* < 0.05). Genotype effects on p(Tyr694)STAT5 levels after rhGH and rhPAPP-A2 treatments (F_1,31_ = 8.24, *p* < 0.01 and F_1,22_ = 10.5, *p* < 0.01, respectively) were only found in females, whereas a genotype effect on p(Tyr705)STAT3 levels after rhGH treatment (F_1,30_ = 10.1, *p* < 0.01) was only observed in males. See the Supplementary Tables S8 and S9 for further statistical analysis.

Multiple comparisons showed that *Pappa2* deletion decreased p(Tyr1008)JAK2 levels only in rhGH-treated females, but not males, compared to respective untreated mice (^#^*p* < 0.05; Fig. 8C). In contrast, *Pappa2* deletion increased p(Tyr705)STAT3 levels in all treated males, but not females (^#^*p* < 0.05; Fig. 8D), and increased p(Tyr694)STAT5 levels in all treated females, but not males (^##^*p* < 0.01; Fig. 8E), compared to respective untreated mice. Regarding treatment, rhGH specifically increased p(Tyr705)STAT3 levels in *Pappa2*^wt/wt^ males only (*p < 0.05; Fig. 8D). In addition, rhGH and rhIGF1 treatments increased p(Tyr694)STAT5 levels in *Pappa2*^wt/wt^ females only (*/**p < 0.05/0.01; Fig. 8E). Finally, rhPAPP-A2 treatment specifically decreased p(Tyr694)STAT5 levels in *Pappa2*^ko/ko^ females only (**p < 0.01; Fig. 8E). Representative immunoblots are shown in Fig. 8F (see also the Supplementary Figure S4 for unedited blots).

In summary, we observed sex-specific effects of genotype and treatment on liver protein levels of key regulators of GH signaling pathways. Specifically, *Pappa2* deletion increased a phosphorylated form of STAT3 in the liver of males, but not females, and increased a phosphorylated form of STAT5 in the liver of females, but not males. Only rhPAPP-A2 treatment decreased STAT5 phosphorylation in the liver of *Pappa2*^ko/ko^ females, suggesting that treatment with rhPAPP-A2 suppresses STAT5 activation probably by continuous GH stimulation specifically in females.

## Discussion

The present study aims to evaluate whether *Pappa2* deficiency and pharmacological manipulation of the GH/IGF1 system are associated with sex-specific differences in growth-related signaling pathways. We describe the expression of the main components of IGF1 ternary complexes and IGF1 intracellular signaling pathways in the hypothalamus, pituitary gland and liver, key tissues involved in the physiological control of growth. Moreover, special emphasis was placed on the differential responses of males and females to this genetic alteration and on the response to pharmacological administration of rhGH, rhIGF1 and rhPAPP-A2. It is known that PAPP-A2 is a key regulator of IGF1 bioavailability and that IGF1, acting as an autocrine, endocrine and paracrine factor, is responsible for promoting efficient growth, development and maintenance at all physiological stages. As growth and the GH/IGF system are sexually dimorphic in rodents [30] and human in an age-specific manner [12], analyzing sex-specific IGF1 system alterations due to the lack of PAPP-A2 proteolytic activity and the response to rhGH, rhIGF1 and rhPAPP-A2 treatments can provide relevant information about the physiopathological mechanisms and signaling pathways underlying the differential implications of PAPP-A2 in growth physiology between males and females. In addition, results may also support the use of promising drugs to alleviate postnatal growth retardation underlying low IGF1 bioavailability observed in PAPP-A2 deficient patients.

Homozygous *Pappa2*^ko/ko^ mice recapitulate several features of the phenotype described in patients with *PAPP-A2* mutations [1, 3, 4, 11], especially regarding auxology (short stature, thin long bones, decreased bone mineral density), hormone levels (elevated serum concentrations of IGF1, IGFBP3, IGFBP5 and IGFALS) and metabolic status (disturbance in carbohydrate metabolism). *Pappa2* deficient mice have growth retardation from the neonatal period to adulthood, with *Pappa2*^ko/ko^ mice having reduced body and bone (femur and tibia) length throughout lifespan [20, 21, 23], which was also found here. In addition, the IGF1 system was described to be altered at the local level in bone of these mice, which probably contributes to not only reduced bone growth but also to the alterations in bone structure (crystallographic parameters), composition (mineral-to-organic matrix ratio, carbonate substitution, collagen maturity) and remodeling (osteopontin, osteocalcin, alpha-1 type 1 collagen) in a sex-dependent manner [16, 23].

Here we demonstrate sex-specific reductions in body length at puberty and adulthood, as well as femur length (raw and relative to body length) in adulthood, and female-specific increases in body and femur length at puberty in mice treated with rhPAPP-A2 for 30 days (PND5-35). Females have been previously described to be smaller and have a lighter skeleton than males [21, 23, 31]. Moreover, we previously described sex-specific differences in bone structure and composition of *Pappa2* deletion mice [23]. The present study indicates that loss of *Pappa2* differentially affects body and femur length in adulthood, but not at puberty onset, in males and females, and thus also altering the normal sex differences in these parameters. At puberty onset, *Pappa2*^ko/ko^ males and females are significantly shorter than the respective *Pappa2*^wt/wt^ males and females, with females being shorter than males in both genotypes. In adulthood, *Pappa2*^ko/ko^ females, but not *Pappa2*^ko/ko^ males, are significantly shorter than their respective *Pappa2*^wt/wt^ controls. On the contrary, in adulthood the femur of *Pappa2*^ko/ko^ males, but not of females, is significantly shorter than that of their respective *Pappa2*^wt/wt^ controls. Compared to rhGH and rhIGF1, rhPAPP-A2 administration specifically increased body and femur length in *Pappa2*^ko/ko^ females at puberty onset. As these sex differences in response to *Pappa2* loss and rhPAPP2 treatment emerged at puberty, our results suggest that the action of PAPP-A2 on growth interacts with pubertal sex steroids through changes at the biochemical and molecular levels in the GH-IGF1 axis.

Mice lacking *Pappa2* also altered plasma concentrations of the main components of IGF1 ternary/binary complexes (e.g., IGF1, IGFBP5), similar to PAPP-A2 deficient patients [4], with a difference between the sexes shown in mice. The absence of *Pappa2* caused an increase in plasma levels of total IGF1, especially in female mice. *Pappa2*^ko/ko^ females also had significant increases in plasma levels of IGFBP5, whose binding to IGF1 is directly controlled by the proteolytic activity of PAPP-A2 [7, 8]. Total IGF1 levels were found to have a similar plasma profile, suggesting that part of IGF1 is bound in ternary and/or binary complexes in conjunction with IGFBP5. Therefore, one would expect reduced IGF1 actions through IGF1R in target tissues. Contrary to rhGH effects, rhIGF1 and rhPAPP-A2 administrations decreased plasma levels of total IGF1 and IGFBP5. These results contrast to the elevated levels of total IGF1 maintained in PAPP-A2 deficient patients treated with rhIGF1 [4]. Plasma levels of bioactive IGF1 exert negative feedback actions on the production and secretion of GH by somatotropic cells of the anterior pituitary gland. In both *Pappa2*^ko/ko^ males and females GH concentrations were increased in the pituitary gland, an effect that can be caused by low IGF1 availability and could result in more liver production of IGF1 and IGFBP5 [32], as can be partially observed after rhGH administration. In our animal model, at least in the liver of *Pappa2*^ko/ko^ females, increased GH secretion is supported by higher mRNA levels of GH targets, such as *Ghr*, *Igf1*, *Igfbp3*, *Igfals* and *Stc1*, similar to rhGH administration that was associated with higher mRNA levels of *Igf1*, *Igf1r*, *Igfbp5*, *Igfals* and *Stc1*, suggesting upregulation of IGF ternary complexes.

Although low IGF1 bioavailability would increase GH synthesis, as observed here, this increase is not reflected in the plasma of animals lacking *Pappa2*, as previously reported in patients with PAPP-A2 deficiency [1]. However, as GH is secreted in a pulsatile fashion, a single plasma measurement does not necessarily reflect its total integrated levels, nor the efficiency of the pulsatile pattern to promote growth. Several studies reported that there is not always a perfect correlation between IGF1 and GH actions, suggesting that IGF1 production depends on additional factors that modulate this axis [33–35]. In agreement, mice with liver IGF1 deficiency (LID) have reduced circulating total IGF1 levels and a consequent increase in plasma GH levels, whereas mice with *Igfals* gene deletion (ALSko) have reduced levels of circulating total IGF1 with normal GH levels [33]. Only double-knockout mice (LID + ALSko) were described to have a marked increase in free IGF1 levels [33].

**Fig. 9:**
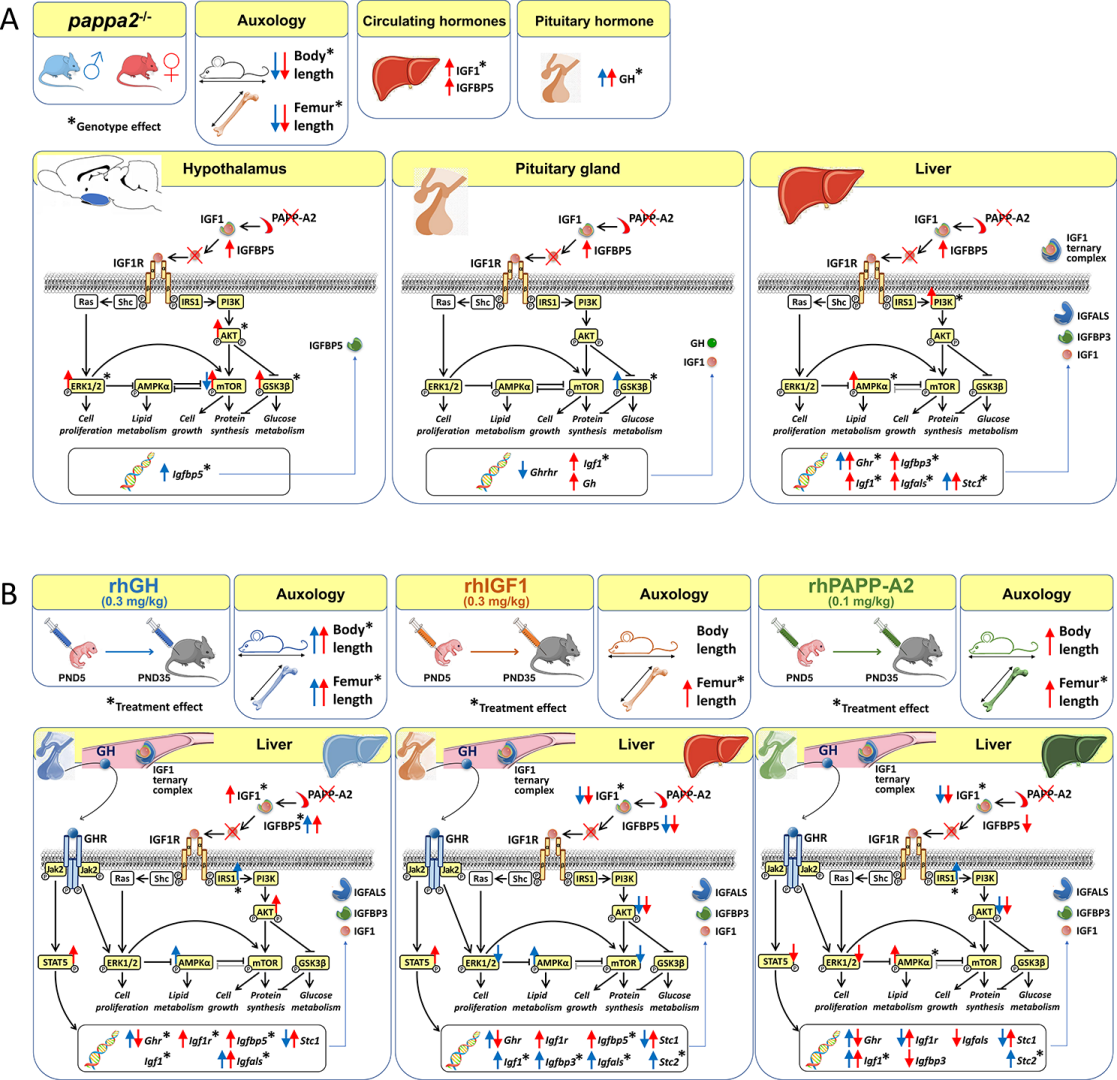
(**A**) Schematic diagrams summarizing the alterations in the hypothalamus-pituitary-liver axis that result in decreased body and femur length in *Pappa2*^ko/ko^ mice (males and females) in adulthood. Increased and decreased levels are represented by a blue arrow for males and a red arrow for females. The effect of genotype is indicated by an asterisk (*). Reduced body and femur length of *Pappa2*^ko/ko^ mice and female-specific increased expressions of components of IGF1 binary/ternary complexes (IGF1 and IGFBP5 in plasma, *Igfbp5* in hypothalamus, and *Igfbp3* and *Igfals* in liver) could be consistent with reduced IGF1 availability and signaling in target tissues. Sex-specific modulation of key sensors of IGF1 signaling pathways (phosphorylation of AKT, mTOR, GSK3β and ERK1/2 in the female hypothalamus, phosphorylation of GSK3β and ERK1/2 in the male pituitary gland, and phosphorylation of PI3K and AMPKα in the female liver) may be relevant to explain growth impairment through a dysregulated IGF1 system due to PAPP-A2 deficiency. (**B**) Schematic diagrams summarizing changes in IGF1 ternary complex components and IGF1 signaling pathways that result in increased body and/or femur length after the pharmacological administration of rhGH, rhIGF1 and rhPAPP-A2 for 30 days (PND5-35) in *Pappa2*^ko/ko^ mice (males and females) at puberty onset. Increased and decreased levels are represented by a blue arrow for males and a red arrow for females. The effect of treatment is indicated by an asterisk (*). Female-specific increases in body and femur length and female-specific decreases in expressions of components of IGF1 ternary complexes (IGF1 and IGFBP5 in plasma, and *Igfbp3* and *Igfals* in liver) could be consistent with increased IGF1 availability and signaling in target tissues. Female-specific modulation of key sensors of liver IGF1 signaling pathways (reduced phosphorylation of AKT and ERK2, and increased phosphorylation of AMPKα) may be relevant to explain growth improvement through a regulated IGF1 system by rhPAPP-A2 treatment

The sex differences associated with the effects of *Pappa2* deletion and low IGF1 bioavailability are supported by strong evidence for cross-regulation between IGF1 and estrogen receptors (ERα and GPER) in growth, sexual maturation and neuroendocrine function [36–40]. In particular, the non-classical estrogen receptor GPER (GPR30) has been described to be relevant in bone growth in a sex-dependent manner [41, 42]. Here, we analyzed sex differences in the gene expression of the main components of the IGF1 system in the hypothalamus, pituitary gland and liver of male and female *Pappa2*^ko/ko^ mice. As expected, *Pappa2* mRNA levels were dramatically reduced in the hypothalamus and pituitary gland of *Pappa2*^ko/ko^ male and female mice, and no *Pappa2* expression was detected in their liver. Significantly attenuated proteolytic activity of PAPP-A2 in the hypothalamus and pituitary gland (and probably in the liver) would most likely reduce local IGF1 bioavailability and increase circulating IGF1 half-life, due to increased expression of components of IGF ternary/binary complexes. Following a comprehensive analysis of the mRNA levels of IGF1 system components in the hypothalamus-pituitary-liver axis, animals lacking *Pappa2* presented changes in the gene expression of *Igf1*, *Igfbp3*, *Igfbp5*, *Igfals*, *Stc1*, *Ghr*, with some changes being sex-dependent (*Pappa2*, *Gh*, *Ghrhr*, *Igf1*, *Igfbp3*). In fact, there are marked sex differences in the mRNA levels of IGF1 system components (*Pappa2*, *Ghrh*, *Ghrhr*, *Gh*, *Ghr*, *Igf1*, *Igf1r*, *Igfbp5*, *Igfals*) in different tissues in this animal model (Fig. 9A). In *Pappa2*^ko/ko^ mice increased mRNA levels of *Igfbp5* were found in the hypothalamus, *Igf1* in the pituitary gland, and *Igf1*, *Igfbp3*, *Igfals* and *Stc1* in the liver. These results, together with increased circulating concentrations of IGF1 and IGFBP5, agree with an upregulated expression of key components of the IGF1 ternary/binary complexes that probably result in lower local levels of bioactive IGF1 in the hypothalamus, pituitary gland and liver. Although animals lacking *Pappa2* show a dysregulated IGF1 system, the gene expression of hypothalamic hormones involved in pituitary GH production, such as *Ghrh* and *Ghih*, is not particularly affected by genotype. These results suggest that the increased GH levels in the pituitary gland could be a direct consequence of decreased negative feedback of IGF1 at this level. It should also be noted that previous studies suggest a central and peripheral role of IGFBP5 in energy metabolism [43]. *Igfbp5* expression was specifically increased in the hypothalamus of male mice fed a high-fat diet without affecting other members of the IGF system [44], and IGFBP5 was altered in the hypothalamus of *Pappa2*^ko/ko^ mice of both sexes.

The increased *Ghr* mRNA levels in the liver of *Pappa2*^ko/ko^ male and female mice (genotype effect) substantiate an increase in GH-stimulated IGF1 expression in the liver [32]. Sex-specific effects of *Pappa2* deficiency on the gene expression of IGF1 system components are represented by: (1) a specific increase of *Igfbp5* in the hypothalamus of *Pappa2*^ko/ko^ males; (2) specific increases of *Gh* and *Igf1* in the pituitary gland of *Pappa2*^ko/ko^ females; (3) a decrease of *Ghrhr* in the pituitary gland of *Pappa2*^ko/ko^ males; and (4) increases of *Igf1*, *Igfbp3* and *Igfals* in the liver of *Pappa2*^ko/ko^ females. The female-specific increase in the expression of components of the IGF1 system in liver suggests that the increased synthesis of IGF1, most likely stimulated by GHR activation in the liver (increased expression of pituitary GH and liver *Ghr*) is mainly sequestered in IGF ternary/binary complexes of *Pappa2*^ko/ko^ females, also supported by the increased levels of circulating levels of IGFBP5. However, there was no change in the expression of the hypothalamic factors and the intracellular signaling pathways analyzed were either unaffected or increased in *Pappa2*^ko/ko^ females, indicating that the lack of *Pappa2* may have limited effects on the IGF system at the level of the hypothalamus. It is plausible to assume that reduced IGF1 bioavailability and alteration of IGF1 intracellular signaling pathways were tissue and sex-specific.

There are similar sex-specific effects of rhGH, rhIGF1 and rhPAPP-A2 treatments on liver *Ghr* mRNA levels, resulting in increased levels in males and decreased levels in females. Compared to rhGH and rhIGF1, where an overall increased expression of the liver IGF1 system components were detected, sex-specific effects of rhPAPP-A2 on the liver gene expression of IGF1 system components are represented by: (1) increases of *Igf1* and *Stc2*, and decreases of *Igfr1* and *Stc1* in liver of males; (2) increases of *Igf1*, *Igf1r* and *Stc1*, and decreases of *Igfbp3* and *Igfals* in liver of females (Fig. 9B). These results support rhPAPP-A2-induced reduction in IGF ternary/binary complexes and suggest higher IGF1 bioavailability involving subsequent changes of IGF1 intracellular signaling pathways.

Longitudinal growth and somatic maturation are mainly regulated by IGF1 binding to IGF1R and the subsequent IGF1R activation of main intracellular signaling pathways in target tissues [32, 45–47]. Intracellular signal of the IGF1R is reported to be regulated by cross-interaction with ER and differential activation of synergistic PI3K/AKT, GSK3, ERK and Wnt pathways, with these actions possibly being tissue/cell specific [37, 48–51]. To determine sex differences in putative IGF1 signaling pathway dysregulation in response to *Pappa2* deficiency, we analyzed key intracellular pathways, including PI3K, AKT, AMPKα, ERK1/2, mTOR and GSK3β, among others, in the hypothalamus-pituitary-liver axis of *Pappa2*^ko/ko^ male and female mice, and the liver of mice treated with rhGH, rhIGF1 and rhPAPP-A2. Sex-specific effects of *Pappa2* deficiency on these signaling pathways included: (1) a decrease of p(Ser2448)-mTOR in the hypothalamus of *Pappa2*^ko/ko^ males, and increases of p(Ser2448)-mTOR, p(Thr202/Tyr204)-ERK1/2, p(Ser473)-AKT and p(Ser9)-GSK3β in the hypothalamus of *Pappa2*^ko/ko^ females; (2) an increase of p(Ser9)-GSK3β (a tendency of p(Thr202/Tyr204)-ERK1/2) in the pituitary gland of *Pappa2*^ko/ko^ males; and (3) increases of p(Tyr607)-PI3K and p(Thr172)-AMPKα in the liver of *Pappa2*^ko/ko^ females (Fig. 9A). Sex-specific effects of treatment on these signaling pathways in liver included: (1) increases of p(Tyr618/Ser612)IRS1 in rhGH and rhPAPPA2-treated males; (2) decreases of p(Ser473)-AKT in rhIGF1 and rhPAPPA2-treated mice, and increases in rhGH-treated females; (3) increases of p(Thr172)-AMPKα in the liver of rhGH and rhIGF1-treated males, and rhPAPPA2-treated females; and (4) decreases of p(Ser2448)-mTOR and p(Tyr204)-ERK2 in rhIGF1-treated males (Fig. 9B).

There are key signaling pathways via RAS/MEK/ERK and PI3K/AKT/mTOR that are activated following cellular activation of IGF1R, and mainly serve the function of regulating unrestricted cell proliferation and decreased sensitivity to apoptotic mechanisms that involve changes in glucose and lipid metabolism and protein synthesis in target tissues responsible for promoting growth, development, and maintenance at all physiological stages [52–54]. AKT, a principal target of PI3K signaling for IGF1, regulates glucose metabolism via GSK3 phosphorylation/inhibition and mTOR activation [52, 54, 55]. Increased levels of AKT phosphorylation and the p110α/PI3K subunit were also described to be related to the astrocytic regulation of IGF1 in neuroinflammation in a sex-dependent manner [51]. In our study, increased phosphorylated levels of AKT, GSK3β and mTOR were specifically found in the hypothalamus of *Pappa2*^ko/ko^ females, and this may suggest a close regulatory control of glucose metabolism and protein synthesis in the female hypothalamus. Effects of local activation of the PI3K/AKT signaling pathway, via subsequent mTOR activation, are promoted by the suppression of GSK3β (Ser9)-phosphorylation/inactivation, resulting in a key role in protein synthesis to coordinate neural cell proliferation, neuronal differentiation and polarization and axonal growth [56, 57]. Our results indicated increased expression of GSK3β (Ser9)-phosphorylation in the hypothalamus of *Pappa2*^ko/ko^ female mice, suggesting possible affectation of neuronal proliferation/maturation. Our results also suggest that higher ERK1/2 phosphorylation (also known as p44 and p42 MAPK) is probably inducing cell proliferation in the hypothalamus of *Pappa2*^ko/ko^ females (Fig. 9A). Together these results could indicate increased cell turnover, or even increased metabolism; hypotheses that should be analyzed in future studies.

It is also important to focus on the hypothalamic AMPK signaling pathway via mTOR, a primary oxygen and nutrient sensing pathway that plays a key role in the central modulation of glucose and amino acid utilization and signaling through IGF1, an effect that could explain differences in growth and development in a sex-specific manner [58, 59]. Indeed, AMPK-mTOR signaling is also necessary for bone development through promotion of chondrocyte proliferation and hypertrophy [60]. Our results suggest that sex-specific effects of *Pappa2* deficiency on the hypothalamic expression of phosphorylated mTOR, that is decreased in *Pappa2*^ko/ko^ males and increased in *Pappa2*^ko/ko^ females (Fig. 9A), could result in changes in the hypothalamic control of growth physiology and maintenance in a sex-dependent manner.

In pituitary somatotropic cells, ERK signaling becomes activated by GHRH and inhibited by somatostatin, resulting in increased proliferative and non-proliferative responses, respectively [61]. In addition, activation of ERK signaling is required for GH production, while inhibition of PKA/CREB signaling pathway via GSK3β dephosphorylation/activation is necessary to suppress GH synthesis in somatotrophs [61–63]. Despite no evidence of an AMPK role in hypothalamic control of somatotropic function, AMPK activation was followed by a reduction of GH storage and release in the pituitary gland, an effect that may be inhibited by ERK1/2 phosphorylation and enhanced by the negative feedback regulatory action of IGF1 on the pituitary somatotropic cells [18]. These studies support the fact that the genotype effect due to increased levels of phosphorylated GSK3β (and a tendency in phosphorylated ERK1/2) agree with increased GH secretion in the pituitary gland at least in *Pappa2*^ko/ko^ males, as described above, whereas normal GSK3 expression in the pituitary gland of *Pappa2*^ko/ko^ females may result in a limited effect on GH production (Fig. 9A).

Accumulated evidence indicates that PI3K/AKT pathway activation and transcription factor-induced synthesis and release of IGF1 in the liver are required for normal longitudinal growth before and during puberty, body composition (increasing body mass and decreasing body fat) and protein, lipid, carbohydrate and mineral metabolism [17]. IGF1 elicits transient insulin-like activation of the PI3K p85 subunit, leading to AKT phosphorylation and GSK3 inactivation, and thereby facilitating glycogen synthesis, fatty acid synthesis for storage, cell apoptosis protection and cell cycle progression [64]. In addition, AMPK activity has the potential to interfere with GH-induced IGF1-mediated cellular anabolic events (glycogen and protein synthesis) by modulating PI3K/AKT pathways and inhibiting the mTOR response in the liver [18]. A controversial study associated IGFBP5 overexpression, that may result in low IGF1 bioavailability, with increased glucose uptake and glycogen synthesis through activating both the IRS1/AKT and AMPK pathways [65]. Our results suggest that increased phosphorylated levels of PI3K and AMPK, specifically observed in the liver of *Pappa2*^ko/ko^ female mice, may explain higher production of IGF1 and IGFBP5, and key components of IGF1 ternary/binary complexes (*Igf1*, *Igfbp3* and *Igfals*), in addition to blocking anabolic events leading to a negative energy balance [4]. Our results also indicate that these changes observed specifically in *Pappa2*^ko/ko^ females can be counteracted after rhPAPP-A2 treatment, downregulating IGF1 ternary/binary complexes through decreased phosphorylated AKT and ERK2 levels and increased phosphorylated AMPK levels.

IGF1 secretion in the liver is induced by GH activation of JAK2-STAT5b intracellular signaling (Choi and Waxman, 2000). Activation of this pathway is required for the sex-dependent effects of GH on liver gene expression, including components of IGF1 ternary/binary complexes, and whole-body growth rates. Our results indicated a female-specific alteration of phosphorylated JAK2 and STAT5 in the liver, an effect that was genotype- and treatment-dependent. Notably, the increased STAT5 phosphorylation observed specifically in *Pappa2*^ko/ko^ females were normalized after rhPAPP-A2 treatment only, supporting downregulation of IGF1 ternary/binary complexes through decreased phosphorylated STAT5.

### Perspective and sifnificance

This study presents a systematic description of the sex-specific changes found in body and femur length, IGF1 system and IGF1 signaling pathways through the hypothalamus-pituitary-liver axis in a *Pappa2*-deficient animal model with growth retardation, and in the liver of these mice after pharmacological manipulation of the GH/IGF1 system by rhGH, rhIGF1 and rhPAPP-A2 administrations for 30 days. Reduction in body and bone length of *Pappa2*^ko/ko^ mice and higher expression of specific components of IGF1 binary/ternary complexes in a female-specific manner (higher levels of IGF1 and IGFBP5 in plasma, *Igfbp5* in hypothalamus, and *Igfbp3* and *Igfals* in liver) could be consistent with reduced IGF1 availability and signaling in target tissues. Indeed, sex-specific alterations of IGF1 signaling pathways (AKT, mTOR, GSK3β and ERK1/2 phosphorylation in the female hypothalamus, GSK3β and ERK1/2 phosphorylation in the male pituitary gland, and PI3K and AMPKα phosphorylation in the female liver) and GH signaling pathway (STAT5 phosphorylation in the female liver) could be relevant to explain growth impairment through a dysregulated IGF1 system due to PAPP-A2 deficiency. Compared to rhGH and rhIGF1, rhPAPP-A2 treatment was associated with increased body and femur length, and lower expression of specific components of IGF1 binary/ternary complexes in a female-specific manner (lower levels of IGF1 and IGFBP5 in plasma, and *Igfbp3* and *Igfals* in liver, and higher levels of *Igf1* and *Igf1r* in liver). Moreover, female-specific changes of IGF1 and GH signaling pathways (AKT, AMPKα and STAT5 phosphorylation in the female liver) could be relevant to explain growth improvement through increased IGF1 bioavailability.

In conclusion, these results shed light on the involvement of PAPP-A2 in sex-based growth physiology, contribute to elucidate IGF1 signaling disruption in a female-specific manner, and support the use of promising drugs to alleviate progressive postnatal growth retardation underlying low IGF1 bioavailability in PAPP-A2 deficient patients. Further studies are necessary to determine how pubertal sex steroids and estrogen receptors, which interact with the GH-IGF system to regulate sex-specific growth promotion, are altered in the absence of *Pappa2*.

### Electronic supplementary material

Below is the link to the electronic supplementary material.

Supplementary Material 1

Supplementary Material 2

Supplementary Material 3

Supplementary Material 4

Supplementary Material 5

Supplementary Material 6

Supplementary Material 7

Supplementary Material 8

Supplementary Material 9

Supplementary Material 10

Supplementary Material 11

Supplementary Material 12

Supplementary Material 13

Supplementary Material 14

Supplementary Material 15

Supplementary Material 16

Supplementary Material 17

## Data Availability

The datasets used and/or analyzed during the current study are available from the corresponding author upon reasonable request.
