# Supplementary material for: Sex-based differences in growth-related IGF1 signaling in response to PAPP-A2 deficiency: comparative effects of rhGH, rhIGF1 and rhPAPP-A2 treatments
Source: Biol Sex Differ. 2024 Apr 8;15:34. doi: 10.1186/s13293-024-00603-5 (PMC11000399; doi:10.1186/s13293-024-00603-5)
Supplement: Supplementary file 2 — Supplementary Material 2 [file 13293_2024_603_MOESM2_ESM.docx]

**Supplementary Table S1.** Primers used for the TaqMan® Gene Expression Assays (ThermoFisher).

| **Gene ID** | **GenBank accession numbers** | **Products size (bp)** |
| --- | --- | --- |
| *Actb (β-actin)* | Mm02619580_g1 | 143 |
| *β2M* | Mm00560865_m1 | 58 |
| *Pappa2* | Mm01284029_m1 | 70 |
| *Ghrh* | Mm00439100_m1 | 65 |
| *Ghih-sst* | Mm00436671_m1 | 86 |
| *Ghrhr* | Mm01326479_m1 | 65 |
| *Sstr1* | Mm00436679_s1 | 60 |
| *Gh* | Mm00433590_g1 | 56 |
| *Ghr* | Mm00439093_m1 | 61 |
| *Igf1* | Mm00439560_m1 | 77 |
| *Igf2* | Mm00439564_m1 | 107 |
| *Igf1r* | Mm00802831_m1 | 106 |
| *Igfbp3* | Mm01187817_m1 | 78 |
| *Igfbp5* | Mm00516037_m1 | 70 |
| *Igfals* | Mm07307442_s1 | 63 |
| *Stc1* | Mm01322191_m1 | 63 |
| *Stc2* | Mm00441560_m1 | 60 |

Abbreviations: *Actb*, β-actin; *β2M*, beta-2 microglobulin; *Gh*, growth hormone; *Ghr*, growth hormone receptor; *Ghih-sst*, growth hormone-inhibiting hormone-somatostatin; *Ghrh*, growth hormone-releasing hormone; *Ghrhr*, growth hormone-releasing hormone receptor; *Igf1*, insulin-like growth factor 1; *Igf2*, insulin-like growth factor 2; *Igf1r*, insulin-like growth factor 1 receptor; *Igfals*, insulin-like growth factor binding protein acid labile subunit; *Igfbp3*, insulin-like growth factor-binding protein 3; *Igfbp5*, insulin-like growth factor-binding protein 5; *Sstr 1*, somatostatin receptor; *Stc1*, staniocalcin 1; *Stc2*, staniocalcin 2.
