# Supplementary material for: Sex-based differences in growth-related IGF1 signaling in response to PAPP-A2 deficiency: comparative effects of rhGH, rhIGF1 and rhPAPP-A2 treatments
Source: Biol Sex Differ. 2024 Apr 8;15:34. doi: 10.1186/s13293-024-00603-5 (PMC11000399; doi:10.1186/s13293-024-00603-5)
Supplement: Supplementary file 3 — Supplementary Material 3 [file 13293_2024_603_MOESM3_ESM.docx]

**Supplementary Table S2.** Antibodies used for protein expression by Western blotting.

| **Antigen** | **MW (kDa)** | **Immunogen** | **Manufacturing details** | **Dilution** |
| --- | --- | --- | --- | --- |
| Adaptin-γ | 104 | Mouse adaptin-γ aa. 642-821 | BD Biosciences (#610385). Mouse monoclonal antibody | 1:2000 |
| β-Actin | 52 | Slightly modified β-cytoplasmic actin N-terminal peptide, Ac-Asp-Asp-Asp-Ile-Ala-Ala-Leu-Val-Ile-Asp-Asn-Gly-Ser-Gly-Lys. | Merck (#A5316). Mouse monoclonal antibody | 1:2000 |
| Mammalian target of rapamycin (mTOR) | 289 | Synthetic peptide corresponding to residues surrounding Ser2481 of human mTOR. | Cell Signaling Technology (#2972). Rabbit polyclonal antibody | 1:1000 |
| Phospho-mTOR (Ser2448) | 289 | Synthetic phosphopeptide corresponding to residues surrounding Ser2448 of human mTOR. | Cell Signaling Technology (#2971). Rabbit polyclonal antibody | 1:1000 |
| Phosphoinositide 3-kinase or PI3K p85 [19H8] | 85 | Synthetic peptide corresponding to the sequence of human PI3K p85. | Cell Signaling Technology (#4257). Rabbit monoclonal antibody | 1:1000 |
| Phospho-PI3K-p85 (Tyr607) | 85 | Synthetic peptide corresponding to Human PI 3 Kinase p85 alpha (phospho Y607) conjugated to keyhole limpet haemocyanin. | Abcam (#182651). Rabbit polyclonal antibody | 1:1000 |
| AMP-activated protein kinase or AMPKα [D63G4] | 62 | Synthetic peptide corresponding to residues surrounding Lys40 of human AMPKα. | Cell Signaling Technology (#5832). Rabbit monoclonal antibody | 1:1000 |
| Phospho-AMPKα (Thr172) [40H9] | 62 | Synthetic peptide corresponding to residues surrounding Thr172 of human AMPKα protein. | Cell Signaling Technology (#2535). Rabbit monoclonal antibody | 1:1000 |
| Protein kinase B (PKB) or AKT | 60 | Synthetic peptide corresponding to the carboxy-terminal sequence of mouse AKT. | Cell Signaling Technology (#9272). Rabbit polyclonal antibody | 1:1000 |
| Phospho-AKT (Ser473) | 60 | Synthetic phosphopeptide corresponding to residues surrounding Ser473 of mouse AKT. | Cell Signaling Technology (#9271). Rabbit polyclonal antibody | 1:1000 |
| Glycogen synthase kinase-3 or GSK-3β [D5C5Z] | 46 | Recombinant protein specific to the carboxy terminus of human GSK-3β protein. | Cell Signaling Technology (#12456). Rabbit monoclonal antibody | 1:1000 |
| Phospho-GSK-3β (Ser9) [D85E12] | 46 | Synthetic phosphopeptide corresponding to residues surrounding Ser9 of human GSK-3β. | Cell Signaling Technology (#5558). Rabbit monoclonal antibody | 1:1000 |
| p44/42 Mitogen-activated protein kinases - MAPK (ERK1/2) | 42, 44 | Synthetic peptide corresponding to residues near the C-terminus of human p44 MAP kinase. | Cell Signaling Technology (#4695). Rabbit monoclonal antibody | 1:1000 |
| Phospho-p44/42 MAPK (ERK1/2) (Thr202/Tyr204) | 42, 44 | Synthetic phosphopeptide corresponding to residues surrounding Thr202/Tyr204 of human p44 MAP kinase. | Cell Signaling Technology (#9101). Rabbit polyclonal antibody | 1:1000 |
| Glygogen synthase (GS) | 84 | Synthetic peptide corresponding to the sequence of human glycogen synthase. | Cell Signaling Technology (#3893). Rabbit polyclonal antibody | 1:1000 |
| Phospho-GS (Ser641) | 85 | Synthetic phosphopeptide corresponding to residues surrounding Ser641 of human liver glycogen synthase. | Cell Signaling Technology (#3891). Rabbit polyclonal antibody | 1:1000 |
| Janus kinase 2 or JAK2 | 125 | Synthetic peptide corresponding to residues surrounding Pro841 of Jak2. | Cell Signaling Technology (#3230). Rabbit monoclonal antibody | 1:1000 |
| Phospho-JAK2 (Tyr1008) | 125 | Synthetic peptide corresponding to residues surrounding Tyr1008 of human Jak2 protein. | Cell Signaling Technology (#8082). Rabbit monoclonal antibody | 1:1000 |
| Signal transducer and activator of transcription 3 or STAT3 | 79, 86 | Synthetic peptide centered around amino acid Gln692 of human Stat3. | Cell Signaling Technology (#91395). Mouse monoclonal antibody | 1:1000 |
| Phospho-STAT3 (Tyr703) | 79, 86 | Synthetic phosphopeptide corresponding to residues surrounding Tyr705 of mouse Stat3. | Cell Signaling Technology (#91455). Rabbit monoclonal antibody | 1:2000 |
| Signal transducer and activator of transcription 5 or STAT5 | 90 | Synthetic peptide corresponding to an amino-terminal region within Stat5. | Cell Signaling Technology (#9363). Rabbit polyclonal antibody | 1:1000 |
| Phospho-STAT5 (Tyr694) | 90 | Synthetic phosphopeptide corresponding to residues surrounding Tyr694 of mouse Stat5a. | Cell Signaling Technology (#9351). Rabbit polyclonal antibody | 1:1000 |

Abbreviations: AKT, protein kinase B; p-AKT^Ser473^, AKT phosphorylated at serine 473; AMPKα, 5' AMP-activated protein kinase; p-AMPKα^Thr172^, AMPKα phosphorylated at threonine 172; ERK1/2, extracellular signal-regulated kinase 1 and 2; p-ERK1^Thr202^/p-ERK2^Tyr204^, ERK1/2 phosphorylated at threonine 204 and tyrosine 204 respectively; GSK3β, glycogen synthase kinase-3 beta; p-GSK3β^Tyr279^, GSK3β phosphorylated at tyrosine 279; p-GSK3β^Ser609^, GSK3β phosphorylated at serine 609; IRS1, insulin receptor substrate 1; p-IRS1^Ser612^, IRS1 phosphorylated at serine 612; p-IRS1^Tyr618^, IRS1 phosphorylated at tyrosine 618; JAK2, janus kinase 2; p-JAK2^Tyr1008^, JAK2 phosphorylated at tyrosine 1008; mTOR, mammalian target of rapamycin; p-mTOR^Ser2448^, mTOR phosphorylated at serine 2448; PI3K, phosphoinositide 3-kinase; p-PI3K^Tyr607^, PI3K phosphorylated at tyrosine 607; STAT3/5, signal transducer and activator of transcription 3 and 5; p-STAT3^Tyr703^/p-STAT5^Tyr694^, STAT3/5 phosphorylated at tyrosines 703 and 694 respectively.
