# Supplementary material for: Sex-based differences in growth-related IGF1 signaling in response to PAPP-A2 deficiency: comparative effects of rhGH, rhIGF1 and rhPAPP-A2 treatments
Source: Biol Sex Differ. 2024 Apr 8;15:34. doi: 10.1186/s13293-024-00603-5 (PMC11000399; doi:10.1186/s13293-024-00603-5)
Supplement: Supplementary file 4 — Supplementary Material 4 [file 13293_2024_603_MOESM4_ESM.docx]

**Supplementary Table S3.** Interactions and main effects of treatment (rhGH, rhIGF1 and rhPAPP-A2), genotype (*Pappa2*^wt/wt^ and *Pappa2*^ko/ko^) and sex (males and females) on plasma levels of total IGF1 and IGFBP5.

| **A** | **rhGH treatment** | |  | **B** | **rhIGF1 treatment** | |  | **C** | **rhPAPPA2 treatment** | |
| --- | --- | --- | --- | --- | --- | --- | --- | --- | --- | --- |
| **Three-way ANOVA** | **IGF1** | **IGFBP5** |  | **Three-way ANOVA** | **IGF1** | **IGFBP5** |  | **Three-way ANOVA** | **IGF1** | **IGFBP5** |
| **Genotype (G)** | *F*1,67=12.8 *P=*.001 | *F*1,68=22.4 *P<*.001 |  | **Genotype (G)** | *F*1,65=4.89 *P=*.031 | *F*1,63=43.0 *P<*.001 |  | **Genotype (G)** | *F*1,65=9.66 *P=*.003 | *F*1,64=18-8 *P=*.001 |
| **Sex (S)** | *F*1,67=33.4 *P<*.001 | *F*1,68=73.7 *P<*.001 |  | **Sex (S)** | *F*1,65=32.1 *P<*.001 | *F*1,62=34.2 *P<*.001 |  | **Sex (S)** | *F*1,65=24.8 *P<*.001 | *F*1,64=51.9 *P<*.001 |
| **Treatment**  **(T)** | *F*1,67=12.1 *P=*.001 | *F*1,68=14.7 *P<*.001 |  | **Treatment**  **(T)** | *F*1,65=39.8 *P<*.001 | *ns* |  | **Treatment**  **(T)** | *F*1,65=82.4 *P<*.001 | *ns* |
| **G*S** | *F*1,67=26.6 *P<*.001 | *ns* |  | **G*S** | *F*1,65=25.0 *P<*.001 | *F*1,63=7.92 *P=*.006 |  | **G*S** | *F*1,65=15.0 *P<*.001 | *ns* |
| **T*G** | *ns* | *F*1,68=13.3 *P=*.001 |  | **T*G** | *ns* | *F*1,63=33.8 *P<*.001 |  | **T*G** | *ns* | *F*1,64=5.15 *P=*.027 |
| **T*S** | *ns* | *ns* |  | **T*S** | *F*1,65=6.45 *P=*.014 | *ns* |  | **T*S** | *F*1,65=17.8 *P<*.001 | *ns* |
| **T*G*S** | *ns* | *ns* |  | **T*G*S** | *ns* | *ns* |  | **T*G*S** | *F*1,65=5.47 *P=*.022 | *ns* |
|  |  |  |  |  |  |  |  |  |  |  |
| **D** | **rhGH treatment in males** | |  | **E** | **rhIGF1 treatment in males** | |  | **F** | **rhPAPPA2 treatment in males** | |
| **Two-way ANOVA** | **IGF1** | **IGFBP5** |  | **Two-way ANOVA** | **IGF1** | **IGFBP5** |  | **Two-way ANOVA** | **IGF1** | **IGFBP5** |
| **Genotype (G)** | *F*1,37=39.0 *P<*.001 | *F*1,37=34.8 *P<.*001 |  | **Genotype (G)** | *F*1,34=21.4 *P<*.001 | *F*1,34=69.9 *P<*.001 |  | **Genotype (G)** | *F*1,35=23.8 *P<*.001 | *F*1,35=14.0 *P=*.001 |
| **Treatment (T)** | *ns* | *F*1,37=6.83 *P=*.013 |  | **Treatment (T)** | *F*1,34=32.2 *P<*.001 | *ns* |  | **Treatment (T)** | *F*1,35=86.2 *P<*.001 | *ns* |
| **T*G** | *ns* | *ns* |  | **T*G** | *ns* | *F*1,34=24.7 *P<*.001 |  | **T*G** | *F*1,35=4.90 *P=*.033 | *ns* |
|  |  |  |  |  |  |  |  |  |  |  |
| **G** | **rhGH treatment in females** | |  | **H** | **rhIGF1 treatment in females** | |  | **I** | **rhPAPPA2 treatment in females** | |
| **Two-way ANOVA** | **IGF1** | **IGFBP5** |  | **Two-way ANOVA** | **IGF1** | **IGFBP5** |  | **Two-way ANOVA** | **IGF1** | **IGFBP5** |
| **Genotype (G)** | *ns* | *ns* |  | **Genotype (G)** | *F*1,34=5.31 *P=*.028 | *F*1,31=4.86 *P=*.035 |  | **Genotype (G)** | *ns* | *F*1,29=5.99 *P=*.021 |
| **Treatment (T)** | *F*1,30=13.8 *P=*.001 | *F*1,31=7.52 *P=*.010 |  | **Treatment (T)** | *F*1,31=9.74 *P=*.004 | *ns* |  | **Treatment (T)** | *F*1,29=12.9 *P=*.001 | *ns* |
| **T*G** | *ns* | *F*1,31=10.1 *P=*.003 |  | **T*G** | *ns* | *F*1,34=12.8 *P=*.001 |  | **T*G** | *ns* | *ns* |
