# Supplementary material for: Sex-based differences in growth-related IGF1 signaling in response to PAPP-A2 deficiency: comparative effects of rhGH, rhIGF1 and rhPAPP-A2 treatments
Source: Biol Sex Differ. 2024 Apr 8;15:34. doi: 10.1186/s13293-024-00603-5 (PMC11000399; doi:10.1186/s13293-024-00603-5)
Supplement: Supplementary file 5 — Supplementary Material 5 [file 13293_2024_603_MOESM5_ESM.docx]

**Supplementary Table S4.** Interactions and main effects of treatment (rhGH, rhIGF1 and rhPAPP-A2), genotype (*Pappa2*^wt/wt^ and *Pappa2*^ko/ko^) and sex (males and females) on liver gene expression of IGF1 system components.

| **A** | **rhGH treatment** | | | | | | | |
| --- | --- | --- | --- | --- | --- | --- | --- | --- |
| **Three-way ANOVA** | ***Igf1*** | ***Ghr*** | ***Igf1r*** | ***Igfbp3*** | ***Igfbp5*** | ***Igfals*** | ***Stc1*** | ***Stc2*** |
| **Genotype (G)** | *ns* | *ns* | *ns* | *F*1,62=4.51 *P=*.038 | *ns* | *ns* | *F*1,60=21.1 *P<*.001 | *ns* |
| **Sex (S)** | *ns* | *F*1,62=22.7 *P<*.001 | *F*1,62=49.5 *P<*.001 | *ns* | *F*1,60=18.9 *P<*.001 | *F*1,60=28.7 *P<*.001 | *ns* | *ns* |
| **Treatment**  **(T)** | *F*1,62=4.26 *P=*.044 | *ns* | *F*1,62=5.46 *P=*.023 | *F*1,62=56.3 *P<*.001 | *ns* | *F*1,60=24.6 *P<*.001 | *ns* | *ns* |
| **G*S** | *ns* | *F*1,62=4.53 *P=*.038 | *ns* | *F*1,62=9.41 *P=*.003 | *ns* | *ns* | *ns* | *ns* |
| **T*G** | *ns* | *F*1,62=7.12 *P=*.010 | *ns* | *ns* | *ns* | *ns* | *ns* | *ns* |
| **T*S** | *ns* | *F*1,62=15.1 *P<*.001 | *F*1,62=30.1 *P<*.001 | *ns* | *ns* | *F*1,60=15.6 *P<*.001 | *F*1,60=7.94 *P=*.007 | *F*1,58=5.70 *P=*.021 |
| **T*G*S** | *ns* | *ns* | *ns* | *ns* | *ns* | *ns* | *F*1,60=6.39 *P=*.015 | *ns* |
|  |  |  |  |  |  |  |  |  |
| **B** | **rhIGF1 treatment** | | | | | | | |
| **Three-way ANOVA** | ***Igf1*** | ***Ghr*** | ***Igf1r*** | ***Igfbp3*** | ***Igfbp5*** | ***Igfals*** | ***Stc1*** | ***Stc2*** |
| **Genotype (G)** | *ns* | *ns* | *ns* | *F*1,63=8.15 *P=*.006 | *F*1,59=5.24 *P=*.026 | *ns* | *F*1,58=10.4 *P=*.002 | *ns* |
| **Sex (S)** | *ns* | *F*1,62=23.5 *P<*.001 | *F*1,62=30.9 *P<*.001 | *F*1,63=13.7 *P<*.001 | *F*1,59=10.5 *P=*.002 | *F*1,61=22.7 *P<*.001 | *ns* | *ns* |
| **Treatment**  **(T)** | *F*1,63=10.2 *P=*.002 | *ns* | *ns* | *F*1,63=8.52 *P=*.005 | *F*1,59=6.66 *P=*.013 | *F*1,61=8.82 *P=*.004 | *ns* | *F*1,57=15.4 *P<*.001 |
| **G*S** | *ns* | *ns* | *ns* | *F*1,63=8.58 *P=*.005 | *ns* | *F*1,61=6.76 *P=*.012 | *ns* | *ns* |
| **T*G** | *ns* | *F*1,63=6.89 *P=*.011 | *ns* | *ns* | *ns* | *ns* | *ns* | *ns* |
| **T*S** | *ns* | *F*1,63=15.9 *P<*.001 | *F*1,62=17.1 *P<*.001 | *ns* | *ns* | *F*1,61=8.70 *P=*.005 | *F*1,58=9.22 *P=*.004 | *ns* |
| **T*G*S** | *ns* | *F*1,63=6.50 *P=*.014 | *ns* | *ns* | *ns* | *ns* | *F*1,58=9.70 *P=*.003 | *ns* |
|  |  |  |  |  |  |  |  |  |
| **C** | **rhPAPP-A2 treatment** | | | | | | | |
| **Three-way ANOVA** | ***Igf1*** | ***Ghr*** | ***Igf1r*** | ***Igfbp3*** | ***Igfbp5*** | ***Igfals*** | ***Stc1*** | ***Stc2*** |
| **Genotype (G)** | *ns* | *ns* | *ns* | *ns* | *ns* | *ns* | *F*1,57=10.3 *P=*.002 | *ns* |
| **Sex (S)** | *ns* | *F*1,59=30.6 *P<*.001 | *F*1,59=47.5 *P<*.001 | *F*1,60=13.8 *P<*.001 | *F*1,58=6.05 *P=*.017 | *F*1,58=15.9 *P<*.001 | *ns* | *ns* |
| **Treatment**  **(T)** | *F*1,60=34.7 *P<*.001 | *ns* | *ns* | *ns* | *ns* | *ns* | *ns* | *F*1,57=6.68 *P=*.013 |
| **G*S** | *ns* | *ns* | *ns* | *F*1,60=4.65 *P=*.036 | *ns* | *F*1,58=4.86 *P=*.032 | *ns* | *ns* |
| **T*G** | *ns* | *F*1,59=20.6 *P<*.001 | *F*1,59=4.64 *P=*.036 | *F*1,60=5.38 *P=*.024 | *ns* | *ns* | *ns* | *ns* |
| **T*S** | *ns* | *F*1,59=20.7 *P<*.001 | *F*1,59=26.0 *P<*.001 | *ns* | *ns* | *ns* | *F*1,57=6.61 *P=*.013 | *F*1,57=5.29 *P=*.026 |
| **T*G*S** | *ns* | *F*1,59=11.9 *P=*.001 | *ns* | *ns* | *ns* | *ns* | *F*1,57=7.75 *P=*.008 | *ns* |
