# Supplementary material for: Sex-based differences in growth-related IGF1 signaling in response to PAPP-A2 deficiency: comparative effects of rhGH, rhIGF1 and rhPAPP-A2 treatments
Source: Biol Sex Differ. 2024 Apr 8;15:34. doi: 10.1186/s13293-024-00603-5 (PMC11000399; doi:10.1186/s13293-024-00603-5)
Supplement: Supplementary file 6 — Supplementary Material 6 [file 13293_2024_603_MOESM6_ESM.docx]

**Supplementary Table S5.** Interactions and main effects of treatment (rhGH, rhIGF1 and rhPAPP-A2) and genotype (*Pappa2*^wt/wt^ and *Pappa2*^ko/ko^) on liver gene expression of IGF1 system components by analyzing the sexes separately.

| **A** | **rhGH treatment in males** | | | | | | | |
| --- | --- | --- | --- | --- | --- | --- | --- | --- |
| **Two-way ANOVA** | ***Igf1*** | ***Ghr*** | ***Igf1r*** | ***Igfbp3*** | ***Igfbp5*** | ***Igfals*** | ***Stc1*** | ***Stc2*** |
| **Genotype (G)** | *ns* | *ns* | *ns* | *ns* | *ns* | *ns* | *F*1,27=21.1 *P<*.001 | *ns* |
| **Treatment**  **(T)** | *ns* | *ns* | *F*1,30=6.24 *P=*.019 | *F*1,30=22.5 *P<*.001 | *ns* | *ns* | *F*1,27=5.57 *P=*.027 | *F*1,27=5.57 *P=*.030 |
| **T*G** | *ns* | *F*1,30=12.1 *P=*.002 | *ns* | *ns* | *ns* | *ns* | *ns* | *ns* |
| **B** | **rhGH treatment in females** | | | | | | | |
| **Two-way ANOVA** | ***Igf1*** | ***Ghr*** | ***Igf1r*** | ***Igfbp3*** | ***Igfbp5*** | ***Igfals*** | ***Stc1*** | ***Stc2*** |
| **Genotype (G)** | *ns* | *ns* | *ns* | *F*1,31=18.6 *P<*.001 | *ns* | *ns* | *ns* | *ns* |
| **Treatment**  **(T)** | *ns* | *F*1,31=14.0 *P=*.001 | *F*1,31=25.8 *P<*.001 | *F*1,31=37.2 *P<*.001 | *ns* | *F*1,31=26.1 *P<*.001 | *ns* | *ns* |
| **T*G** | *ns* | *ns* | *ns* | *ns* | *ns* | *ns* | *ns* | *ns* |
|  |  |  |  |  |  |  |  |  |
| **C** | **rhIGF1 treatment in males** | | | | | | | |
| **Two-way ANOVA** | ***Igf1*** | ***Ghr*** | ***Igf1r*** | ***Igfbp3*** | ***Igfbp5*** | ***Igfals*** | ***Stc1*** | ***Stc2*** |
| **Genotype (G)** | *ns* | *ns* | *ns* | *ns* | *ns* | *ns* | *F*1,27=13.6 *P=*.001 | *ns* |
| **Treatment**  **(T)** | *F*1,31=11.5 *P=*.002 | *F*1,31=5.80 *P=*.023 | *ns* | *F*1,31=8.81 *P=*.006 | *F*1,28=5.18 *P=*.032 | *ns* | *ns* | *F*1,27=15.3 *P=*.001 |
| **T*G** | *ns* | *F*1,31=10.7 *P=*.003 | *ns* | *ns* | *ns* | *ns* | *F*1,27=14.5 *P*=.001 | *ns* |
| **D** | **rhIGF1 treatment in females** | | | | | | | |
| **Two-way ANOVA** | ***Igf1*** | ***Ghr*** | ***Igf1r*** | ***Igfbp3*** | ***Igfbp5*** | ***Igfals*** | ***Stc1*** | ***Stc2*** |
| **Genotype (G)** | *ns* | *ns* | *ns* | *F*1,31=19.4 *P<*.001 | *ns* | *F*1,30=6.24 *P=*.019 | *ns* | *ns* |
| **Treatment**  **(T)** | *ns* | *F*1,31=11.7 *P=*.002 | *F*1,31=13.5 *P=*.002 | *ns* | *ns* | *F*1,31=12.1 *P=*.002 | *F*1,31=8.18 *P=*.008 | *ns* |
| **T*G** | *ns* | *ns* | *ns* | *ns* | *ns* | *ns* | *ns* | *ns* |
|  |  |  |  |  |  |  |  |  |
| **E** | **rhPAPP-A2 treatment in males** | | | | | | | |
| **Two-way ANOVA** | ***Igf1*** | ***Ghr*** | ***Igf1r*** | ***Igfbp3*** | ***Igfbp5*** | ***Igfals*** | ***Stc1*** | ***Stc2*** |
| **Genotype (G)** | *ns* | *ns* | *ns* | *ns* | *ns* | *ns* | *F*1,28=20.7 *P<*.001 | *ns* |
| **Treatment**  **(T)** | *F*1,30=33.2 *P<*.001 | *F*1,29=10.1 *P=*.004 | *F*1,30=8.11 *P=*.008 | *ns* | *ns* | *ns* | *ns* | *F*1,28=13.0 *P=*.001 |
| **T*G** | *ns* | *F*1,29=37.2 *P<*.001 | *ns* | *ns* | *ns* | *ns* | *F*1,28=16.4 *P<*.001 | *ns* |
| **F** | **rhPAPP-A2 treatment in females** | | | | | | | |
| **Two-way ANOVA** | ***Igf1*** | ***Ghr*** | ***Igf1r*** | ***Igfbp3*** | ***Igfbp5*** | ***Igfals*** | ***Stc1*** | ***Stc2*** |
| **Genotype (G)** | *ns* | *ns* | *ns* | *F*1,29=5.53 *P=*.027 | *ns* | *ns* | *ns* | *ns* |
| **Treatment**  **(T)** | *F*1,29=12.0 *P=*.002 | *F*1,29=10.5 *P=*.003 | *F*1,29=21.2 *P<*.001 | *ns* | *ns* | *ns* | *F*1,29=5.45 *P=*.028 | *ns* |
| **T*G** | *ns* | *ns* | *F*1,29=5.53 *P=*.027 | *F*1,29=7.70 *P=*.010 | *ns* | *ns* | *ns* | *ns* |
