# Supplementary material for: Sex-based differences in growth-related IGF1 signaling in response to PAPP-A2 deficiency: comparative effects of rhGH, rhIGF1 and rhPAPP-A2 treatments
Source: Biol Sex Differ. 2024 Apr 8;15:34. doi: 10.1186/s13293-024-00603-5 (PMC11000399; doi:10.1186/s13293-024-00603-5)
Supplement: Supplementary file 7 — Supplementary Material 7 [file 13293_2024_603_MOESM7_ESM.docx]

**Supplementary Table S6.** Interaction and main effects of treatment (rhGH, rhIGF1 and rhPAPP-A2), genotype (*Pappa2*wt/wt and *Pappa2*ko/ko) and sex (males and females) on liver protein and phosphoprotein expression of key intracellular signaling pathway regulators.

| **A** | **rhGH treatment** | | | | | | | | | | | | | |
| --- | --- | --- | --- | --- | --- | --- | --- | --- | --- | --- | --- | --- | --- | --- |
| **Three-way ANOVA** | **IRS1-T/**  **Adaptin-γ** | **IRS1-PTyr/ IRS1-PSer** | **PI3K-T/**  **Adaptin-γ** | **PI3K-PTyr/ PI3K-T** | **AKT-T/**  **Adaptin-γ** | **AKT-PSer/ AKT-T** | **mTOR-T/**  **Adaptin-γ** | **mTOR-PSer/ mTOR-T** | **GSK3β-T/**  **Adaptin-γ** | **GSK3β-PTyr/ GSK3β-PSer** | **ERK2-T/**  **Adaptin-γ** | **ERK2-PTyr/ ERK2-T** | **AMPKα-T/**  **Adaptin-γ** | **AMPKα-PThr/ AMPKα-T** |
| **Genotype (G)** | *ns* | *ns* | *ns* | *ns* | *ns* | *ns* | *F*1,45=9.30 *P=*.004 | *F*1,45=19.9 *P<*.001 | *F*1,46=5.79 *P=*.021 | *ns* | *F*1,45=4.73 *P=*.036 | *F*1,47=8.62 *P=*.005 | *ns* | *ns* |
| **Sex (S)** | *ns* | *ns* | *ns* | *ns* | *ns* | *ns* | *ns* | *ns* | *F*1,46=12.6 *P<*.001 | *F*1,46=5.45 *P=*.025 | *ns* | *ns* | *ns* | *ns* |
| **Treatment (T)** | *ns* | *F*1,40=9.40 *P=*.004 | *ns* | *ns* | *ns* | *ns* | *ns* | *ns* | *ns* | *ns* | *ns* | *ns* | *ns* | *ns* |
| **G*S** | *ns* | *F*1,40=4.41 *P=*.043 | *ns* | *ns* | *F*1,47=6.55 *P=*.014 | *ns* | *ns* | *ns* | *ns* | *F*1,46=6.97 *P=*.012 | *ns* | *F*1,47=7.22 *P=*.010 | ns | ns |
| **T*G** | *ns* | *ns* | *ns* | *ns* | *ns* | *ns* | *ns* | *ns* | *ns* | *ns* | *ns* | *ns* | *ns* | *ns* |
| **T*S** | *ns* | *F*1,40=13.5 *P=*.001 | *ns* | *ns* | *ns* | *ns* | *F*1,45=4.67 *P=*.037 | *ns* | *ns* | *ns* | *ns* | *ns* | *F*1,45=7.55 *P=*.009 | *F*1,46=6.38 *P=*.016 |
| **T*G*S** | *ns* | *F*1,40=4.42 *P=*.043 | *ns* | *ns* | *ns* | *ns* | *ns* | *ns* | *ns* | *ns* | *ns* | *ns* | ns | *ns* |
|  |  |  |  |  |  |  |  |  |  |  |  |  |  |  |
| **B** | **rhIGF1 treatment** | | | | | | | | | | | | | |
| **Three-way ANOVA** | **IRS1-T/**  **Adaptin-γ** | **IRS1-PTyr/ IRS1-PSer** | **PI3K-T/**  **Adaptin-γ** | **PI3K-PTyr/ PI3K-T** | **AKT-T/**  **Adaptin-γ** | **AKT-PSer/ AKT-T** | **mTOR-T/**  **Adaptin-γ** | **mTOR-PSer/ mTOR-T** | **GSK3β-T/**  **Adaptin-γ** | **GSK3β-PTyr/ GSK3β-PSer** | **ERK2-T/**  **Adaptin-γ** | **ERK2-PTyr/ ERK2-T** | **AMPKα-T/**  **Adaptin-γ** | **AMPKα-PThr/ AMPKα-T** |
| **Genotype (G)** | *ns* | *ns* | *ns* | *ns* | *ns* | *ns* | *ns* | *ns* | *ns* | *ns* | *F*1,48=5.34 *P=*.026 | *F*1,48=10.6 *P=*.002 | *ns* | *ns* |
| **Sex (S)** | *ns* | *F*1,40=7.37 *P=*.010 | *ns* | *ns* | *ns* | *ns* | *ns* | *ns* | *F*1,44=48.4 *P<*.001 | *ns* | *ns* | *ns* | *ns* | *ns* |
| **Treatment (T)** | *ns* | *ns* | *ns* | *ns* | *ns* | *ns* | *ns* | *ns* | *ns* | *ns* | *ns* | *ns* | *ns* | *ns* |
| **G*S** | *F*1,40=4.10 *P=*.050 | *ns* | *ns* | *ns* | *F*1,47=6.55 *P=*.014 | *ns* | *ns* | *ns* | *F*1,44=9.60 *P=*.004 | *F*1,47=7.03 *P=*.011 | *ns* | *ns* | ns | *ns* |
| **T*G** | *ns* | *ns* | *F*1,47=3.87 *P=*.056 | *ns* | *ns* | *ns* | *ns* | *ns* | *ns* | *ns* | *ns* | *ns* | *ns* | *ns* |
| **T*S** | *ns* | *ns* | *F*1,47=4.15 *P=*.048 | *ns* | *ns* | *ns* | *ns* | *ns* | *ns* | *F*1,47=5.90 *P=*.020 | *ns* | *ns* | ns | *ns* |
| **T*G*S** | *ns* | *ns* | *F*1,47=11.6 *P=*.002 | *F*1,47=4.80 *P=*.034 | *ns* | *ns* | *ns* | *ns* | *ns* | *ns* | *ns* | *ns* | *ns* | *ns* |
|  |  |  |  |  |  |  |  |  |  |  |  |  |  |  |
| **C** | **rhPAPP-A2 treatment** | | | | | | | | | | | | | |
| **Three-way ANOVA** | **IRS1-T/**  **Adaptin-γ** | **IRS1-PTyr/ IRS1-PSer** | **PI3K-T/**  **Adaptin-γ** | **PI3K-PTyr/ PI3K-T** | **AKT-T/**  **Adaptin-γ** | **AKT-PSer/ AKT-T** | **mTOR-T/**  **Adaptin-γ** | **mTOR-PSer/ mTOR-T** | **GSK3β-T/**  **Adaptin-γ** | **GSK3β-PTyr/ GSK3β-PSer** | **ERK2-T/**  **Adaptin-γ** | **ERK2-PTyr/ ERK2-T** | **AMPKα-T/**  **Adaptin-γ** | **AMPKα-PThr/ AMPKα-T** |
| **Genotype (G)** | *ns* | *ns* | *ns* | *ns* | *ns* | *ns* | *ns* | *ns* | *ns* | *ns* | *ns* | *ns* | *F*1,45=4.75 *P=.*036 | *ns* |
| **Sex (S)** | *ns* | *F*1,40=12.4 *P=*.001 | *F*1,48=3.77 *P=*.059 | *ns* | *ns* | *ns* | *ns* | *ns* | *F*1,46=22.1 *P<*.001 | *ns* | *ns* | *ns* | *ns* | *F*1,45=10.4 *P=.*003 |
| **Treatment (T)** | *ns* | *F*1,40=7.03 *P=*.012 | *ns* | *ns* | *ns* | *ns* | *ns* | *ns* | *ns* | *ns* | *ns* | *ns* | *ns* | *F*1,45=10.2 *P=.*003 |
| **G*S** | *ns* | *ns* | *ns* | *ns* | *F*1,47=6.55 *P=*.014 | *ns* | *ns* | *ns* | *F*1,46=5.68 *P=*.022 | *F*1,46=7.38 *P=*.010 | *F*1,46=4.33 *P=*.044 | *ns* | *ns* | *ns* |
| **T*G** | *ns* | *ns* | *ns* | *ns* | *ns* | *ns* | *ns* | *ns* | *ns* | *ns* | *ns* | *F*1,46=9.24 *P=*.004 | *ns* | *ns* |
| **T*S** | *ns* | *ns* | *ns* | *ns* | *ns* | *ns* | *F*1,44=6.08 *P=*.018 | *ns* | *ns* | *ns* | *ns* | *ns* | *F*1,45=6.88 *P=.*012 | *F*1,45=16.5 *P<.*001 |
| **T*G*S** | *ns* | *ns* | *ns* | *ns* | *ns* | *ns* | *ns* | *ns* | *ns* | *ns* | *ns* | *ns* | *ns* | *ns* |
