# Supplementary material for: Sex-based differences in growth-related IGF1 signaling in response to PAPP-A2 deficiency: comparative effects of rhGH, rhIGF1 and rhPAPP-A2 treatments
Source: Biol Sex Differ. 2024 Apr 8;15:34. doi: 10.1186/s13293-024-00603-5 (PMC11000399; doi:10.1186/s13293-024-00603-5)
Supplement: Supplementary file 8 — Supplementary Material 8 [file 13293_2024_603_MOESM8_ESM.docx]

**Supplementary Table S7.** Interaction and main effects of treatment (rhGH, rhIGF1 and rhPAPP-A2) and genotype (*Pappa2*wt/wt and *Pappa2*ko/ko) on liver protein and phosphoprotein expression of key intracellular signaling pathway regulators by analyzing the sexes separately.

| **A** | **rhGH treatment in males** | | | | | | | | | | | | | |
| --- | --- | --- | --- | --- | --- | --- | --- | --- | --- | --- | --- | --- | --- | --- |
| **Two-way ANOVA** | **IRS1-T/**  **Adaptin-γ** | **IRS1-PTyr/ IRS1-PSer** | **PI3K-T/**  **Adaptin-γ** | **PI3K-PTyr/ PI3K-T** | **AKT-T/**  **Adaptin-γ** | **AKT-PSer/ AKT-T** | **mTOR-T/**  **Adaptin-γ** | **mTOR-PSer/ mTOR-T** | **GSK3β-T/**  **Adaptin-γ** | **GSK3β-PTyr/ GSK3β-PSer** | **ERK2-T/**  **Adaptin-γ** | **ERK2-PTyr/ ERK2-T** | **AMPKα-T/**  **Adaptin-γ** | **AMPKα-PThr/ AMPKα-T** |
| **Genotype (G)** | *ns* | *F*1,23=16.1 *P=.001* | *ns* | *ns* | *ns* | *ns* | *F*1,23=10.2 *P=*.005 | *F*1,23=7.25 *P=*.014 | *F*1,23=8.56 *P=*.008 | *F*1,23=4.74 *P=*.042 | *ns* | *ns* | *ns* | *ns* |
| **Treatment (T)** | *ns* | *F*1,23=87.8 *P<*.001 | *ns* | *ns* | *ns* | *ns* | *F*1,23=6.41 *P=*.020 | *ns* | *F*1,23=4.39 *P=*.049 | *ns* | *ns* | *ns* | *ns* | *F*1,23=6.06 *P=*.023 |
| **T*G** | *ns* | *F*1,23=12.3 *P=*.002 | *ns* | *ns* | *ns* | *ns* | *ns* | *ns* | *ns* | *ns* | *ns* | *ns* | *ns* | *ns* |
| **B** | **rhGH treatment in females** | | | | | | | | | | | | | |
| **Two-way ANOVA** | **IRS1-T/**  **Adaptin-γ** | **IRS1-PTyr/ IRS1-PSer** | **PI3K-T/**  **Adaptin-γ** | **PI3K-PTyr/ PI3K-T** | **AKT-T/**  **Adaptin-γ** | **AKT-PSer/ AKT-T** | **mTOR-T/**  **Adaptin-γ** | **mTOR-PSer/ mTOR-T** | **GSK3β-T/**  **Adaptin-γ** | **GSK3β-PTyr/ GSK3β-PSer** | **ERK2-T/**  **Adaptin-γ** | **ERK2-PTyr/ ERK2-T** | **AMPKα-T/**  **Adaptin-γ** | **AMPKα-PThr/ AMPKα-T** |
| **Genotype (G)** | *ns* | *ns* | *ns* | *ns* | *ns* | *ns* | *ns* | *F*1,23=13.9 *P=*.001 | *ns* | *ns* | *F*1,23=10.5 *P=*.004 | *F*1,23=9.82 *P=*.005 | *ns* | *ns* |
| **Treatment (T)** | *ns* | *ns* | *ns* | *ns* | *ns* | *ns* | *ns* | *ns* | *ns* | *ns* | *ns* | *ns* | *F*1,23=7.99 *P=*.010 | *ns* |
| **T*G** | *ns* | *ns* | *ns* | *ns* | *ns* | *ns* | *ns* | *ns* | *ns* | *ns* | *ns* | *ns* | *ns* | *ns* |
|  |  |  |  |  |  |  |  |  |  |  |  |  |  |  |
| **C** | **rhIGF1 treatment in males** | | | | | | | | | | | | | |
| **Two-way ANOVA** | **IRS1-T/**  **Adaptin-γ** | **IRS1-PTyr/ IRS1-PSer** | **PI3K-T/**  **Adaptin-γ** | **PI3K-PTyr/ PI3K-T** | **AKT-T/**  **Adaptin-γ** | **AKT-PSer/ AKT-T** | **mTOR-T/**  **Adaptin-γ** | **mTOR-PSer/ mTOR-T** | **GSK3β-T/**  **Adaptin-γ** | **GSK3β-PTyr/ GSK3β-PSer** | **ERK2-T/**  **Adaptin-γ** | **ERK2-PTyr/ ERK2-T** | **AMPKα-T/**  **Adaptin-γ** | **AMPKα-PThr/ AMPKα-T** |
| **Genotype (G)** | *F*1,23=6.02 *P*=.023 | *ns* | *ns* | *ns* | *ns* | *ns* | *ns* | *ns* | *F*1,23=13.5 *P*=.001 | *F*1,23=9.89 *P*=.005 | *ns* | *ns* | *ns* | *ns* |
| **Treatment (T)** | *ns* | *ns* | *ns* | *ns* | *ns* | *F*1,23=9.98 *P*=.005 | *ns* | *ns* | *ns* | *ns* | *ns* | *ns* | *ns* | *F*1,23=7.44 *P=*.013 |
| **T*G** | *ns* | *ns* | *ns* | *F*1,23=6.88 *P*=.016 | *ns* | *ns* | *F*1,23=7.08 *P*=.015 | *ns* | *ns* | *ns* | *ns* | *ns* | *ns* | *ns* |
|  |  |  |  |  |  |  |  |  |  |  |  |  |  |  |
| **D** | **rhIGF1 treatment in females** | | | | | | | | | | | | | |
| **Two-way ANOVA** | **IRS1-T/**  **Adaptin-γ** | **IRS1-PTyr/ IRS1-PSer** | **PI3K-T/**  **Adaptin-γ** | **PI3K-PTyr/ PI3K-T** | **AKT-T/**  **Adaptin-γ** | **AKT-PSer/ AKT-T** | **mTOR-T/**  **Adaptin-γ** | **mTOR-PSer/ mTOR-T** | **GSK3β-T/**  **Adaptin-γ** | **GSK3β-PTyr/ GSK3β-PSer** | **ERK2-T/**  **Adaptin-γ** | **ERK2-PTyr/ ERK2-T** | **AMPKα-T/**  **Adaptin-γ** | **AMPKα-PThr/ AMPKα-T** |
| **Genotype (G)** | *ns* | *ns* | *ns* | *ns* | *ns* | *ns* | *ns* | *ns* | *ns* | *ns* | *ns* | *F*1,23=7.69 *P=*.012 | *ns* | *ns* |
| **Treatment (T)** | *ns* | *ns* | *ns* | *ns* | *ns* | *ns* | *ns* | *ns* | *ns* | *ns* | *ns* | *ns* | *ns* | *ns* |
| **T*G** | *ns* | *ns* | *F*1,23=12.8 *P=*.002 | *ns* | *ns* | *F*1,23=5.70 *P=*.027 | *ns* | *ns* | *ns* | *ns* | *ns* | *ns* | *ns* | *ns* |
|  |  |  |  |  |  |  |  |  |  |  |  |  |  |  |
| **E** | **rhPAPP-A2 treatment in males** | | | | | | | | | | | | | |
| **Two-way ANOVA** | **IRS1-T/**  **Adaptin-γ** | **IRS1-PTyr/ IRS1-PSer** | **PI3K-T/**  **Adaptin-γ** | **PI3K-PTyr/ PI3K-T** | **AKT-T/**  **Adaptin-γ** | **AKT-PSer/ AKT-T** | **mTOR-T/**  **Adaptin-γ** | **mTOR-PSer/ mTOR-T** | **GSK3β-T/**  **Adaptin-γ** | **GSK3β-PTyr/ GSK3β-PSer** | **ERK2-T/**  **Adaptin-γ** | **ERK2-PTyr/ ERK2-T** | **AMPKα-T/**  **Adaptin-γ** | **AMPKα-PThr/ AMPKα-T** |
| **Genotype (G)** | *ns* | *ns* | *ns* | *ns* | *ns* | *ns* | *ns* | *ns* | *F*1,23=11.4 *P*=.003 | *F*1,23=11.1 *P*=.003 | *ns* | *ns* | *ns* | *ns* |
| **Treatment (T)** | *ns* | *F*1,23=4.70 *P*=.042 | *F*1,23=5.37 *P*=.031 | *ns* | *ns* | *F*1,23=7.65 *P*=.012 | *ns* | *ns* | *F*1,23=4.92 *P*=.038 | *ns* | *ns* | *ns* | *F*1,23=6.64 *P*=.018 | *F*1,23=18.9 *P<*.001 |
| **T*G** | *ns* | *ns* | *ns* | *ns* | *ns* | *ns* | *ns* | *ns* | *ns* | *ns* | *ns* | *ns* | *ns* | *ns* |
| **F** | **rhPAPP-A2 treatment in females** | | | | | | | | | | | | | |
| **Two-way ANOVA** | **IRS1-T/**  **Adaptin-γ** | **IRS1-PTyr/ IRS1-PSer** | **PI3K-T/**  **Adaptin-γ** | **PI3K-PTyr/ PI3K-T** | **AKT-T/**  **Adaptin-γ** | **AKT-PSer/ AKT-T** | **mTOR-T/**  **Adaptin-γ** | **mTOR-PSer/ mTOR-T** | **GSK3β-T/**  **Adaptin-γ** | **GSK3β-PTyr/ GSK3β-PSer** | **ERK2-T/**  **Adaptin-γ** | **ERK2-PTyr/ ERK2-T** | **AMPKα-T/**  **Adaptin-γ** | **AMPKα-PThr/ AMPKα-T** |
| **Genotype (G)** | *ns* | *ns* | *ns* | *ns* | *ns* | *ns* | *ns* | *ns* | *ns* | *ns* | *F*1,23=7.45 *P=*.013 | *ns* | *ns* | *ns* |
| **Treatment (T)** | *ns* | *F*1,23=4.92 *P=*.038 | *ns* | *ns* | *ns* | *F*1,23=4.85 *P=*.039 | *F*1,23=9.05 *P=*.007 | *ns* | *ns* | *ns* | *ns* | *ns* | *ns* | *ns* |
| **T*G** | *ns* | *ns* | *ns* | *ns* | *ns* | *ns* | *ns* | *ns* | *ns* | *ns* | *ns* | *F*1,23=6.34 *P=*.020 | *ns* | *F*1,23=5.42 *P=*.031 |
