# Supplementary material for: Sex-based differences in growth-related IGF1 signaling in response to PAPP-A2 deficiency: comparative effects of rhGH, rhIGF1 and rhPAPP-A2 treatments
Source: Biol Sex Differ. 2024 Apr 8;15:34. doi: 10.1186/s13293-024-00603-5 (PMC11000399; doi:10.1186/s13293-024-00603-5)
Supplement: Supplementary file 9 — Supplementary Material 9 [file 13293_2024_603_MOESM9_ESM.docx]

**Supplementary Table S8.** Interaction and main effects of treatment (rhGH, rhIGF1 and rhPAPP-A2), genotype (*Pappa2*wt/wt and *Pappa2*ko/ko) and sex (males and females) on liver protein and phosphoprotein expression of JAK2-STAT3-STAT5 signaling.

| **A** | **rhGH treatment** | | | | | |
| --- | --- | --- | --- | --- | --- | --- |
| **Three-way ANOVA** | **JAK2-T/**  **Adaptin-γ** | **JAK2-PTyr/ JAK2-T** | **STAT3-T/**  **Adaptin-γ** | **STAT3-PTyr/ STAT3-T** | **STAT5-T/**  **Adaptin-γ** | **STAT5-PTyr/ STAT5-T** |
| **Genotype (G)** | *ns* | *ns* | *ns* | *ns* | *ns* | *ns* |
| **Sex (S)** | *ns* | *F*1,47=4.42 *P=*.042 | *ns* | *ns* | *ns* | *ns* |
| **Treatment (T)** | *ns* | *ns* | *ns* | *ns* | *ns* | *ns* |
| **G*S** | *ns* | *ns* | *ns* | *F*1,47=5.56 *P=*.023 | *ns* | *ns* |
| **T*G** | *ns* | *ns* | *ns* | *ns* | *ns* | *ns* |
| **T*S** | *ns* | *ns* | *ns* | *ns* | *ns* | *ns* |
| **T*G*S** | *ns* | *ns* | *ns* | *ns* | *ns* | *ns* |
|  |  |  |  |  |  |  |
| **B** | **rhIGF1 treatment** | | | | | |
| **Three-way ANOVA** | **JAK2-T/**  **Adaptin-γ** | **JAK2-PTyr/ JAK2-T** | **STAT3-T/**  **Adaptin-γ** | **STAT3-PTyr/ STAT3-T** | **STAT5-T/**  **Adaptin-γ** | **STAT5-PTyr/ STAT5-T** |
| **Genotype (G)** | *ns* | *ns* | *ns* | *ns* | *ns* | *ns* |
| **Sex (S)** | *ns* | *ns* | *ns* | *ns* | *ns* | *ns* |
| **Treatment (T)** | *ns* | *ns* | *ns* | *ns* | *ns* | *ns* |
| **G*S** | *ns* | *ns* | *ns* | *ns* | *ns* | *ns* |
| **T*G** | *ns* | *ns* | *ns* | *ns* | *ns* | *F*1,47=4.65 *P=*.037 |
| **T*S** | *ns* | *ns* | *ns* | *ns* | *ns* | *ns* |
| **T*G*S** | *ns* | *ns* | *ns* | *ns* | *ns* | *ns* |
|  |  |  |  |  |  |  |
| **C** | **rhPAPP-A2 treatment** | | | | | |
| **Three-way ANOVA** | **JAK2-T/**  **Adaptin-γ** | **JAK2-PTyr/ JAK2-T** | **STAT3-T/**  **Adaptin-γ** | **STAT3-PTyr/ STAT3-T** | **STAT5-T/**  **Adaptin-γ** | **STAT5-PTyr/ STAT5-T** |
| **Genotype (G)** | *ns* | *ns* | *ns* | *ns* | *ns* | *F*1,47=4.28 *P=*.045 |
| **Sex (S)** | *ns* | *ns* | *ns* | *ns* | *ns* | *F*1,47=4.71 *P=*.036 |
| **Treatment (T)** | *ns* | *ns* | *ns* | *ns* | *ns* | *F*1,47=6.83 *P=*.013 |
| **G*S** | *ns* | *ns* | *ns* | *ns* | *ns* | *ns* |
| **T*G** | *ns* | *ns* | *ns* | *ns* | *ns* | *F*1,47=4.23 *P=*.047 |
| **T*S** | *ns* | *ns* | *ns* | *ns* | *ns* | *ns* |
| **T*G*S** | *ns* | *ns* | *ns* | *ns* | *ns* | *ns* |
