# Supplementary material for: Sex-based differences in growth-related IGF1 signaling in response to PAPP-A2 deficiency: comparative effects of rhGH, rhIGF1 and rhPAPP-A2 treatments
Source: Biol Sex Differ. 2024 Apr 8;15:34. doi: 10.1186/s13293-024-00603-5 (PMC11000399; doi:10.1186/s13293-024-00603-5)
Supplement: Supplementary file 10 — Supplementary Material 10 [file 13293_2024_603_MOESM10_ESM.docx]

**Supplementary Table S9.** Interaction and main effects of treatment (rhGH, rhIGF1 and rhPAPP-A2) and genotype (*Pappa2*wt/wt and *Pappa2*ko/ko) on liver protein and phosphoprotein expression of JAK2-STAT3-STAT5 signaling by analyzing the sexes separately.

| **A** | **rhGH treatment in males** | | | | | |
| --- | --- | --- | --- | --- | --- | --- |
| **Two-way ANOVA** | **JAK2-T/**  **Adaptin-γ** | **JAK2-PTyr/ JAK2-T** | **STAT3-T/**  **Adaptin-γ** | **STAT3-PTyr/ STAT3-T** | **STAT5-T/**  **Adaptin-γ** | **STAT5-PTyr/ STAT5-T** |
| **Genotype (G)** | *ns* | *ns* | *ns* | *F*1,30=10.1 *P=*.004 | *ns* | *ns* |
| **Treatment (T)** | *ns* | *ns* | *ns* | *ns* | *ns* | *ns* |
| **T*G** | *ns* | *ns* | *ns* | *ns* | *ns* | *ns* |
|  |  |  |  |  |  |  |
| **C** | **rhIGF1 treatment in males** | | | | | |
| **Two-way ANOVA** | **JAK2-T/**  **Adaptin-γ** | **JAK2-PTyr/ JAK2-T** | **STAT3-T/**  **Adaptin-γ** | **STAT3-PTyr/ STAT3-T** | **STAT5-T/**  **Adaptin-γ** | **STAT5-PTyr/ STAT5-T** |
| **Genotype (G)** | *ns* | *ns* | *ns* | *ns* | *ns* | *ns* |
| **Treatment (T)** | *ns* | *ns* | *ns* | *ns* | *ns* | *ns* |
| **T*G** | *ns* | *ns* | *ns* | *ns* | *ns* | *ns* |
|  |  |  |  |  |  |  |
| **E** | **rhPAPP-A2 treatment in males** | | | | | |
| **Two-way ANOVA** | **JAK2-T/**  **Adaptin-γ** | **JAK2-PTyr/ JAK2-T** | **STAT3-T/**  **Adaptin-γ** | **STAT3-PTyr/ STAT3-T** | **STAT5-T/**  **Adaptin-γ** | **STAT5-PTyr/ STAT5-T** |
| **Genotype (G)** | *ns* | *ns* | *ns* | *ns* | *ns* | *ns* |
| **Treatment (T)** | *ns* | *ns* | *ns* | *ns* | *ns* | *ns* |
| **T*G** | *ns* | *ns* | *ns* | *ns* | *ns* | *ns* |
| **B** | **rhGH treatment in females** | | | | | |
| **Two-way ANOVA** | **JAK2-T/**  **Adaptin-γ** | **JAK2-PTyr/ JAK2-T** | **STAT3-T/**  **Adaptin-γ** | **STAT3-PTyr/ STAT3-T** | **STAT5-T/**  **Adaptin-γ** | **STAT5-PTyr/ STAT5-T** |
| **Genotype (G)** | *ns* | *ns* | *ns* | *ns* | *ns* | *F*1,31=8.24 *P=*.008 |
| **Treatment (T)** | *ns* | *ns* | *ns* | *ns* | *ns* | *F*1,31=12.8 *P=*.001 |
| **T*G** | *ns* | *ns* | *ns* | *ns* | *ns* | *F*1,31=11.2 *P=*.002 |
|  |  |  |  |  |  |  |
| **D** | **rhIGF1 treatment in females** | | | | | |
| **Two-way ANOVA** | **JAK2-T/**  **Adaptin-γ** | **JAK2-PTyr/ JAK2-T** | **STAT3-T/**  **Adaptin-γ** | **STAT3-PTyr/ STAT3-T** | **STAT5-T/**  **Adaptin-γ** | **STAT5-PTyr/ STAT5-T** |
| **Genotype (G)** | *ns* | *ns* | *ns* | *ns* | *ns* | *ns* |
| **Treatment (T)** | *ns* | *ns* | *ns* | *ns* | *ns* | *ns* |
| **T*G** | *ns* | *ns* | *ns* | *ns* | *ns* | *F*1,22=10.0 *P=*.005 |
|  |  |  |  |  |  |  |
| **F** | **rhPAPP-A2 treatment in females** | | | | | |
| **Two-way ANOVA** | **JAK2-T/**  **Adaptin-γ** | **JAK2-PTyr/ JAK2-T** | **STAT3-T/**  **Adaptin-γ** | **STAT3-PTyr/ STAT3-T** | **STAT5-T/**  **Adaptin-γ** | **STAT5-PTyr/ STAT5-T** |
| **Genotype (G)** | *ns* | *ns* | *ns* | *ns* | *ns* | *F*1,22=10. 5 *P=*.004 |
| **Treatment (T)** | *ns* | *ns* | *ns* | *ns* | *ns* | *F*1,22=4.63 *P=*.045 |
| **T*G** | *ns* | *ns* | *ns* | *ns* | *ns* | *F*1,22=8.64 *P=*.008 |
