# Supplementary material for: Sex-based differences in growth-related IGF1 signaling in response to PAPP-A2 deficiency: comparative effects of rhGH, rhIGF1 and rhPAPP-A2 treatments
Source: Biol Sex Differ. 2024 Apr 8;15:34. doi: 10.1186/s13293-024-00603-5 (PMC11000399; doi:10.1186/s13293-024-00603-5)
Supplement: Supplementary file 11 — Supplementary Material 11 [file 13293_2024_603_MOESM11_ESM.pdf]

Supplementary Figure S1.
Western Blot results in Hypothalamus

- **WT ♂/♀**
- **KO ♂/♀**

Two batches of 2 gels each:

1st batch: 13-10-2021

2º batch: 19-11-2021

1st batch

(13/10/2021)

Gel 1

Genotype	WT						WT/KO					KO					
Sex	♂			♀			♂			♀		♂			♀		
Sample No	1	2	3	46	47	48	16	18	19	61	62	31	32	33	75	76	77

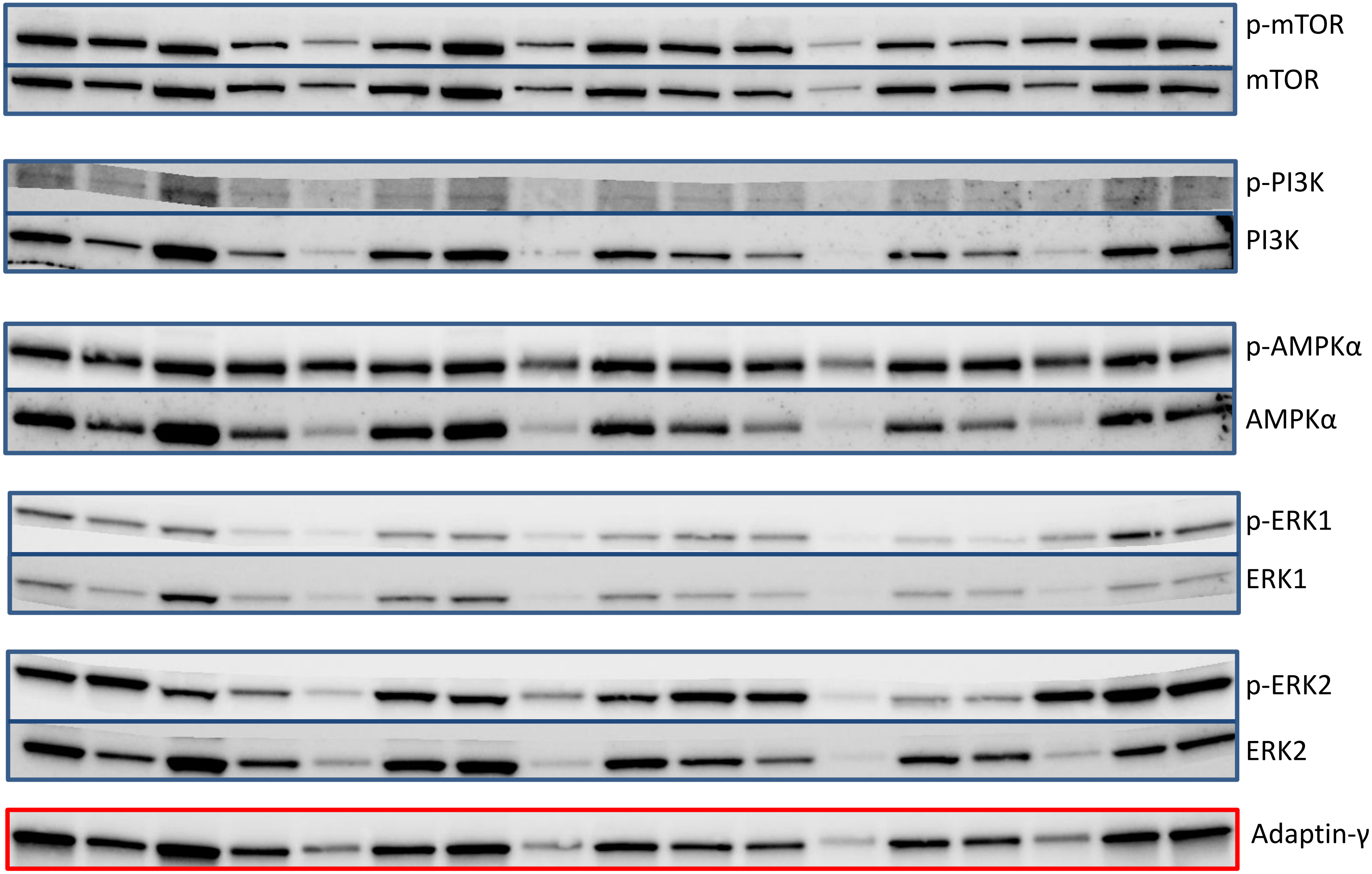

Gel 2

WT						WT/KO					KO						Genotype
♂			♀			♂		♀			♂			♀			Sex
4	5	6	49	50	51	20	21	63	64	65	34	35	36	78	79	80	Sample No

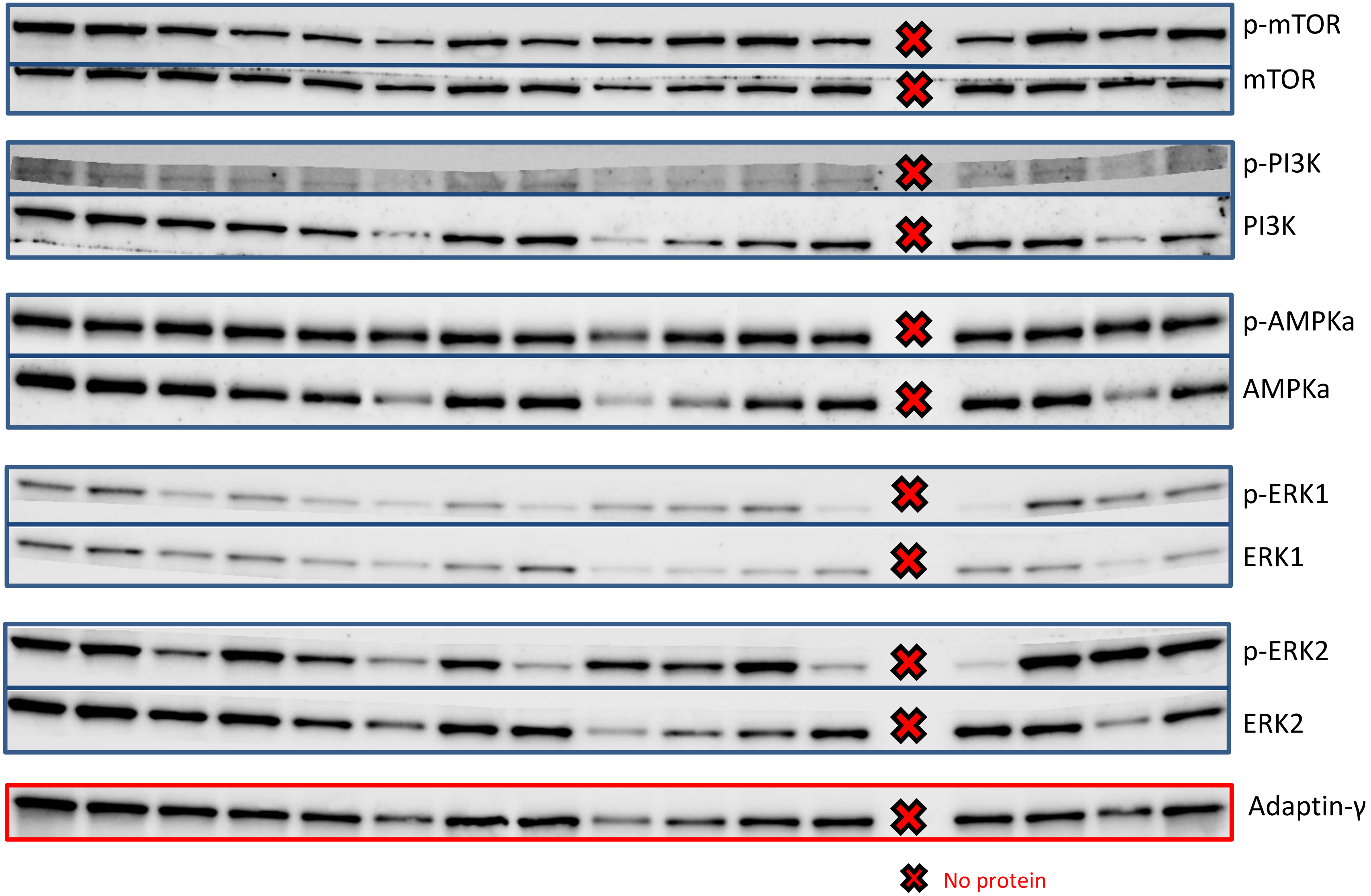

mTOR (≈289 kDa)

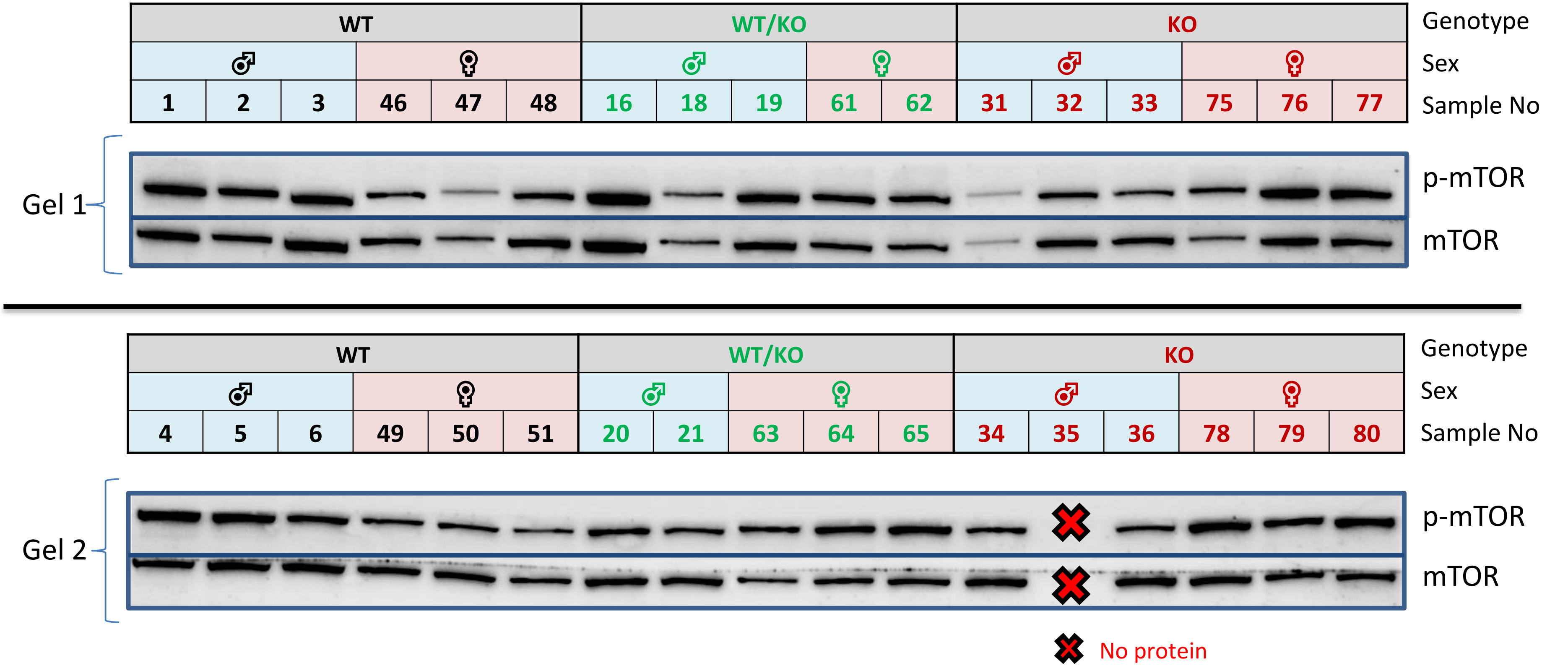

WT
WT/KO
KO
Genotype
Sex
Sample No
1 2 3 46 47 48 16 18 19 61 62 31 32 33 75 76 77
Gel 1
p-mTOR
mTOR
WT
WT/KO
KO
Genotype
Sex
Sample No
4 5 6 49 50 51 20 21 63 64 65 34 35 36 78 79 80
Gel 2
p-mTOR
mTOR
No protein

PI3K (≈85 kDa)

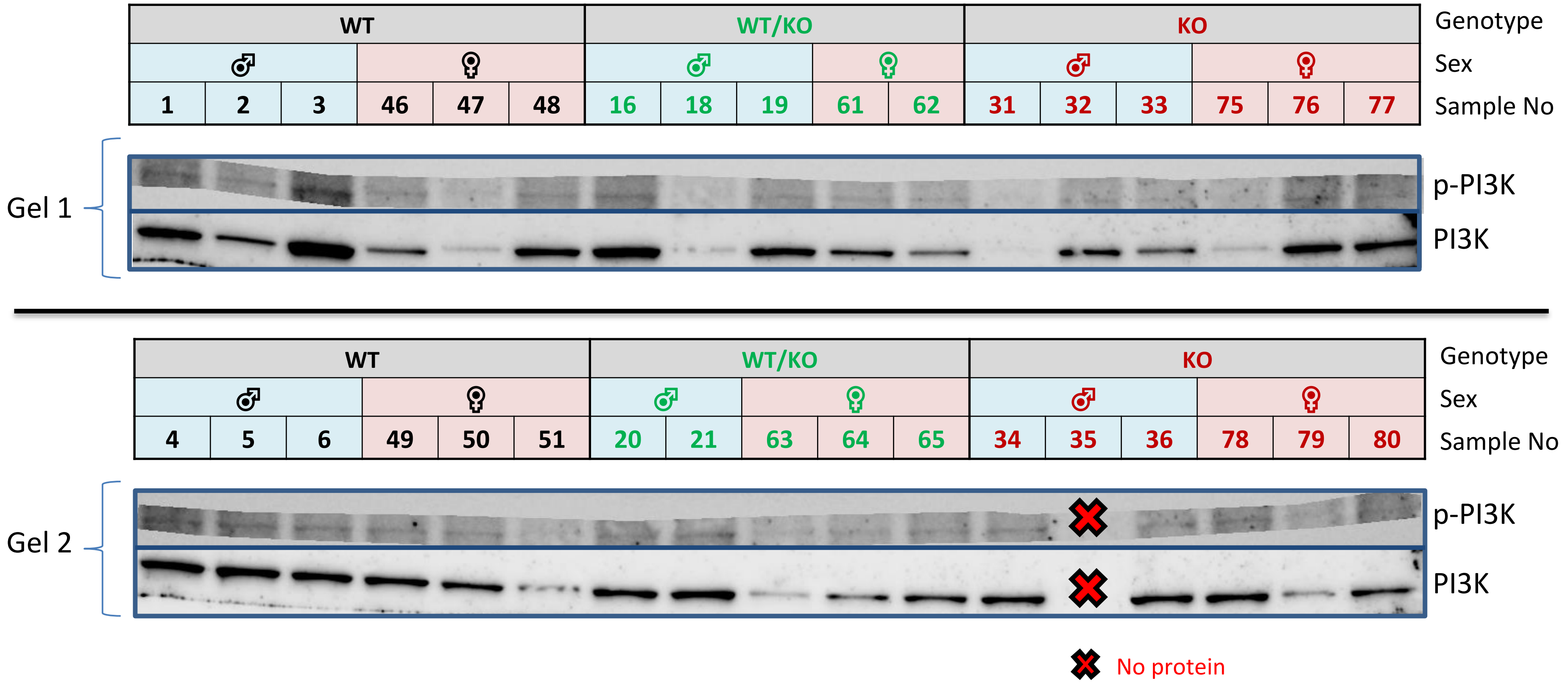

WT
WT/KO
KO
Genotype
Sex
Sample No
1 2 3 46 47 48 16 18 19 61 62 31 32 33 75 76 77
Gel 1
p-PI3K
PI3K
WT
WT/KO
KO
Genotype
Sex
Sample No
4 5 6 49 50 51 20 21 63 64 65 34 35 36 78 79 80
Gel 2
p-PI3K
PI3K
No protein

AMPKα (≈62 kDa)

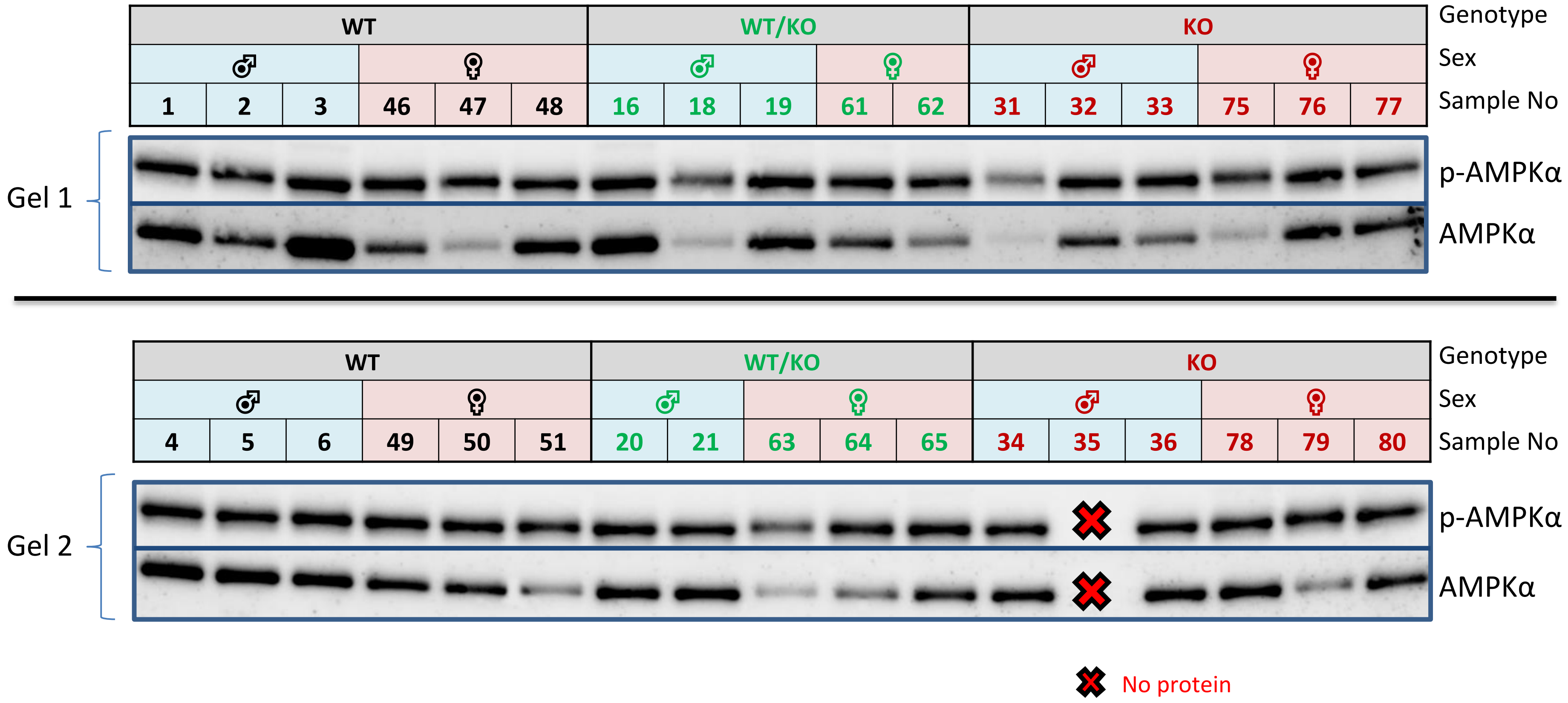

WT
WT/KO
KO
Genotype
Sex
Sample No
1 2 3 46 47 48 16 18 19 61 62 31 32 33 75 76 77
Gel 1
p-AMPKα
AMPKα
WT
WT/KO
KO
Genotype
Sex
Sample No
4 5 6 49 50 51 20 21 63 64 65 34 35 36 78 79 80
Gel 2
p-AMPKα
AMPKα
No protein

ERK1 (≈44 kDa)

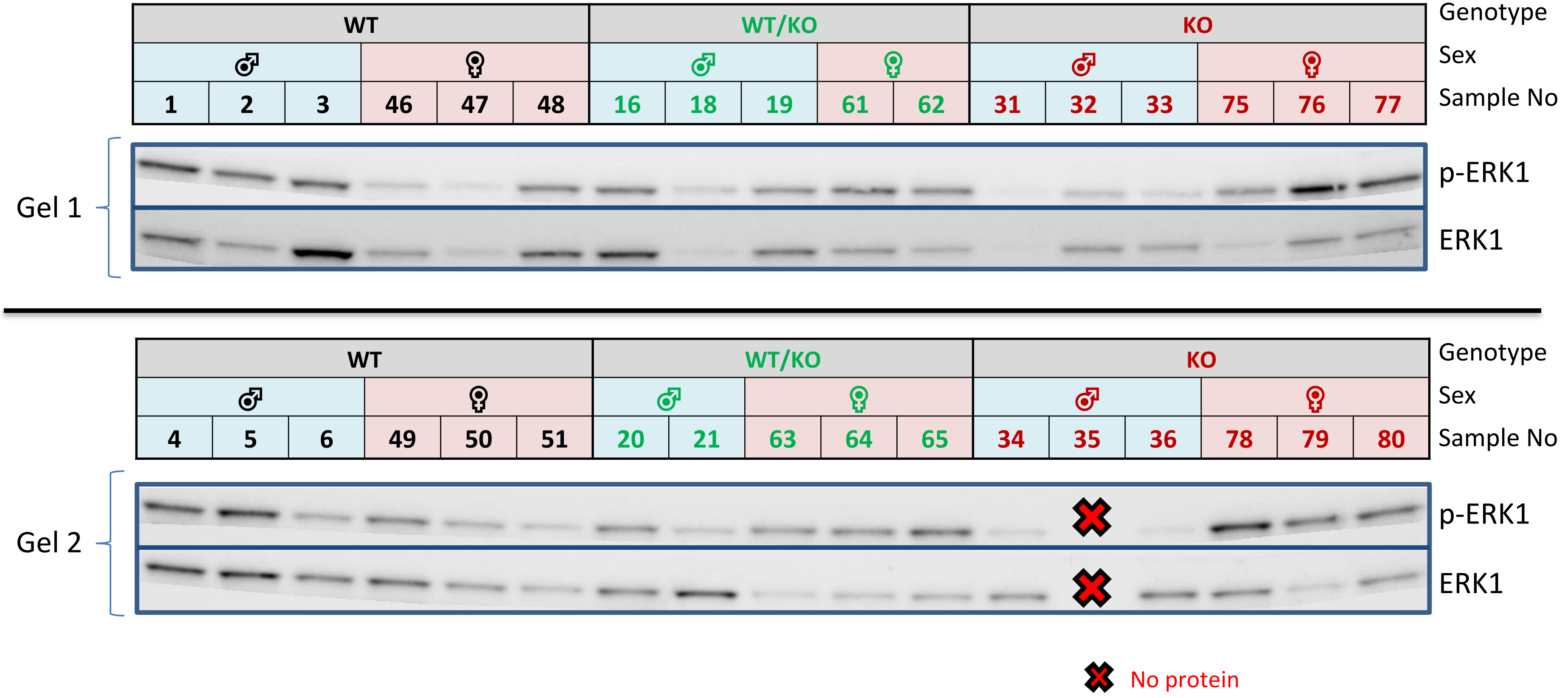
WT
WT/KO
KO
Genotype
Sex
Sample No
1
2
3
46
47
48
16
18
19
61
62
31
32
33
75
76
77
Gel 1
p-ERK1
ERK1
4
5
6
49
50
51
20
21
63
64
65
34
35
36
78
79
80
Gel 2
No protein

ERK2 (≈42 kDa)

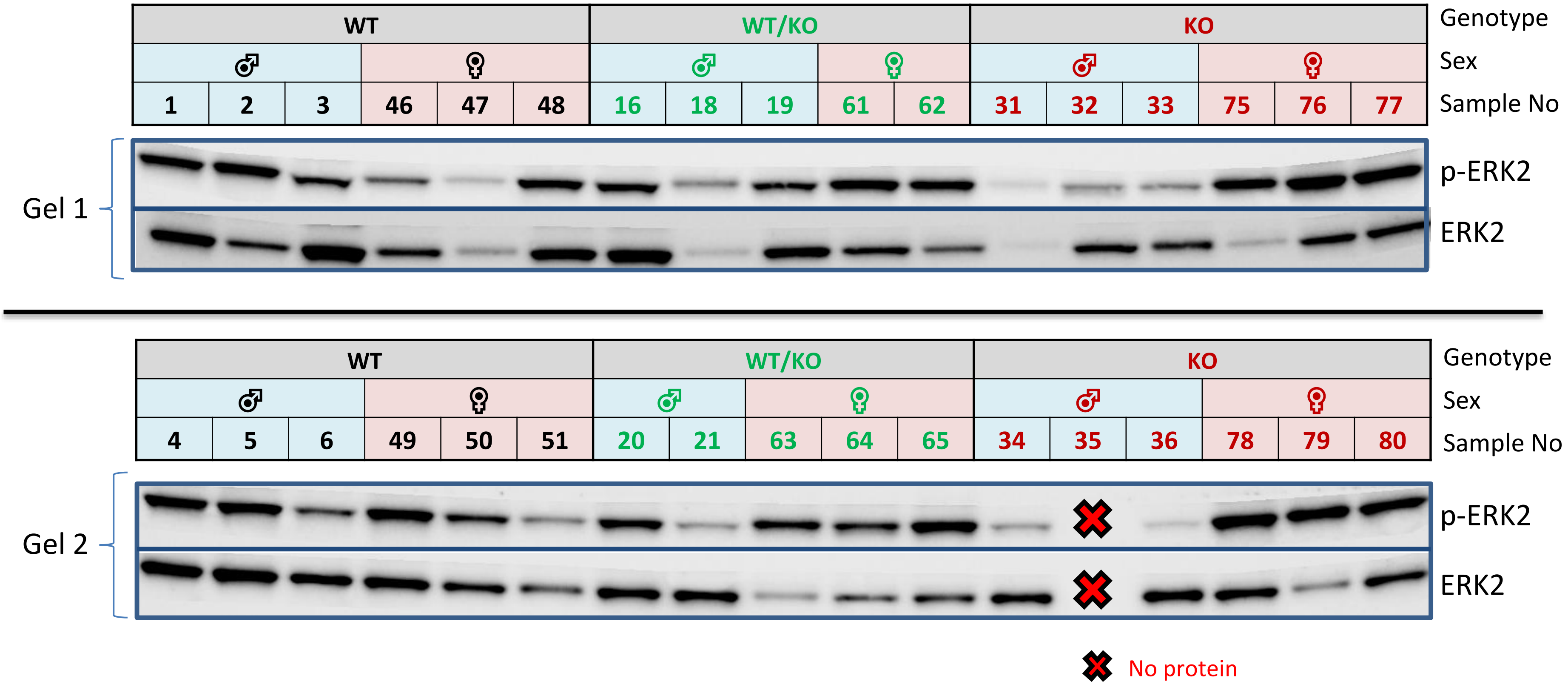
WT
WT/KO
KO
Genotype
Sex
Sample No
1 2 3 46 47 48 16 18 19 61 62 31 32 33 75 76 77
Gel 1
p-ERK2
ERK2
WT
WT/KO
KO
Genotype
Sex
Sample No
4 5 6 49 50 51 20 21 63 64 65 34 35 36 78 79 80
Gel 2
p-ERK2
ERK2
No protein

2º batch

(19/11/2021)

Gel 1

WT						WT/KO				KO						WT/KO	Genotype
♂			♀			♂		♀		♂			♀			♂	Sex
1	2	3	46	52	48	16	22	61	62	31	32	33	81	76	77	19	Sample No

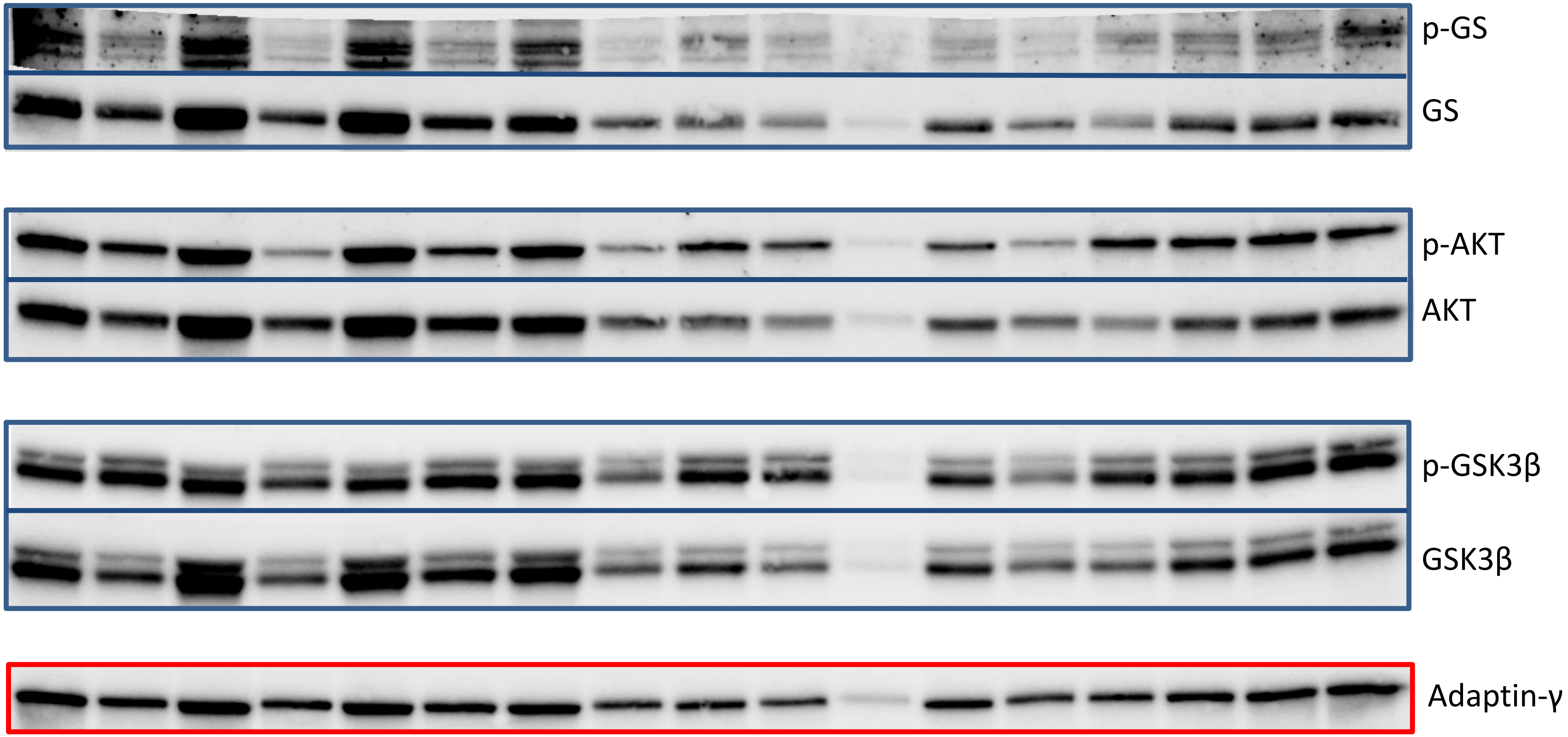

Gel 2

Genotype	WT						WT/KO					KO					
Sex	♂			♀			♂		♀			♂			♀		
Sample No	4	5	6	49	50	51	20	21	66	64	65	34	37	36	78	79	80

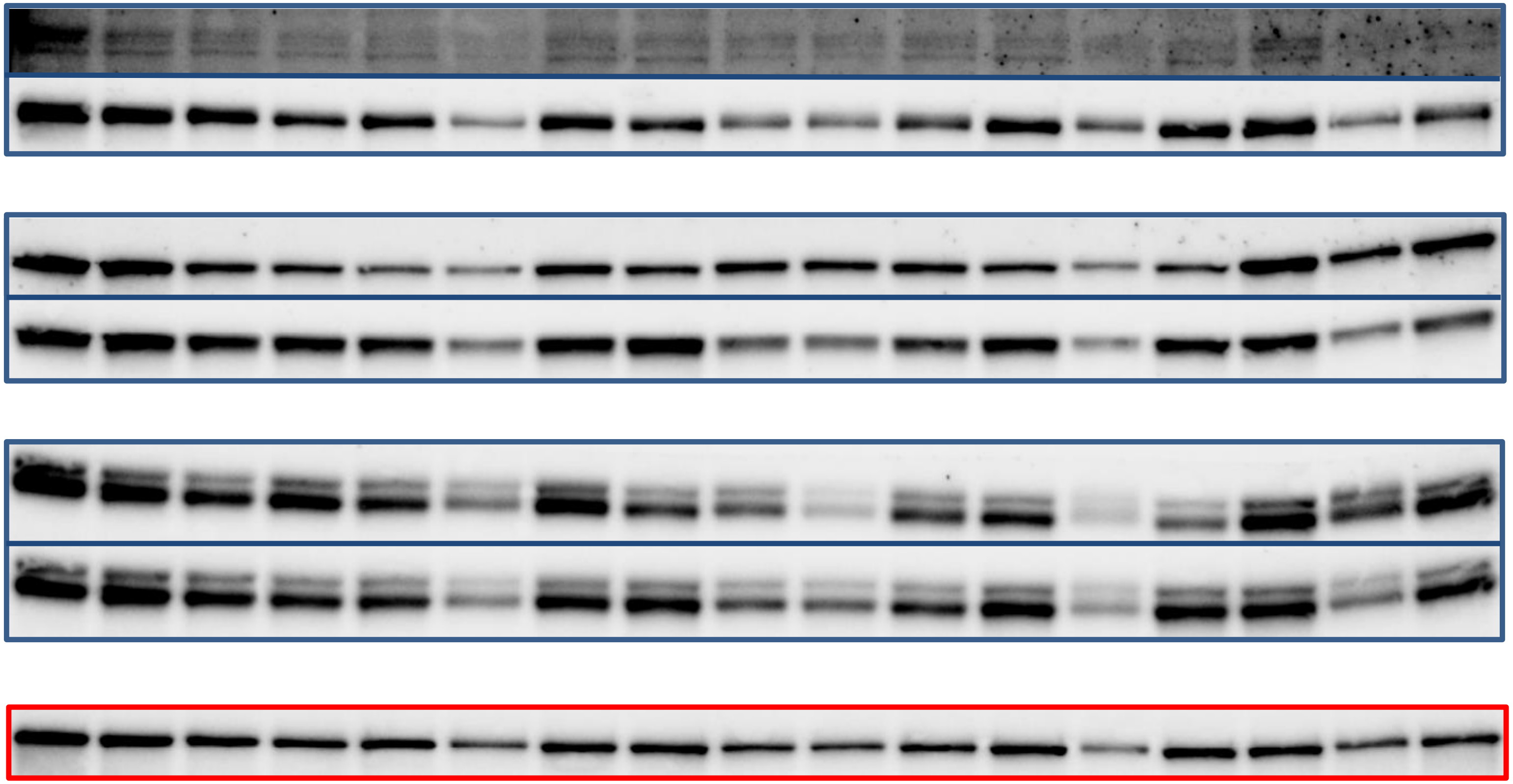

GS (≈84 kDa)

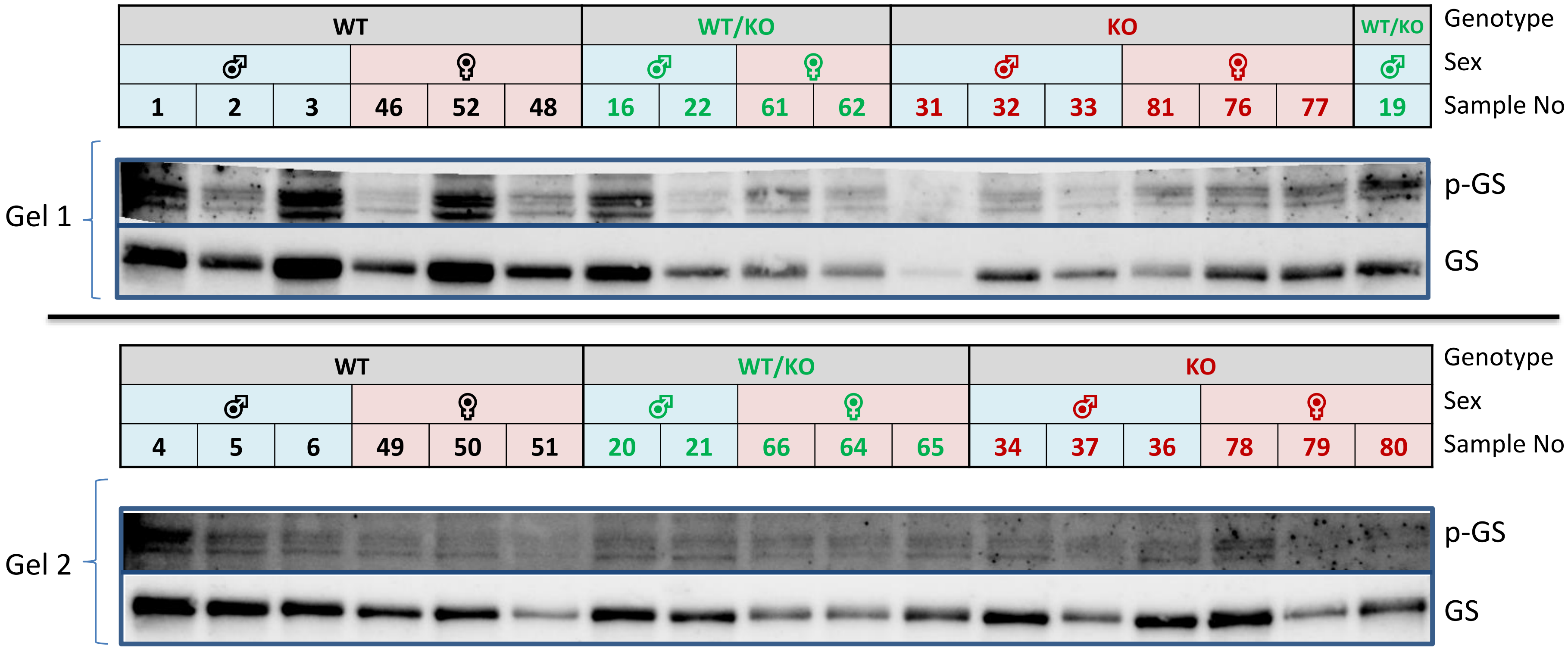
WT
WT/KO
KO
WT/KO
Genotype
Sex
1
2
3
46
52
48
16
22
61
62
31
32
33
81
76
77
19
Sample No
Gel 1
p-GS
GS
WT
WT/KO
KO
Genotype
Sex
4
5
6
49
50
51
20
21
66
64
65
34
37
36
78
79
80
Sample No
Gel 2
p-GS
GS

AKT (≈60 kDa)

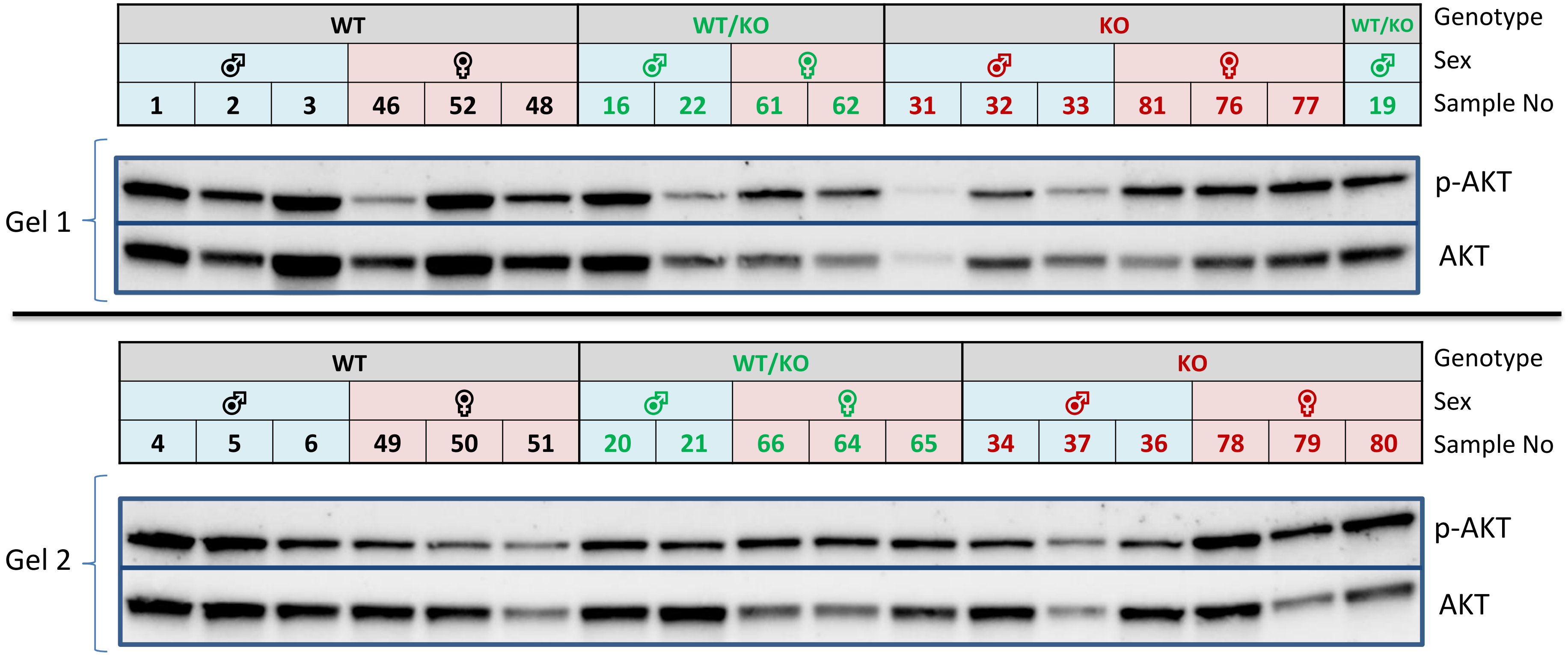

Gel 1
Genotype: WT | WT/KO | KO | WT/KO
Sex
Sample No: 1 2 3 46 52 48 16 22 61 62 31 32 33 81 76 77 19
p-AKT
AKT
Gel 2
Genotype: WT | WT/KO | KO
Sex
Sample No: 4 5 6 49 50 51 20 21 66 64 65 34 37 36 78 79 80
p-AKT
AKT

GSK3β (≈46 kDa)

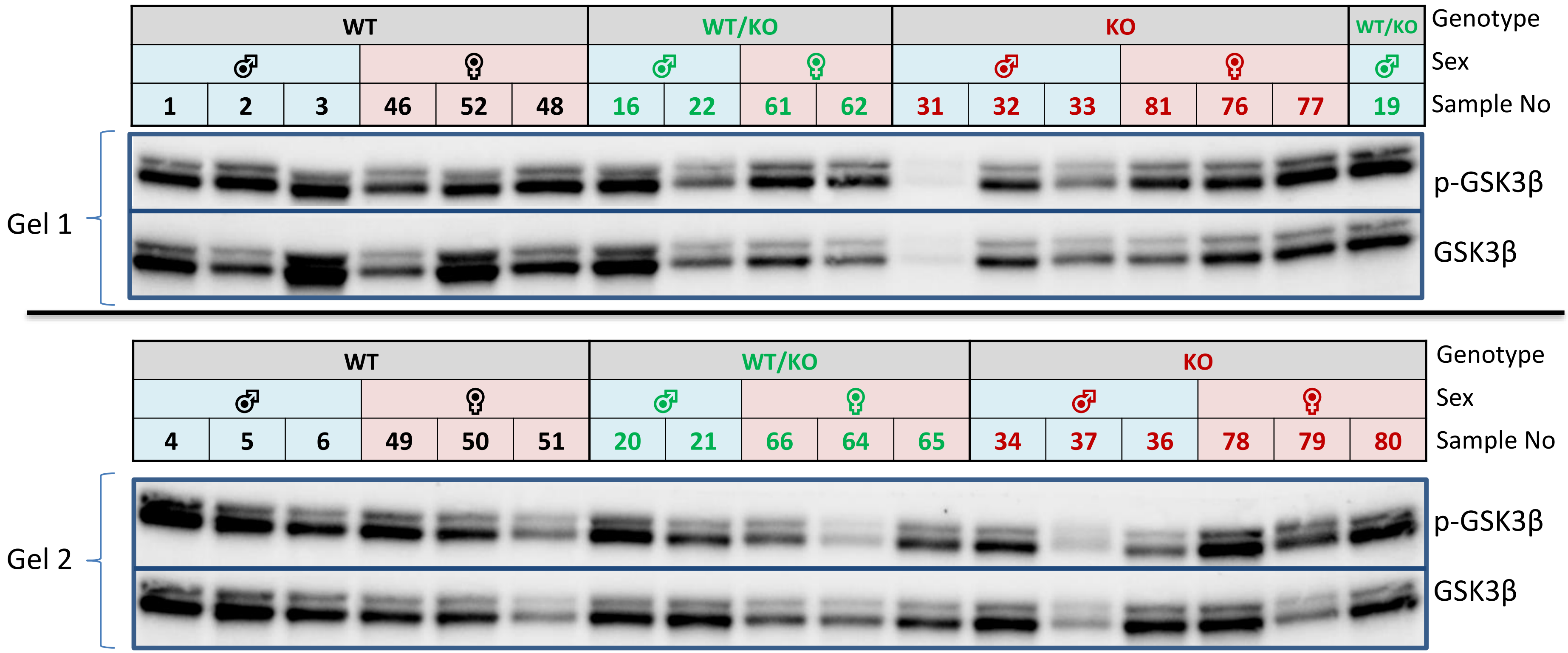
Genotype
Sex
Sample No
WT
WT/KO
KO
WT/KO
1
2
3
46
52
48
16
22
61
62
31
32
33
81
76
77
19
p-GSK3β
GSK3β
Gel 1
Genotype
Sex
Sample No
WT
WT/KO
KO
4
5
6
49
50
51
20
21
66
64
65
34
37
36
78
79
80
p-GSK3β
GSK3β
Gel 2
