# Supplementary material for: Sex-based differences in growth-related IGF1 signaling in response to PAPP-A2 deficiency: comparative effects of rhGH, rhIGF1 and rhPAPP-A2 treatments
Source: Biol Sex Differ. 2024 Apr 8;15:34. doi: 10.1186/s13293-024-00603-5 (PMC11000399; doi:10.1186/s13293-024-00603-5)
Supplement: Supplementary file 12 — Supplementary Material 12 [file 13293_2024_603_MOESM12_ESM.pdf]

Supplementary Figure S2.
Western Blot results in Pituitary Gland

- **WT ♂/♀**
- **KO ♂/♀**

Two batches of 2 gels each:

1st batch: 05-03-22

2º batch: 19-04-22

1st batch

(05-03-22)

Gel 1

WT							KO					Genotype
♂			♀				♂			♀		Sex
13	14	20	15	16	17	18	1	2	3	9	10	Sample No

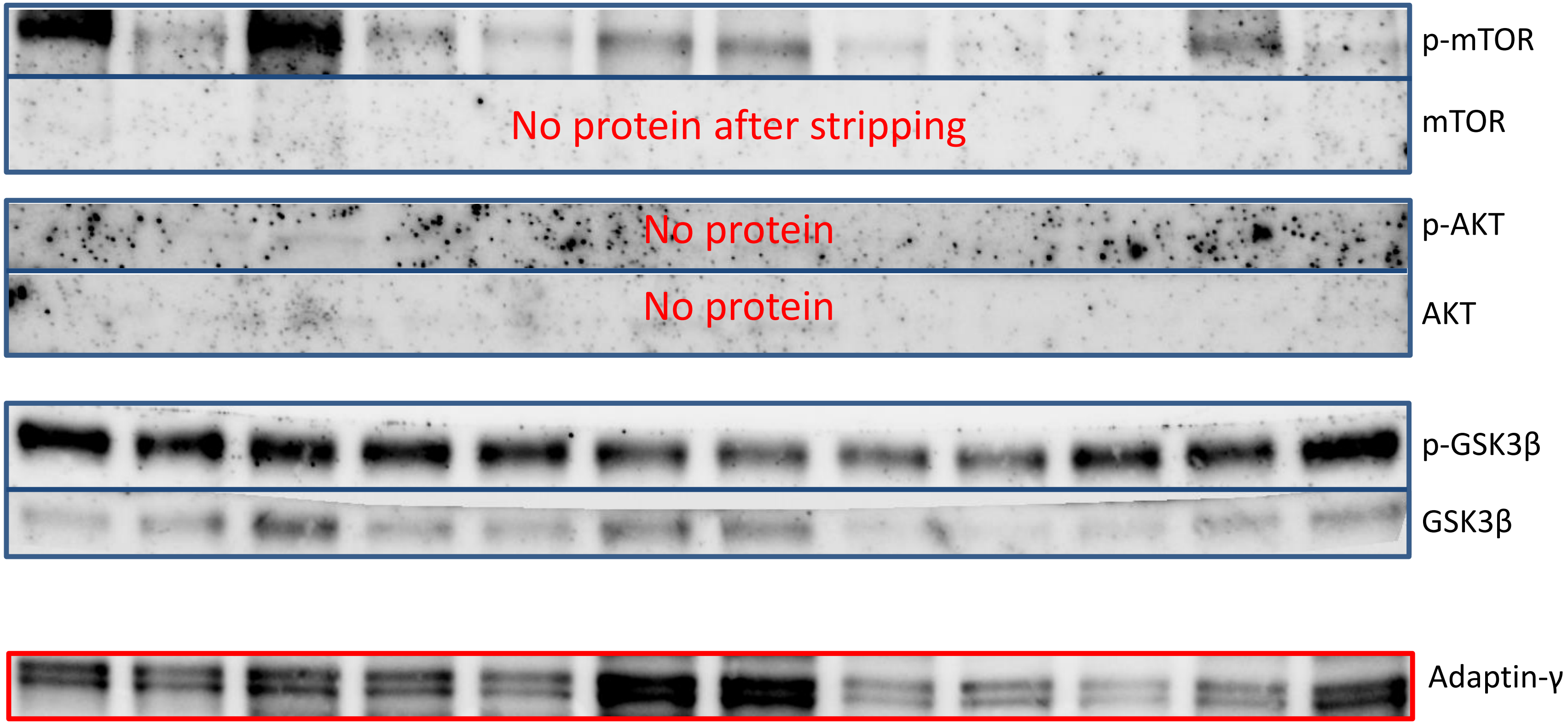

Gel 2

WT						KO						Genotype
♂			♀			♂				♀		Sex
27	28	29	21	22	24	4	5	7	8	25	26	Sample No

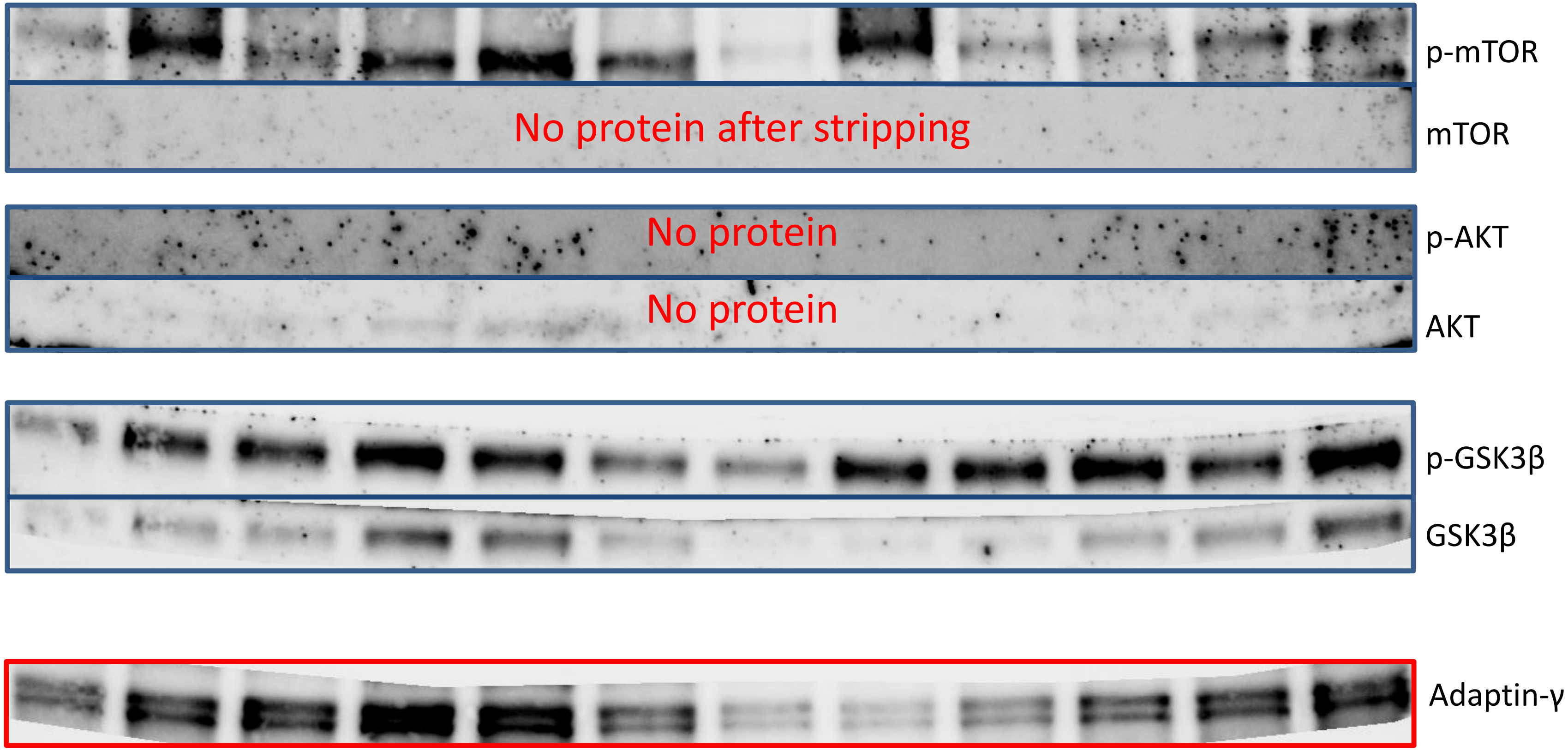

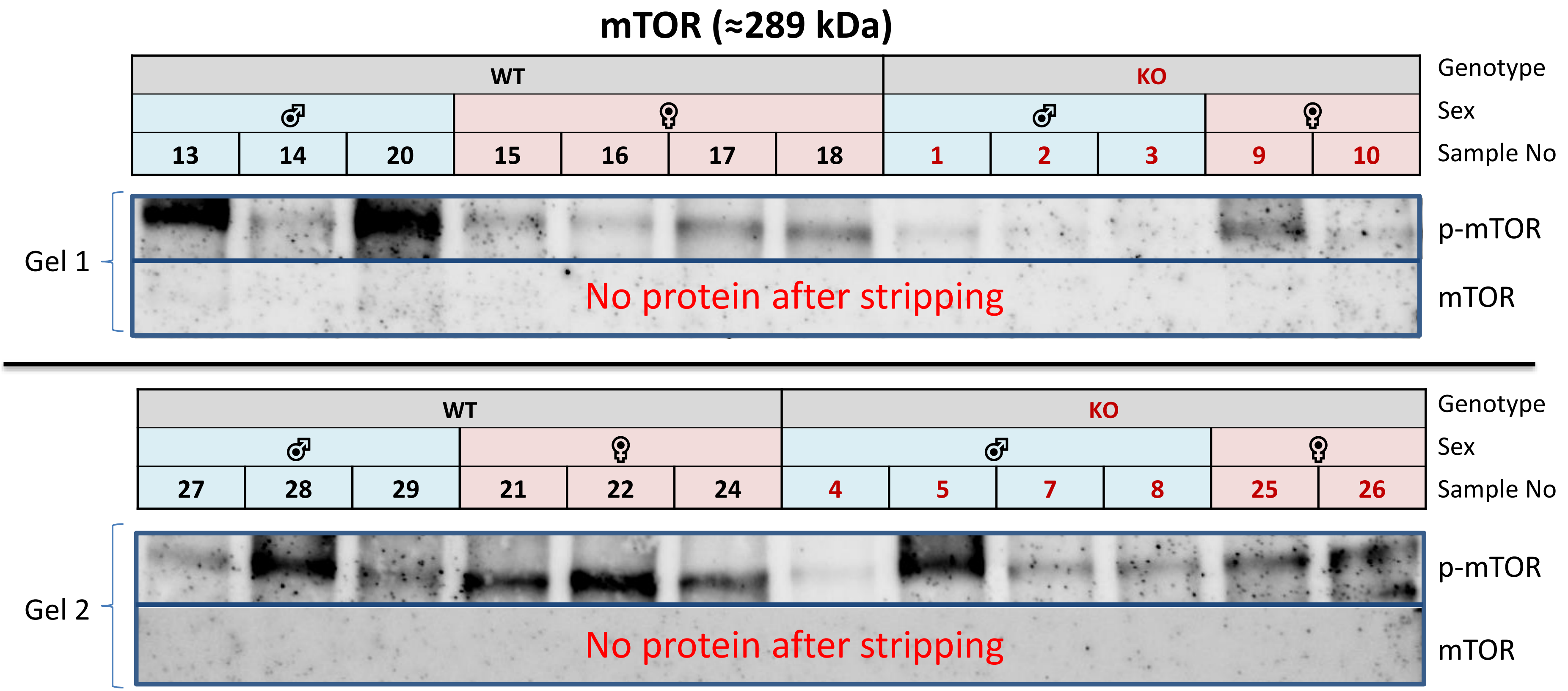
mTOR (≈289 kDa)
Genotype
Sex
Sample No
WT
KO
♂
♀
13
14
20
15
16
17
18
1
2
3
9
10
Gel 1
p-mTOR
mTOR
No protein after stripping
WT
KO
27
28
29
21
22
24
4
5
7
8
25
26
Gel 2
p-mTOR
mTOR
No protein after stripping

AKT (≈60 kDa)

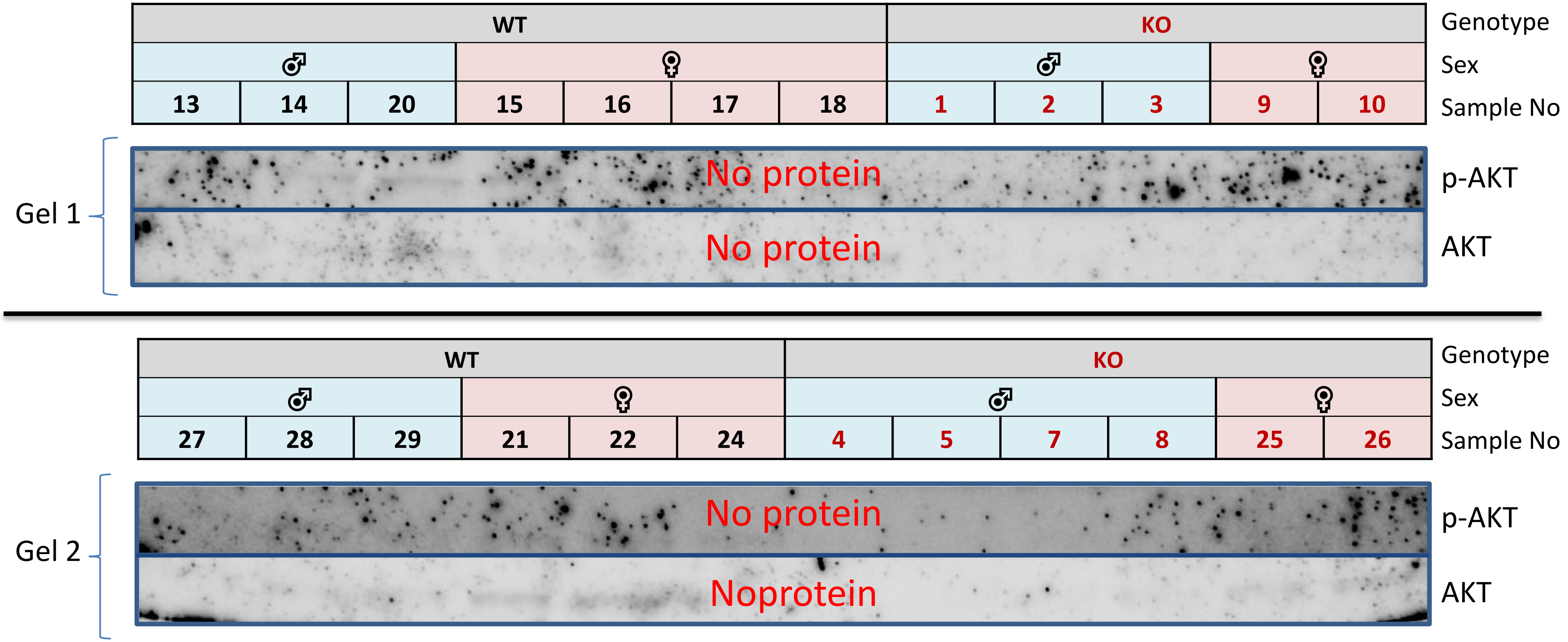
WT
KO
Genotype
♂
♀
♂
♀
Sex
13
14
20
15
16
17
18
1
2
3
9
10
Sample No
Gel 1
No protein
p-AKT
No protein
AKT
WT
KO
Genotype
♂
♀
♂
♀
Sex
27
28
29
21
22
24
4
5
7
8
25
26
Sample No
Gel 2
No protein
p-AKT
Noprotein
AKT

GSK3β (≈46 kDa)

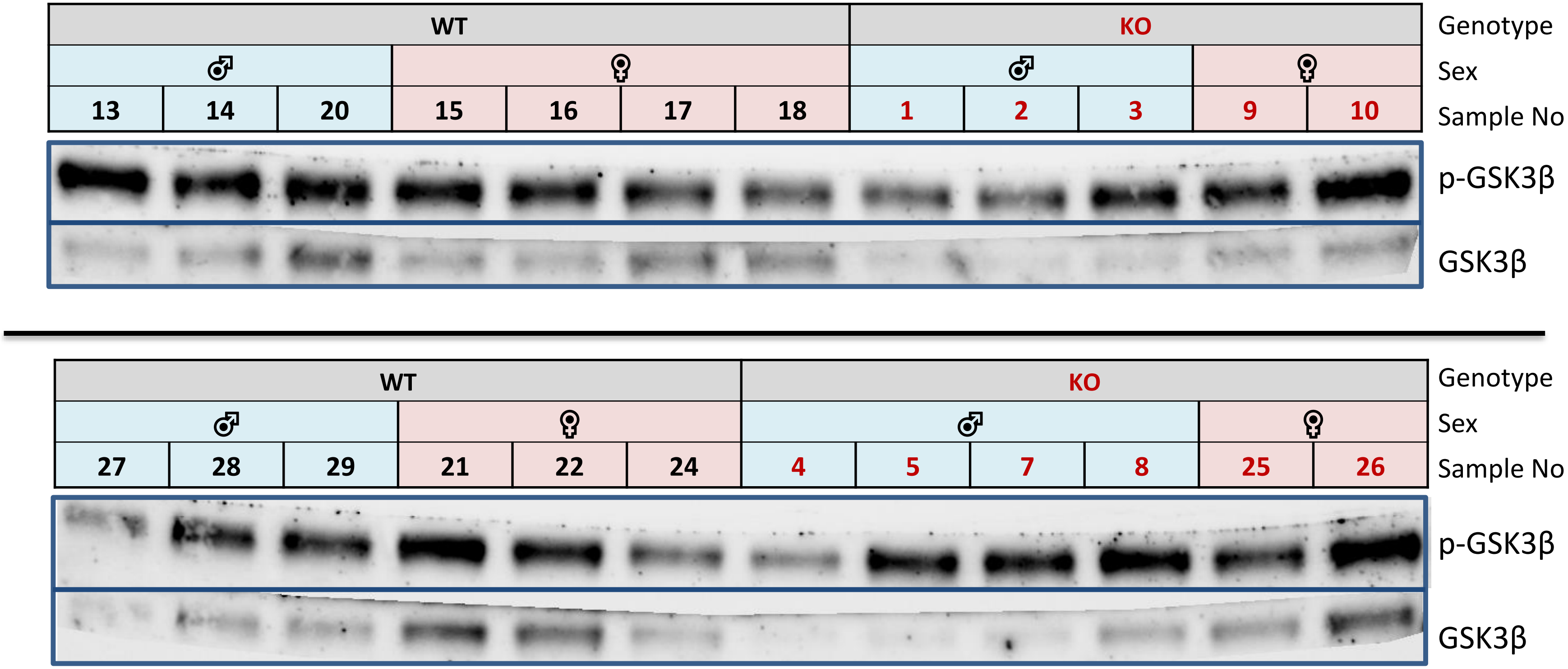
WT
KO
Genotype
Sex
Sample No
13
14
20
15
16
17
18
1
2
3
9
10
p-GSK3β
GSK3β
WT
KO
Genotype
Sex
Sample No
27
28
29
21
22
24
4
5
7
8
25
26
p-GSK3β
GSK3β

2º Batch

(19/04/2022)

Gel 1

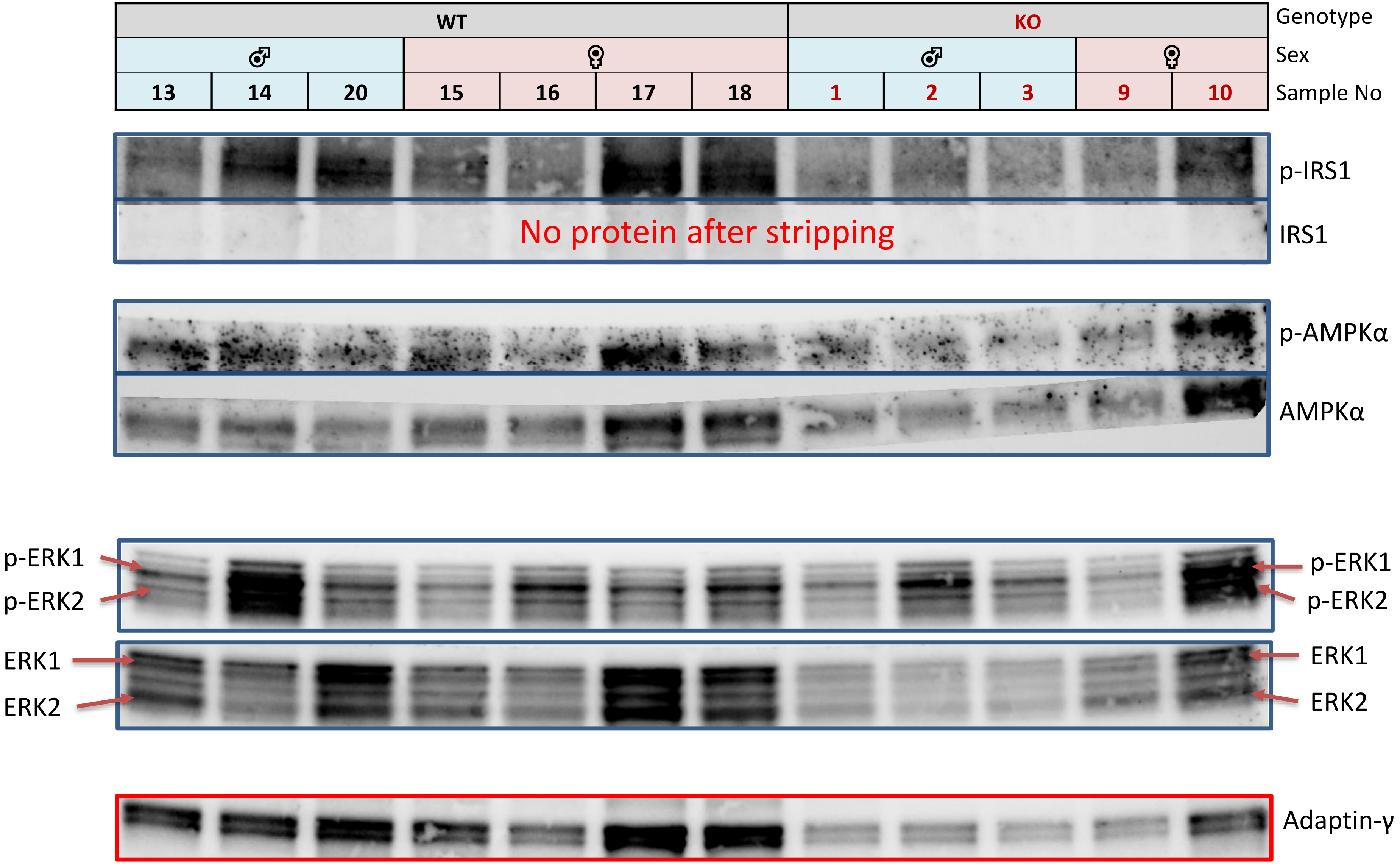

WT
KO
Genotype
♂
♀
♂
♀
Sex
13
14
20
15
16
17
18
1
2
3
9
10
Sample No
p-IRS1
No protein after stripping
IRS1
p-AMPKα
AMPKα
p-ERK1
p-ERK2
p-ERK1
p-ERK2
ERK1
ERK2
ERK1
ERK2
Adaptin-γ

Gel 2

WT						KO						Genotype
♂			♀			♂				♀		Sex
27	28	29	21	22	24	4	5	7	8	25	26	Sample No

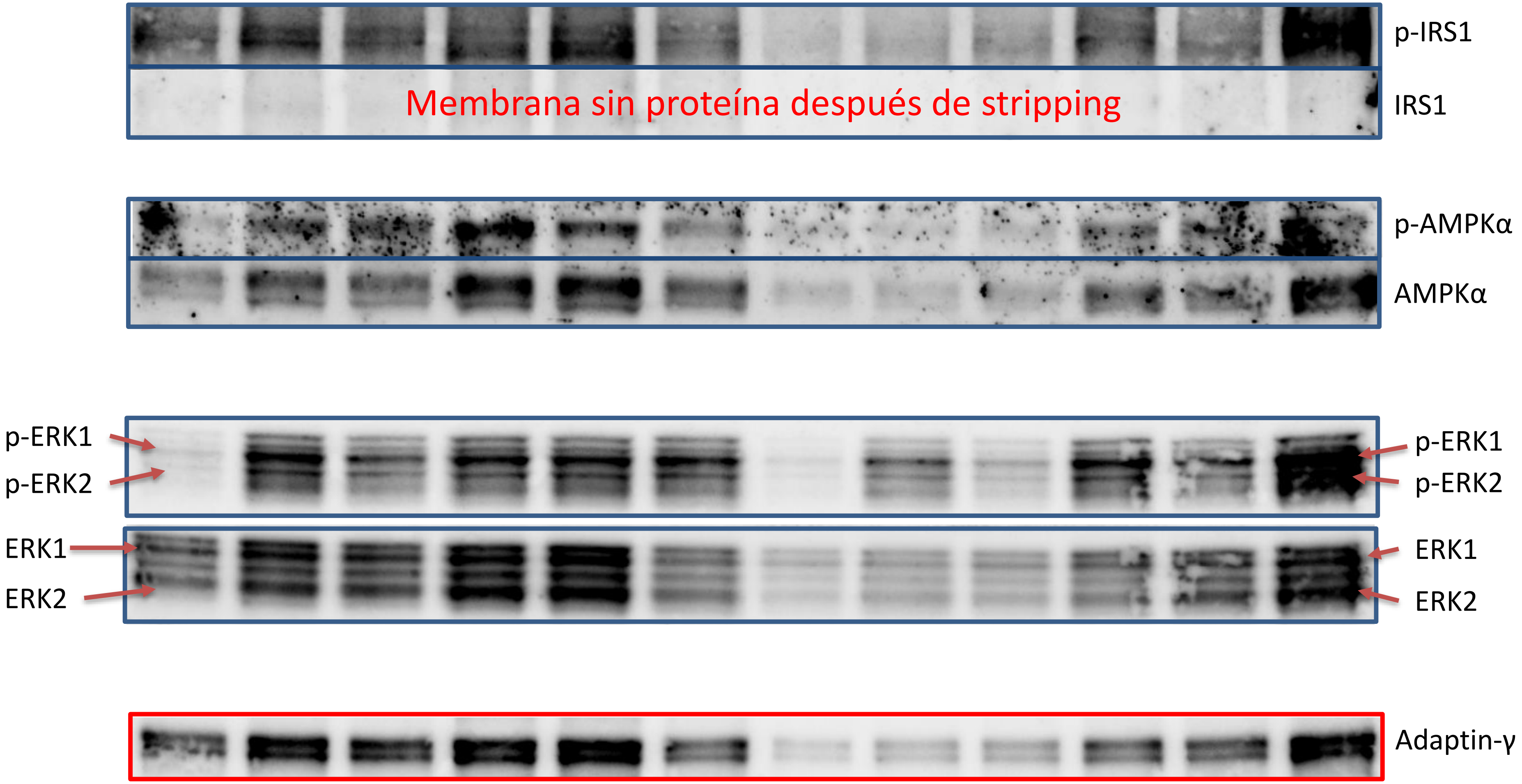

IRS1 (≈180 Kda)

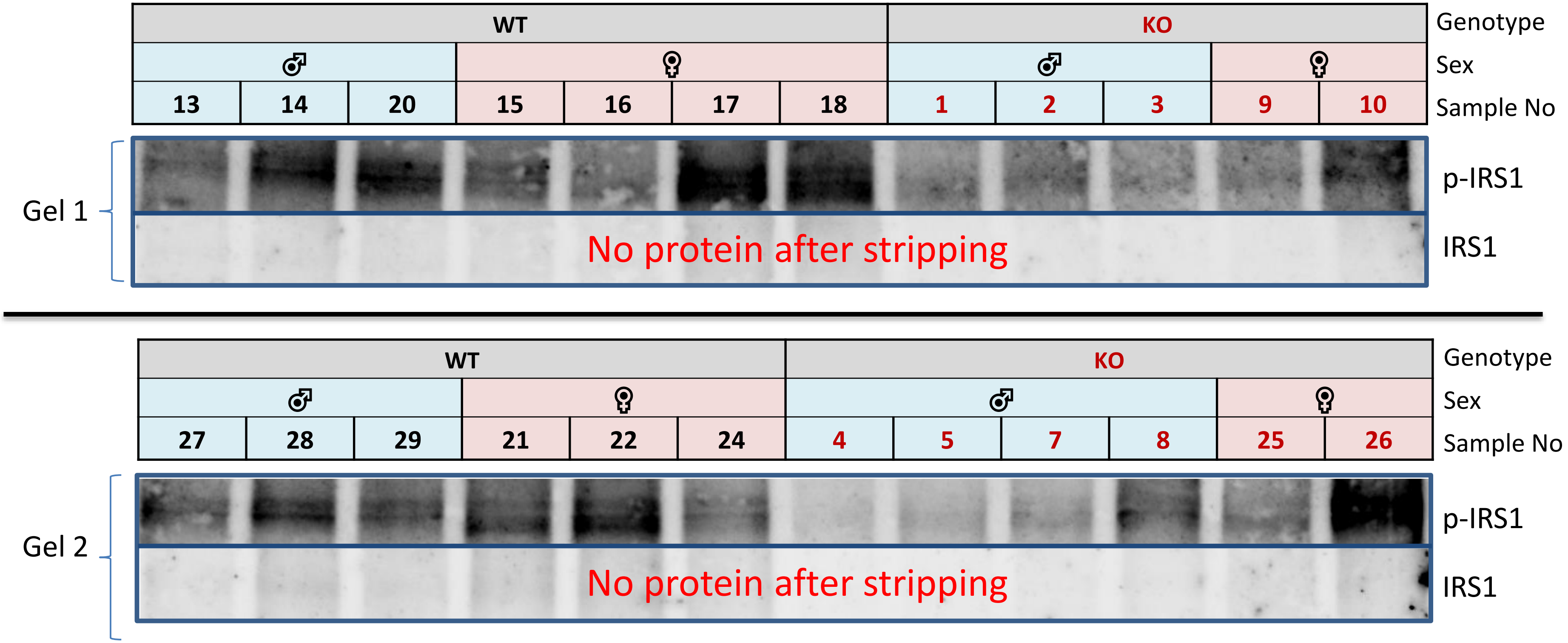
WT
KO
Genotype
Sex
Sample No
13
14
20
15
16
17
18
1
2
3
9
10
Gel 1
p-IRS1
IRS1
No protein after stripping
27
28
29
21
22
24
4
5
7
8
25
26
Gel 2
No protein after stripping

AMPKα (≈62 kDa)

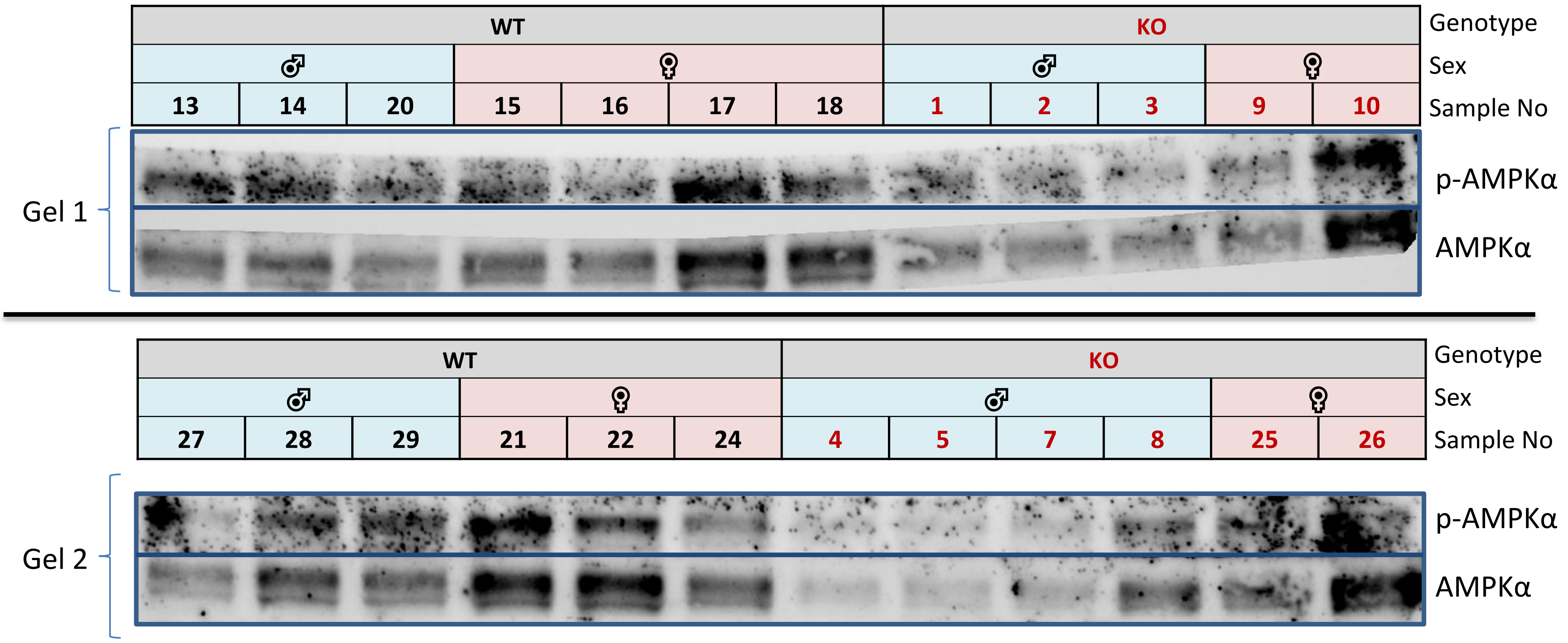
WT
KO
Genotype
Sex
Sample No
13
14
20
15
16
17
18
1
2
3
9
10
Gel 1
p-AMPKα
AMPKα
WT
KO
Genotype
Sex
Sample No
27
28
29
21
22
24
4
5
7
8
25
26
Gel 2
p-AMPKα
AMPKα

ERK1 (≈44 kDa)

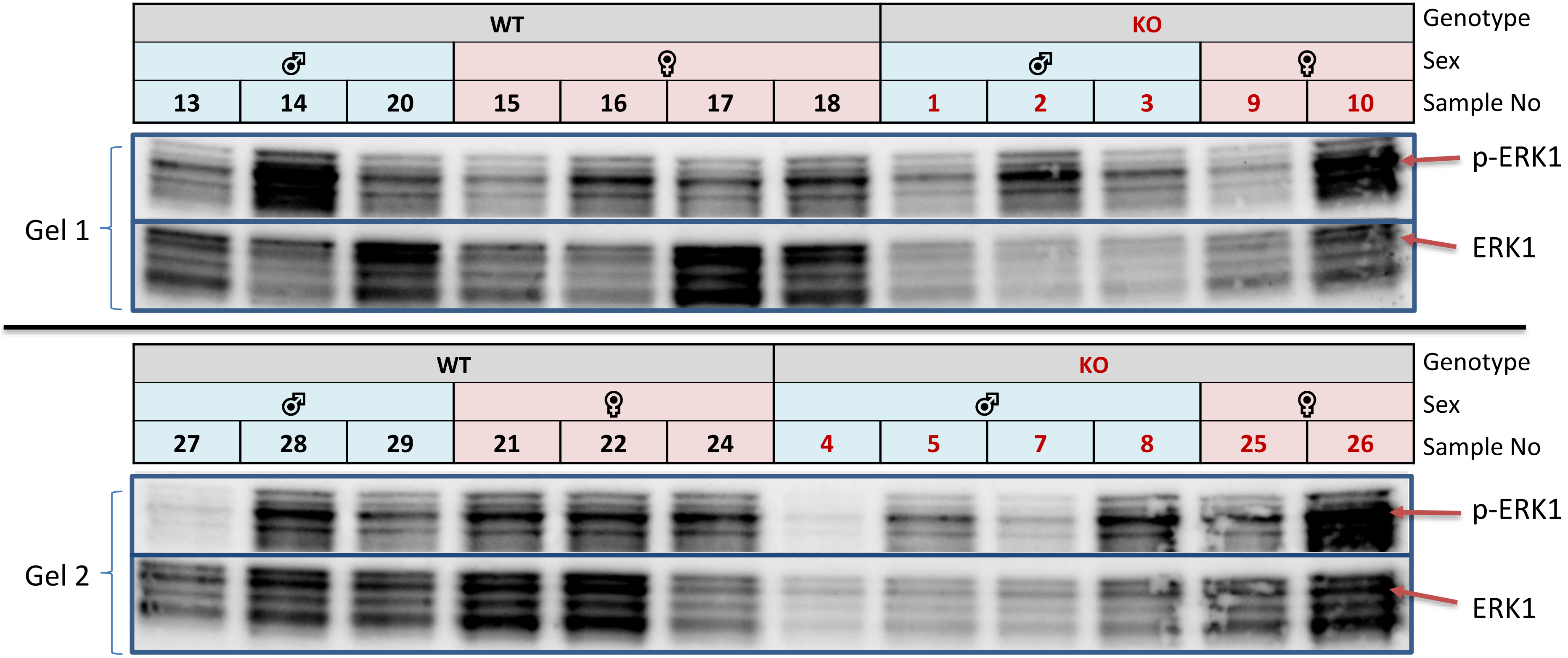
WT
KO
Genotype
♂
♀
Sex
13
14
20
15
16
17
18
1
2
3
9
10
Sample No
Gel 1
p-ERK1
ERK1
WT
KO
Genotype
♂
♀
Sex
27
28
29
21
22
24
4
5
7
8
25
26
Sample No
Gel 2
p-ERK1
ERK1

ERK2 (≈42 kDa)

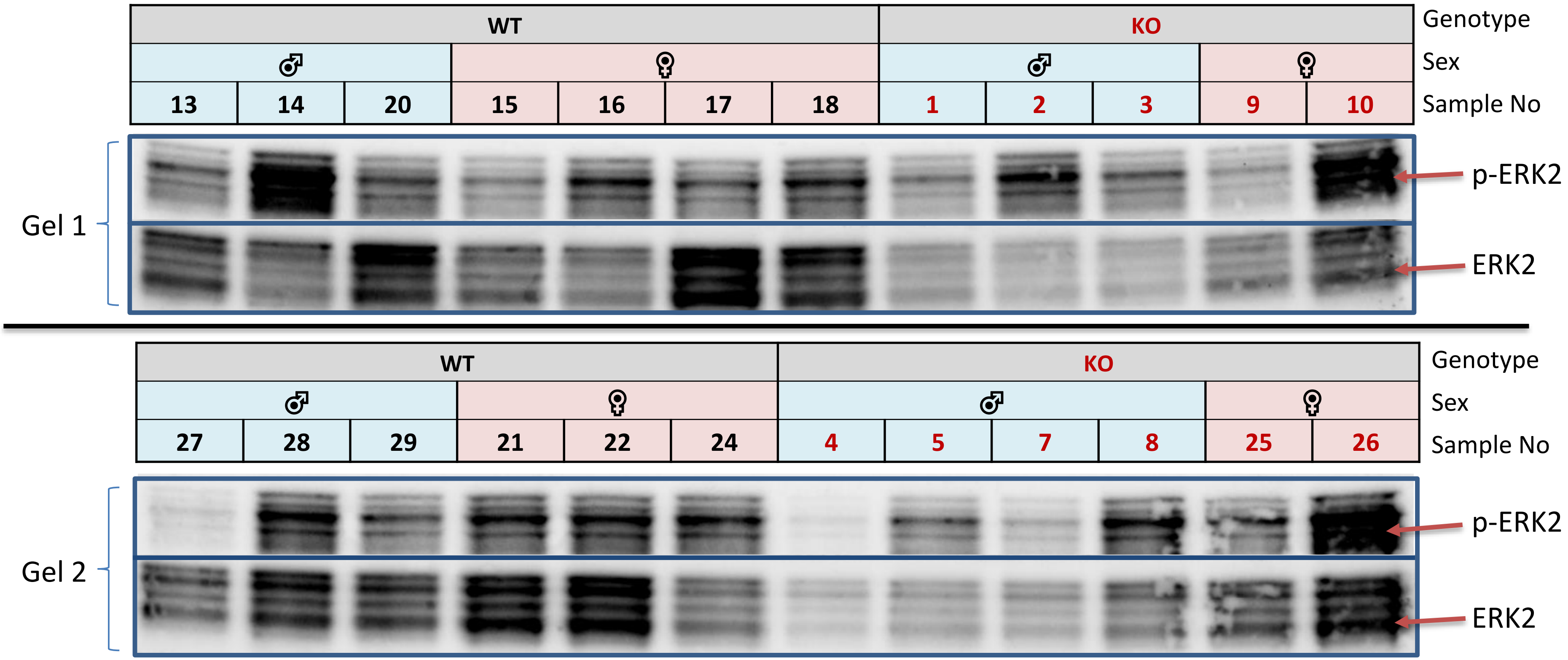
WT
KO
Genotype
Sex
13
14
20
15
16
17
18
1
2
3
9
10
Sample No
Gel 1
p-ERK2
ERK2
WT
KO
Genotype
Sex
27
28
29
21
22
24
4
5
7
8
25
26
Sample No
Gel 2
p-ERK2
ERK2

Figure 7E

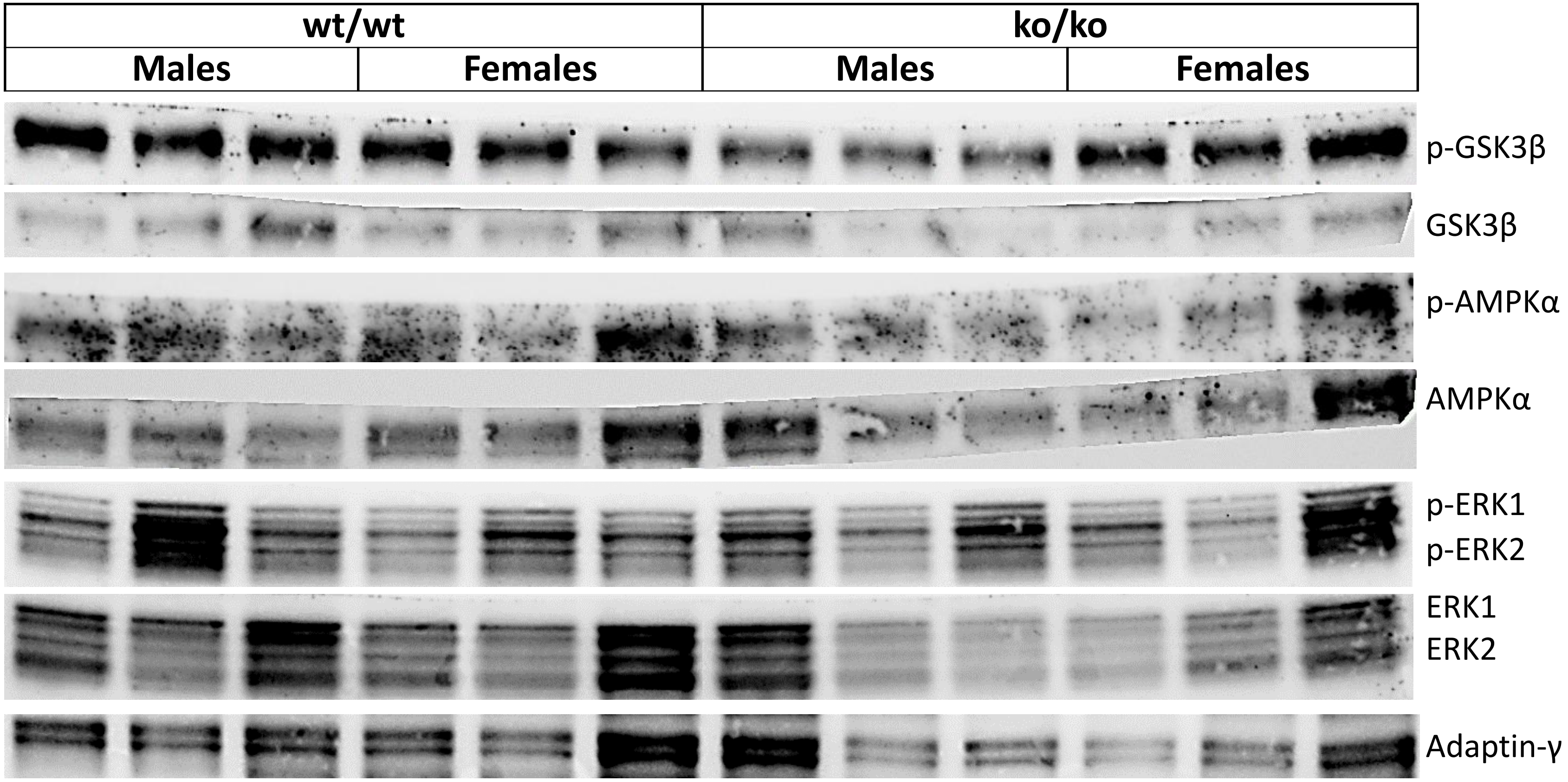
wt/wt
ko/ko
Males
Females
Males
Females
p-GSK3β
GSK3β
p-AMPKα
AMPKα
p-ERK1
p-ERK2
ERK1
ERK2
Adaptin-γ
