# Supplementary material for: Sex-based differences in growth-related IGF1 signaling in response to PAPP-A2 deficiency: comparative effects of rhGH, rhIGF1 and rhPAPP-A2 treatments
Source: Biol Sex Differ. 2024 Apr 8;15:34. doi: 10.1186/s13293-024-00603-5 (PMC11000399; doi:10.1186/s13293-024-00603-5)
Supplement: Supplementary file 13 — Supplementary Material 13 [file 13293_2024_603_MOESM13_ESM.pdf]

Supplementary Figure S3.
Western Blot results in Liver

- **WT ♂/♀**
- **KO ♂/♀**

Two batches of 2 gels each:

1er batch: 26-10-21

2º batch: 3-12-21

1er batch

(26/10/2021)

Gel 1

WT					WT/KO						KO						Genotype
♂			♀		♂			♀			♂			♀			Sex
1	2	3	46	52	16	17	18	62	66	67	31	32	33	75	76	77	Sample No

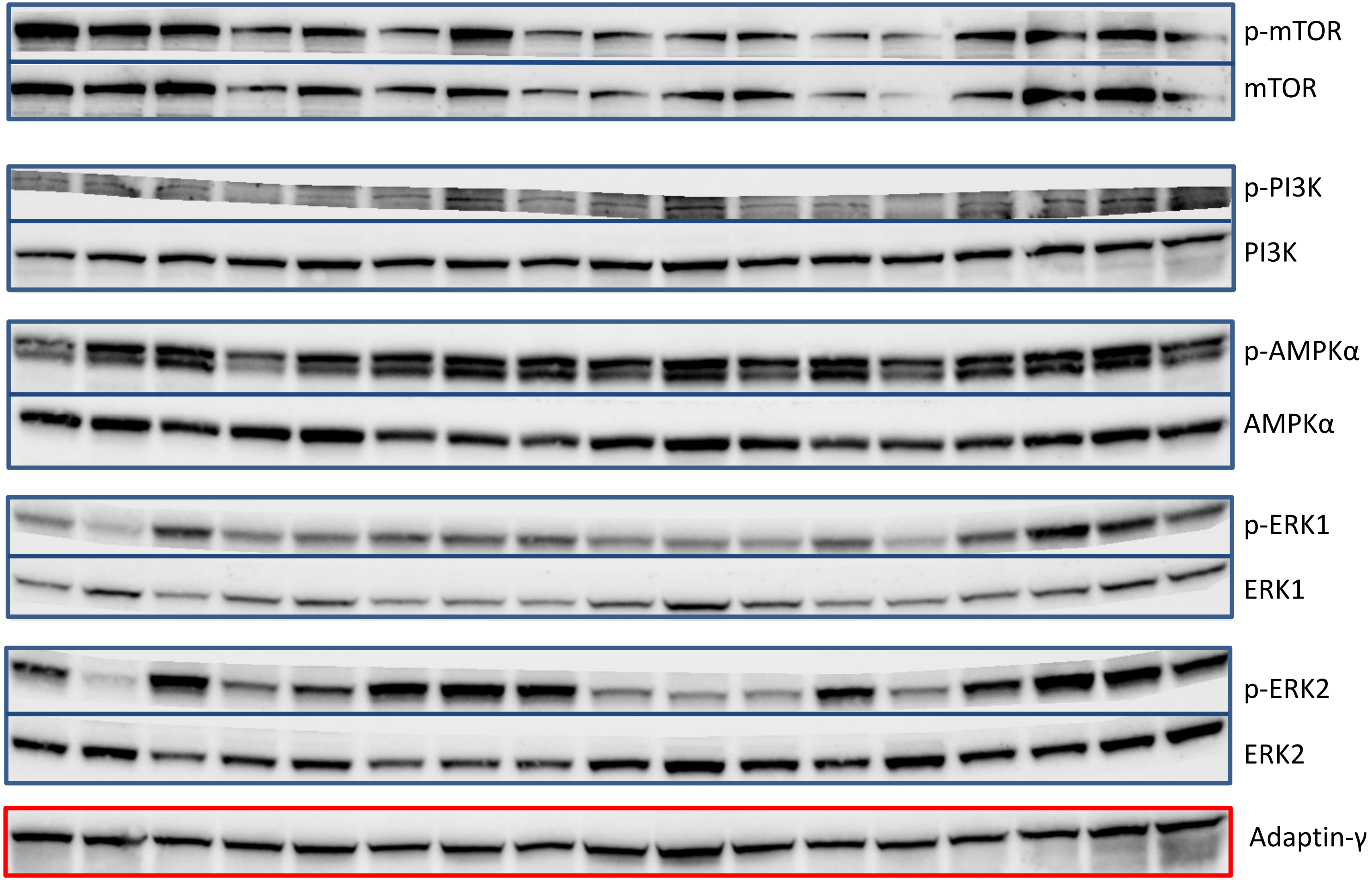

Gel 2

Genotype	WT						WT/KO					KO					
Sex	♂			♀			♂		♀			♂			♀		
Sample No	4	5	6	49	50	51	19	20	63	64	65	34	35	36	78	79	80

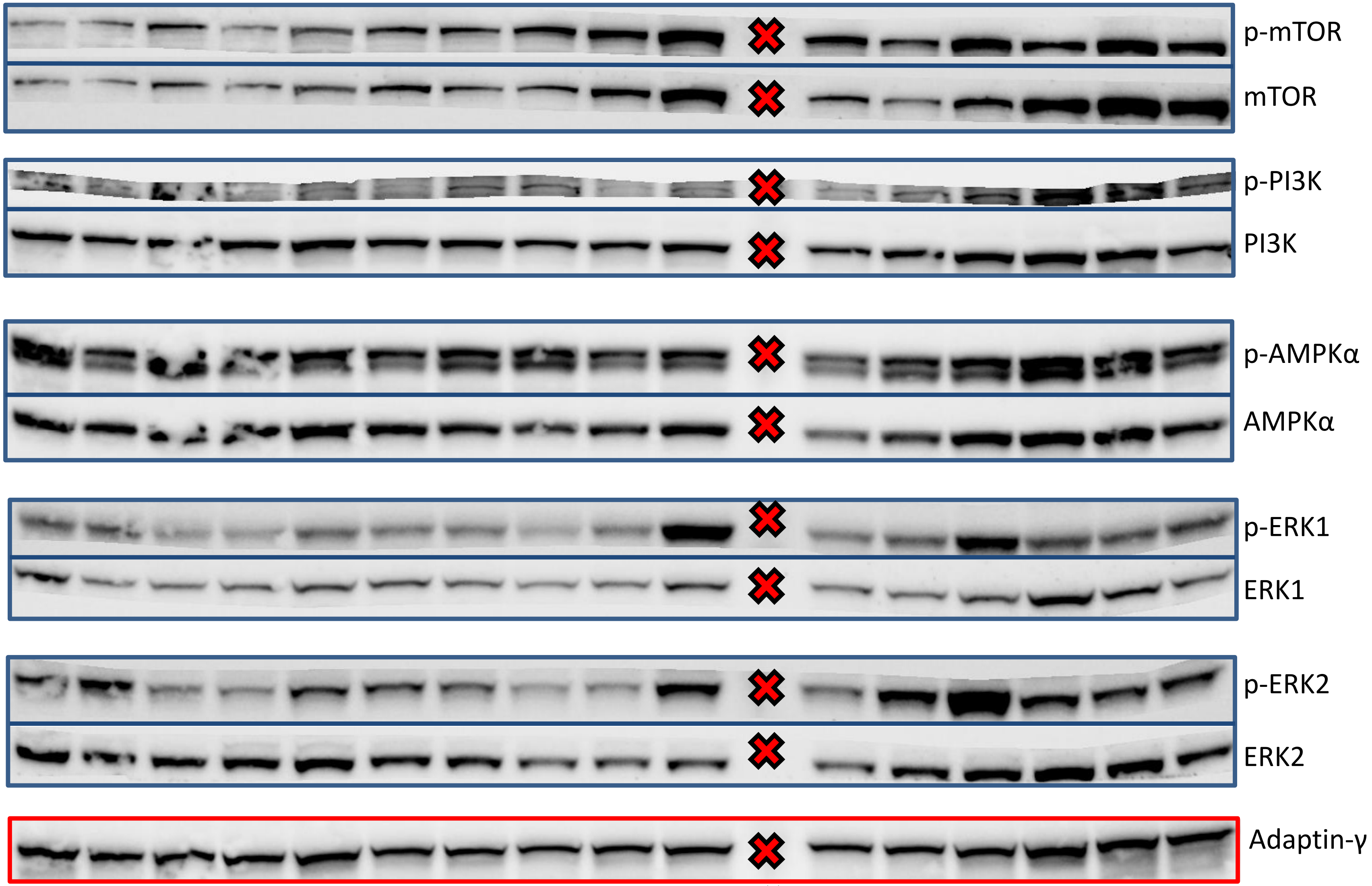

✖ Excluído por no haber proteína

mTOR (≈289 kDa)

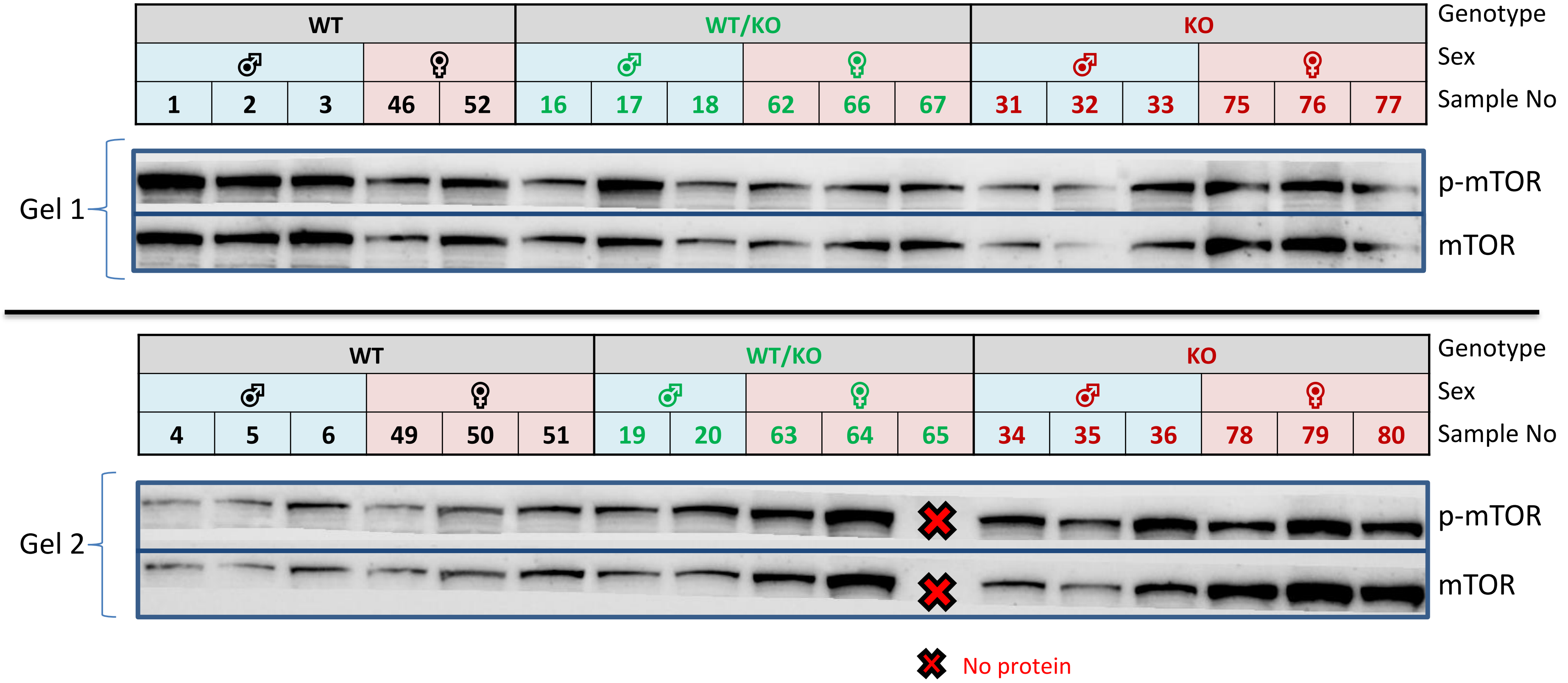
WT
WT/KO
KO
Genotype
Sex
Sample No
1 2 3 46 52 16 17 18 62 66 67 31 32 33 75 76 77
Gel 1
p-mTOR
mTOR
WT
WT/KO
KO
Genotype
Sex
Sample No
4 5 6 49 50 51 19 20 63 64 65 34 35 36 78 79 80
Gel 2
p-mTOR
mTOR
No protein

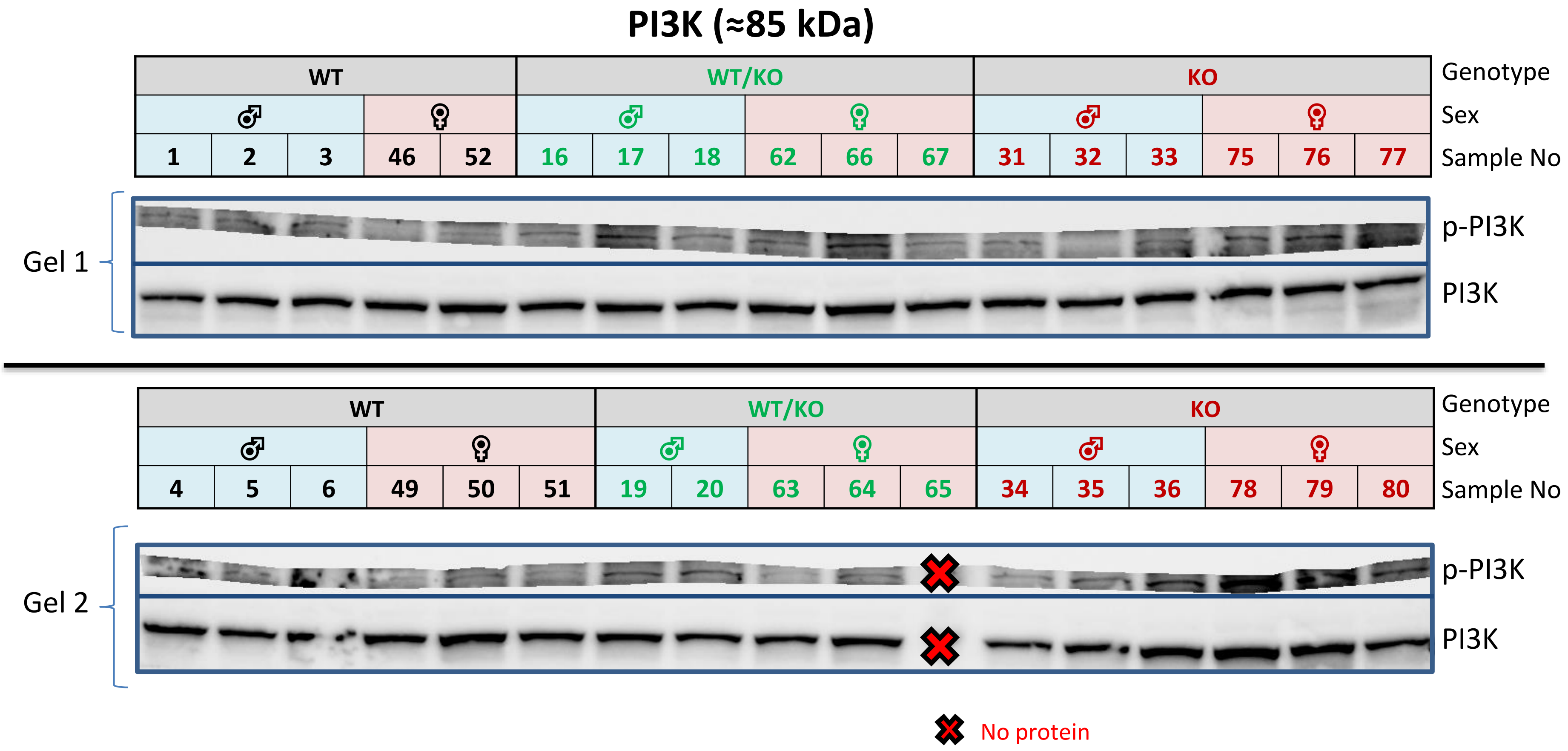
PI3K (≈85 kDa)
WT
WT/KO
KO
Genotype
Sex
1
2
3
46
52
16
17
18
62
66
67
31
32
33
75
76
77
Sample No
Gel 1
p-PI3K
PI3K
WT
WT/KO
KO
Genotype
Sex
4
5
6
49
50
51
19
20
63
64
65
34
35
36
78
79
80
Sample No
Gel 2
p-PI3K
PI3K
No protein

AMPKα (≈62 kDa)

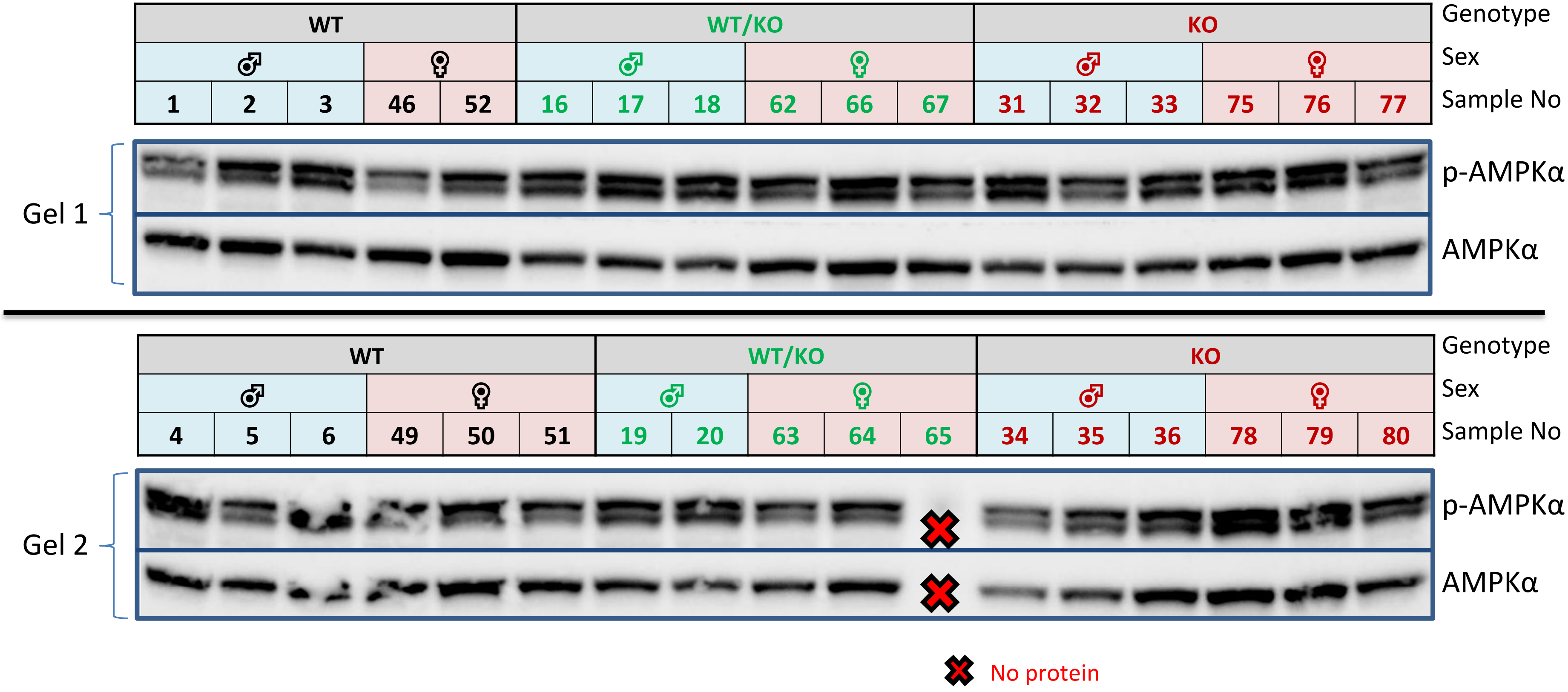
WT
WT/KO
KO
Genotype
Sex
Sample No
1 2 3 46 52 16 17 18 62 66 67 31 32 33 75 76 77
Gel 1
p-AMPKα
AMPKα
4 5 6 49 50 51 19 20 63 64 65 34 35 36 78 79 80
Gel 2
No protein

ERK1 (≈44 kDa)

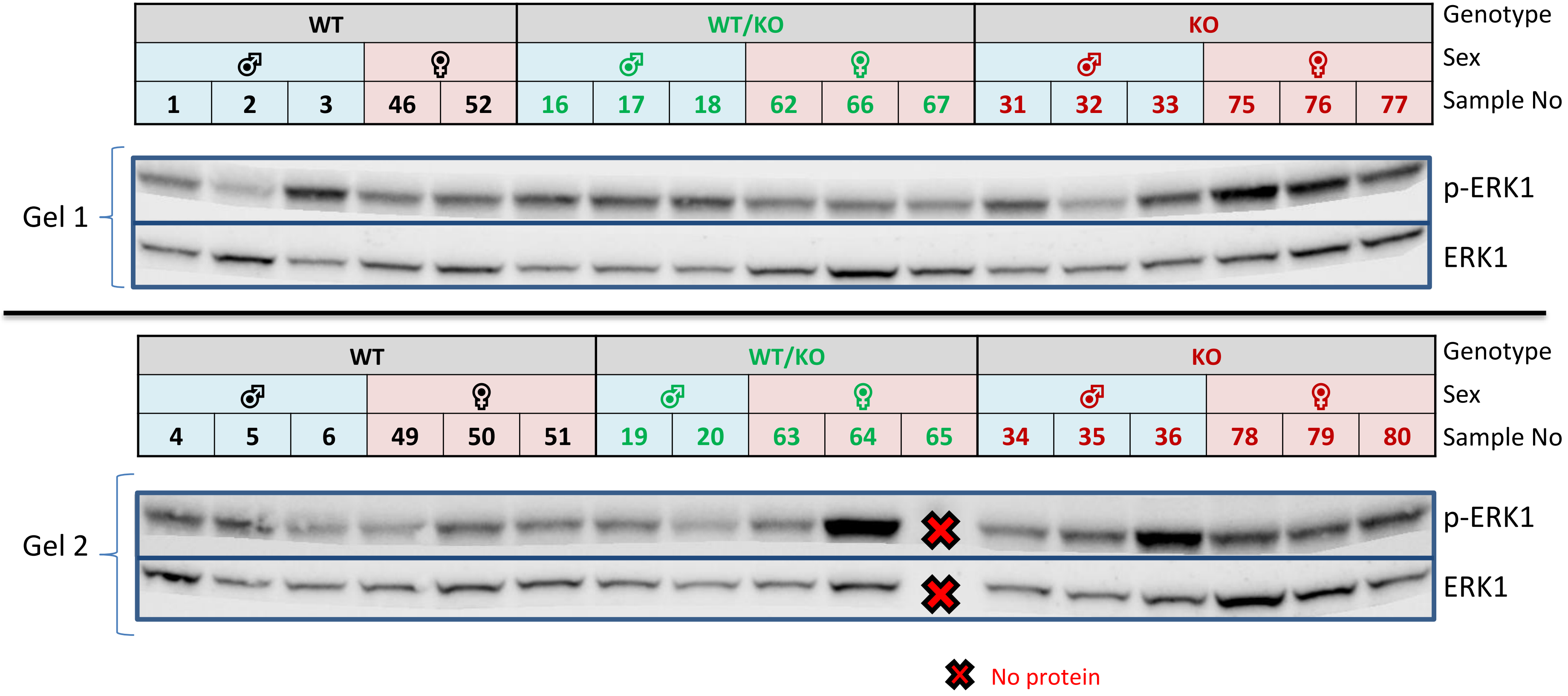

WT
WT/KO
KO
Genotype
Sex
Sample No
1 2 3 46 52 16 17 18 62 66 67 31 32 33 75 76 77
Gel 1
p-ERK1
ERK1
4 5 6 49 50 51 19 20 63 64 65 34 35 36 78 79 80
Gel 2
No protein

ERK2 (≈42 kDa)

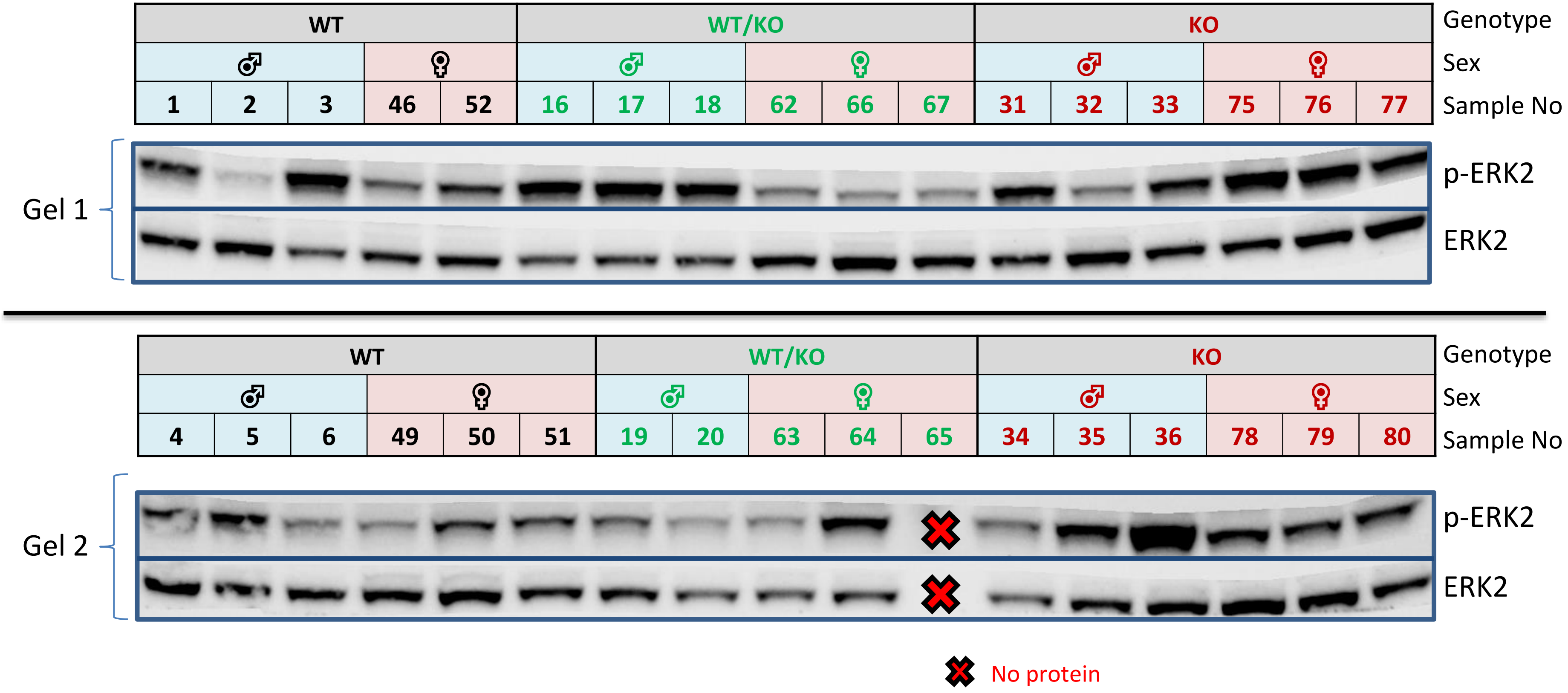
WT
WT/KO
KO
Genotype
Sex
1 2 3 46 52 16 17 18 62 66 67 31 32 33 75 76 77
Sample No
Gel 1
p-ERK2
ERK2
WT
WT/KO
KO
Genotype
Sex
4 5 6 49 50 51 19 20 63 64 65 34 35 36 78 79 80
Sample No
Gel 2
p-ERK2
ERK2
No protein

2º batch

(3/12/2021)

Gel 1

WT					WT/KO						KO						Genotype
♂			♀		♂			♀			♂			♀			Sex
1	2	3	46	52	16	17	18	62	66	67	31	32	33	75	76	77	Sample No

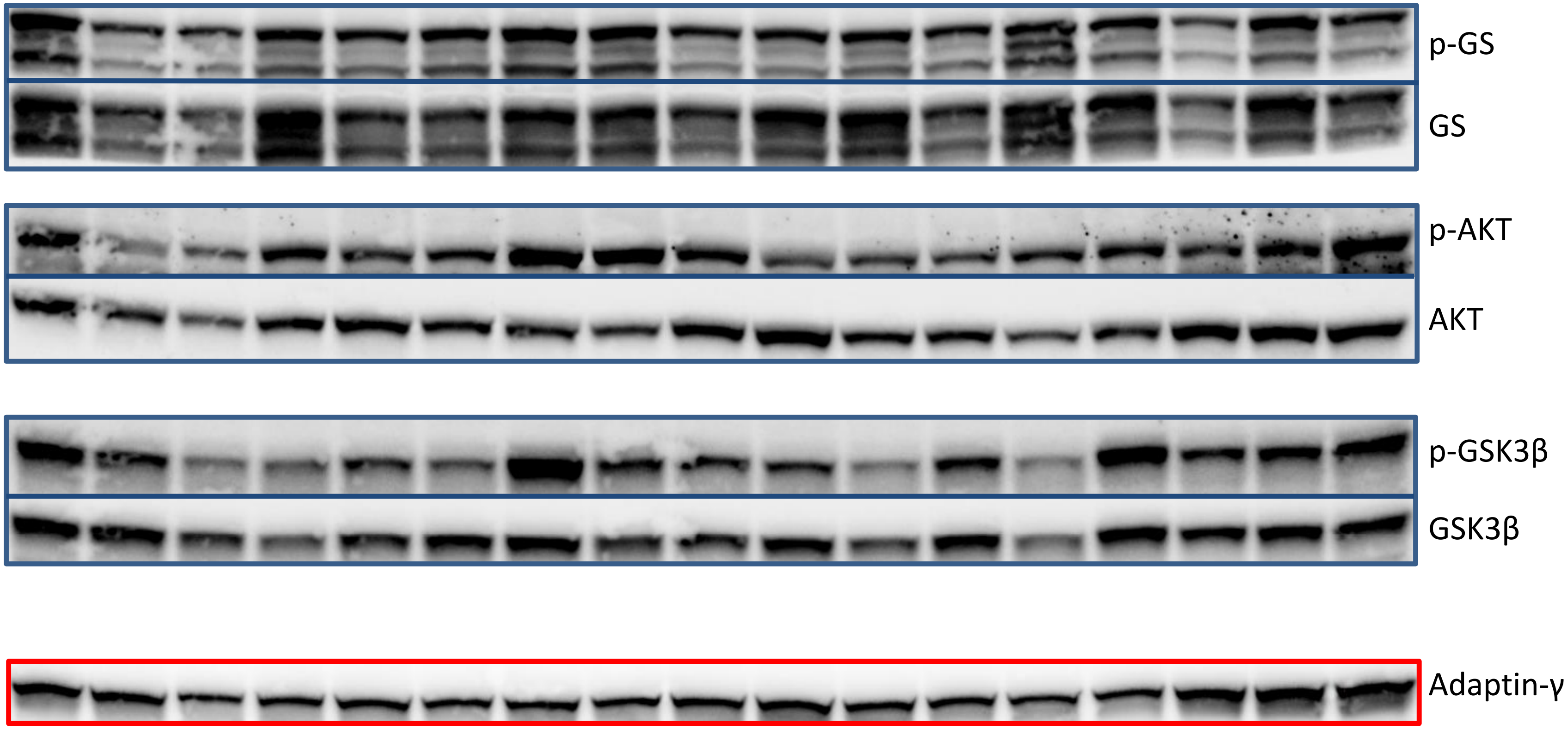

Gel 2

WT						WT/KO				WT	KO						Genotype
♂			♀			♂		♀		♂	♂			♀			Sex
4	5	6	49	50	51	19	20	63	64		34	35	36	78	79	80	Sample No

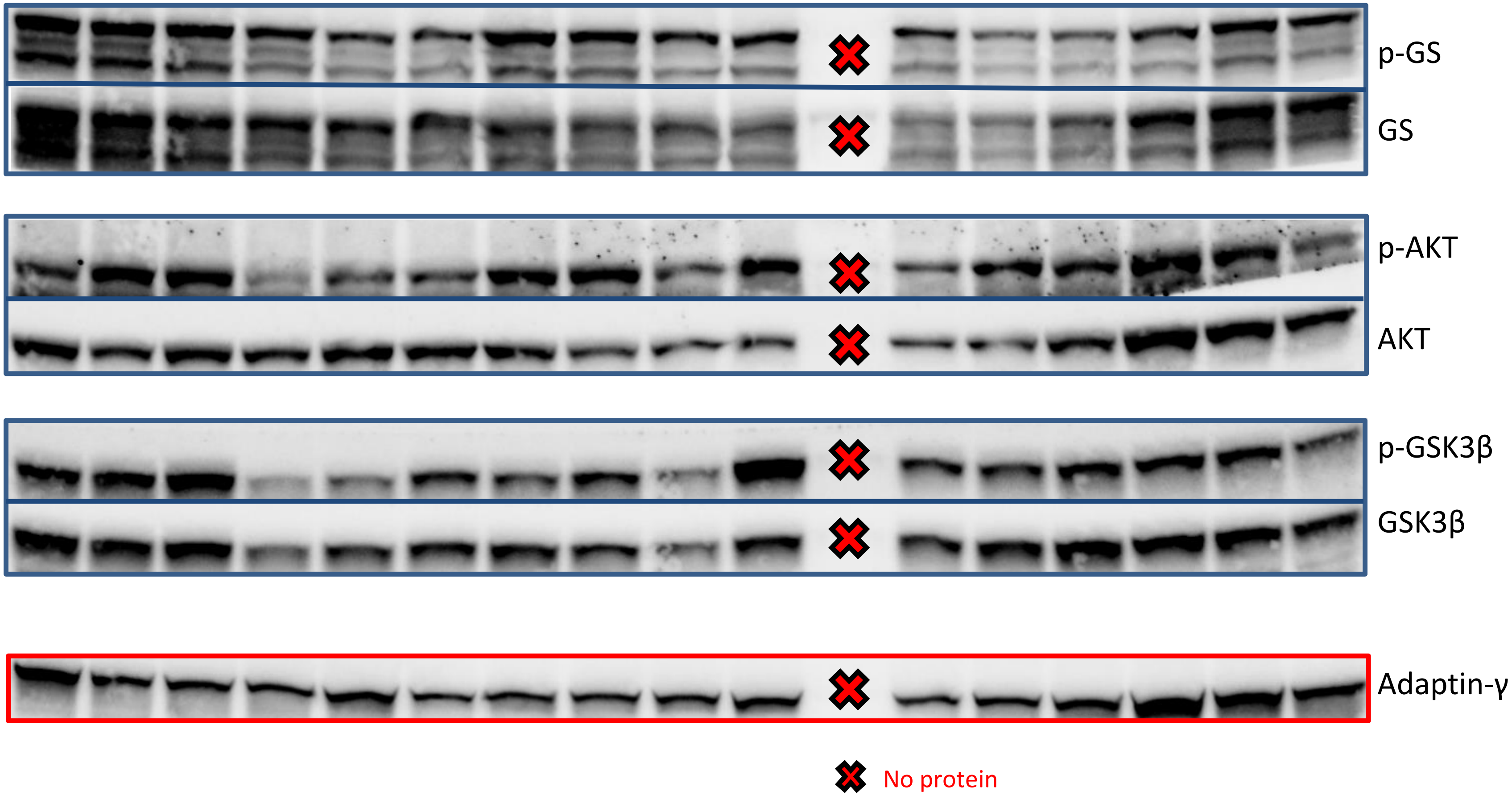

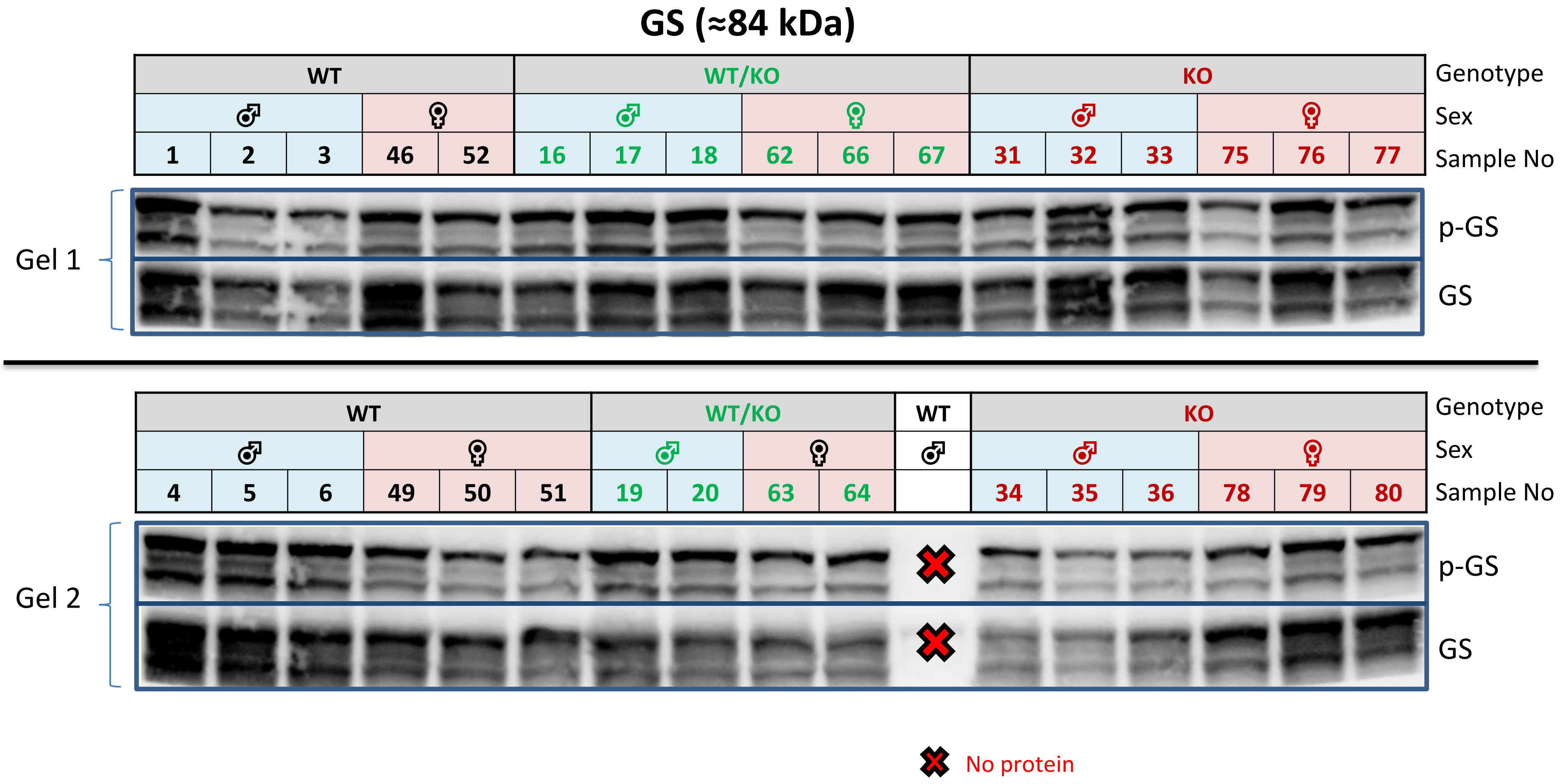
GS (≈84 kDa)
WT
WT/KO
KO
Genotype
Sex
Sample No
1
2
3
46
52
16
17
18
62
66
67
31
32
33
75
76
77
Gel 1
p-GS
GS
WT
WT/KO
WT
KO
Genotype
Sex
Sample No
4
5
6
49
50
51
19
20
63
64
34
35
36
78
79
80
Gel 2
p-GS
GS
No protein

AKT (≈60 kDa)

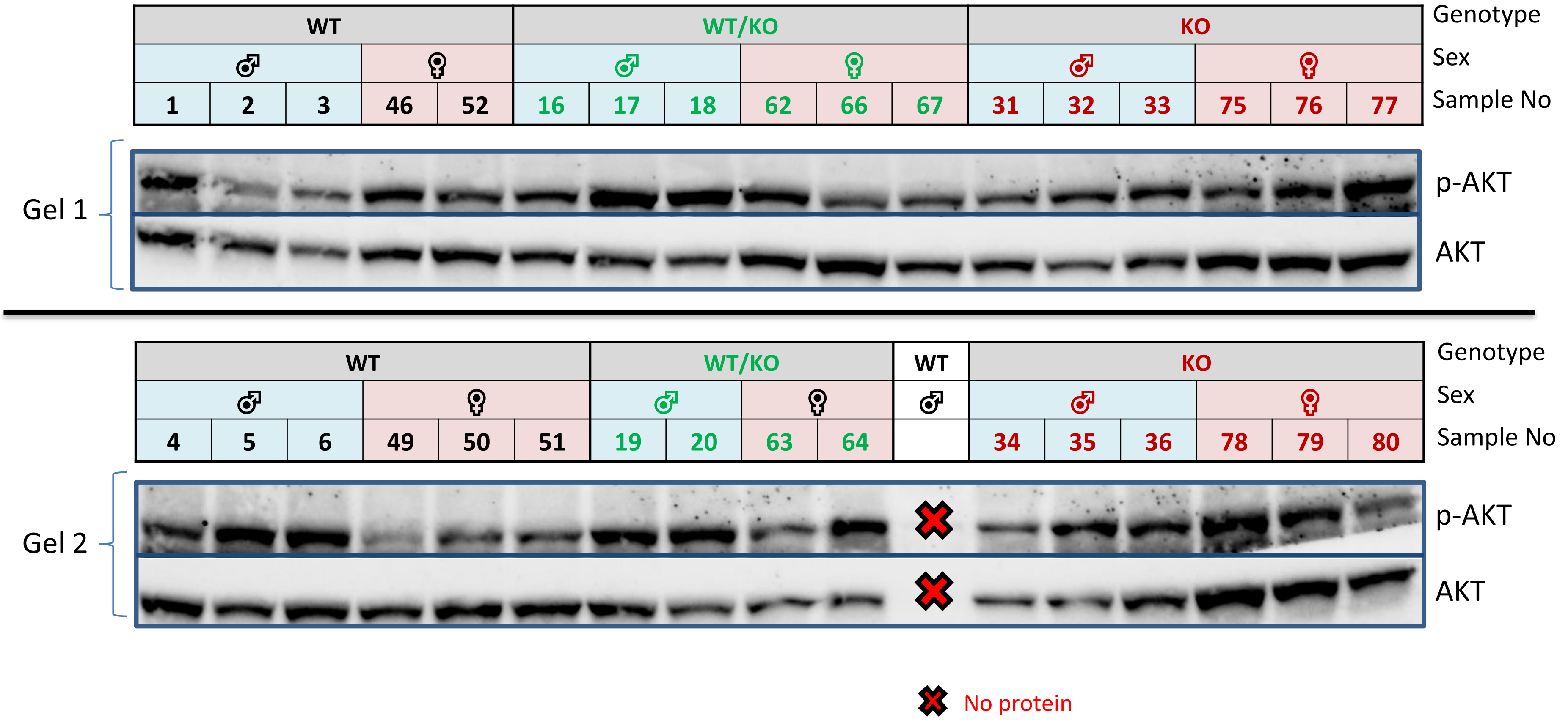

Genotype
Sex
Sample No
WT
WT/KO
KO
1 2 3 46 52 16 17 18 62 66 67 31 32 33 75 76 77
Gel 1
p-AKT
AKT
WT
WT/KO
WT
KO
4 5 6 49 50 51 19 20 63 64 34 35 36 78 79 80
Gel 2
p-AKT
AKT
No protein

GSK3β (≈46 kDa)

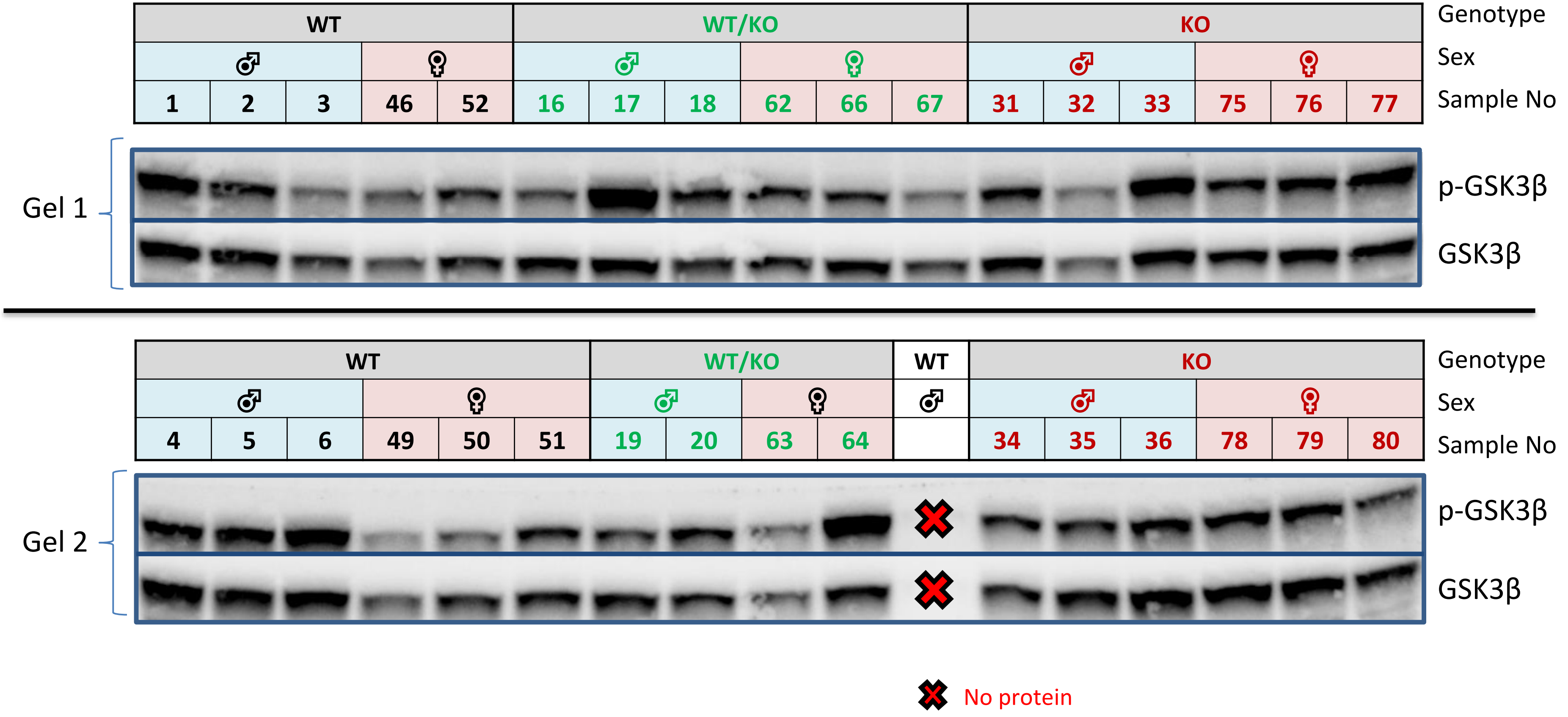

WT
WT/KO
KO
Genotype
Sex
Sample No
1 2 3 46 52
16 17 18 62 66 67
31 32 33 75 76 77
Gel 1
p-GSK3β
GSK3β
WT
WT/KO
WT
KO
Genotype
Sex
Sample No
4 5 6 49 50 51
19 20 63 64
34 35 36 78 79 80
Gel 2
p-GSK3β
GSK3β
No protein
