# Supplementary material for: Sex-based differences in growth-related IGF1 signaling in response to PAPP-A2 deficiency: comparative effects of rhGH, rhIGF1 and rhPAPP-A2 treatments
Source: Biol Sex Differ. 2024 Apr 8;15:34. doi: 10.1186/s13293-024-00603-5 (PMC11000399; doi:10.1186/s13293-024-00603-5)

Figure S4.

A. Unedited mTOR blots in male and female *Pappa2*$^{ko/ko}$ after rhGH, rhIGF1 and rhPAPP-A2 treatments

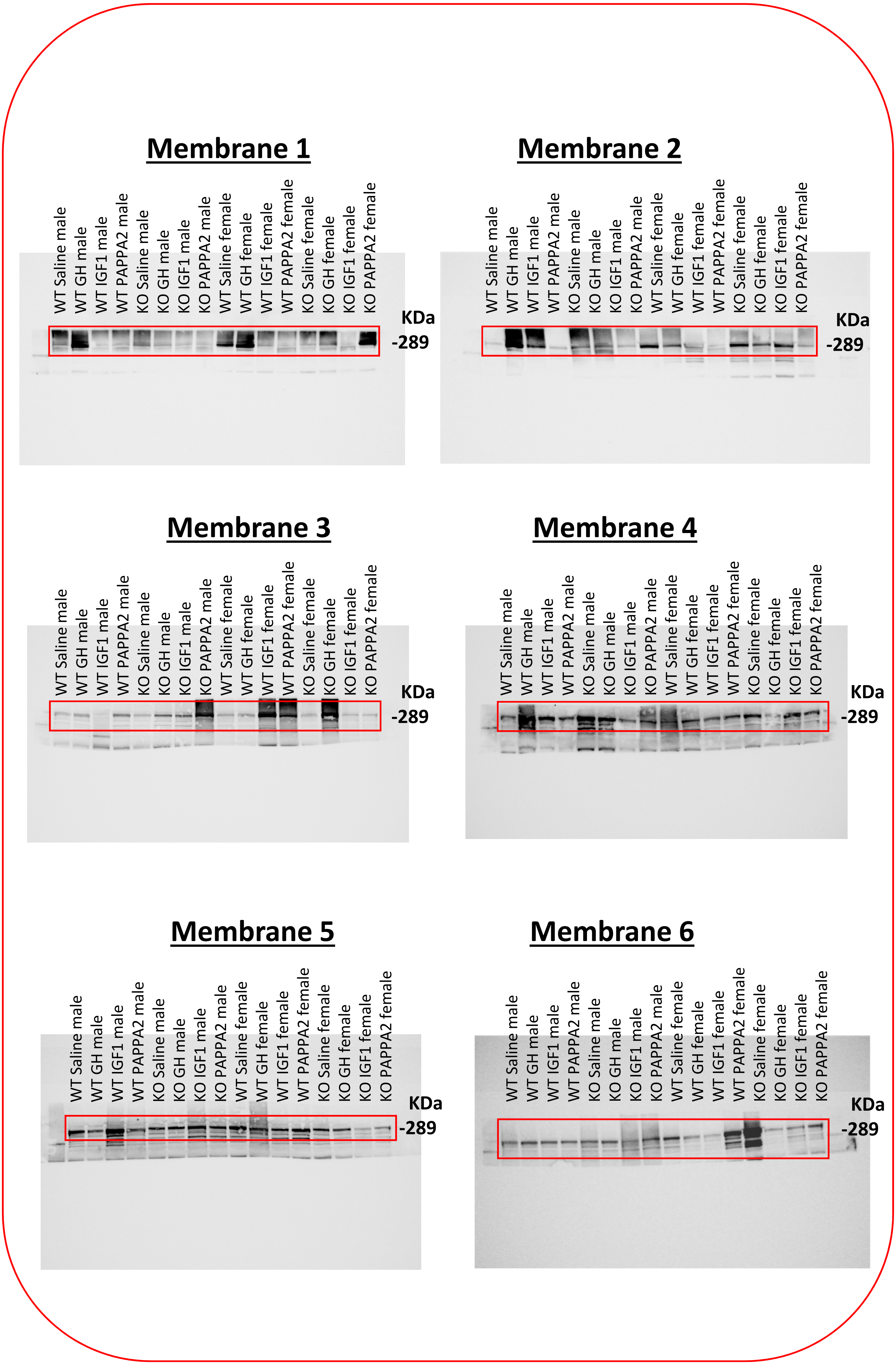

Figure S4.

B. Unedited p(S^{2448})-mTOR blots in male and female *Pappa2*$^{ko/ko}$ after rhGH, rhIGF1 and rhPAPP-A2 treatments

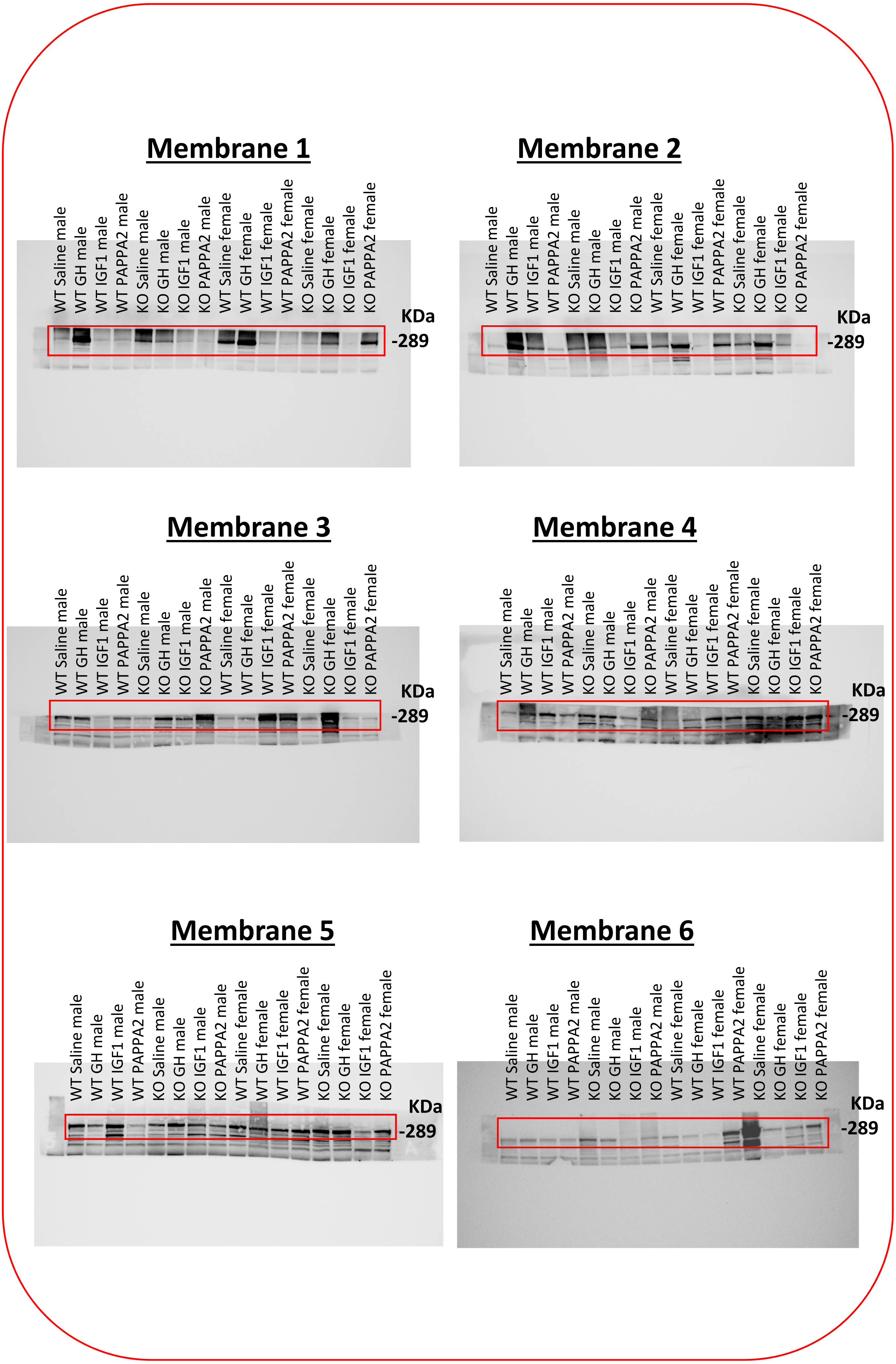

Figure S4.
C. Unedited IRS1 blots in male and female *Pappa2*$^{ko/ko}$ after rhGH, rhIGF1 and rhPAPP-A2 treatments

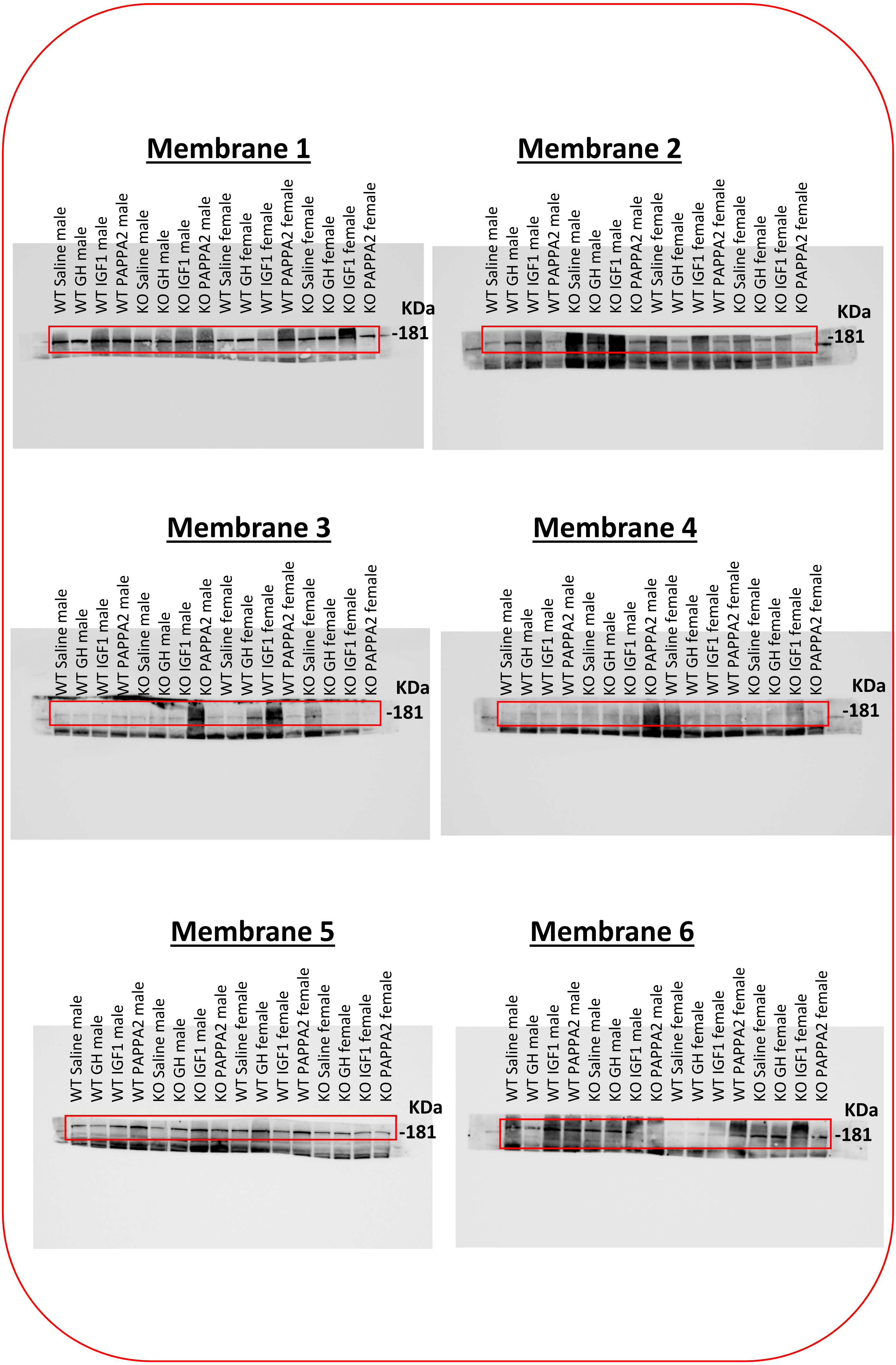

Figure S4.
D. Unedited p(Y^{618})-IRS1 blots in male and female *Pappa2*$^{ko/ko}$ after rhGH, rhIGF1 and rhPAPP-A2 treatments

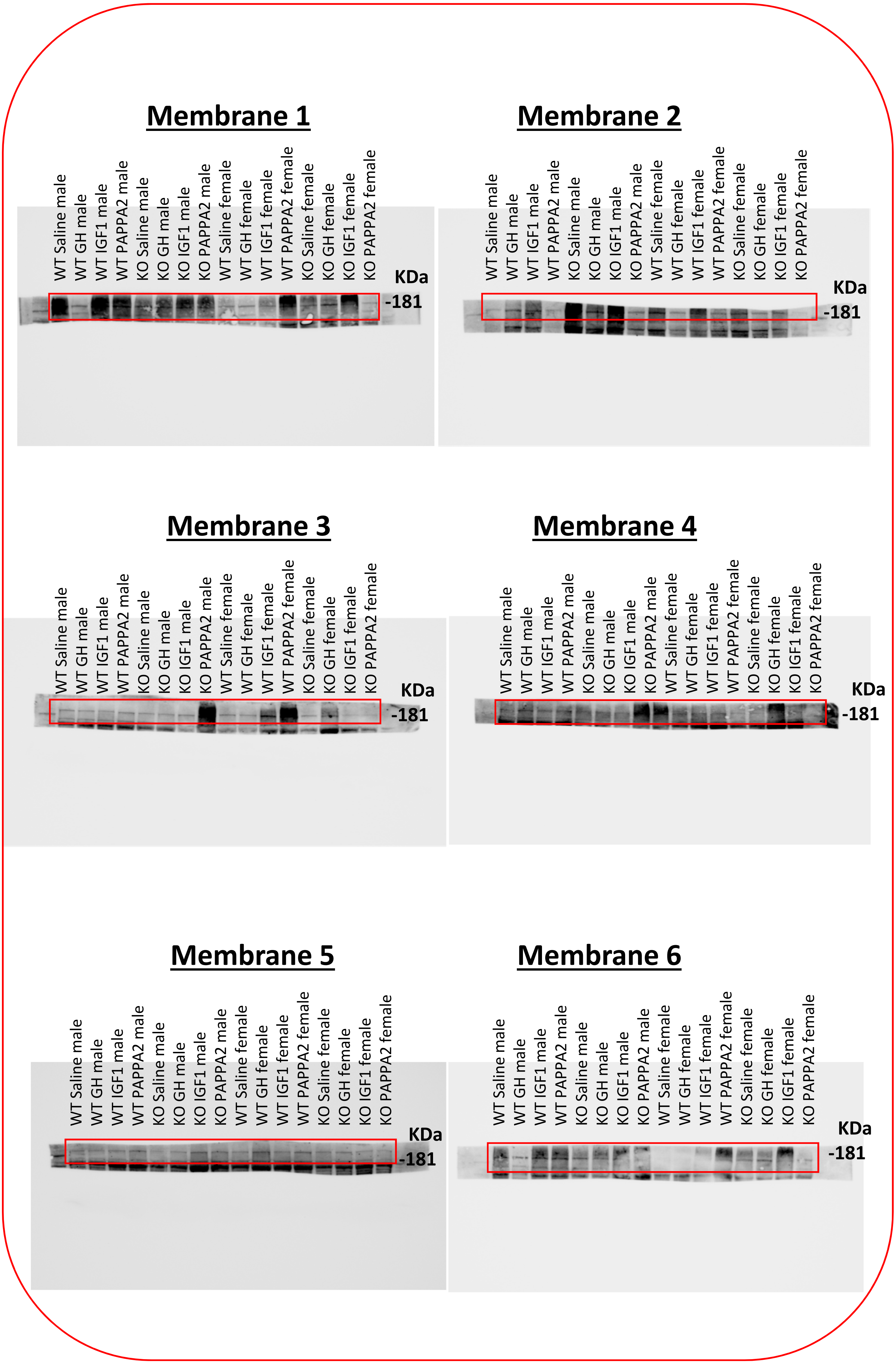

Figure S4.

E. Unedited p(S^{612})**-**IRS1 blots in male and female *Pappa2*$^{ko/ko}$ after rhGH, rhIGF1 and rhPAPP-A2 treatments

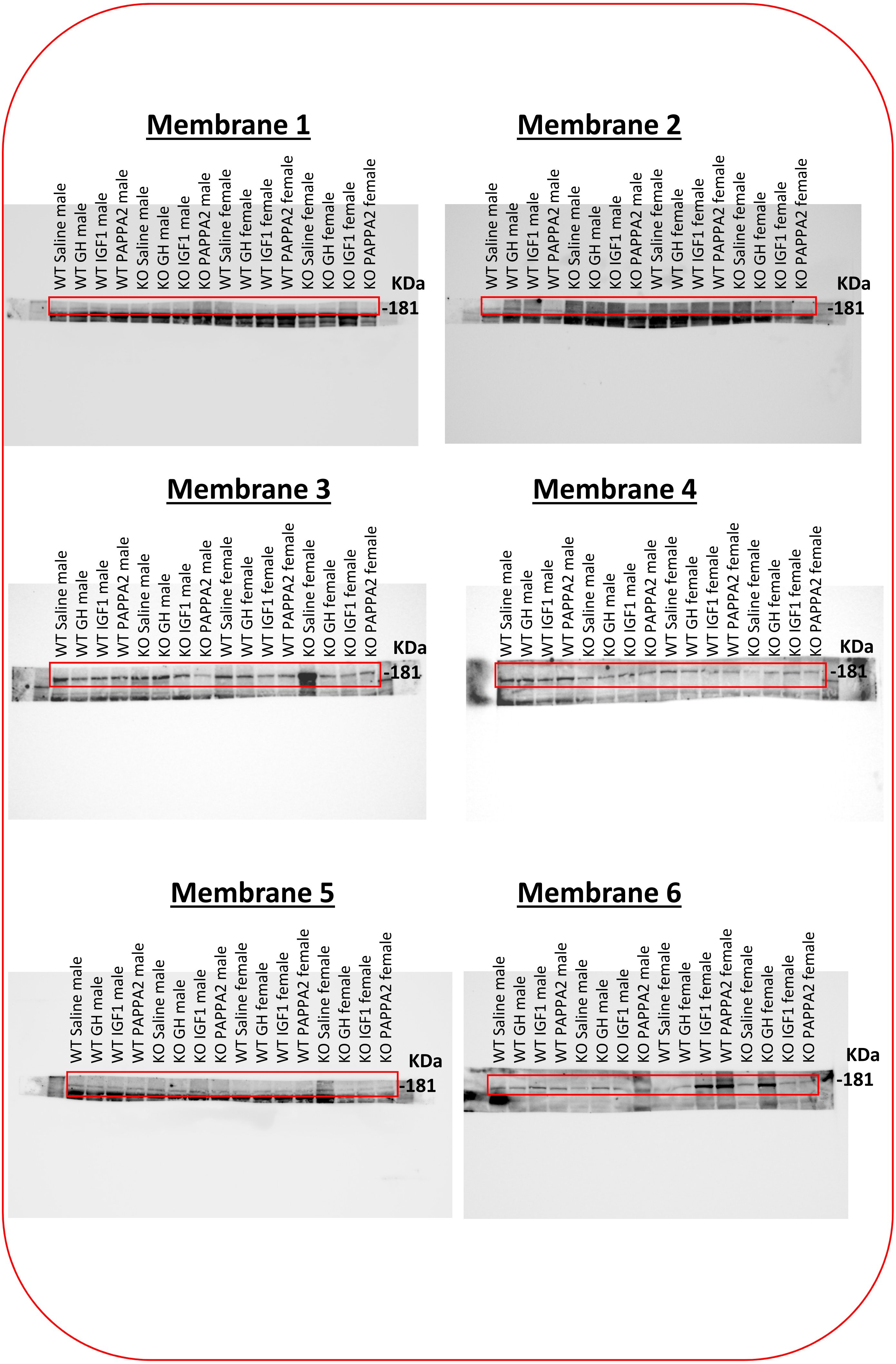

Figure S4.
F. Unedited PI3K blots in male and female *Pappa2*$^{ko/ko}$ after rhGH, rhIGF1 and rhPAPP-A2 treatments

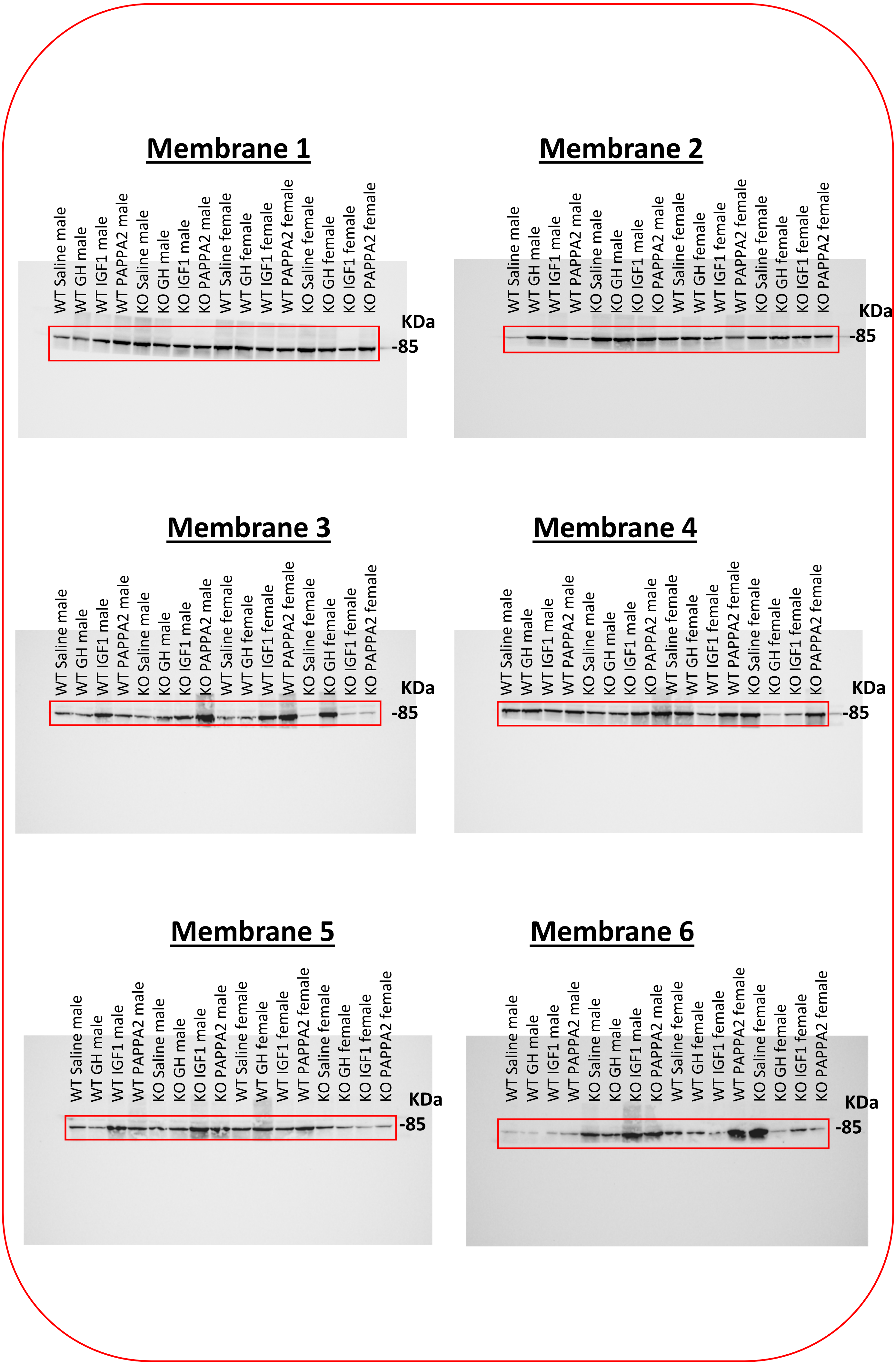

Figure S4.

G. Unedited p(Y^{607})-PI3K blots in male and female *Pappa2*$^{ko/ko}$ after rhGH, rhIGF1 and rhPAPP-A2 treatments

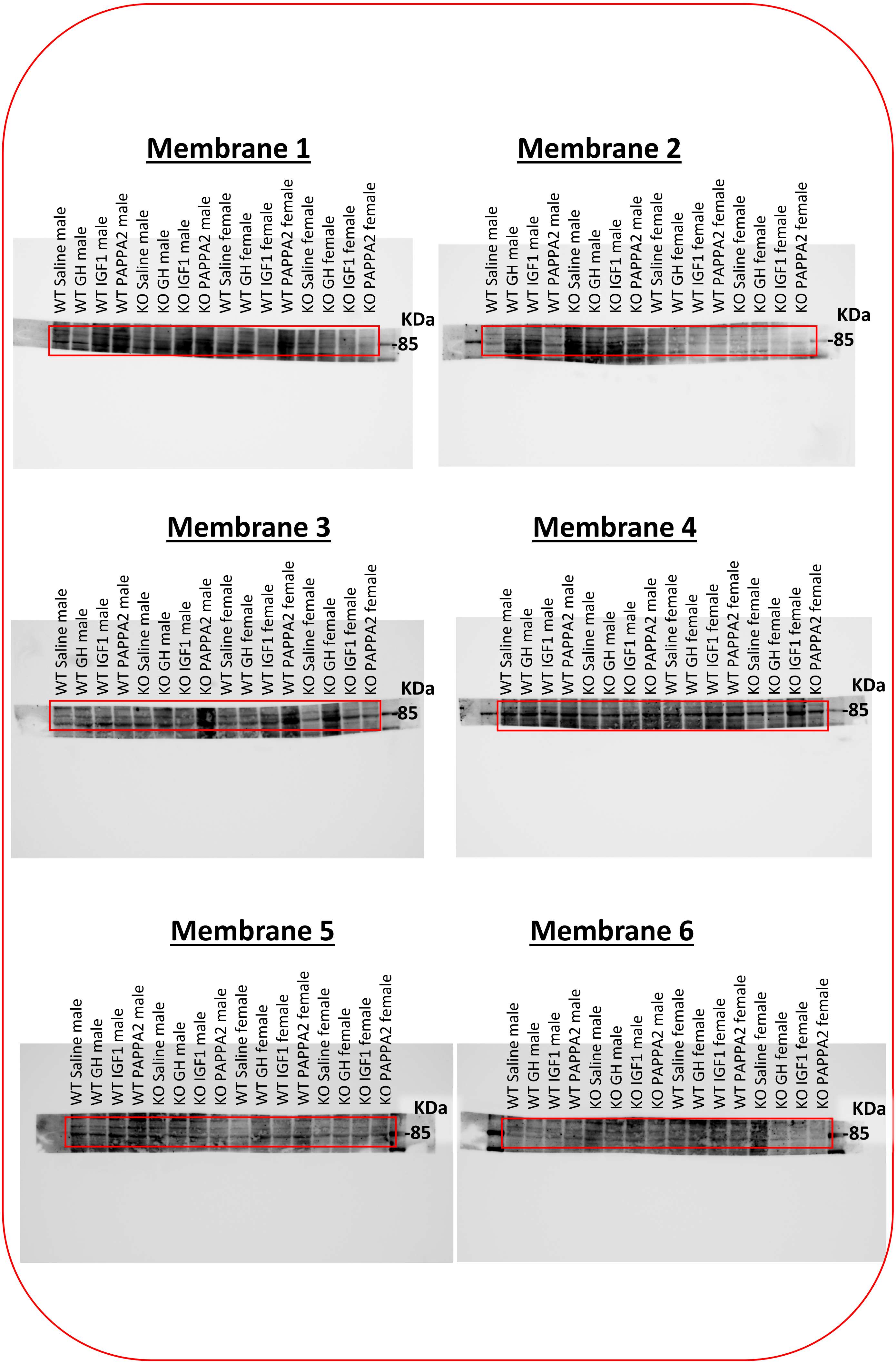

Figure S4.

H. Unedited AKT blots in male and female *Pappa2*$^{ko/ko}$ after rhGH, rhIGF1 and rhPAPP-A2 treatments

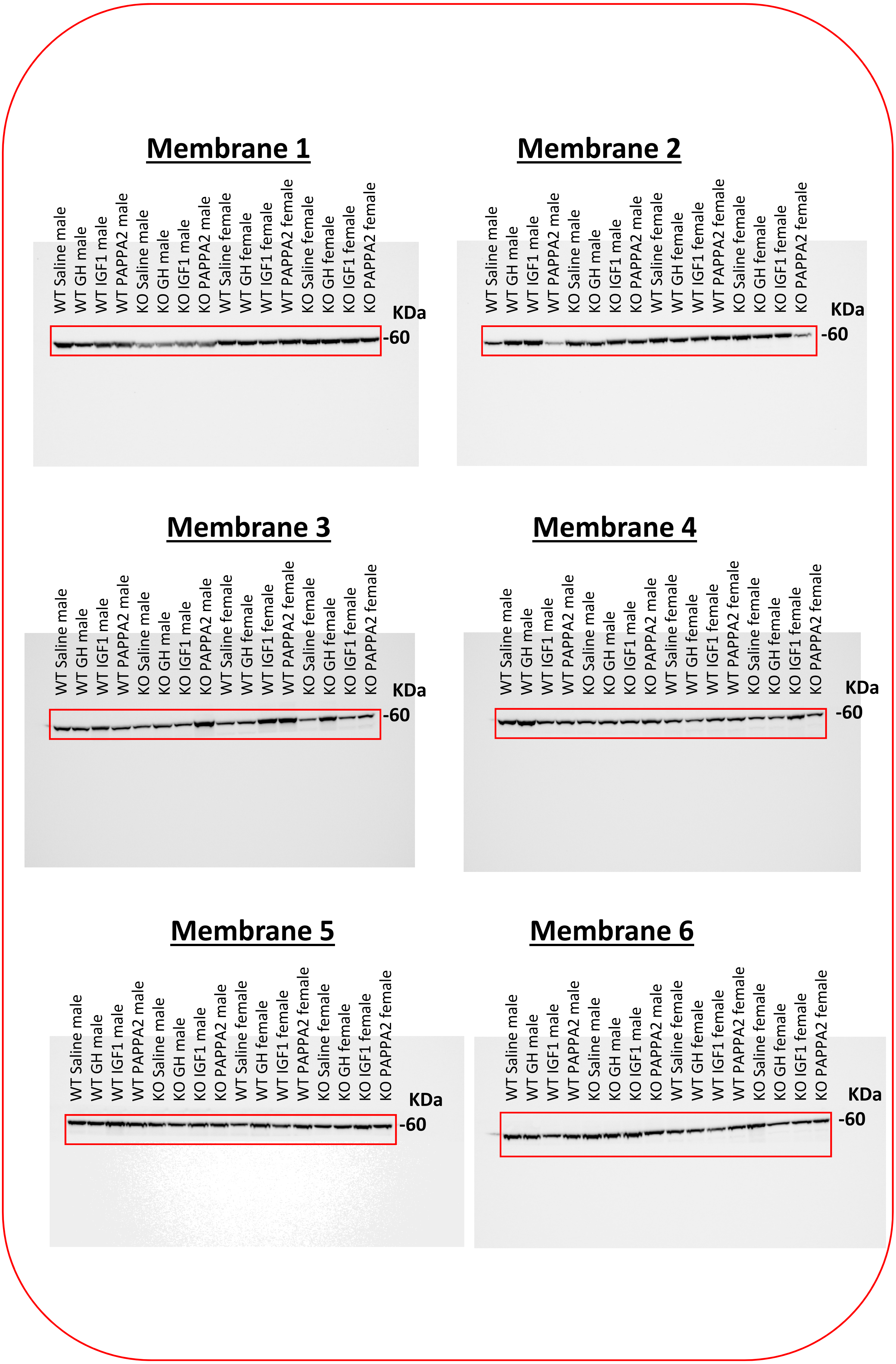

Figure S4.

I. Unedited p(S^{473})-AKT blots in male and female *Pappa2*$^{ko/ko}$ after rhGH, rhIGF1 and rhPAPP-A2 treatments

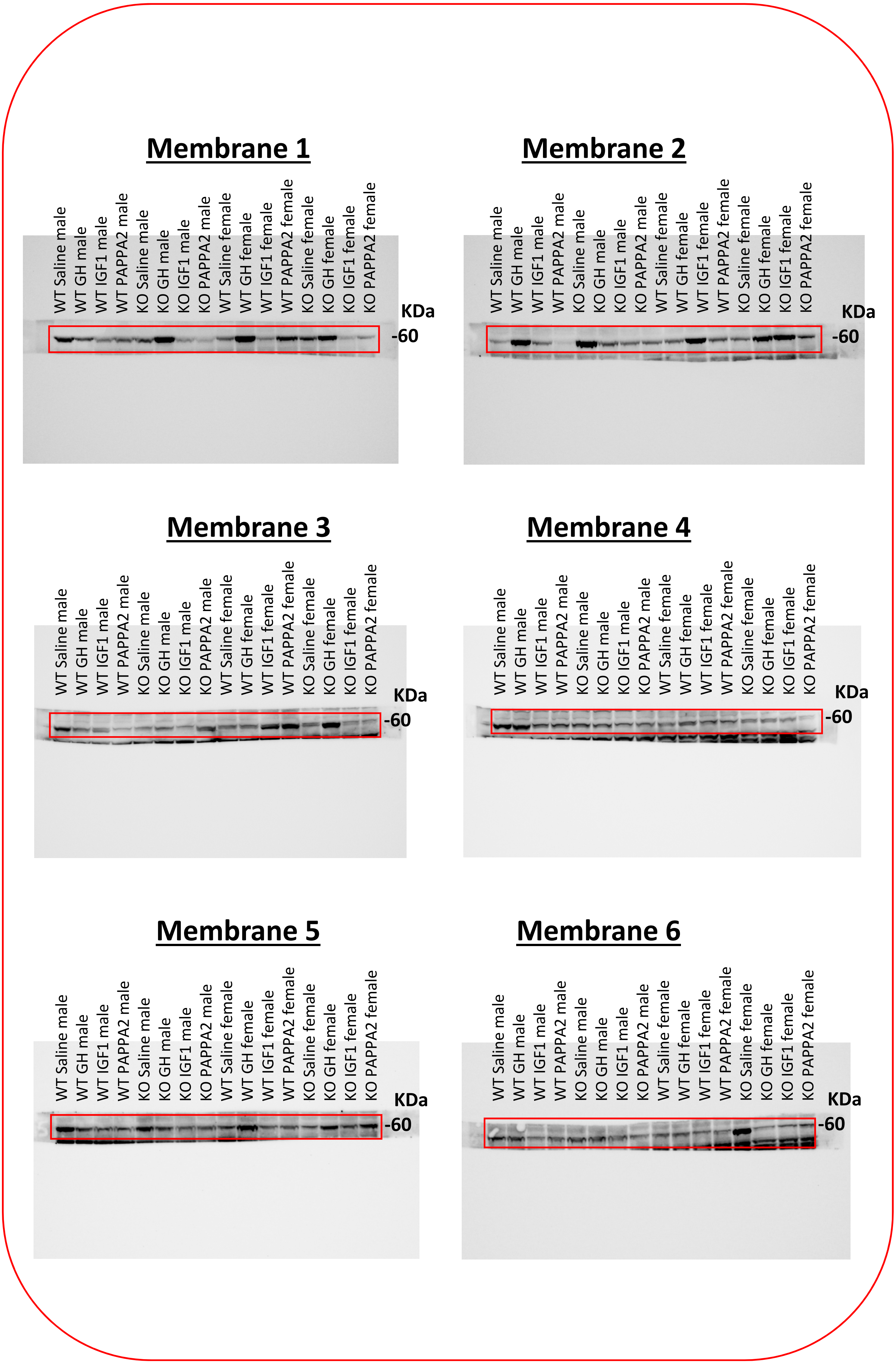

Figure S4.
J. Unedited AMPKα blots in male and female *Pappa2*$^{ko/ko}$ after rhGH, rhIGF1 and rhPAPP-A2 treatments

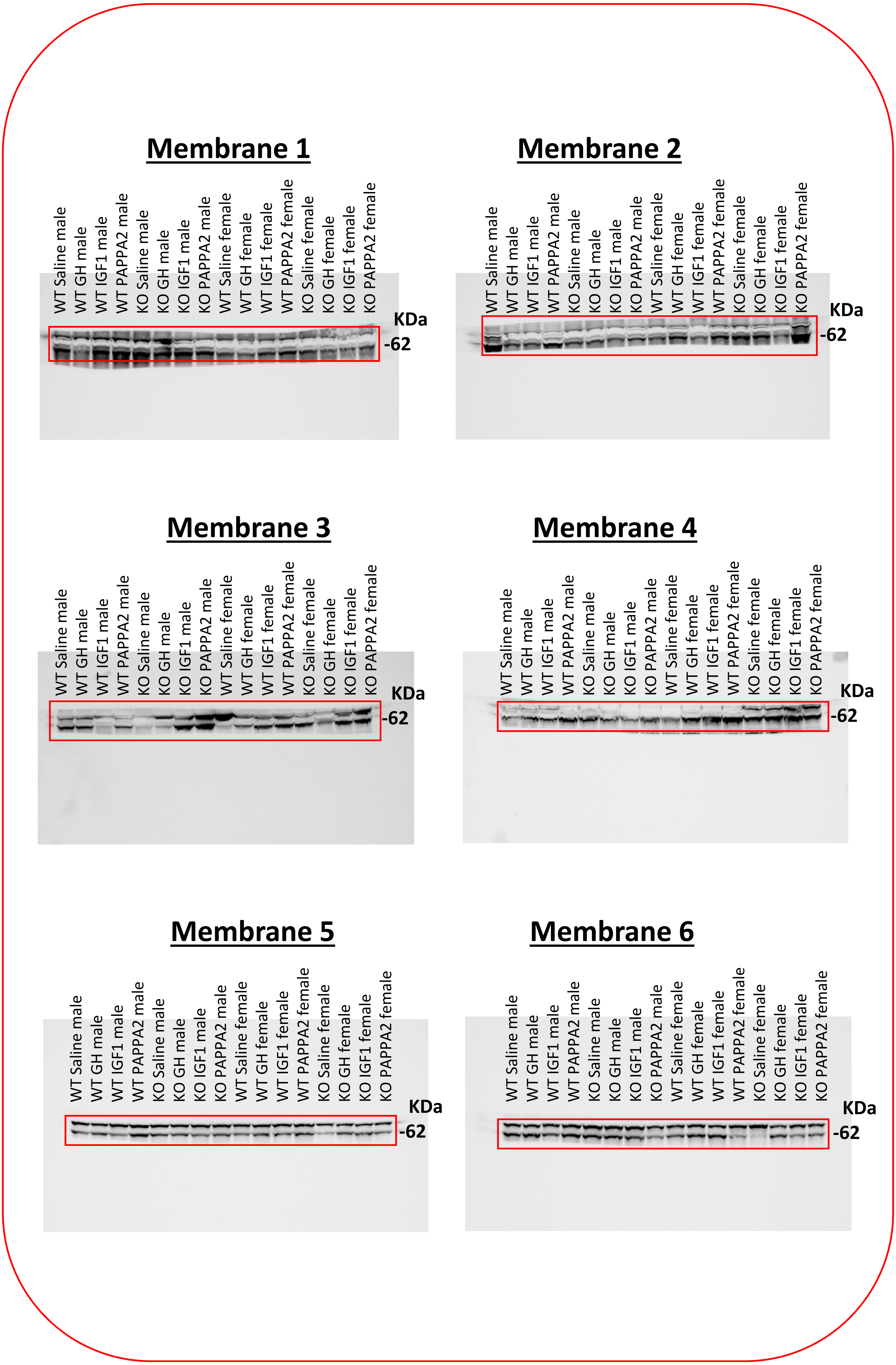

Figure S4.
K. Unedited p(T^{172})-AMPKα blots in male and female *Pappa2*$^{ko/ko}$ after rhGH, rhIGF1 and rhPAPP-A2 treatments

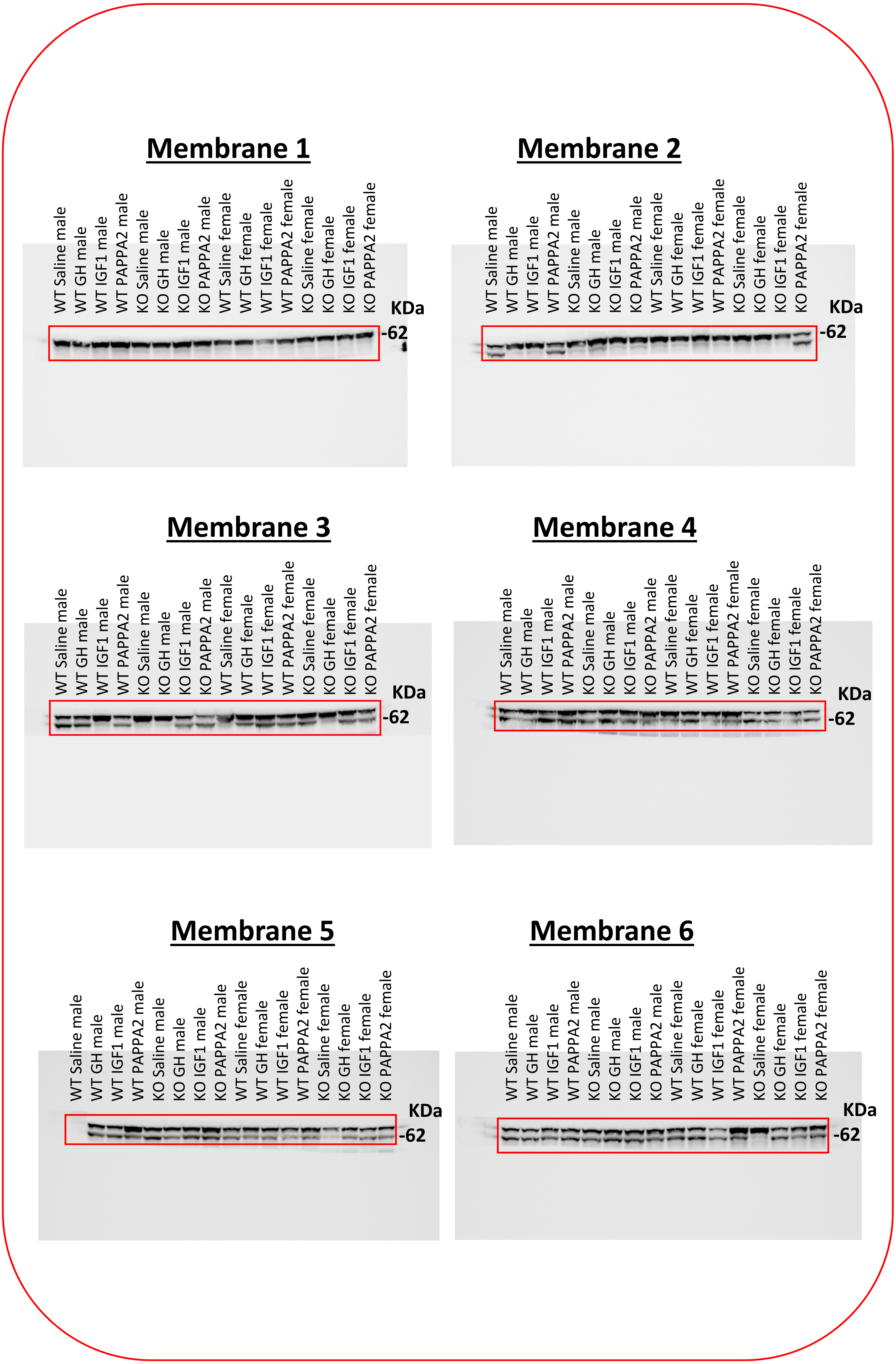

Figure S4.
L. Unedited GSK3β blots in male and female *Pappa2*$^{ko/ko}$ after rhGH, rhIGF1 and rhPAPP-A2 treatments

Membrane 1

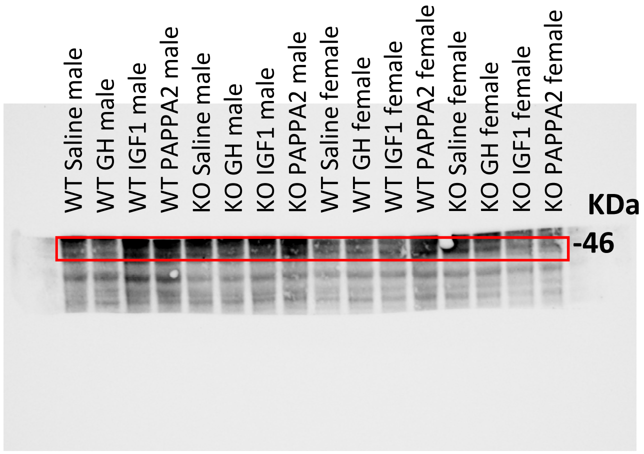

Membrane 2

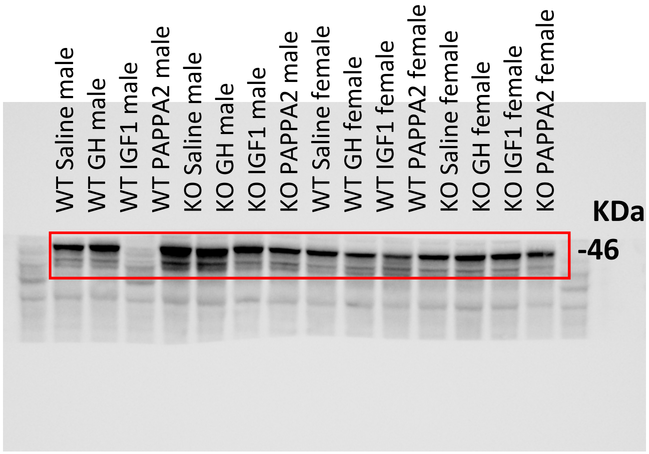

Membrane 3

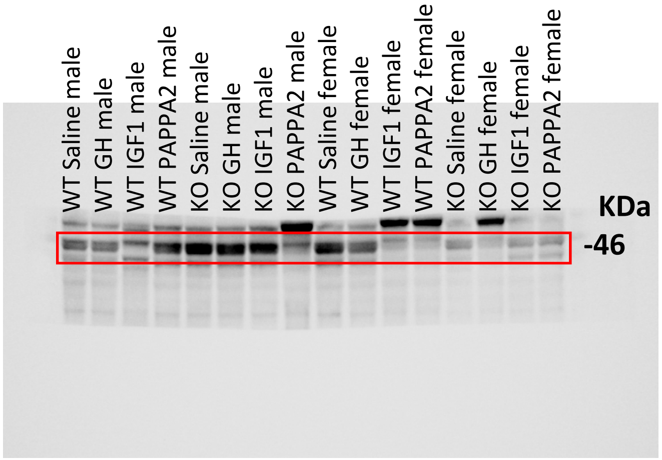

Membrane 4

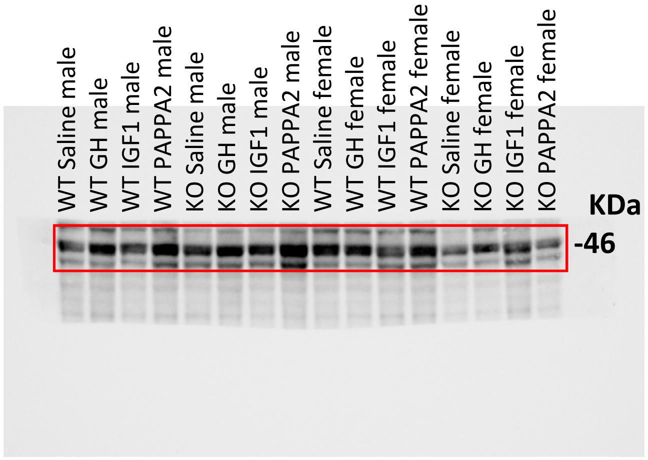

Membrane 5

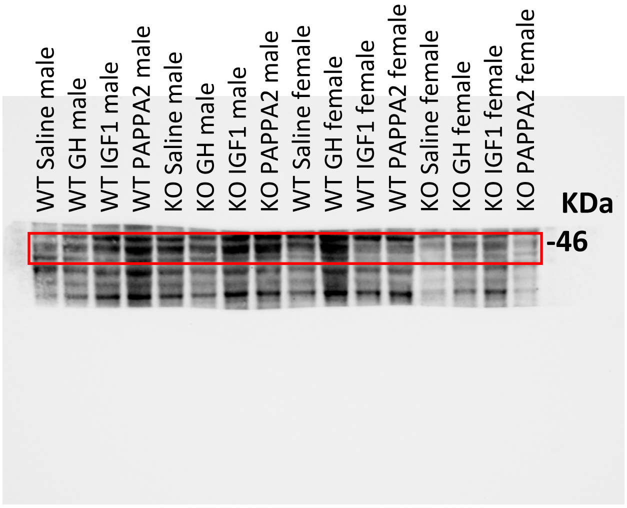

Membrane 6

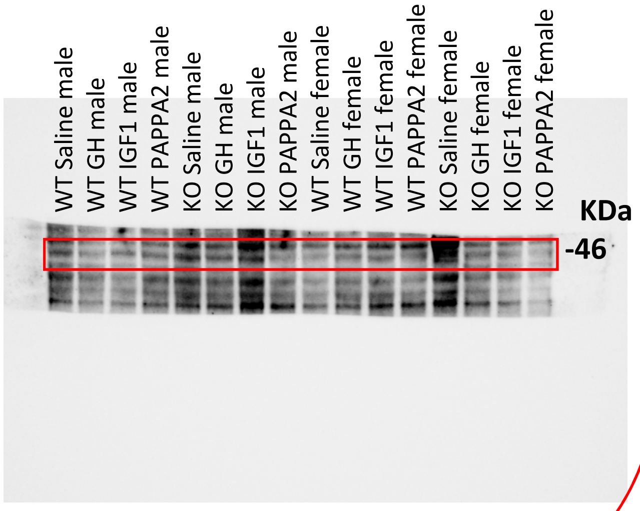

Figure S4.
M. Unedited p(Y^{279})-GSK3β blots in male and female *Pappa2*$^{ko/ko}$ after rhGH, rhIGF1 and rhPAPP-A2 treatments

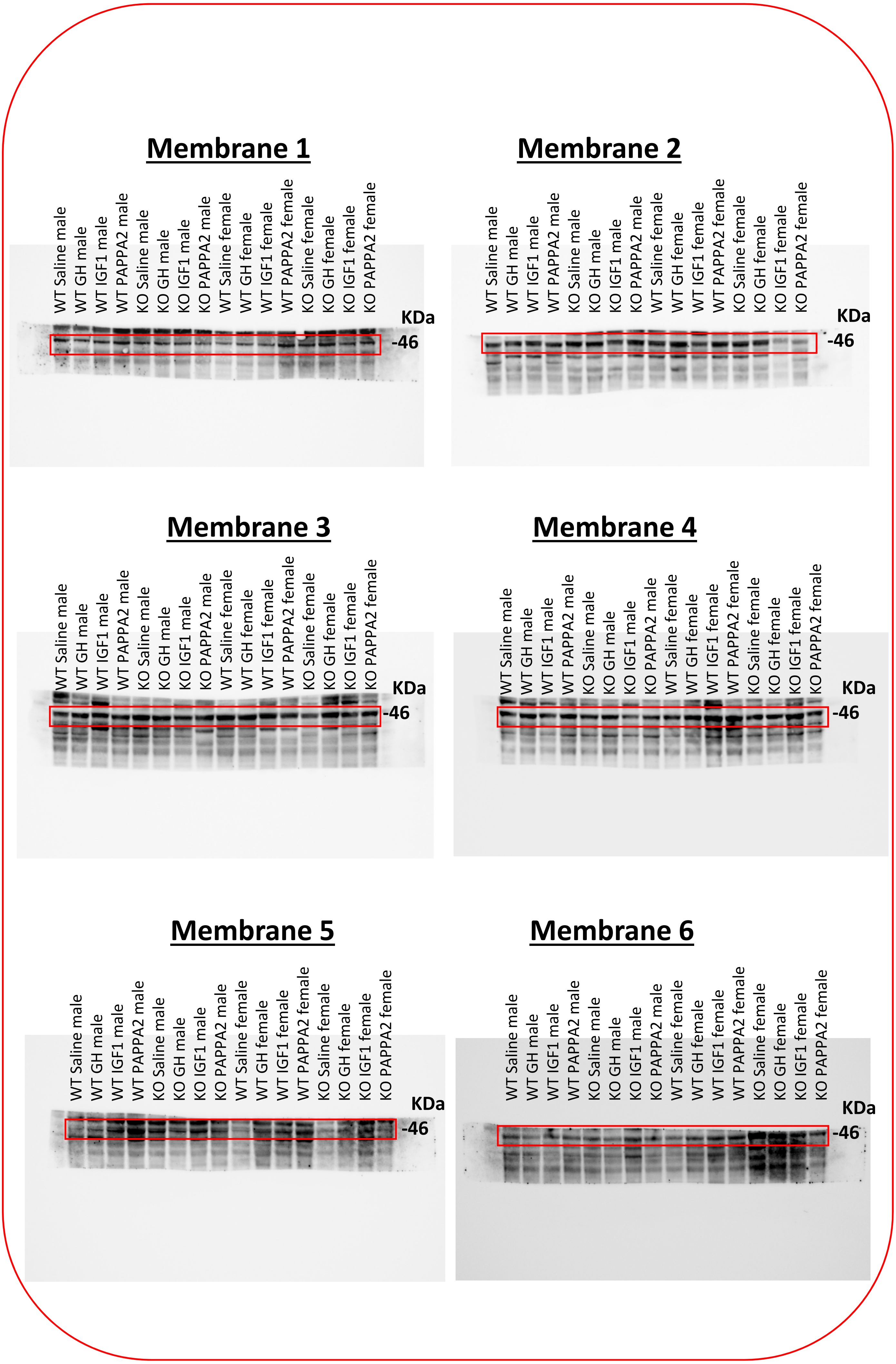

Figure S4

N. Unedited p(S^{609})-GSK3β blots in male and female *Pappa2*$^{ko/ko}$ after rhGH, rhIGF1 and rhPAPP-A2 treatments

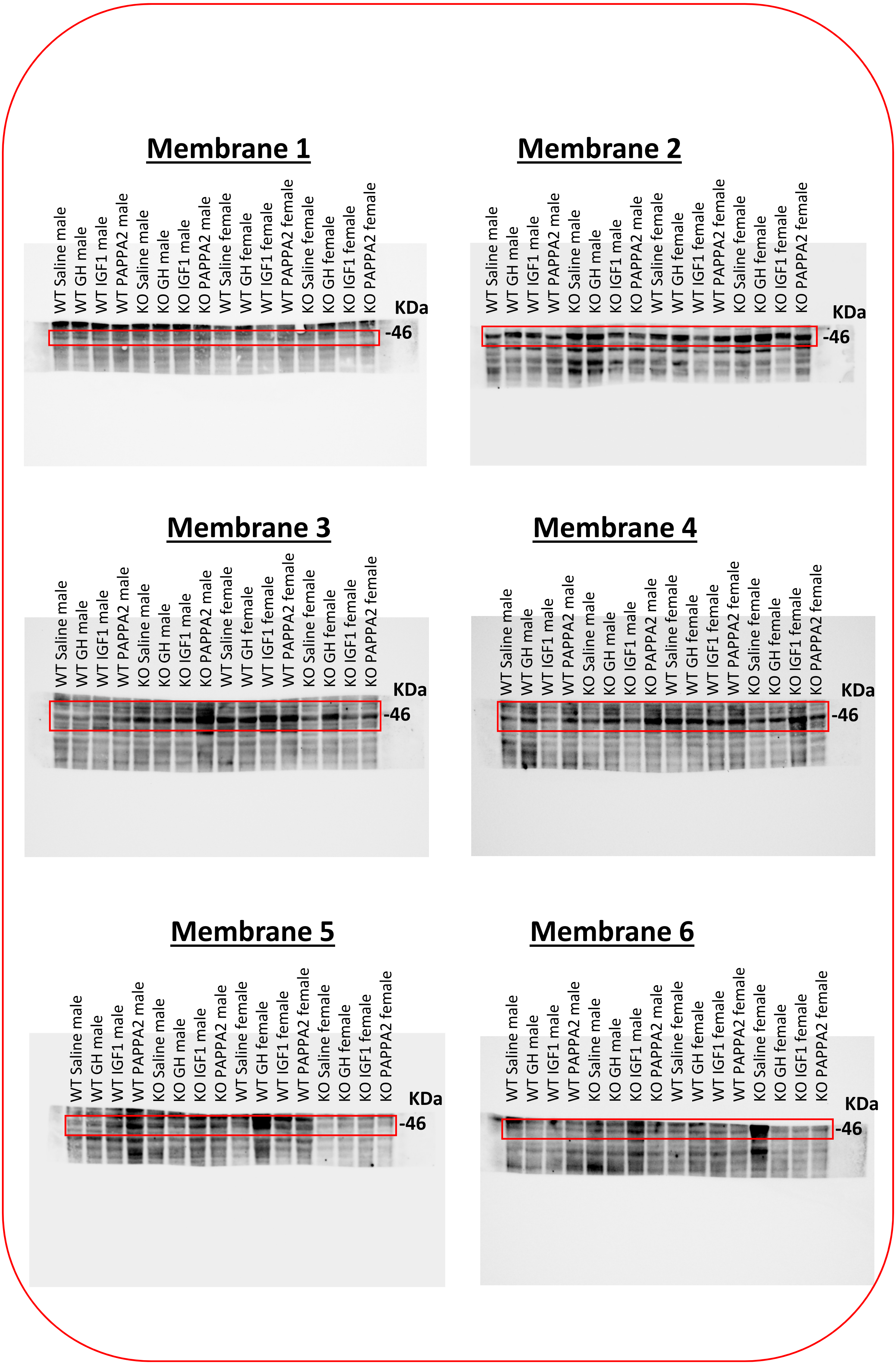

Figure S4

O. Unedited ERK1/2 blots in male and female *Pappa2*$^{ko/ko}$ after rhGH, rhIGF1 and rhPAPP-A2 treatments

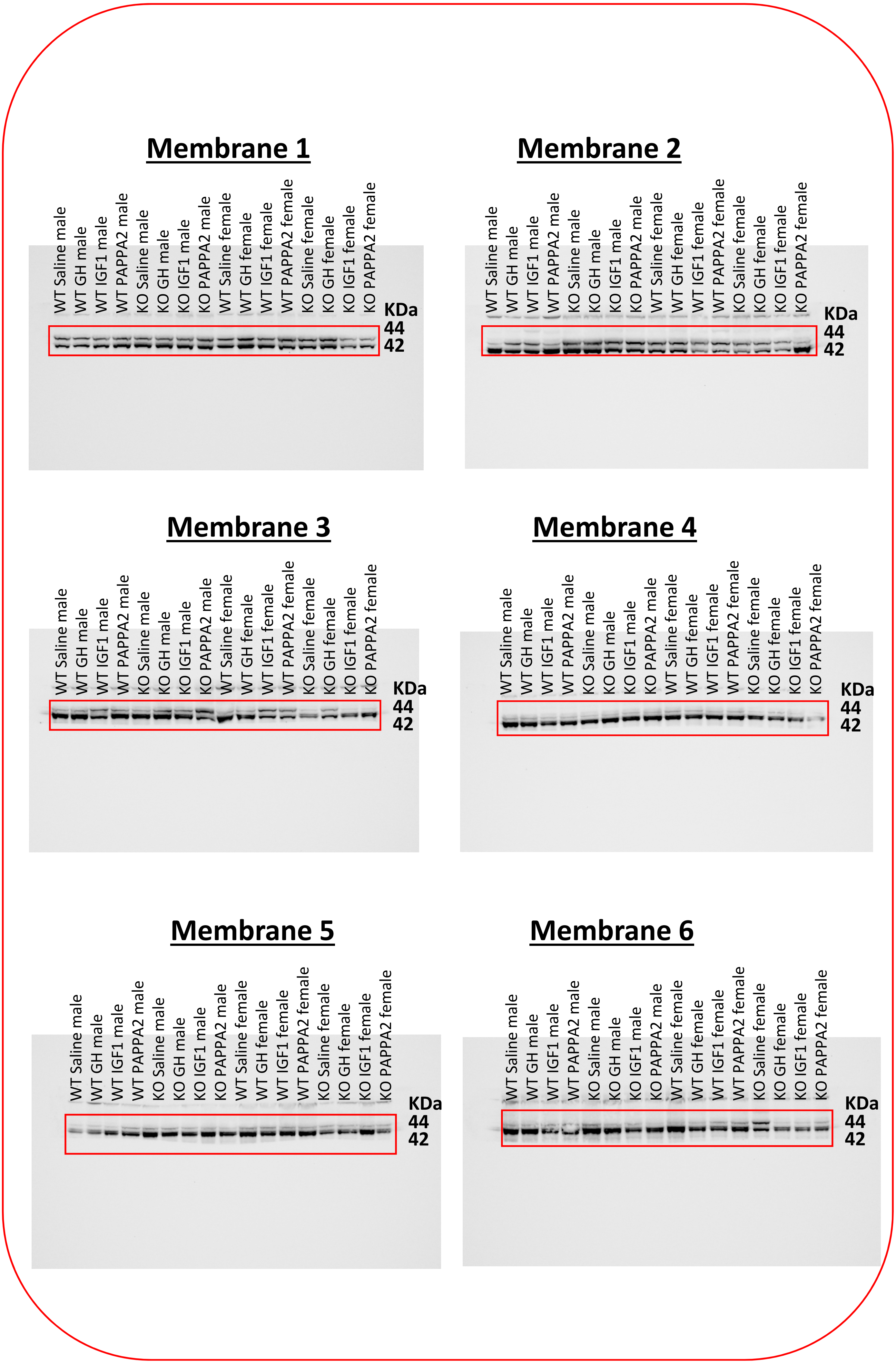

Figure S4.
P. Unedited p(T[202]/Y[204])-ERK1/2 blots in male and female *Pappa2*[ko/ko] after rhGH, rhIGF1 and rhPAPP-A2 treatments

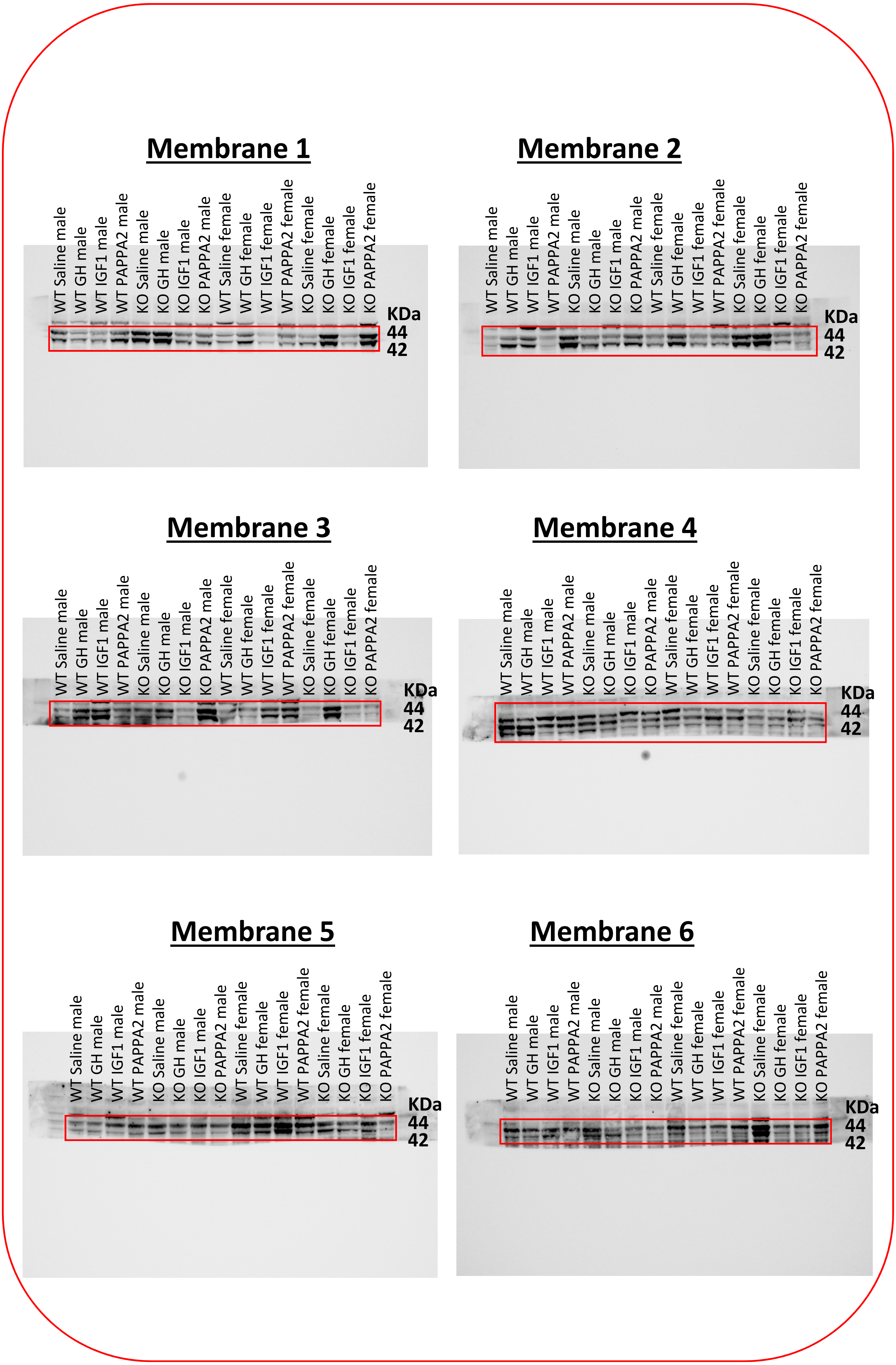

Figure S4
Q. Unedited Adaptin-γ blots in male and female *Pappa2*$^{ko/ko}$ after rhGH, rhIGF1 and rhPAPP-A2 treatments

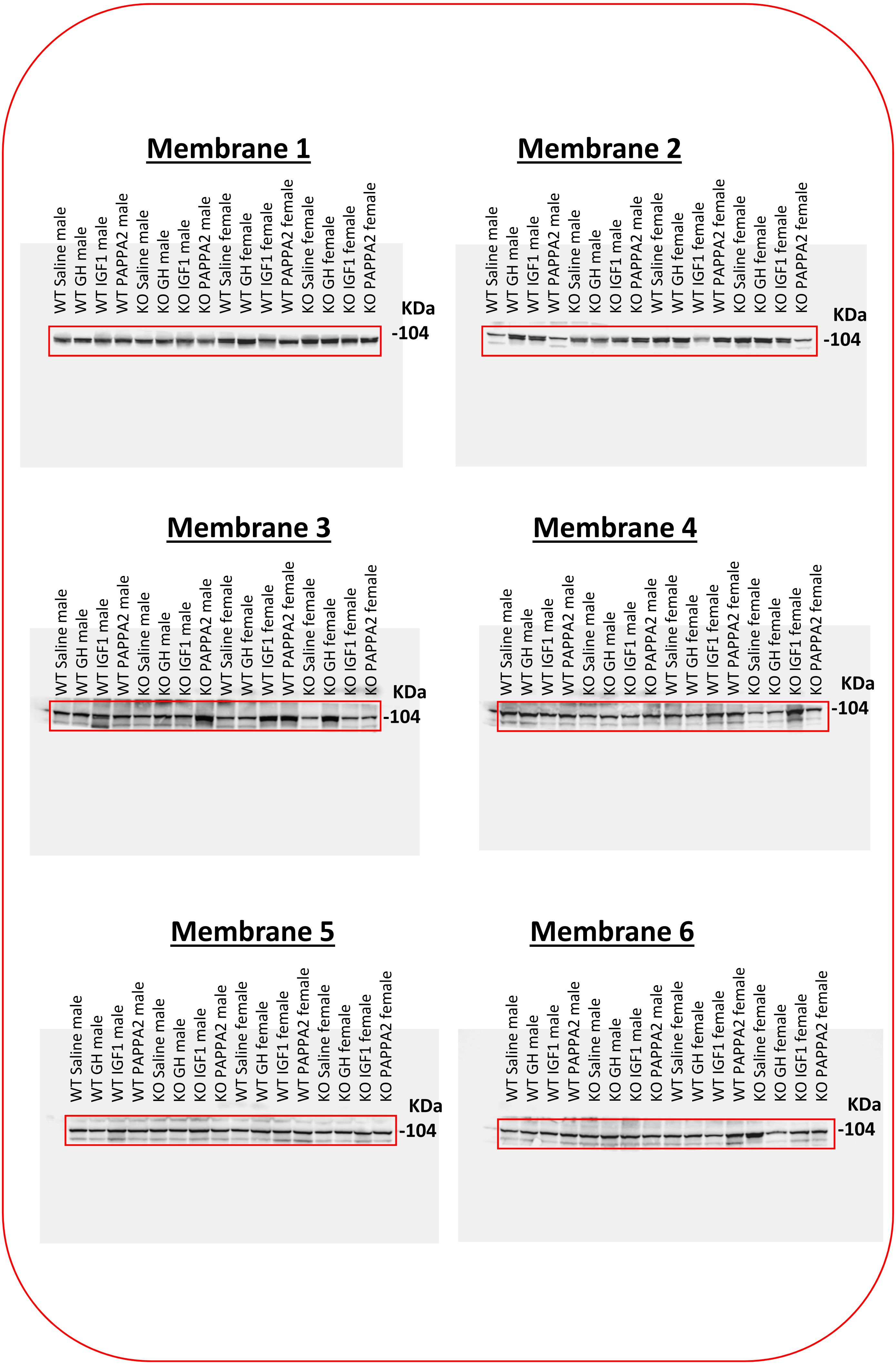

Figure S4.

R. Unedited Jak2 blots in male and female *Pappa2*$^{ko/ko}$ after rhGH, rhIGF1 and rhPAPP-A2 treatments

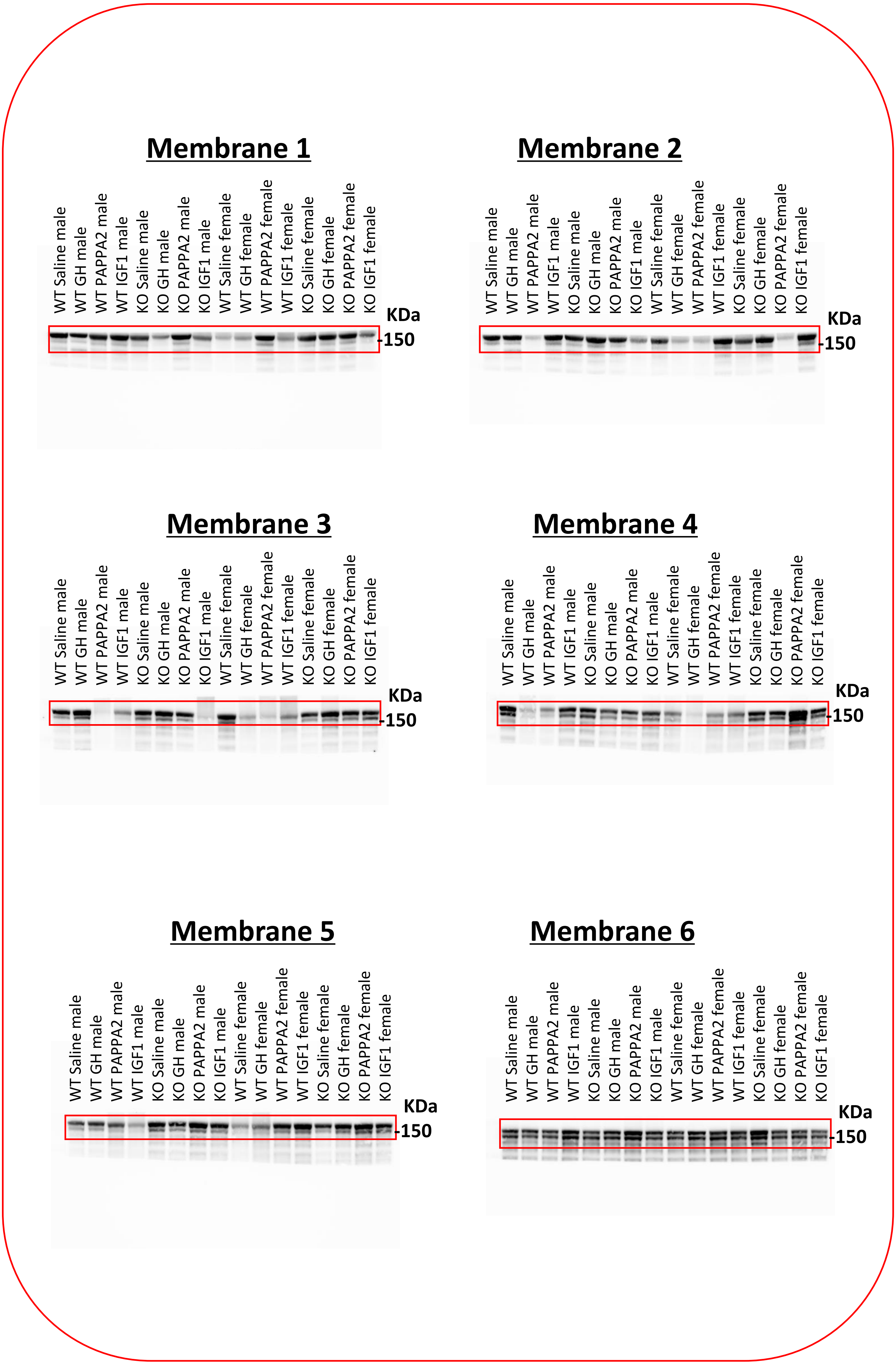

Figure S4.

S. Unedited p(Y^{1008})-Jak2 blots in male and female *Pappa2*$^{ko/ko}$ after rhGH, rhIGF1 and rhPAPP-A2 treatments

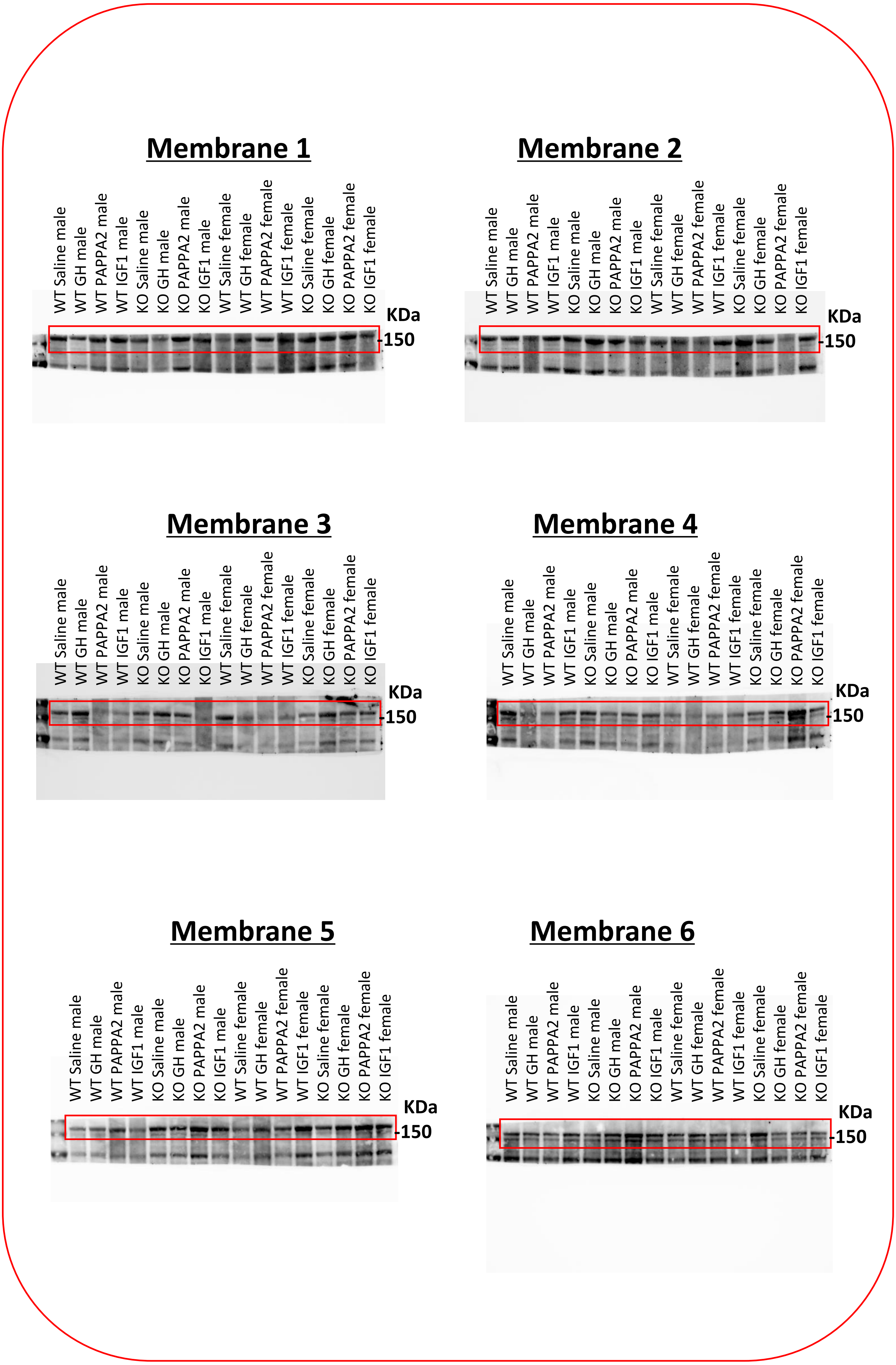

Figure S4.

T. Unedited β-Actin blots in male and female *Pappa2*$^{ko/ko}$ after rhGH, rhIGF1 and rhPAPP-A2 treatments

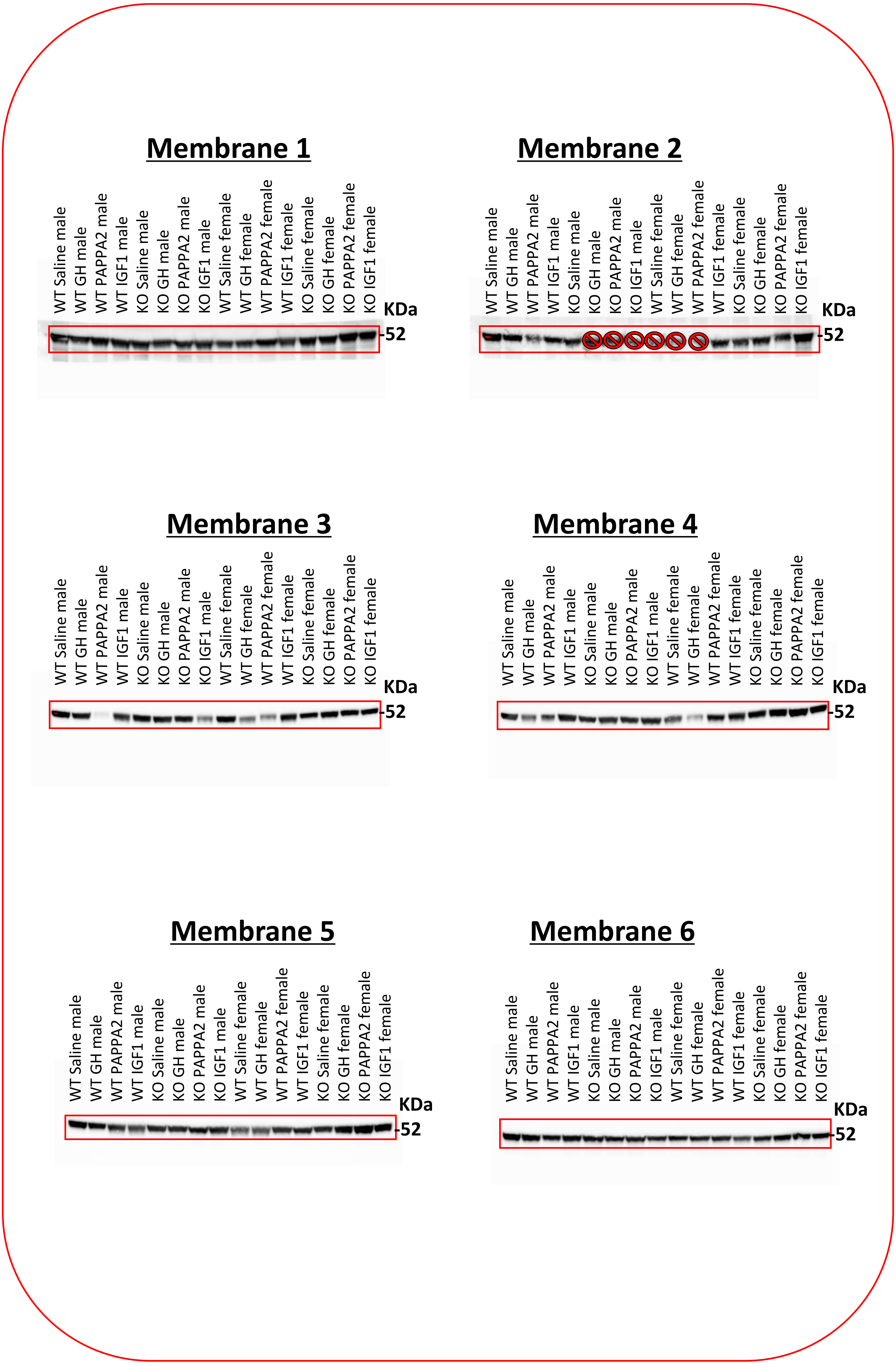

Figure S4.

U. Unedited STAT3 blots in male and female *Pappa2*$^{ko/ko}$ after rhGH, rhIGF1 and rhPAPP-A2 treatments

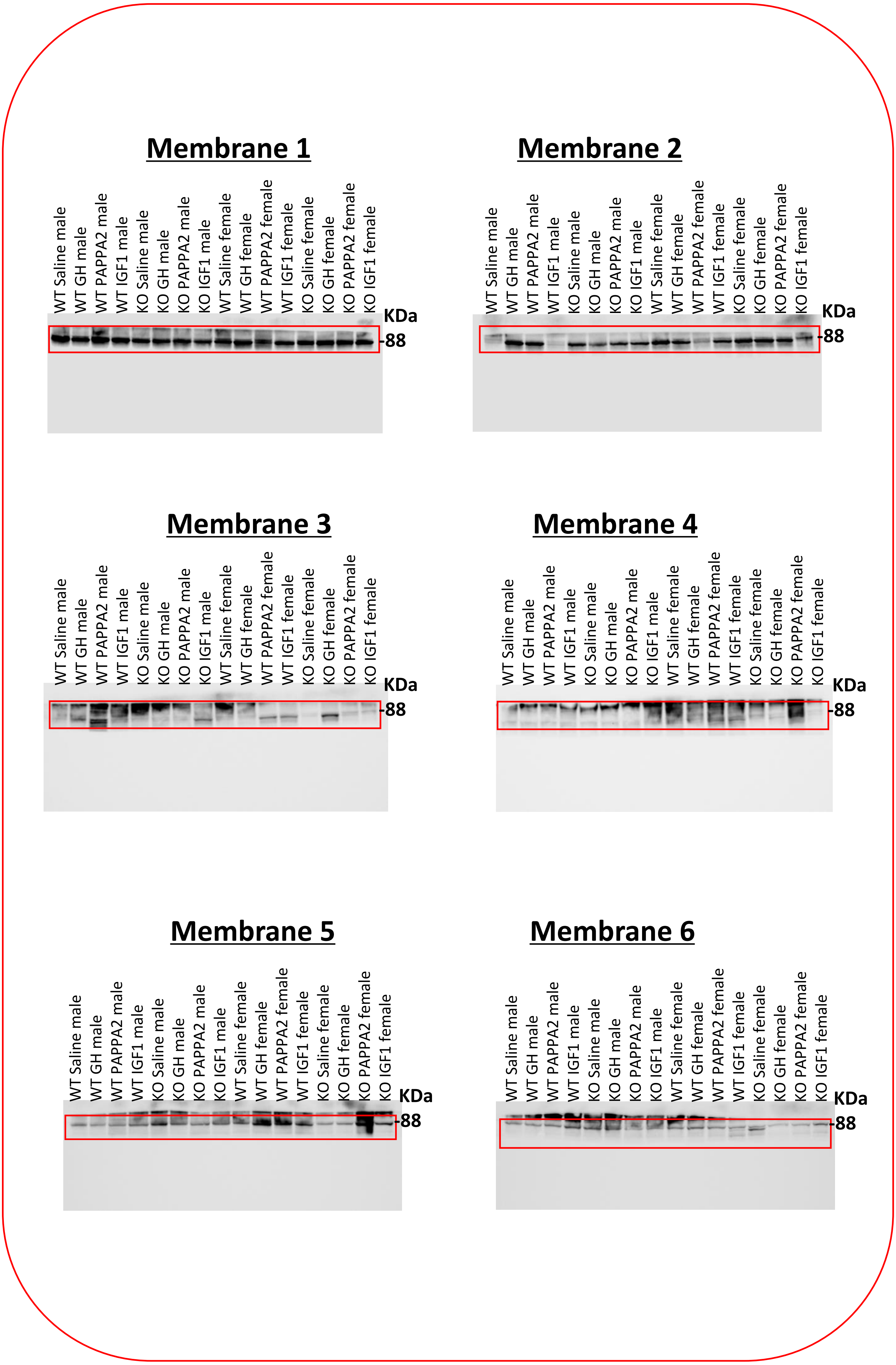

Figure S4.

W. Unedited STAT3-P blots in male and female *Pappa2*$^{ko/ko}$ after rhGH, rhIGF1 and rhPAPP-A2 treatments

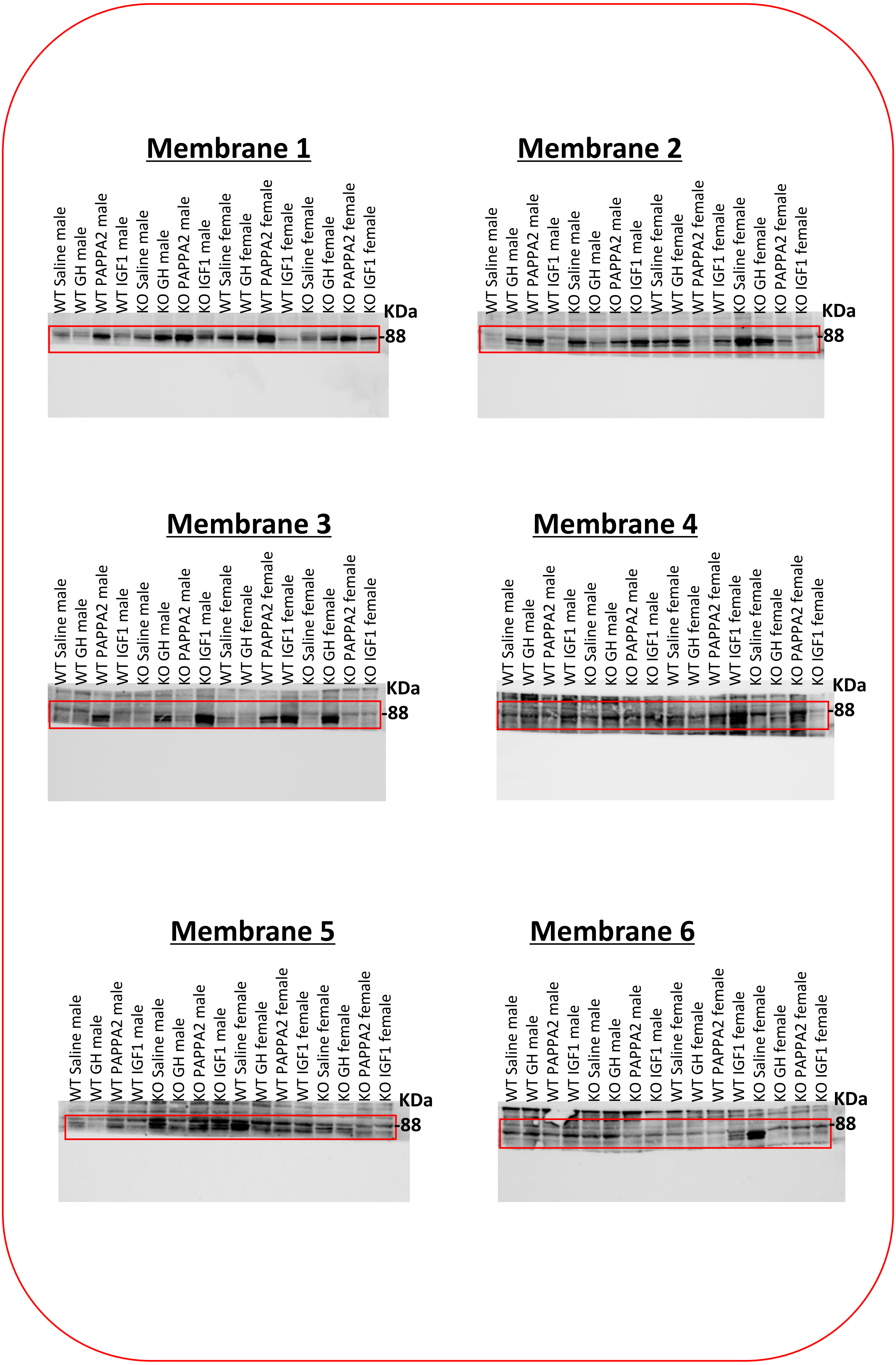

Figure S4.

X. Unedited STAT5 blots in male and female *Pappa2$^{ko/ko}$* after rhGH, rhIGF1 and rhPAPP-A2 treatments

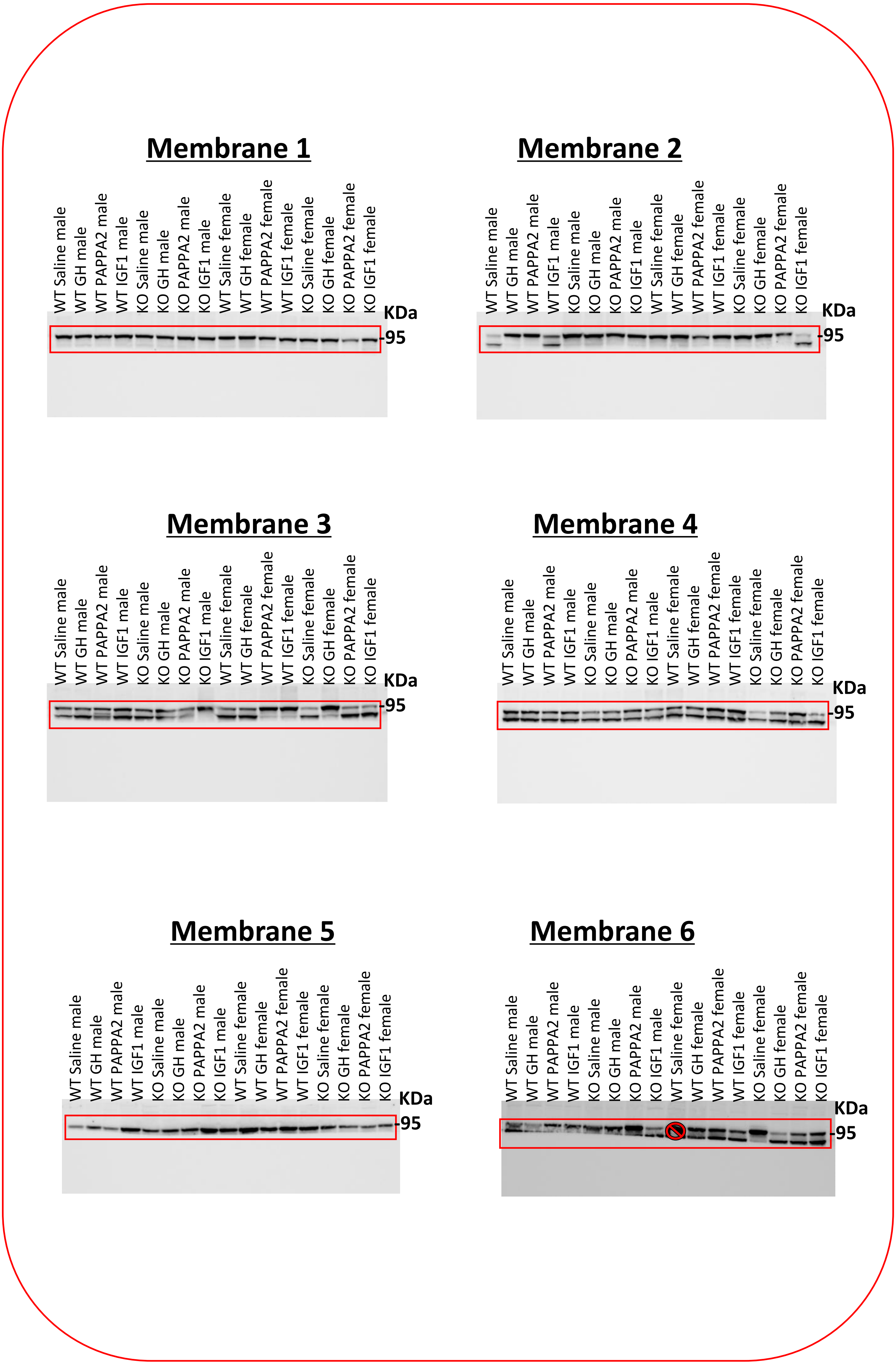

Figure S4.

Y. Unedited STAT5-P blots in male and female *Pappa2*$^{ko/ko}$ after rhGH, rhIGF1 and rhPAPP-A2 treatments

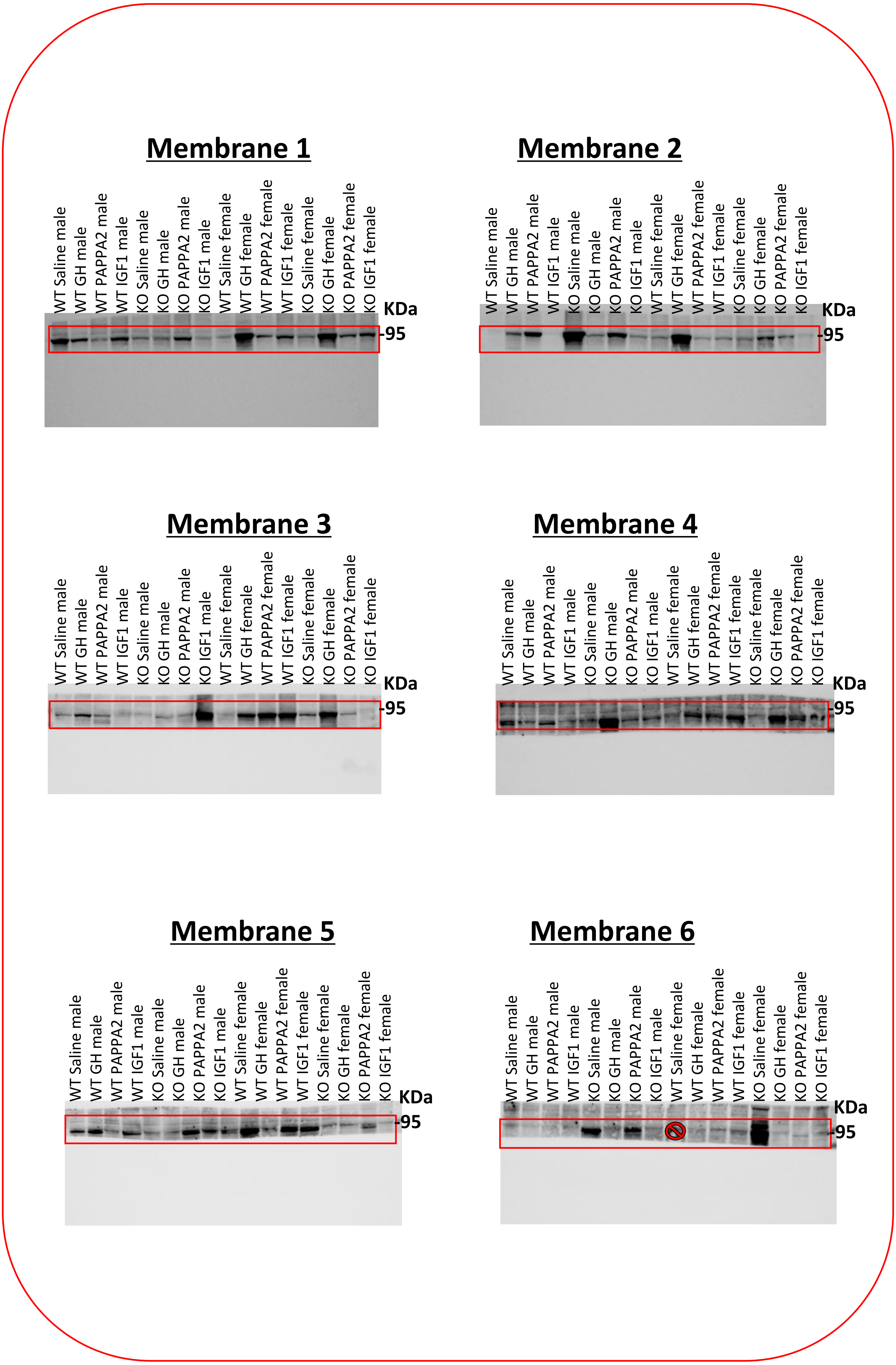

Figure S4.

Z. Unedited γ-Adaptin blots in male and female *Pappa2*$^{ko/ko}$ after rhGH, rhIGF1 and rhPAPP-A2 treatments

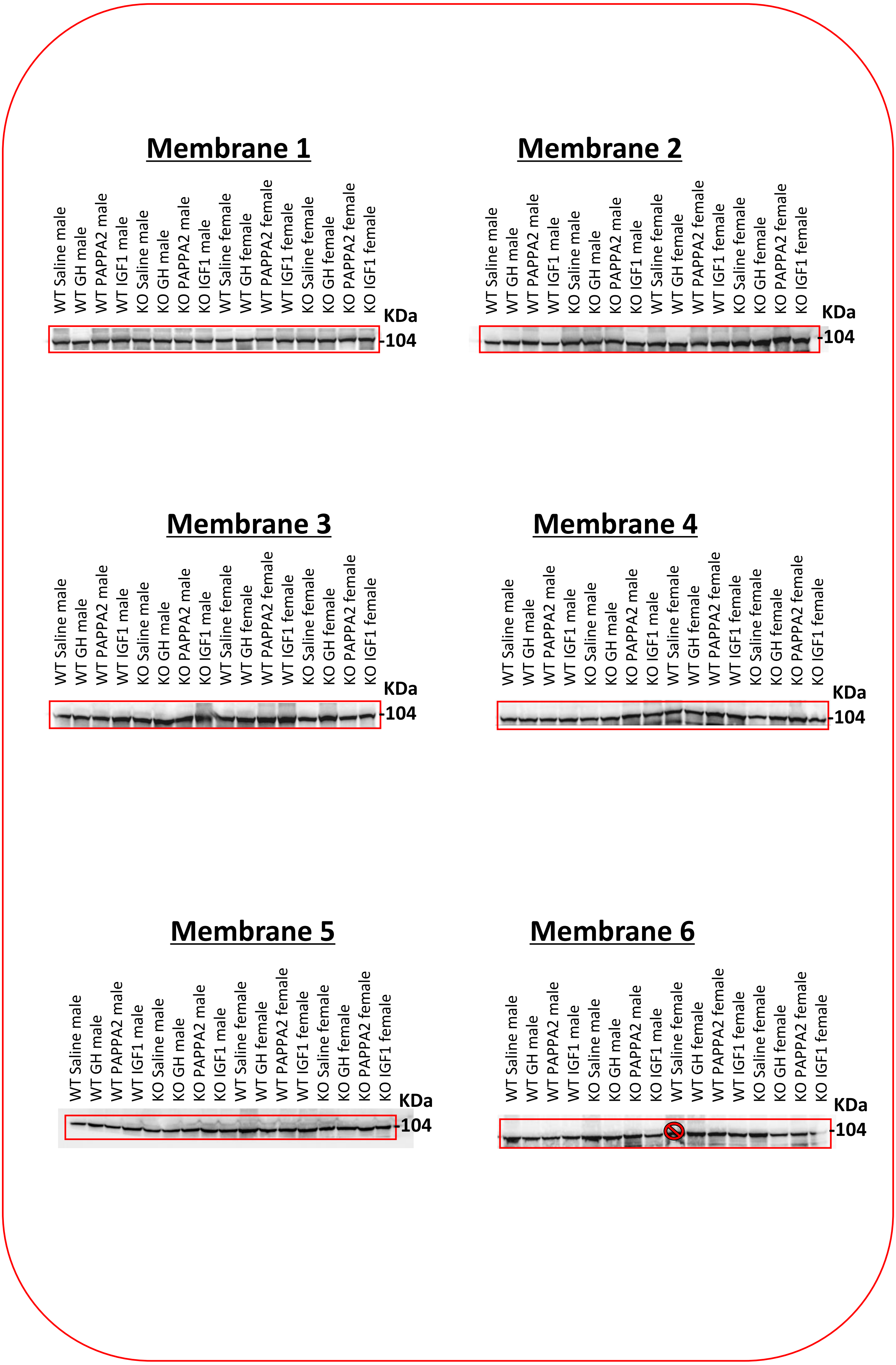

Supplement: Supplementary file 14 — Supplementary Material 14 [file 13293_2024_603_MOESM14_ESM.pdf]
