# Supplementary figures and images for: Sex-based differences in growth-related IGF1 signaling in response to PAPP-A2 deficiency: comparative effects of rhGH, rhIGF1 and rhPAPP-A2 treatments
Source: Biol Sex Differ. 2024 Apr 8;15:34. doi: 10.1186/s13293-024-00603-5 (PMC11000399; doi:10.1186/s13293-024-00603-5)

Figure S5

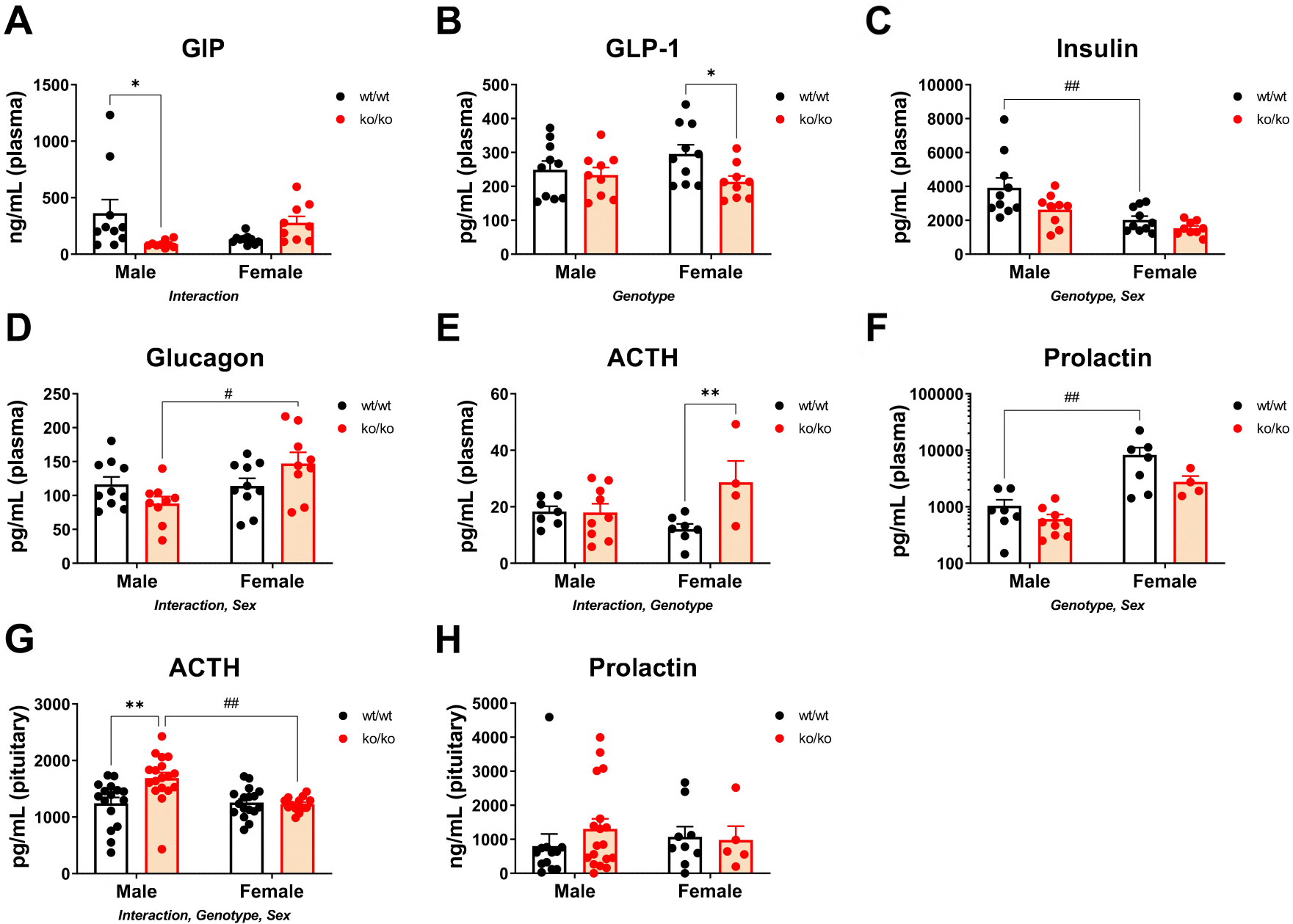

Supplement: Supplementary file 15 — Supplementary Material 15 [file 13293_2024_603_MOESM15_ESM.pdf]

Figure S6

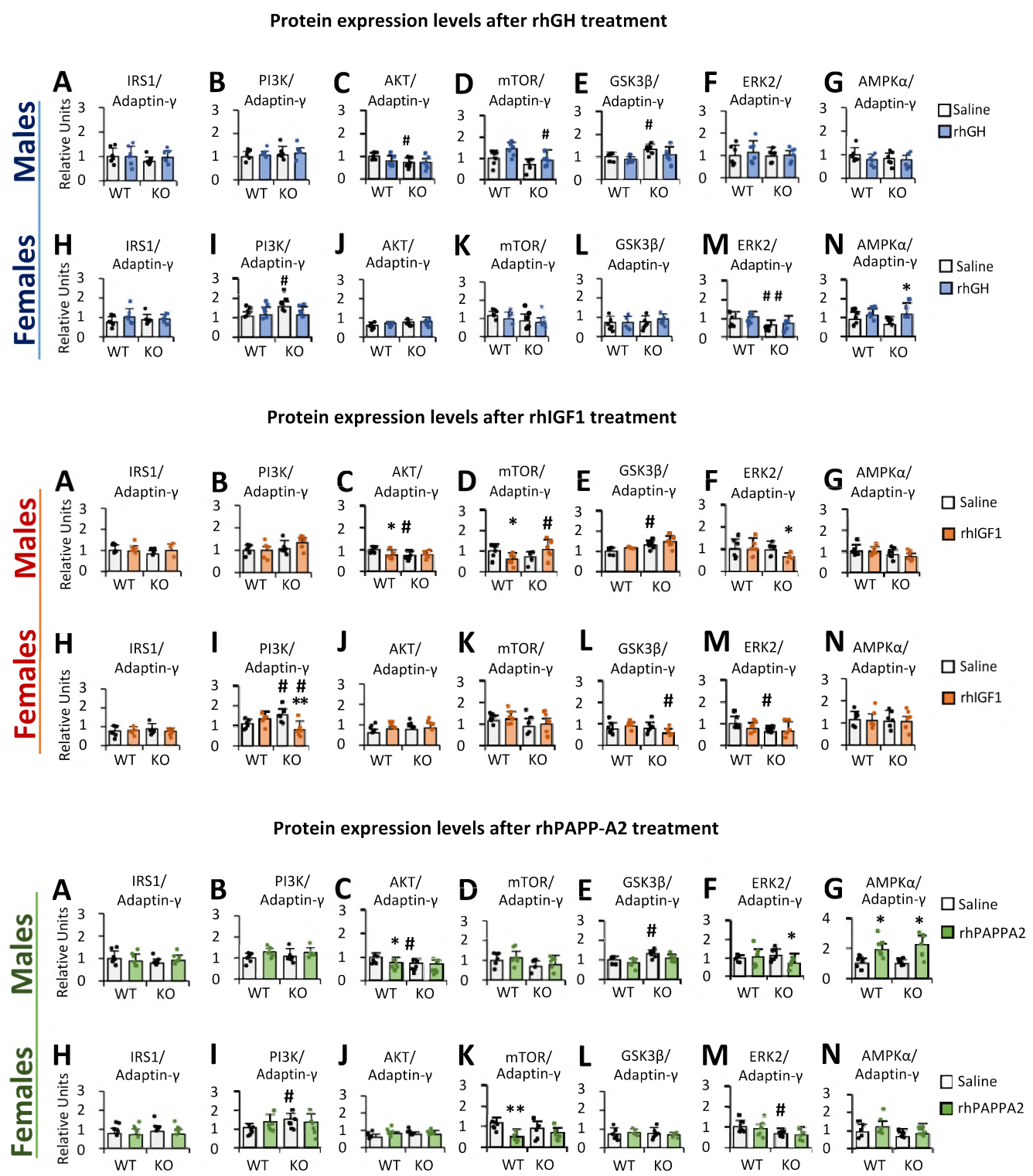

Supplement: Supplementary file 16 — Supplementary Material 16 [file 13293_2024_603_MOESM16_ESM.pdf]

Figure S7

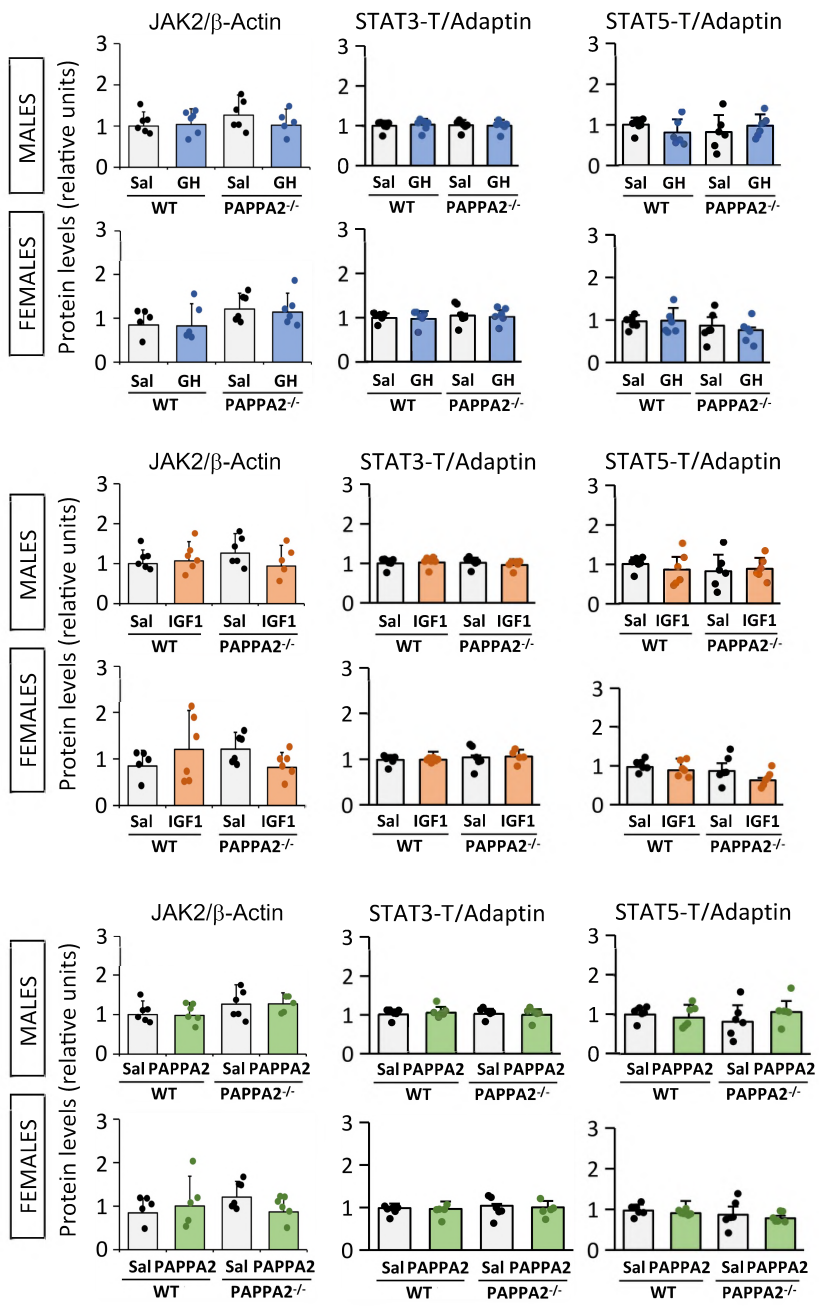

Supplement: Supplementary file 17 — Supplementary Material 17 [file 13293_2024_603_MOESM17_ESM.pdf]
