# Supplementary material for: Sex-based differences in growth-related IGF1 signaling in response to PAPP-A2 deficiency: comparative effects of rhGH, rhIGF1 and rhPAPP-A2 treatments
Source: Biol Sex Differ. 2024 Apr 8;15:34. doi: 10.1186/s13293-024-00603-5 (PMC11000399; doi:10.1186/s13293-024-00603-5)
Supplement: Supplementary file 1 — Supplementary Material 1 [file 13293_2024_603_MOESM1_ESM.docx]

**Juan Suárez, Ph.D.**

Biomedical Research Institute of Malaga

University of Málaga

Bulevar Louis Pasteur 32, 29071 Málaga

Telephone: +34 95133433

E-Mail: juan.suarez@uma.es

September 6, 2023

***Biology of Sex Differences***

Dear Editor-in-Chief:

Please find attached a copy of our manuscript that is now titled “**Sex-based differences in growth-related IGF1 signaling in response to PAPP-A2 deficiency: comparative effects of rhGH, rhIGF1 and rhPAPP-A2 treatments**” by Fernandez-Arjona et al., which we wish to submit for publication in *Biology of Sex Differences*. This manuscript reflects a multidisciplinary collaboration between clinical and preclinical groups.

After receiving the suggestions of the reviewers of BOSD-D-23-00014 and following the recommendations of the EIC, we have incorporated the effects of the chronic pharmacological administration of rhGH, rhIGF1 and rhPAPP-A2 in the resubmitted manuscript. We hope that with this additional information that manuscript can now be considered for publication.

Understanding the physiological role of pregnancy-associated plasma protein-A2 (PAPP-A2), a proteinase involved in the insulin-like growth factor-1 (IGF1) availability to regulate growth, could provide insight into new treatments for patients with short stature and skeletal abnormalities. Although growth and the GH/IGF system are sexually dimorphic in rodents and human and sex differences in these parameters change in an age-specific manner, we know little regarding the underlying differences in the IGF1 system and its signaling pathways. Moreover, here we have included studies demonstrating how males and females respond differently to different physiological treatments, as well as the intracellular signaling pathways that might contribute to growth improvement.

The present study examines whether *Pappa2* deficiency and pharmacological administration of rhGH, rhIGF1 and rhPAPP-A2 are associated with sex-specific differences in IGF1 ternary complexes and growth-related signaling pathways. The main results indicated that:

1. Reduced body and femur length of *Pappa2*^ko/ko^ mice is associated with sex- and tissue-specific alteration of IGF ternary/binary complexes and IGF1 signaling pathways.
2. Compared to rhGH and rhIGF1, rhPAPP-A2 administration for 30 days induced a female-specific increase in body and femur length and a reduction in IGF ternary/binary complexes through AKT-ERK-AMPK signaling pathways in liver.

The involvement of PAPP-A2 in sex-based growth physiology supports the use of promising drugs to alleviate postnatal growth retardation underlying low IGF1 bioavailability in a female-specific manner.

We hope that the new version of the manuscript can be considered for publication in *Biology of Sex Differences*.

Sincerely,

Jesús Argente, M.D., PhD., Professor

Juan Suárez, PhD., Associate Professor

Patricia Rivera, PhD., Senior Researcher
